# Development of Bifunctional, Raman Active Diyne‐Girder Stapled α‐Helical Peptides**

**DOI:** 10.1002/chem.202300855

**Published:** 2023-06-13

**Authors:** Danielle C. Morgan, Laura McDougall, Astrid Knuhtsen, Andrew G. Jamieson

**Affiliations:** ^1^ School of Chemistry Advanced Research Centre University of Glasgow 11 Chapel Lane Glasgow G11 6EW UK

**Keywords:** α-helical, bifunctional, protease stable, Raman active, stapled peptide

## Abstract

Stapled peptides are a unique class of cyclic *α*‐helical peptides that are conformationally constrained via their amino acid side‐chains. They have been transformative to the field of chemical biology and peptide drug discovery through addressing many of the physicochemical limitations of linear peptides. However, there are several issues with current chemical strategies to produce stapled peptides. For example, two distinct unnatural amino acids are required to synthesize *i*, *i*+7 alkene stapled peptides, leading to high production costs. Furthermore, low purified yields are obtained due to *cis*/*trans* isomers produced during ring‐closing metathesis macrocyclisation. Here we report the development of a new *i*, *i*+7 diyne‐girder stapling strategy that addresses these issues. The asymmetric synthesis of nine unnatural Fmoc‐protected alkyne‐amino acids facilitated a systematic study to determine the optimal (*S*,*S*)‐stereochemistry and 14‐carbon diyne‐girder bridge length. Diyne‐girder stapled T‐STAR peptide **29** was demonstrated to have excellent helicity, cell permeability and stability to protease degradation. Finally, we demonstrate that the diyne‐girder constraint is a Raman chromophore with potential use in Raman cell microscopy. Development of this highly effective, bifunctional diyne‐girder stapling strategy leads us to believe that it can be used to produce other stapled peptide probes and therapeutics.

## Graphical abstract

**Stapled peptides**: The development of an *i*, *i*+7 diyne‐girder stapling strategy that produces α‐helical, protease stable, cell permeable and Raman active stapled peptides.

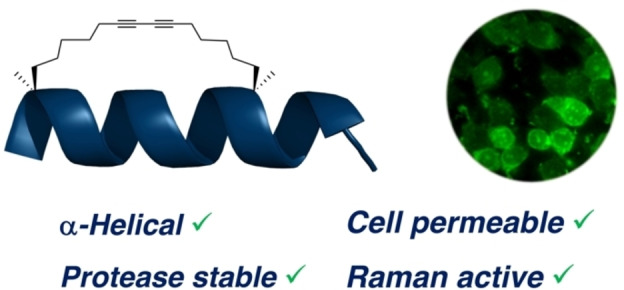

## Introduction

Stapled peptides have come of age as tool compounds in chemical biology for the regulation of protein‐protein interactions (PPIs) and as lead compounds in drug discovery, with several stapled peptides progressing through clinical trials.[^1^, ^2^] Conformationally constrained stapled peptides occupy a Goldilocks zone with physicochemical properties between small molecules and biologics. They have also been demonstrated to have relatively safe metabolites and have the ability to avoid drug resistance through extended drug target interfaces, making them attractive drug candidates.[^3^, ^4^]

PPIs were regarded as undruggable due to the difficulty in targeting extended, flat interaction motifs.^[5]^ Stapled peptides have the ability to mimic one of the protein epitopes, bind to a topologically shallow surface of the protein partner, and thus selectively disrupt these molecular recognition events.[^6^, ^7^, ^8^, ^9^, ^10^] In addition, stapled peptides overcome some of the physicochemical inadequacies of native peptides. Constraining a peptide into a helical conformation decreases the polarity through the adoption of an extended intramolecular hydrogen bonding network between the NH and carbonyl of the backbone amide functionality.^[11]^ Stapled peptides are thus less polar and have improved cell‐permeability in comparison to their linear peptide analogues.^[12]^ Conformationally constrained peptides also adopt their helical bioactive secondary structure, paying the entropic penalty of folding, which results in favourable binding affinity and improved selectivity for their target protein. Stapled peptides also have improved blood serum stability, compared with their linear peptide analogues, due to their reduced ability to adopt the linear conformation required for protease enzyme substrate recognition.[^13^, ^14^]

Stapled peptides refer specifically to conformationally constrained α‐helical peptides which are covalently linked through their amino acid side‐chains. The most common synthetic strategy is hydrocarbon stapling. This involves cyclisation of two unnatural amino acids featuring terminal alkenes using a key ring closing metathesis (RCM) reaction. The method was first reported by H. Blackwell and R. Grubbs in 1998. In 2000, Schafmeister et al. developed an all‐hydrocarbon alkene stapling method with optimal stereochemistry and linker length for *i*, *i*+3, *i*, *i*+4 and *i*, *i*+7 constraints.[^14^, ^15^]

Although the *i*, *i*+4 hydrocarbon staple is most reported, the *i*, *i*+7 constraint is more effective at constraining extended peptides into an *α*‐helical conformation. However, there are several issues with the design of the *i*, *i*+7 hydrocarbon staple. The procedure relies on two different amino acid building blocks, (*R*)‐*N*‐Fmoc‐*α*‐(7‐octenyl)alanine (*R*_8_) and (*S*)‐*N*‐Fmoc‐*α*‐(4‐pentenyl)alanine (*S*_5_) resulting in significant cost of starting materials. Macrocyclisation of these two amino acids in the *i*, *i*+7 positions of the peptide by RCM using Grubbs’ catalyst results in a mixture of *cis* and *trans* alkene isomers.^[16]^ The *trans* alkene is the more effective *i*, *i*+7 constraint and thus this isomeric mixture results in unsatisfactory yield. A third, more general issue with stapled peptides is that for cell imaging a chromophore must be appended to the peptide, changing the physicochemical properties.[^17^, ^18^]

Recently, Dawson and co‐workers have reported diyne peptide macrocycles using mono‐substituted propargyl serine amino acids.^[19]^ Limited helicity was observed for these peptides, a key objective in this work. Ballet and co‐workers later reported the development of the same type of constraint produced via a combination of side chain propargylated D‐and L‐Ser residues (*i*, *i*+7) and subsequent macrolactamization.^[20]^ Significantly, epimerisation of the staple amino acids was observed during synthesis. Dawson and co‐workers have also reported incorporation of an *i*, *i*+2 diyne constraint to construct stretched β‐strand mimics.^[21]^ The rigid diyne brace allowed the peptides to adopt stable conformations in solution, and could be generated in excellent conversions. Notably, this reported staple type differs from this work, exhibiting different amino acid positioning, peptide secondary structure and finally does not include α‐methyl carbons.

With these issues in mind, we set out to develop a new peptide stapling strategy that would involve straightforward chemistry, act as an effective conformational constraint and incorporate a chromophore for cell imaging.

**Figure 1:**
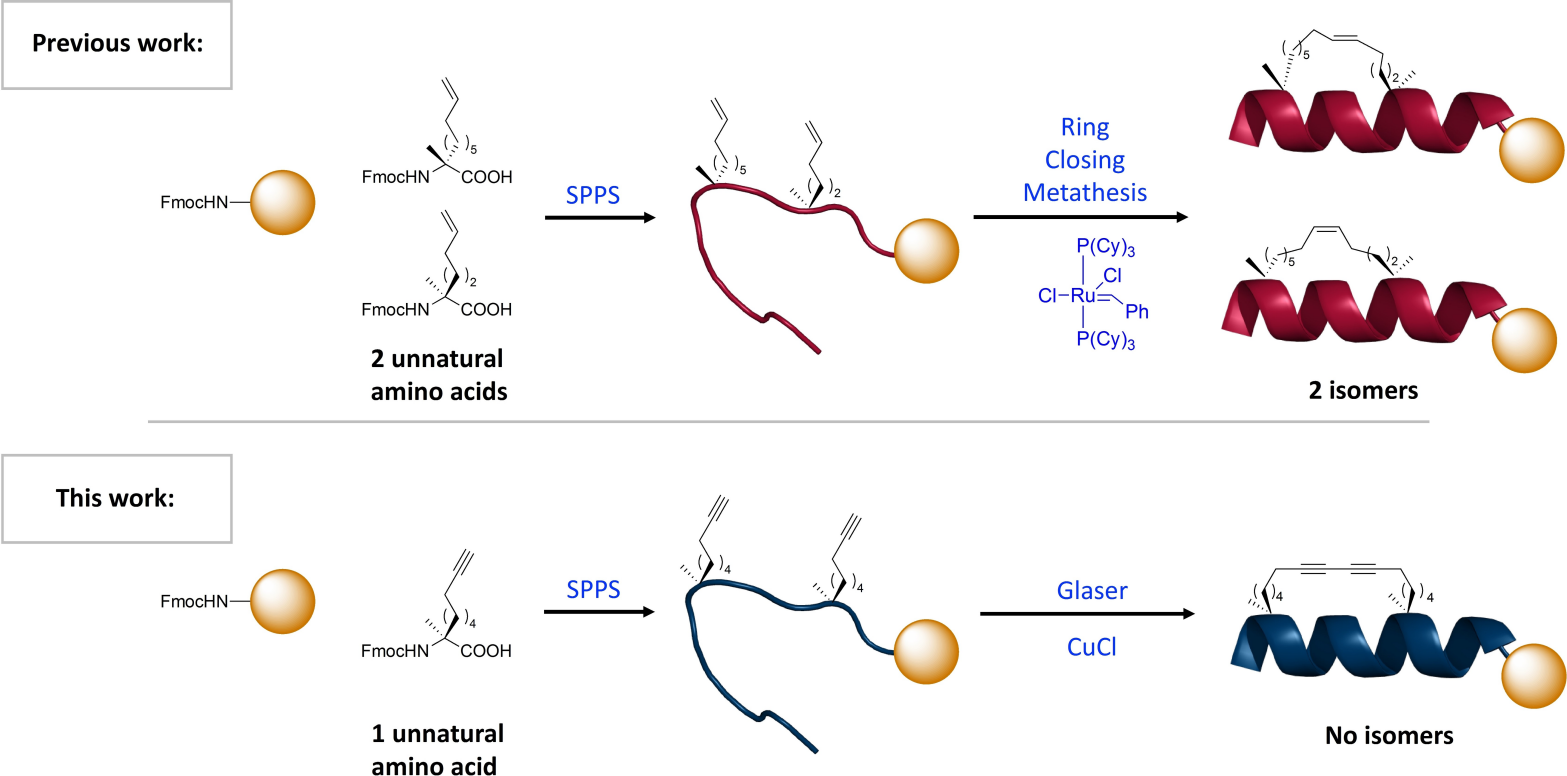
Previous all‐hydrocarbon *i*, *i*+7 stapling technique requires two distinct amino acids and results in a mix of *cis*/*trans* isomers. This work demonstrates the use of one amino acid type to create a symmetrical, rigid diyne constraint, resulting in improved yield, cheaper starting material costs, improved α‐helicity and protease stability, and is a Raman chromophore with potential for cell imaging.

In this work we describe the development of 1,3‐diyne‐girder constrained stapled peptides (Figure 1). We call this constraint a “girder” due to the similarities with steel girders used in the construction industry for building bridges. The method requires a single type of amino acid building block that can be used to prepare the diyne‐girder constraint via a Glaser oxidative coupling reaction. Biologically relevant peptides were synthesised containing the diyne‐girder and alkene constraints, showing enhanced degrees of helicity compared to the native peptide counterpart. In addition, we demonstrate using fluorescence microscopy that these diyne‐girder and alkene stapled peptides are cell penetrating, unlike the native peptide. Finally, we exhibit that the diyne‐girder constraint also acts as a Raman chromophore and has the potential to be imaged using confocal Raman imaging to study peptide cell permeability.

## Results and Discussion

Our initial design criteria were born from the observation that in many examples alkene hydrocarbon staples linking the distant amino acid residues is too flexible. This flexibility results in an ineffective conformational constraint, hence limiting the degree of helicity of the stapled peptide. Our central hypothesis was that a highly rigid side‐chain to side‐chain 1,3‐diyne bridge will act as a more effective conformational constraint. Providing peptides with high degrees of α‐helical structure, improved binding affinities and physicochemical properties.

### Asymmetric synthesis of unnatural alkyne amino acids

**Scheme 1:**
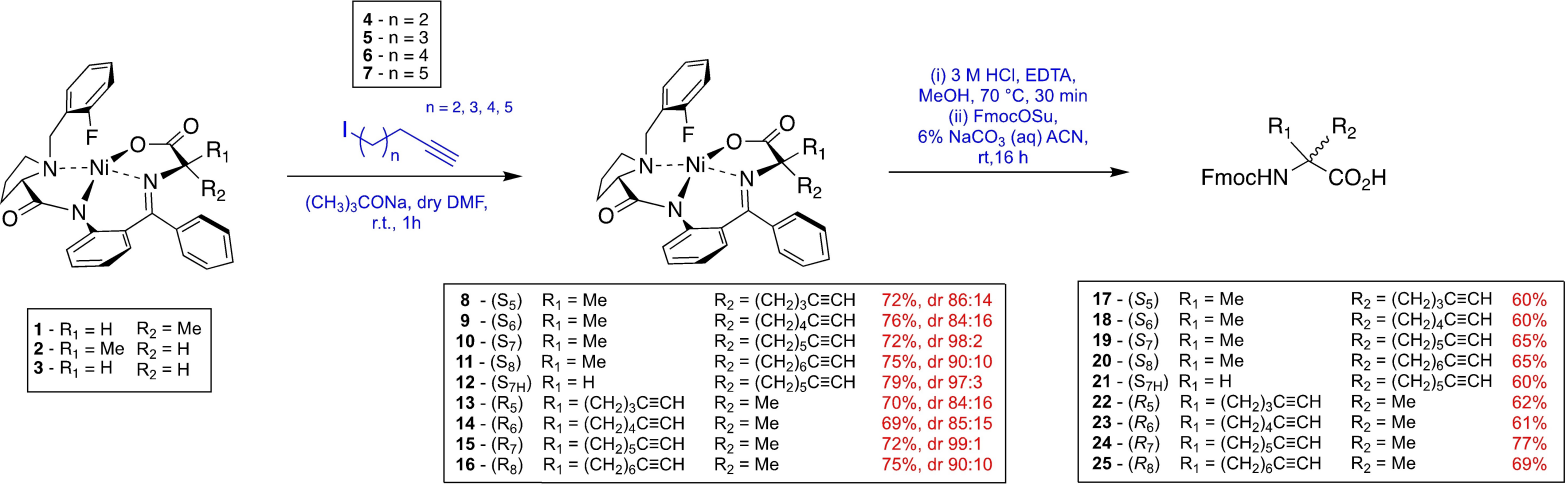
The synthesis of Fmoc‐alkyne amino acids with varying stereochemistry and length using the Ni(II) Schiff Base method.

Our first objective was to develop an asymmetric synthesis of the unnatural alkyne amino acids required to investigate the optimal diyne‐girder bridge length. Incorporation of a methyl group onto the α‐carbon of amino acids invokes a Thorpe‐Ingold effect to enhance a helical peptide conformation and provides additional protease stability.[^22^, ^23^] We previously reported a robust method for the production of α‐methyl‐α‐disubstituted amino acids based on the Belokon Ni(II) Schiff base complex.^[24]^ Synthesis of alkyne amino acids **17**–**25** was achieved using a modified version of this method (Scheme 1). The Ni(II) Schiff base complex **1** & **2** was prepared as described previously.^[25]^ The appropriate alkyne halide electrophiles were either purchased or prepared *via* a zipper reaction to provide the alcohols and then a Finkelstein reaction (see Supporting Information Table S2).^[26]^ Alkylation of Ni(II) Schiff base complex **1** & **2** was achieved under basic conditions to provide complexes **8**–**16** in excellent yields and diastereoselectivities. The diastereomeric ratio (*dr*) of the crude mixture was determined using ^19^F NMR spectroscopy. Decomplexation of alkylated complexes **8**–**11** was achieved under acidic conditions, using EDTA to chelate the nickel by‐product. Fmoc protection was achieved using Fmoc‐OSu under basic conditions to generate the desired (*S*)‐Fmoc amino acids **17**–**20** (Scheme 1). Amino acids with *R*‐configuration were synthesised from (*R,R)‐*Ni(II) Schiff Base complex **2** to generate (*R*)‐Fmoc protected amino acids **22**–**25**. The amino acid with *S*‐configuration and no α‐methyl carbon was synthesised from Ni(II) Schiff Base complex **3** to generate (*S*)‐Fmoc protected amino acid **21**.

### Bridge length and stereochemical requirements for diyne‐girder staple using biologically relevant sequences

**Scheme 2:**
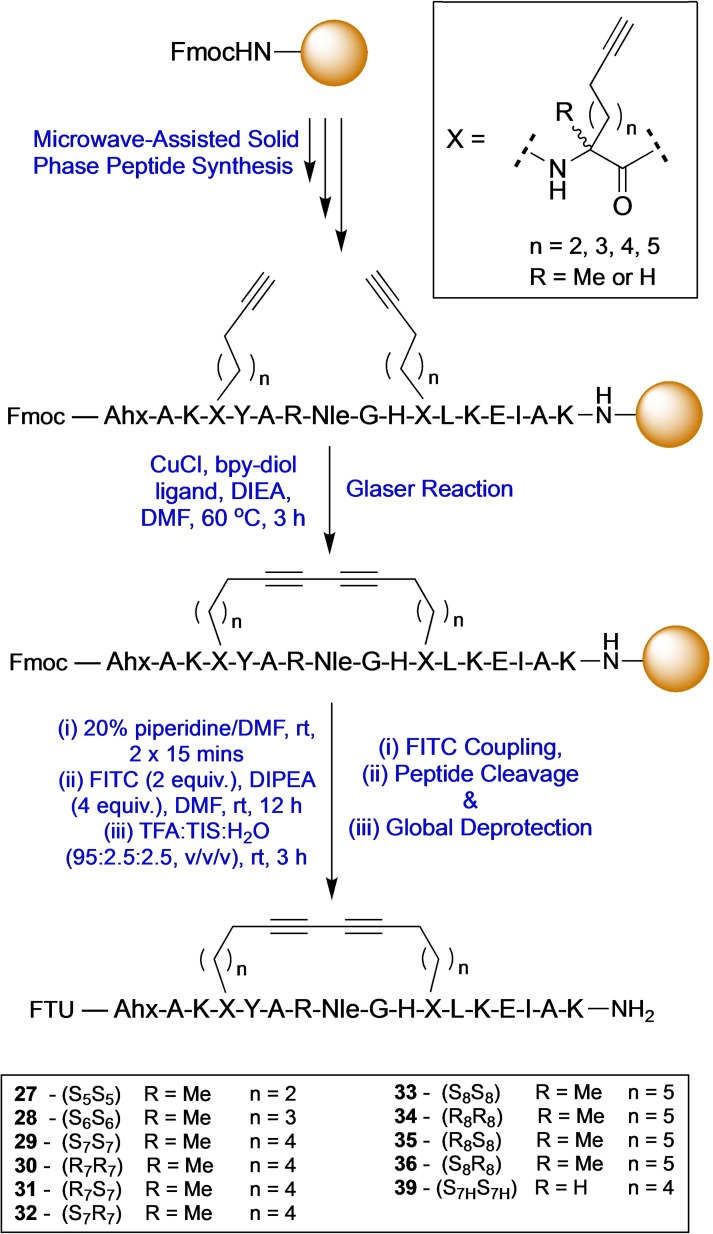
Solid phase peptide synthesis of diyne‐girder stapled peptides. Nle=norleucine. Ahx=6‐aminohexanoic acid. FTU=Fluorescein thiourea label. *n*=number of carbons.

**Figure 2:**
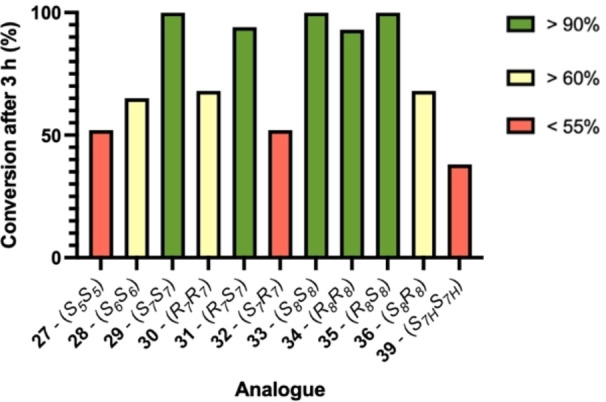
Percentage Glaser cyclisation conversion for 1,3‐diyne‐girder stapled peptides with different bridge lengths and alkyne amino acid stereochemistries.

The RNA binding protein T‐STAR regulates the alternative splicing of neuronal pre‐mRNAs.^[27]^ T‐STAR forms a functionally active homodimer with an α‐helix at the interface. This peptide epitope sequence was therefore chosen as a model system to determine the optimal diyne‐girder bridge length required to provide an effective conformational constraint. Initially, native T‐STAR **26** and thirteen linear T‐STAR peptide analogues **27**–**39** were prepared using microwave‐assisted solid‐phase peptide synthesis (SPPS) using a Fmoc/^t^Bu protection strategy on Rink amide ChemMatrix resin (Scheme 2). Peptides were synthesised incorporating two C_7_ or C_8_ alkyne amino acid building blocks, positioned *i*, *i*+7 to one another, to provide a C_14_ or C_16_ hydrocarbon bridge. Both stereoisomers of the amino acids were applied to provide all four stereoisomeric combinations of each bridge length: *SS*, *SR*, *RR* and *RS*. Glaser reaction conditions (Scheme 2 – CuCl, DIEA, Bpy‐diol), previously described by Dawson and co‐workers, or RCM conditions (Grubbs 1^st^ gen. catalyst) were applied to cyclise the peptides on resin.^[19]^ Conversion to the macrocyclised stapled product was determined by analytical reverse‐phase high performance‐liquid chromatography (RP‐HPLC) and high‐resolution mass spectrometry (HR‐MS) of a cleaved sample, with cyclised products typically eluting with longer retention times. Complete conversion was observed for combinations *R*_8_ *S*_8_, *S*_8_ *S*_8_ and *S*_7_ *S*_7,_ and good conversion (90 %) for *R*_8_ *R*_8_ and *R*_7_S_7_ (Figure 2)_._ However, lower conversions of 68 % and 52 % were obtained for *S*_8_ *R*_8_ and *S*_7_ *R*_7_ respectively. Based on these data, it is clear that both C_16_ and C_14_ bridges are synthetically feasible for this constraint. The *SS* combination of amino acids is favoured as it gave the best conversion for both bridge lengths, and only requires one type of unnatural amino acid.

To establish the optimal bridge length and determine at what point the bridge is too strained to induce an α‐helix, peptides with a C_10_ **27** or C_12_ **28** bridge were synthesised using two *S*_5_ and two *S*_6_ amino acids respectively. Interestingly, a decrease in conversion for the Glaser coupling was observed for both of these peptides with shorter bridge lengths suggesting steric strain has a detrimental effect on macrocyclisation (Figure 2). From this data, it was concluded that the *S*,*S*‐C_14_ bridge is optimal for an *i*, *i*+7 diyne‐girder staple because it is the shortest constraint with a high degree of conversion in the Glaser reaction and only requires one type of unnatural alkynyl amino acid.

### Conformational analysis of diyne‐girder stapled peptides

**Figure 3:**
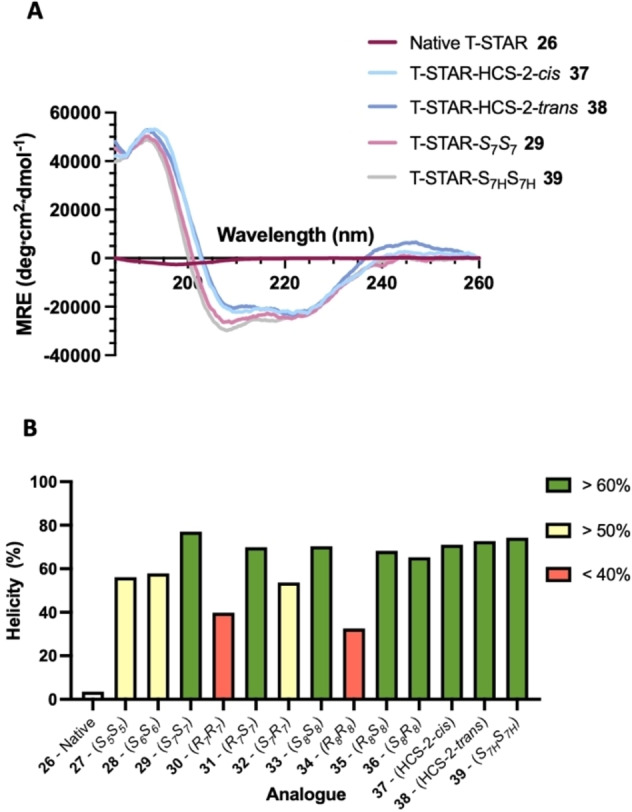
Circular dichroism spectra of native peptide **29** and stapled peptides **27**–**39**. A) CD shown as MRE, B) % peptide helicity at 222 nm. Conditions: peptides 50 μM in PBS, pH 7.4. Spectra recorded between 185 and 260 nm.

The diyne‐girder conformational constraint is designed to induce peptides to form their bioactive α‐helical secondary structure. Conformational analysis was achieved using circular dichroism (CD) spectroscopy to assess the effect of the diyne‐girder staples on the structure of peptides **27**–**38**. Control hydrocarbon stapled peptides **37** (*cis* alkene) and **38** (*trans* alkene) were prepared and analysed for comparison. Peptide **39** incorporates monosubstituted amino acids (S_7H_), providing insight into the role of the α‐methyl group of the unnatural amino acid. CD analysis was undertaken at a concentration of 50 μM, scanning from 185 to 260 nm and is presented as the percentage helicity at 222 nm from mean residue ellipticity (Figure 3 and Supporting Information Figures S1–S3). The control linear native T‐STAR peptide has a very low percentage helicity under these conditions as expected. Three of the constraints (**33**, **35** & **36**) with C_16_ bridge length have high percentage helicity. However, the C_16_ analogue with alkyne amino acid stereochemistry *RR* **34** gave a much lower percentage helicity (33 %).

Similar helicity was observed for the C_14_ bridge analogues with *SS* (77 %) and *RS* (70 %) stereochemistry. The *SR* (54 %) analogue had a lower helicity and the *RR* (40 %) had the lowest helicity of the C_14_ bridge analogues. Interestingly, the C_14_‐S_7H_ analogue **39** of this peptide, with monosubstituted unnatural amino acids, retained comparable helicity (74 %) compared with the α‐methyl amino acid analogue **29** (77 %). These data suggest that, although favourable for facilitating the Glaser macrocyclisation reaction, the α‐methyl functionality has limited effect on the structure of this T‐STAR stapled peptide. A comparison between the C_14_‐*SS*, C_12_‐*SS* and C_10_‐*SS* bridged peptides showed a decreasing trend in helicity. This is most apparent at 190 nm where the MRE value for C_12_ and C_10_ is around half that of C_14_. This result suggests that both the C_12_ and C_10_ constraints are restricting helix formation of the peptide due to increased strain from the shorter bridge lengths. It was concluded from these data that an *S*_7_ *S*_7_ bridge is the optimum in terms of helicity and with the added benefit of requiring only a single type of unnatural amino acid.

### Proteolytic stability of native and stapled T‐STAR peptides

Stapled peptides are constrained into their bioactive α‐helical secondary structure and have increased proteolytic stability because the linear conformation required to engage protease enzyme active sites is restricted. To establish whether incorporation of our diyne‐girder staple increases peptide stability to protease enzymes, a stability study was undertaken. Chymotrypsin was chosen for the experiment as it typically cleaves at the *C*‐terminus of aromatic residues and there is a single Tyr located within the centre of the T‐STAR peptide sequence.^[28]^

**Figure 4:**
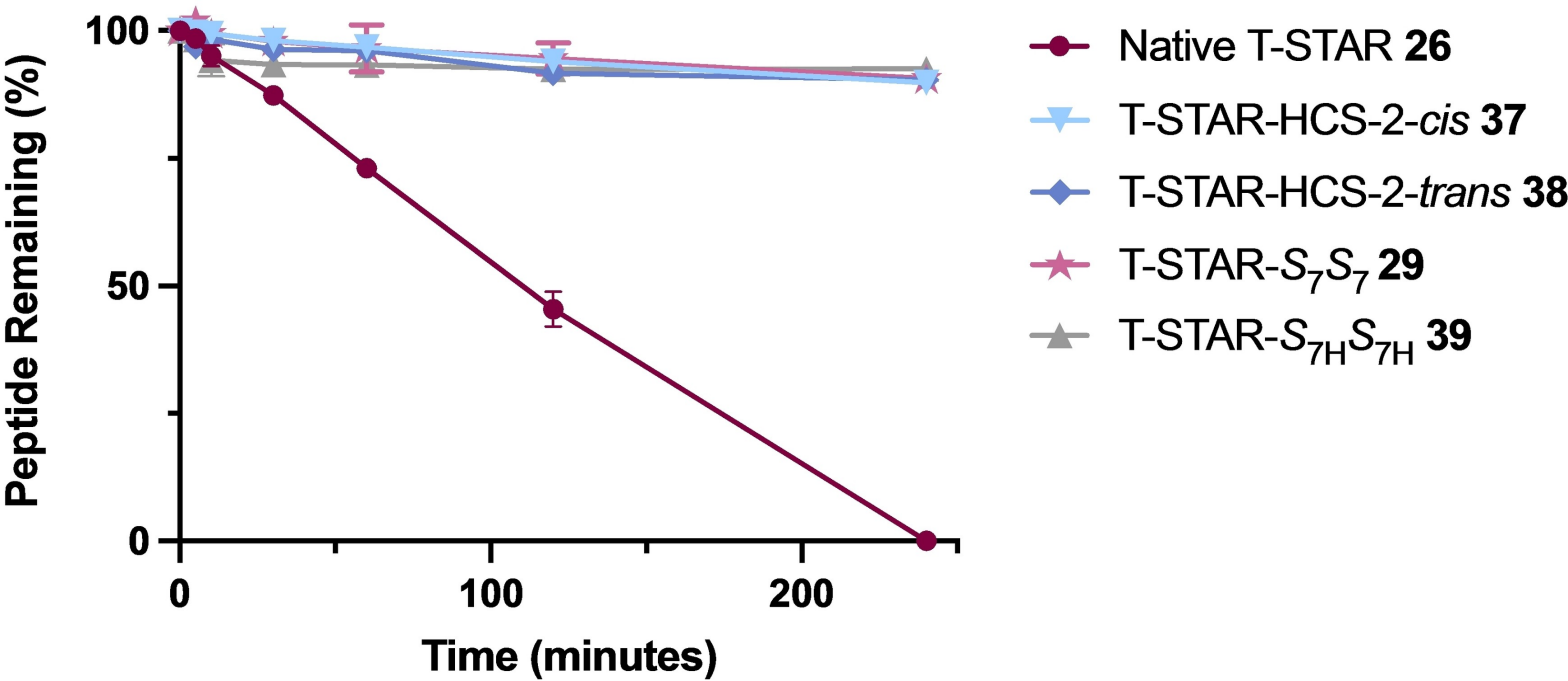
Stability comparison of native T‐STAR‐1 **26**, T‐STAR‐HCS‐2‐*cis* **37**, T‐STAR‐HCS‐2‐*trans* **38**, T‐STAR‐*S*_7_ *S*_7_ **29** and T‐STAR‐*S*_7H_ *S*_7H_ **39** in the presence of chymotrypsin (0.01 mg/mL) from *t*=0 to *t*=4 hours. Points are the mean of three independent experiments ±SEM. Calculated half‐life for native T‐STAR **26**=*t*_1/2_ 109 min.

After 4 h, the native T‐STAR peptide **26** (*t*_1/2_=109 min) was completely degraded by the enzyme as confirmed by analytical RP‐HPLC and LC–MS (Figure 4). Of the stapled analogues tested, both alkene hydrocarbon and diyne‐girder constrained peptides were extremely stable with >92 % of all peptides remaining after the last time point (4 h). This result is consistent with our CD data which showed very similar percentage helicity for T‐STAR‐HCS‐2‐*cis* **37**, T‐STAR‐HCS‐2‐*trans* **38**, T‐STAR‐*S*_7_ *S*_7_ **29** and T‐STAR‐*S*_7H_ *S*_7H_ **39**. The conformational constraints are thus effective at locking the peptide into a helical structure restricting enzymatic degradation by chymotrypsin.

### Cell Permeability Fluorescence Assay

**Figure 5:**
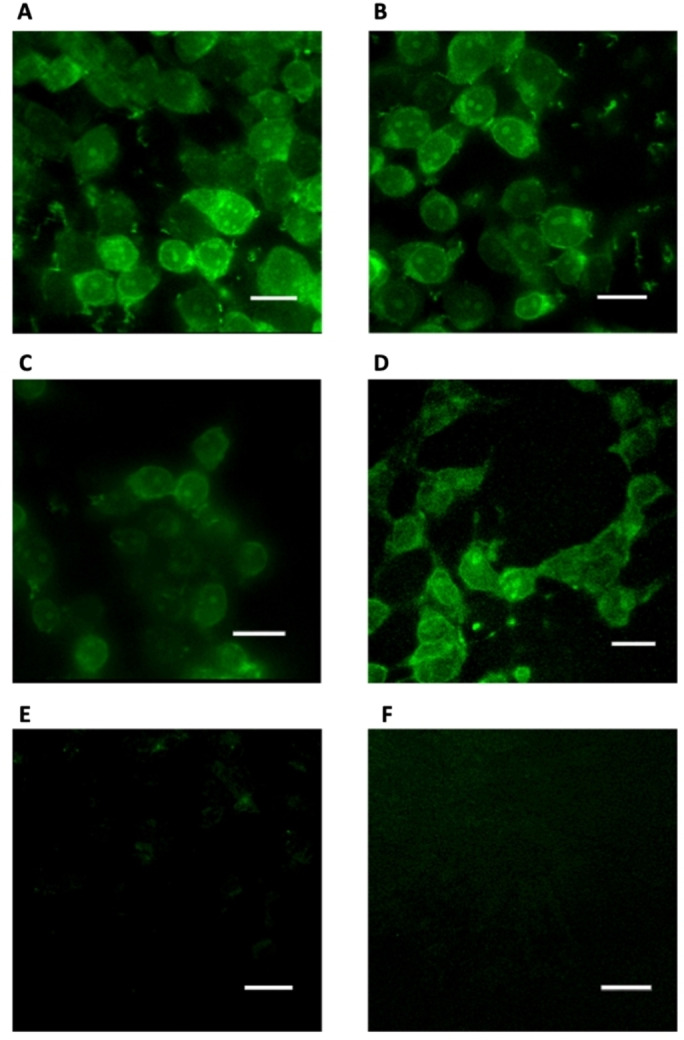
Fluorescence imaging showing A) the diyne‐girder T‐STAR S_7_S_7_ stapled peptide **29**, B and C) alkene stapled peptides **37** & **38** and D) diyne‐girder T‐STAR S_7H_S_7H_ stapled peptide **39** internalised in cells. E) The native T‐STAR peptide **26** showed no internalisation. F) DMSO negative control. Microscopy images were acquired with a custom‐built multi‐modal microscope setup. Fluorescein excitation was conducted at 495 nm. Scale bars=40 μm. Images were processed using MetaMorph software.

To investigate the cellular uptake of the diyne‐girder stapled T‐STAR‐*S*_7_ *S*_7_ **29**, compared to the unmethylated T‐STAR‐*S*_7H_ *S*_7H_ **39**, the alkene stapled peptides **37** & **38** and native T‐STAR **26**, fluorescence microscopy was used. HEK‐293 cells were treated with either 20 μM fluorescein stapled peptides **29, 39**, **37** & **38** or fluorescein native peptide **26** and imaged 3 hours post‐treatment (Figure 5). The stapled analogues **29**, **39**, **37** & **38** showed a diffused intracellular localization, confirming efficient cellular penetration (Figure 5A–D). As expected, the native peptide **26** showed no cellular internalisation (Figure 5E). Brightfield images were also acquired and overlaid with the fluorescence channel images for all experiments (see Supporting Information Figure S4).

### Raman spectroscopy

**Figure 6:**
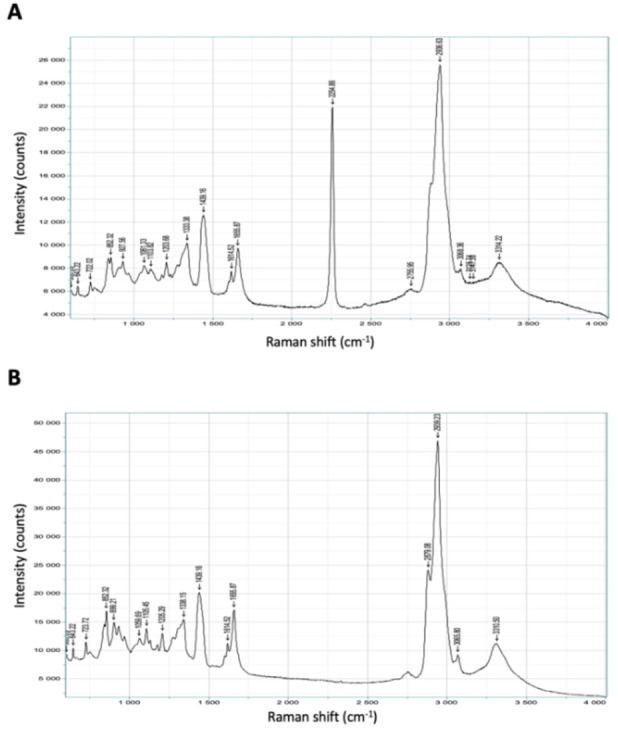
Solid‐state Raman spectra of A) diyne‐girder stapled T‐STAR S_7_S_7_ **41**. The diyne functionality gives a peak at ∼2,255 cm^−1^, in the cell‐silent region and B) the acetylated native T‐STAR **40** showing the absence of the diyne functionality peak.

Traditionally, cell‐uptake experiments are performed using fluorescently labelled analogues of peptides that have different physicochemical properties compared to the unlabelled peptide. A conformational constraint that also acts as a chromophore for imaging would provide a unique advantage. Alkyne groups are Raman chromophores and have been used extensively as molecular probes for cell imaging.^[29]^ We therefore hypothesised that the diyne‐girder constraint would act as a Raman chromophore and facilitate the visualisation of peptides in cells using Raman microscopy. To test this hypothesis, we prepared unlabelled analogues of native T‐STAR **40** and the optimal stapled analogue, T‐STAR‐*S*_7_ *S*_7_ **41** for analysis using Raman spectroscopy. Raman imaging of the solid diyne stapled T‐STAR peptide **41** was conducted using Raman spectroscopy with a laser at 532.02 nm. Figure 6(A) shows the spectra from the diyne stapled peptide **41**. A significant peak can be observed in the cell‐silent region at ∼ 2,255 cm^−1^. Raman was also performed on the native T‐STAR peptide **40** to confirm the absence of this peak at ∼ 2,255 cm^−1^ (Figure 6B).

These data demonstrate that the diyne‐girder stapled peptide **41** shows a significant peak in the cell‐silent region (1,800–2,800 cm^−1,^ Figure 6A), and thus has potential application in confocal cellular Raman imaging.

## Conclusions

Alkene hydrocarbon stapling is used frequently in chemical biology and drug discovery to conformationally constrain peptides to adopt their helical bioactive conformation and improve their physicochemical properties. However, there are several issues with *i*, *i*+7 stapled peptides that still need to be addressed. We report here the development of a new diyne‐girder stapling strategy with several advantages, including the requirement for only one type of unnatural amino acid and produces a single isomer on macrocyclisation. The optimal amino acid stereochemistry and diyne hydrocarbon bridge length was investigated through the asymmetric synthesis of nine Fmoc alkyne amino acids with varying lengths and stereochemistries. An *S*_7_ *S*_7_ hydrocarbon staple including α‐methyl groups was found to be the most helical. Proteolytic stability studies confirmed that the stapled analogues were more stable than their native counterpart. Fluorescence microscopy determined that the alkene and diyne stapled analogues were able to penetrate cells, whilst the native could not. Furthermore, we envisioned that the diyne‐girder constraint may show Raman activity in the cell silent region, which could be useful for cell imaging. We have determined that the diyne‐girder stapled peptide has a significant peak in the cell silent region (1,800–2,800 cm^−1^) using solid‐state Raman spectroscopy. Our current and future investigations will focus on the application of diyne‐girder stapled peptides, including the development of an *i*, *i*+11 diyne‐girder staple, for the regulation of protein‐protein interactions and their visualisation in cells using confocal Raman imagining.

## Experimental Section

**Solid‐phase peptide synthesis**: Peptides were synthesised following the general protocol (see ESI). Fmoc deprotections were carried out using 20 % piperidine in DMF (4.5 mL) spiked with 5 % formic acid at 75 °C for 30 s. A second deprotection (4.5 mL) was then undertaken at 75 °C for 3 min. Resin was washed with DMF at rt (4×4.5 mL). Coupling reactions were performed using Fmoc‐amino acid (4 equiv., 0.2 M in DMF), HCTU (4 equiv., 0.5 M in DMF) and DIPEA (8 equiv., 2 M in NMP). Peptide macrocyclisations were carried out on‐resin using the general protocols (see ESI).

**Circular dichroism (CD)**: CD spectra were obtained using a JASCO J‐810 CD spectrometer. A range of 190–260 nm was scanned at a speed of 50 nm/min, with a 1 nm data pitch, a 1 nm bandwidth, and an 8 s response time. Samples were prepared (50 μM) in phosphate buffered saline (PBS; pH 7.4), and CD spectra measured in a 1 mm or 0.2 mm quartz cuvette. Raw data (mdeg) were converted to mean residue ellipticity (MRE; deg cm^2^ dmol^−1^ res^−1^) by normalizing for path length, peptide concentration, and number of amide bonds. Percentage helicities can be calculated for *α*‐helical peptides. The raw CD data was converted to mean residue ellipticity (MRE) using Equation S1 (see Supporting Information) and the value at 222 nm was used to calculate the % helicity of the peptides using Equation S2 (see Supporting Information).

**Proteolytic stability assay**: Solutions of peptides (300 nM) were prepared in 50 μL DMSO+950 μL PBS buffer (pH 7.4). A standard solution of *m*‐cresol (0.05 mg/mL) and a trypsin/chymotrypsin solution (0.01 mg/mL) were prepared in PBS buffer (pH 7.4). To 100 μL of peptide solution was added 100 μL of trypsin solution and 100 μL of the *m*‐cresol standard solution and the solutions incubated at 37 °C. Aliquots were taken (40 μL) at *t*=0, 5 min, 10 min, 30 min, 1 h and 2 h. Samples were quenched with 15 μL MeCN and 25 μL of 2 % TFA/H_2_O and centrifuged at 13,800 × g for 5 min. The supernatant was analysed by analytical HPLC as previously described. The experiments were performed in triplicate with controls containing either peptide or enzyme alone in buffer. The percentage peptide remaining at each time point was calculated by peak integration relative to the percentage of the standard solution.

**Cell permeability fluorescence assay**: HEK293 cells were cultured in Dulbecco's Modified Eagle Medium (DMEM, high glucose with GlutaMAX, Gibco) supplemented with 10 % (v/v) foetal bovine serum (Gibco) and 1 % (v/v) penicillin/streptomycin (10,000 units/mL penicillin, 10,000 μg/mL streptomycin, Gibco). Cultured cells were maintained in a humidified incubator at 37 °C, 5 % CO_2_ and passaged twice weekly in T‐25 flasks (Corning). For cell counting, an aliquot (10 μL) of cell solution in media was added to a haemocytometer slide which was viewed using a microscope for manual inspection and counting. For experiments, 300,000 cells were seeded into 6‐well plates (CytoOne) on 30 mm cover glass slides pre‐treated with 0.1 mg/mL poly‐D‐lysine and left to grow for two days to reach ca. 80 % confluency before compound incubation. Media was removed and the cells were washed with PBS prior to treatment with compound in PBS (2 h, 20 μM, 37 °C). Cells were then washed again with PBS twice, fixed with a 4 % (w/v) solution of formaldehyde in PBS (10 min, 37 °C) and washed with PBS twice prior to analysis.

**Fluorescence imaging**: Images were acquired on a MetaMorph/Metafluor fluorescence imaging microscope system equipped with a 40× Superfluor objective for an exposure time of 1000 ms. Excitation for fluorescein was conducted at 495 nm. Image analysis and processing was performed using MetaMorph microscopy software.

**Raman spectroscopy experiments**: Raman spectroscopy was performed using the Horiba Jobin Yvon LabRAM HR system with a Ventus CD laser at 532.02 nm, 100 mW. The hole width was 200 μM with a diffraction grating of 600 g/mm using an Olympus x50LWD objective lens. The recorded spectral range was 600–4000 cm^−1^ and data acquisition was performed during 5 seconds with 3 repeats and collected with the Synapse OCD detector. 100 % power was used for peptide 40 and 50 % power was used for stapled peptide 41. Data was analysed using the Labspec 5 software.

## Supporting Information

Supporting Information is available from the Wiley Online Library or from the author. Additional references cited within the Supporting Information.[^30^, ^31^, ^32^, ^33^, ^34^, ^35^, ^36^, ^37^, ^38^, ^39^]

## Author Contributions

Danielle C. Morgan and Laura McDougall contributed equally to this work. Danielle C. Morgan, Laura McDougall and Astrid Knuhtsen synthesised the compounds. Laura McDougall performed the CD experiments and protease stability assays. Danielle C. Morgan performed the cellular and Raman experiments. Danielle C. Morgan and Andrew G. Jamieson wrote the manuscript. All authors read and approved the manuscript.

## Conflict of interest

A patent application [(GB) Patent Application No: 2219576.2] has been submitted on this work.

1

## Supporting information

As a service to our authors and readers, this journal provides supporting information supplied by the authors. Such materials are peer reviewed and may be re‐organized for online delivery, but are not copy‐edited or typeset. Technical support issues arising from supporting information (other than missing files) should be addressed to the authors.

Supporting Information

## Data Availability

The data that support the findings will be available in ChemRxiv at https://doi.org/10.26434/chemrxiv‐2023‐4t60s following an embargo from the date of publication to allow for commercialization of research findings.
