# Supplementary material for: Development of Bifunctional, Raman Active Diyne‐Girder Stapled α‐Helical Peptides
Source: Chemistry. 2023 Jun 13;29(41):e202300855. doi: 10.1002/chem.202300855 (PMC10946806; doi:10.1002/chem.202300855)
Supplement: Supplementary file 1 — Supporting Information [file CHEM-29-0-s001.pdf]

Chemistry–A European Journal

Supporting Information

Development of Bifunctional, Raman Active Diyne-Girder Stapled α-Helical Peptides

Danielle C. Morgan,* Laura McDougall, Astrid Knuhtsen, and Andrew G. Jamieson

Table of Contents

Experimental Procedures *7*

General Information **7**

Peptide synthesis - General methods **7**

Solid Phase Peptide Synthesis (SPPS) and Peptide Modifications **7**

Resin Swelling 8
Amino Acid Coupling 8
Fmoc Deprotection 8
Coupling of Fluorescein Isothiocyanate (FITC) 8
N-Terminal Acetylation (capping) 9
Ring Closing Metathesis (RCM) 9
Glaser Reaction 9
Peptide Test Cleavages 9
Peptide Full Cleavage and Global Deprotection 10
Peptide Purification 10

Circular Dichroism (CD) Spectroscopy **11**

Proteolytic Stability of Peptides **11**

Cell Permeability Fluorescence Assay **12**

Raman Spectroscopy of Native and Diyne Stapled T-STAR Peptide **12**

Amino Acid Synthesis - Experimental Methods *14*

S1 ((2-Fluorobenzyl)-L-proline hydrochloride) **14**

S2 ((2-Fluorobenzyl)-D-proline hydrochloride) **15**

S3 ((*S*)-*N*-(2-Benzoylphenyl)-1-(2-fluorobenzyl)pyrrolidine-2-carboxamide) **16**

S4 ((*R*)-*N*-(2-Benzoylphenyl)-1-(2-fluorobenzyl)pyrrolidine-2-carboxamide) **17**

S5 (7-Octyn-1-ol) **18**

1 ((*S*)-((2-[1-(2-Fluorobenzyl)pyrrolidine-2-carboxamide]phenyl)phenylmethylene)-(*S*)-alaninato-*N,N′,N′′*,O)nickel(II)) **19**

2 ((*R*)-((2-[1-(2-Fluorobenzyl)pyrrolidine-2-carboxamide]phenyl)phenylmethylene)-(*R*)-alaninato-*N,N′,N′′*,O)nickel(II)) **20**

3 ((*S*)-((2-[1-(2-Fluorobenzyl)pyrrolidine-2-carboxamide]phenyl)phenylmethylene)-(*S*)-glycinato-*N,N′,N′′*,O)nickel(II)) **21**

4 (5-Iodopent-1-yne) **22**

5 (6-Iodohex-1-yne) **23**

6 (7-Iodohept-1-yne) **24**

7 (8-Iodooct-1-yne) **25**

8 ((*S*)-((2-[1-(2-Fluorobenzyl)pyrrolidine-2-carboxamide]phenyl)phenylmethylene)-(*S*)-pentynylalaninato-*N,N′,N′′*,O)nickel(II)) **25**

9 ((*S*)-((2-[1-(2-Fluorobenzyl)pyrrolidine-2-carboxamide]phenyl)phenylmethylene)-(*S*)-hexynylalaninato-*N,N′,N′′*,O)nickel(II)) **27**

10 ((*S*)-((2-[1-(2-Fluorobenzyl)pyrrolidine-2-carboxamide]phenyl)phenylmethylene)-(*S*)-heptynylalaninato-*N*,*N'*,*N''*,O)nickel(II)) 28

11 ((*S*)-((2-[1-(2-Fluorobenzyl)pyrrolidine-2-carboxamide]phenyl)phenylmethylene)-(*S*)-octynylalaninato-*N*,*N'*,*N''*,O)nickel(II)) 29

12 ((*S*)-((2-[1-(2-Fluorobenzyl)pyrrolidine-2-carboxamide]phenyl)phenylmethylene)-(*S*)-heptynylglycinato-*N*,*N'*,*N''*,O)nickel(II)) 30

13 ((*R*)-((2-[1-(2-Fluorobenzyl)pyrrolidine-2-carboxamide]phenyl)phenylmethylene)-(*R*)-pentynylalaninato-*N*,*N'*,*N''*,O)nickel(II)) 32

14 ((*R*)-((2-[1-(2-Fluorobenzyl)pyrrolidine-2-carboxamide]phenyl)phenylmethylene)-(*R*)-hexynylalaninato-*N*,*N'*,*N''*,O)nickel(II)) 33

15 ((*R*)-((2-[1-(2-Fluorobenzyl)pyrrolidine-2-carboxamide]phenyl)phenylmethylene)-(*R*)-heptynylalaninato-*N*,*N'*,*N''*,O)nickel(II)) 34

16 ((*R*)-((2-[1-(2-Fluorobenzyl)pyrrolidine-2-carboxamide]phenyl)phenylmethylene)- (*R*)-octynylalaninato-*N*,*N'*,*N''*,O)nickel(II)) 35

17 ((*S*)-2-((((9*H*-fluoren-9-yl)methoxy)carbonyl)amino)-2-methylhept-6-ynoic acid) 37

18 ((*S*)-2-((((9H-fluoren-9-yl)methoxy)carbonyl)amino)-2-methyloct-7-ynoic acid) 38

19 ((*S*)-2-((((9*H*-Fluoren-9-yl)methoxy)carbonyl)amino)-2-methylnon-8-ynoic acid) 39

20 ((*S*)-2-((((9*H*-Fluoren-9-yl)methoxy)carbonyl)amino)-2-methyldec-9-ynoic acid) 40

21 ((*S*)-2-((((9*H*-Fluoren-9-yl)methoxy)carbonyl)amino)-non-8-ynoic acid) 41

22 ((*R*)-2-((((9*H*-fluoren-9-yl)methoxy)carbonyl)amino)-2-methylhept-6-ynoic acid) 42

23 ((*R*)-2-((((9H-fluoren-9-yl)methoxy)carbonyl)amino)-2-methyloct-7-ynoic acid) 43

24 ((*R*)-2-((((9*H*-Fluoren-9-yl)methoxy)carbonyl)amino)-2-methylnon-8-ynoic acid) 44

25 ((*R*)-2-((((9*H*-Fluoren-9-yl)methoxy)carbonyl)amino)-2-methyldec-9-ynoic acid) 45

NMR Data* *46

S1 ((2-Fluorobenzyl)-L-proline hydrochloride) 46

^{1}H NMR 46

^{13}C NMR 46

^{19}F NMR 47

S2 ((2-Fluorobenzyl)-D-proline hydrochloride) 47

^{1}H NMR 47

^{13}C NMR 48

^{19}F NMR 48

S3 ((*S*)-*N*-(2-Benzoylphenyl)-1-(2-fluorobenzyl)pyrrolidine-2-carboxamide) 49

^{1}H NMR 49

^{13}C NMR 49

^{19}F NMR 50

S4 ((*R*)-*N*-(2-Benzoylphenyl)-1-(2-fluorobenzyl)pyrrolidine-2-carboxamide) 51

^{1}H NMR 51

^{13}C NMR 51

^{19}F NMR 52

S5 (Oct-7-yn-1-ol) 52

^{1}H NMR 52

^{13}C NMR 53

1 ((*S*)-((2-[1-(2-Fluorobenzyl)pyrrolidine-2-carboxamide]phenyl)phenylmethylene)-(*S*)-alaninato-*N*,*N′*,*N″*,O)nickel(II)) 54
^{1}H NMR 54
^{13}C NMR 54
^{19}F NMR 55
2 ((*R*)-((2-[1-(2-Fluorobenzyl)pyrrolidine-2-carboxamide]phenyl)phenylmethylene)-(*R*)-alaninato-*N*,*N′*,*N″*,O)nickel(II)) 55
^{1}H NMR 55
^{13}C NMR 56
^{19}F NMR 56
3 ((*S*)-((2-[1-(2-Fluorobenzyl)pyrrolidine-2-carboxamide]phenyl)phenylmethylene)-glycinato-*N*,*N′*,*N″*, O)nickel(II)) 57
^{1}H NMR 57
^{13}C NMR 57
^{19}F NMR 58
4 (5-Iodopent-1-yne) 58
^{1}H NMR 58
^{13}C NMR 59
5 (6-Iodohex-1-yne) 59
^{1}H NMR 59
^{13}C NMR 60
6 (7-Iodohept-1-yne) 60
^{1}H NMR 60
^{13}C NMR 61
7 (8-Iodooct-1-yne) 61
^{1}H NMR 61
^{13}C NMR 62
8 (C5 *S* Alkylated Complex - (*S*)-((2-[1-(2-Fluorobenzyl)pyrrolidine-2-carboxamide]phenyl)phenylmethylene)-(*S*)-pentynylalaninato-*N*,*N′*,*N″*,O)nickel(II)) 63
^{1}H NMR 63
^{13}C NMR 63
^{19}F NMR 64
9 (C6 *S* Alkylated Complex - (*S*)-((2-[1-(2-Fluorobenzyl)pyrrolidine-2-carboxamide]phenyl)phenylmethylene)-(*S*)-hexynylalaninato-*N*,*N′*,*N″*,O)nickel(II)) 64
^{1}H NMR 64
^{13}C NMR 65
^{19}F NMR 65
10 (C7 *S* Alkylated Complex - (*S*)-((2-[1-(2-Fluorobenzyl)pyrrolidine-2-carboxamide]phenyl)phenylmethylene)-(*S*)-heptynylalaninato-*N*,*N′*,*N″*,O)nickel(II)) 66
^{1}H NMR 66
^{13}C NMR 66
^{19}F NMR 67
11 (C8 *S* Alkylated Complex - (*S*)-((2-[1-(2-Fluorobenzyl)pyrrolidine-2-carboxamide]phenyl)phenylmethylene)-(*S*)-octynylalaninato-*N*,*N′*,*N″*,O)nickel(II)) 67
^{1}H NMR 67
^{13}C NMR 68
^{19}F NMR 68
12 (S7 Gly Alkylated Complex - (*S*)-((2-[1-(2-Fluorobenzyl)pyrrolidine-2-carboxamide]phenyl)phenylmethylene)-(*S*)-heptynylglycinato-*N*,*N′*,*N″*,O)nickel(II)) 69

^{1}H NMR 69
^{13}C NMR 69
^{19}F NMR 70

13 (C5 *R* Alkylated Complex - (*R*)-((2-[1-(2-Fluorobenzyl)pyrrolidine-2-carboxamide]phenyl)phenylmethylene)-(*R*)-pentynylalaninato-*N*,*N'*,*N''*,O)nickel(II)) 70
^{1}H NMR 70
^{13}C NMR 71
^{19}F NMR 71

14 (C6 *R* Alkylated Complex - (*R*)-((2-[1-(2-Fluorobenzyl)pyrrolidine-2-carboxamide]phenyl)phenylmethylene)-(*R*)-hexynylalaninato-*N*,*N'*,*N''*,O)nickel(II)) 72
^{1}H NMR 72
^{13}C NMR 72
^{19}F NMR 73

15 (C7 *R* Alkylated Complex - (*R*)-((2-[1-(2-Fluorobenzyl)pyrrolidine-2-carboxamide]phenyl)phenylmethylene)-(*R*)-heptynylalaninato-*N*,*N'*,*N''*,O)nickel(II)) 73
^{1}H NMR 73
^{13}C NMR 74
^{19}F NMR 74

16 (C8 *R* Alkylated Complex - (*R*)-((2-[1-(2-Fluorobenzyl)pyrrolidine-2-carboxamide]phenyl)phenylmethylene)- (*R*)-octynylalaninato-*N*,*N'*,*N''*,O)nickel(II)) 75
^{1}H NMR 75
^{13}C NMR 75
^{19}F NMR 76

17 (C5 *S* AA - (*S*)-2-((((9*H*-fluoren-9-yl)methoxy)carbonyl)amino)-2-methylhept-6-ynoic acid) .. 76
^{1}H NMR 76
^{13}C NMR 77

18 (C6 *S* AA - (*S*)-2-((((9H-fluoren-9-yl)methoxy)carbonyl)amino)-2-methyloct-7-ynoic acid) 77
^{1}H NMR 77
^{13}C NMR 78

19 (C7 *S* AA - (*S*)-2-((((9*H*-Fluoren-9-yl)methoxy)carbonyl)amino)-2-methylnon-8-ynoic acid)... 78
^{1}H NMR 78
^{13}C NMR 79

20 (C8 *S* AA - (*S*)-2-((((9*H*-Fluoren-9-yl)methoxy)carbonyl)amino)-2-methyldec-9-ynoic acid) ... 79
^{1}H NMR 79
^{13}C NMR 80

21 (C7 *S* H AA - (*S*)-2-((((9*H*-Fluoren-9-yl)methoxy)carbonyl)amino)-non-8-ynoic acid)........ 80
^{1}H NMR 80
^{13}C NMR 81

22 (C5 *R* AA - (*R*)-2-((((9*H*-fluoren-9-yl)methoxy)carbonyl)amino)-2-methylhept-6-ynoic acid) . 81
^{1}H NMR 81
^{13}C NMR 82

23 (C6 *R* AA - ((*R*)-2-((((9H-fluoren-9-yl)methoxy)carbonyl)amino)-2-methyloct-7-ynoic acid)... 82
^{1}H NMR 82
^{13}C NMR 83

24 (C7 *R* AA - (*R*)-2-((((9*H*-Fluoren-9-yl)methoxy)carbonyl)amino)-2-methylnon-8-ynoic acid) .. 83
^{1}H NMR 83

25 (C8 *R* AA - (*R*)-2-((((9*H*-Fluoren-9-yl)methoxy)carbonyl)amino)-2-methyldec-9-ynoic acid)... 84
^{1}H NMR 84
^{13}C NMR 85

Peptide Characterisation Data *86*
Peptide Characterisation Table **86**
26 (Native T-STAR) **90**
20 min HPLC 90
50 min HPLC 90
HR-MS 91
27 (T-STAR-diyne-S_5S_5) **91**
20 min HPLC 91
50 min HPLC 92
HR-MS 92
28 (T-STAR-diyne-S_6S_6) **93**
20 min HPLC 93
50 min HPLC 93
HR-MS 94
29 (T-STAR-diyne-S_7S_7) **94**
20 min HPLC 94
50 min HPLC 95
HR-MS 95
30 (T-STAR-diyne-R_7R_7) **96**
20 min HPLC 96
50 min HPLC 96
HR-MS 97
31 (T-STAR-diyne-R_7S_7) **97**
20 min HPLC 97
50 min HPLC 98
HR-MS 98
32 (T-STAR-diyne-S_7R_7) **99**
20 min HPLC 99
50 min HPLC 99
HR-MS 100
33 (T-STAR-diyne-S_8S_8) **100**
20 min HPLC 100
50 min HPLC 101
HR-MS 101
34 (T-STAR-diyne-R_8R_8) **102**
20 min HPLC 102
50 min HPLC 102
HR-MS 103
35 (T-STAR-diyne-R_8S_8) **103**
20 min HPLC 103
50 min HPLC 104
HR-MS 104
36 (T-STAR-diyne-S_8R_8) **105**
20 min HPLC 105
50 min HPLC 105
HR-MS 106
37 (T-STAR-HCS-2-*cis*) **106**
20 min HPLC 106
50 min HPLC 107
HR-MS 107
38 (T-STAR-HCS-2-*trans*) **108**
20 min HPLC 108
50 min HPLC 108
HR-MS 109
39 (T-STAR-diyne-$S_{7H}S_{7H}$) **109**
20 min HPLC 109

50 min HPLC 110
HR-MS 110
40 (Ac-Native T-STAR) 111
20 min HPLC 111
50 min HPLC 111
HR-MS 112
41 (Ac-T-STAR-diyne-S_7S_7) 112
20 min HPLC 112
50 min HPLC 113
HR-MS 113
Supporting Information Tables and Figures 114
Circular Dichroism Data for all T-STAR Peptide Analogues 114
Fluorescence Microscopy Images for all T-STAR Peptide Analogues 116

Experimental Procedures

General Information

All reagents were purchased from commercial sources and used without further purification. Dry solvents were purified using a PureSolv 500 MD solvent purification system. Thin-layer chromatography was performed on aluminium backed plates (0.25 µm) with silica gel 60 coated F_{254} and visualised by staining with $KMnO_4$ or ninhydrin. Proton (^{1}H), carbon (^{13}C) and fluorine (^{19}F) NMR spectra were obtained using a Bruker Avance 400 MHz spectrometer and chemical shifts δ given in parts per million (ppm) relative to TMS (δ = 0 ppm). Proton and carbon chemical shifts were assigned using proton, carbon, Correlation Spectroscopy (COSY) and Heteronuclear Single Quantum Coherence (HSQC) experiments. LC-MS was performed on a Thermo Scientific Dionex Ultimate 3000 LC system coupled to a Thermo Scientific LCQ FleetTM Ion trap mass spectrometer using a Dr. Maisch Reprosil Gold 120 C_{18} column (110 Å, 3 µm, 150 × 4 mm) with a flow rate of 1 mL/min. A linear gradient of buffer A (95/5 H_2O/MeCN and 0.1% TFA) to buffer B (95/5 MeCN/H_2O and 0.1% TFA) was used over 10 or 20 mins. Normal phase column chromatography was performed on a Biotage Isolera One purification system using prepacked silica Biotage SNAP KP-SIL cartridges. High-resolution ESI mass spectrometry was performed on a Bruker micrOTOF-Q II in positive mode. HRMS data are reported as mass to charge ratio (m/z) = observed/MW. Optical rotation was determined using an Autopol V polarimeter.

Peptide synthesis - General methods

Solid Phase Peptide Synthesis (SPPS) and Peptide Modifications

Peptides were synthesised using an Automated Biotage Initiator+ Alstra microwave synthesiser on 0.1 mmol scale. The Sam68 peptides were synthesised using Rink Amide ChemMatrix resin (0.49 mmol/g). L-configured amino acids were used for peptide synthesis with *N*-Fmoc protecting groups. Analytical RP-HPLC was undertaken on a Shimadzu

instrument equipped with a dual wavelength UV detector and a Phenomenex Aeris 5 µm C18 (150 × 4.6 mm) column at a flow rate of 1 mL/min. A linear gradient of buffer A (95/5 H_2O/MeCN and 0.1% TFA) to buffer B (95/5 MeCN/H_2O and 0.1% TFA) was used over 20 mins or 50 mins. UV measurements were recorded at 214 nm and 280 nm. Peptides were lyophilised using a Christ Alpha 2-4 LDplus freezedryer.

Resin Swelling

Resin was swollen in DMF at 70 °C for 20 min.

Amino Acid Coupling

Coupling reactions were performed using Fmoc-amino acid (4 equiv., 0.2 M in DMF), HCTU (4 equiv., 0.5 M in DMF) and DIPEA (8 equiv., 2 M in NMP). Proteinogenic Fmoc protected amino acids were coupled at 75 °C for 10 mins. Fmoc-Arg(Pbf)-OH was double coupled at rt for 60 mins followed by 5 mins at 75 °C. Non-proteinogenic amino acids (2 equiv.) were coupled using HCTU (2 equiv.) and DIPEA (4 equiv.) for 20 mins. Amino acids following unnatural amino acids were coupled twice. Resin was washed with DMF at rt (4 × 4.5 mL).

Fmoc Deprotection

Fmoc deprotections were carried out using 20% piperidine in DMF (4.5 mL) spiked with 5% formic acid at 75 °C for 30 s. A second deprotection (4.5 mL) was then undertaken at 75 °C for 3 mins. Resin was washed with DMF at rt (4 × 4.5 mL).

Coupling of Fluorescein Isothiocyanate (FITC)

Before addition to the resin, FITC (2 equiv.) and DIPEA (4 equiv.) in DMF were pre-activated for 2 mins. The solution was added to the resin and left to shake shielded from light. After draining, the resin was washed with DMF (3 × 5 mL), MeOH (3 × 5 mL) and DMF (3 × 5 mL).

***N*-Terminal Acetylation (capping)**

Peptides requiring *N*-terminal acetylation were treated on-resin with acetic anhydride (3 equiv.), DIPEA (4.5 equiv.) and DMF (7 mL for 0.1 mmol of resin) for 20 min with agitation. The resin was then washed with DMF (3 x 5 mL) and DCM (3 x 5 mL) prior to peptide cleave and global deprotection.

Ring Closing Metathesis (RCM)

Peptides containing (*R*)-*N*-Fmoc-*α*-(7-Octenyl)alanine and (*S*)-*N*-Fmoc-*α*-(4-pentenyl)alanine were stapled by on-resin ring-closing metathesis (RCM). The resin was suspended in dry DCE before adding Grubbs 1st Catalyst (20 mol%) in DCE (4 mL for 0.1 mmol of resin), leaving for 2 h with agitation and excluding light. The resin was washed with DCE (3 mL) before repeating the RCM. The resin was then washed with DCM (2 x 5 mL) prior to peptide cleave and global deprotection.

Glaser Reaction

Peptides containing (S)-2-((((9H-Fluoren-9-yl)methoxy)carbonyl)amino)-2-methylnon-8-ynoic acid or (S)-2-((((9H-Fluoren-9-yl)methoxy)carbonyl)amino)-O-(pent-4-yn-yl)-L-serine were cyclised on resin. Peptide on resin (1 equiv., 0.1 mmol) was added to a microwave vial followed by a solution of CuCl (5 equiv.) and 4,4'-bis(hydroxymethyl)-2,2'-bipyridine (7.5 equiv.) in DMF (10 mL) and DIPEA (20 equiv.). The reaction was heated to 60 °C for 3 h in a microwave reactor and excess reagents filtered off. The resin was washed with 0.5% sodium diethyldithiocarbamate in 0.5% DIPEA/DMF (5 × 10 mL), DMF (3 × 10 mL), methanol (MeOH) (3 × 10 mL), DMF (3 × 10 mL) and DCM (3 x 10 mL).

Peptide Test Cleavages

Test cleavages were performed on ~5 mg of resin using a cocktail (1 mL) of TFA (95%), TIPS (2.5%) and H_2O (2.5%) for 1 h with agitation. The resin was subsequently filtered and the TFA

evaporated using a stream of N_2, the peptide was precipitated with cold Et_2O and centrifuged (4,500 rpm for 5 min). Peptides were dissolved in a mixture of H_2O and ACN with 0.1% TFA and lyophilized on a Christ Alpha 2-4 LO plus freeze dryer.

Peptide Full Cleavage and Global Deprotection

Peptides were cleaved from the resin using a cocktail (10 mL) of TFA (95%), TIPS (2.5%) and H_2O (2.5%) for 4 h with agitation. The resin was subsequently filtered and the TFA evaporated using a stream of N_2, the peptide was precipitated with cold Et_2O and centrifuged (4,500 rpm for 5 min). Peptides were dissolved in a mixture of H_2O and ACN with 0.1% TFA and lyophilized on a Christ Alpha 2-4 LO plus freeze dryer.

Peptide Purification

Crude peptides were purified by RP-HPLC using either an Agilent Technologies 1260 Infinity RP-HPLC system or a Dionex RP-HPLC system with Dionex P680 pumps and a Dionex UVD170U UV-vis detector (monitoring at 214 nm and 280 nm), each with a Phenomenex Gemini column (5 mm C18, 250 x 21.2 mm). Purified peptides were analysed on a Shimadzu RP-HPLC system with Shimadzu LC-20AT pumps, a Shimadzu SIL20A autosampler and a Shimadzu SPD-20A UV-vis detector using a Phenomenex Aeris column (5 mm C18, 100 Å, 150 x 10 mm). Peptides were eluted with linear gradients at column-dependent flow rates (1 mL/min for the Aeris, 10 mL/min for the Gemini), where buffer A = 0.1% TFA in H_2O and buffer B = 0.1% TFA in ACN. LC-MS was performed on a Thermo Scientific LCQ Fleet Ion Trap Mass Spectrometer using positive mode electrospray ionisation (ESI^+). Where buffer A = 0.1% TFA in H_2O and buffer B = 0.1% TFA in ACN, a linear gradient of 0-100 % B over 20 min with a flow rate of 1 mL/min was used with a Reprosil-Gold column (3 mm C18, 150 x 4 mm).

Circular Dichroism (CD) Spectroscopy

CD spectra were obtained at room temperature using a JASCO J-810 CD spectrometer. A range of 190-260 nm was scanned at a speed of 50 nm/min, with a 1 nm data pitch, a 1 nm bandwidth, and an 8 s response time. Samples were prepared (50 µM) in phosphate buffered saline (PBS; pH 7.4), and CD spectra measured in a 1 mm or 0.2 mm quartz cuvette. Raw data (mdeg) were converted to mean residue ellipticity (MRE; deg cm^2 $dmol^{-1}$ res^{-1}) by normalizing for path length, peptide concentration, and number of amide bonds. Percentage helicities can be calculated for α-helical peptides. The raw CD data was converted to mean residue ellipticity (MRE) using Equation S1. and the value at 222 nm was used to calculate the % helicity of the peptides using Equation S2..[30-32]

Equation S1. MRE calculation. Where θ = machine units in degrees, MRW (mean residue weight) = molecular mass of peptide/number of residues, l = path length (cm) and c = peptide concentration in mg/mL.

$$MRE = \theta\left(\frac{0.1 \times MRW}{l \times c}\right)$$

Equation S2. (**1**) % Helicity equation. (**2**) θ_c = random coil ellipticity calculation proposed by Luo and Baldwin.[32] (**3**) $\theta_{222\infty}$ = α-helix ellipticity calculation determined by Luo and Baldwin,[32] reading observed at 222 nm. T is the temperature in degrees Celsius, Np is the number of amide bonds and *k* = the peptide length correction factor, 3.[30]

$$(\mathbf{1})\ \%\,Helicity\ = \left(\frac{\theta_{222} - \theta_c}{\theta_{222\infty} - \theta_c}\right) \times\ 100$$

$$(\mathbf{2})\ \theta_c = 2220 - 53T$$

$$(\mathbf{3})\ \theta_{222\infty} = (-44000 + (250\ \times T)) \times \left(1 - \frac{k}{Np}\right)$$

Proteolytic Stability of Peptides

Solutions of peptides (300 nM) were prepared in 50 µL DMSO + 950 µL PBS buffer (pH 7.4). A standard solution of *m*-cresol (0.05 mg/mL) and a trypsin/chymotrypsin solution (0.01 mg/mL) were prepared in PBS buffer (pH 7.4). To 100 µL of peptide solution was added 100

µL of trypsin solution and 100 µL of the *m*-cresol standard solution and the solutions incubated at 37 °C. Aliquots were taken (40 µL) at t = 0, 5 mins, 10 mins, 30 mins, 1 h and 2 h. Samples were quenched with 15 µL MeCN and 25 µL of 2% TFA/H_2O and centrifuged at 13,800 × g for 5 mins. The supernatant was analysed by analytical HPLC as previously described. The experiments were performed in triplicate with controls containing either peptide or enzyme alone in buffer. The percentage peptide remaining at each time point was calculated by peak integration relative to the percentage of the standard solution.

Cell Permeability Fluorescence Assay

HEK293 cells were cultured in Dulbecco's Modified Eagle Medium (DMEM, high glucose with GlutaMAX, Gibco) supplemented with 10% (v/v) foetal bovine serum (Gibco) and 1% (v/v) penicillin/streptomycin (10,000 units/mL penicillin, 10,000 µg/mL streptomycin, Gibco). Cultured cells were maintained in a humidified incubator at 37 °C, 5% CO_2 and passaged twice weekly in T-25 flasks (Corning). For cell counting, an aliquot (10 µL) of cell solution in media was added to a haemocytometer slide which was viewed using a microscope for manual inspection and counting. For experiments, 300,000 cells were seeded into 6-well plates (CytoOne) on 30 mm cover glass slides pre-treated with 0.1 mg/mL poly-D-lysine and left to grow for two days to reach ca. 80% confluency before compound incubation. Media was removed and the cells were washed with PBS prior to treatment with compound in PBS (2 h, 20 µM, 37 °C). Cells were then washed again with PBS twice, fixed with a 4% (w/v) solution of formaldehyde in PBS (10 min, 37 °C) and washed with PBS twice prior to analysis.

Fluorescence Imaging. Images were acquired on a MetaMorph/Metafluor fluorescence imaging microscope system equipped with a 40× Superfluor objective for an exposure time of 1000 ms. Excitation for fluorescein was conducted at 495 nm. Image analysis and processing was performed using MetaMorph microscopy software.

Raman Spectroscopy of Native and Diyne Stapled T-STAR Peptide

Raman spectroscopy was performed using the Horiba Jobin Yvon LabRAM HR system with a Ventus CD laser at 532.02 nm, 100 mW. The hole width was 200 µM with a diffraction grating

of 600 g/mm using an Olympus x50LWD objective lens. The recorded spectral range was 600-4000 cm^{-1} and data acquisition was performed during 5 seconds with 3 repeats and collected with the Synapse OCD detector. 100% power was used for peptide **40** and 50% power was used for stapled peptide **41**. Data was analysed using the Labspec 5 software.

Amino Acid Synthesis - Experimental Methods

S1 ((2-Fluorobenzyl)-L-proline hydrochloride)

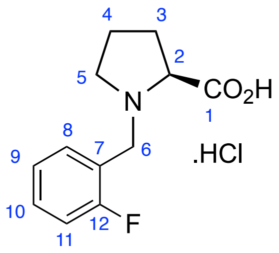

Potassium hydroxide (7.31 g, 0.130 mol, 3 equiv.) was dissolved in isopropanol at 40 °C before addition of L-proline (5.00 g, 43.4 mmol, 1 equiv.). 2-Fluorobenzyl bromide (5.24 mL, 43.4 mmol, 1 equiv.) was added dropwise *via* an addition funnel. The reaction was left to stir at 50 °C for 24 h. $HCl_{(aq.)}$ (37%) was added slowly until the pH of the mixture reached 5.5, as determined using a pH probe. After cooling in an ice bath, the suspension was filtered and concentrated *in vacuo*. The resulting oil was dissolved in DCM and left at 4 °C overnight. A white precipitate was removed by filtration and the filtrate was concentrated *in vacuo* to give **S1** as a viscous brown oil (7.77 g, 79%).

^{1}H NMR (500 MHz, $CDCl_3$) δ 7.40 (td, *J* = 7.5, 1.5 Hz, 1H, 9-H), 7.37 – 7.34 (m, 1H, 8-H), 7.16 (td, *J* = 7.5, 1.2 Hz, 1H, 10-H), 7.13 – 7.09 (m, 1H, 11-H), 4.19 (d, *J* = 12.9 Hz, 1H, 6-H), 4.02 (d, *J* = 12.9 Hz, 1H, 6´-H), 3.62 (dd, *J* = 9.9, 4.5 Hz, 1H, 2-H), 3.38 (ddd, *J* = 9.9, 6.7, 3.0 Hz, 1H, 5-H), 2.76 – 2.71 (m, 1H, 5´-H), 2.31 – 2.17 (m, 2H, 3-H), 1.95 – 1.83 (m, 2H, 4-H).

^{13}C NMR (101 MHz, $CDCl_3$) δ 171.9 (C=O), 161.4 (d, *J* = 248.1 Hz, Ar-C), 133.0 (d, *J* = 3.2 Hz, Ar-CH), 131.6 (d, *J* = 8.3 Hz, Ar-CH), 125.0 (d, *J* = 3.6 Hz, Ar-CH), 119.2 (d, *J* = 14.3 Hz, Ar-C), 116.0 (d, *J* = 21.8 Hz, Ar-CH), 67.5 (CH), 53.7 (CH_2), 51.0 (CH_2), 29.1 (CH_2), 23.2 (CH_2).

^{19}F NMR (376 MHz, $CDCl_3$) δ –116.9.

$[\alpha]_D^{20}$ –26.0 (c 0.1 in MeOH) lit: –24.1.[33]

IR (υ_{max}/cm^{-1}, neat) 3407 (OH), 2978 (C-H), 1617 (C=O).

HRMS-EI (calcd for $C_{12}H_{14}FNO_2$ $[M + Na]^+$) 246.0901, found 246.0892 (Δ = - 3.5 ppm).

The spectroscopic data is in good agreement with the literature.[33]

S2 ((2-Fluorobenzyl)-D-proline hydrochloride)

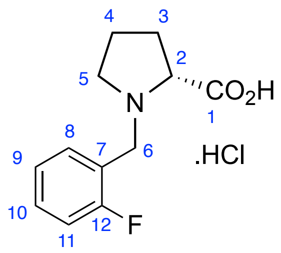

Method as **S1** using D-proline yielding **S2** as a viscous brown oil (2.16 g, 95%).

^{1}H NMR (500 MHz, $CDCl_3$) δ 7.40 (td, *J* = 7.5, 1.8 Hz, 1H, 9-H), 7.37 – 7.34 (m, 1H, 8-H), 7.16 (td, *J* = 7.5, 1.2 Hz, 1H, 10-H), 7.13 – 7.09 (m, 1H, 11-H), 4.19 (d, *J* = 12.9 Hz, 1H, 6-H), 4.02 (d, *J* = 12.9 Hz, 1H, 6´-H), 3.62 (dd, *J* = 9.9, 4.6 Hz, 1H, 2-H), 3.38 (ddd, *J* = 9.9, 6.7, 2.9 Hz, 1H, 5-H), 2.76 – 2.71 (m, 1H, 5´-H), 2.31 – 2.17 (m, 2H, 3-H), 1.95 – 1.83 (m, 2H, 4-H).

^{13}C NMR (101 MHz, $CDCl_3$) δ 172.0 (C=O), 161.5 (d, *J* = 248.0 Hz, Ar-C), 132.9 (d, *J* = 3.2 Hz, Ar-CH), 131.4 (d, *J* = 8.4 Hz, Ar-CH), 124.9 (d, *J* = 3.7 Hz, Ar-CH), 119.5 (d, *J* = 14.3 Hz, Ar-C), 115.9 (d, *J* = 21.8 Hz, Ar-CH), 67.3 (CH), 53.7 (CH_2), 51.0 (CH_2), 29.2 (CH_2), 23.2 (CH_2).

^{19}F NMR (376 MHz, $CDCl_3$) δ –116.9.

$[\alpha]_D^{20}$ = +25.0 (c 0.1 in MeOH).

IR (υ_{max}/cm^{-1}, neat) 3410 (OH), 2977 (C-H) 1620 (C=O).

HRMS-EI (calcd for $C_{12}H_{14}FNO_2$ [M + Na]$^+$) 246.0901, found 246.0892 (Δ = –3.7 ppm).

S3 ((*S*)-*N*-(2-Benzoylphenyl)-1-(2-fluorobenzyl)pyrrolidine-2-carboxamide)

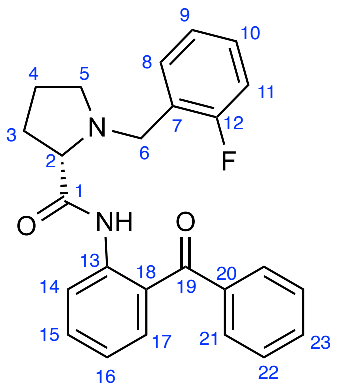

(*S*)-1-(2-Fluorobenzyl)pyrrolidine-2-carboxylic acid **S1** (0.100 g, 0.390 mmol, 1 equiv.) and *N*-methylimidazole (68.5 µL, 0.860 mmol, 2.2 equiv.) were dissolved in dry DCM (2 mL) before the addition of MsCl (36.2 µL, 0.470 mmol, 1.2 equiv.) at 0 °C. After 15 mins 2-aminobenzophenone (69.0 mg, 0.350 mmol, 0.9 equiv.) was added, the reaction mixture heated to 50 °C and left to stir for 18 h. The reaction was cooled and then quenched with aqueous sodium hydrogen carbonate solution (10 mL). The aqueous layer was extracted with DCM (3 × 10 mL) and subsequently washed with brine (3 × 10 mL). The organic layers were dried with $MgSO_4$, filtered and concentrated *in vacuo*. The crude mixture was purified by normal phase flash column chromatography using petroleum ether and EtOAc in a 0-25% gradient to give **S3** as a yellow solid (85.0 mg, 60%).

^{1}H NMR (500 MHz, $CDCl_3$) δ 11.40 (s, 1H, NH), 8.54 (dd, *J* = 8.4, 1.0 Hz, 1H, Ar-H), 7.76 – 7.74 (m, 2H, 2 × Ar-H), 7.62 – 7.58 (m, 1H, Ar-H), 7.55 – 7.44 (m, 5H, 9-H + 4 × Ar-H), 7.14 – 7.07 (m, 2H, 8-H + Ar-H), 6.92 (td, *J* = 7.5, 1.2 Hz, 1H, 10-H), 6.81 – 6.76 (m, 1H, 11-H), 3.89 (d, *J* = 13.2 Hz, 1H, 6-H), 3.73 (d, *J* = 13.2 Hz, 1H, 6´-H), 3.35 (dd, *J* = 10.2, 4.6 Hz, 1H, 2-H), 3.25 – 3.21 (m, 1H, 5-H), 2.49 – 2.43 (m, 1H, 5´-H), 2.29 – 2.19 (m, 1H, 3-H), 1.98 – 1.91 (m, 1H, 3´-H), 1.85 – 1.73 (m, 2H, 4-H).

^{13}C NMR (101 MHz, $CDCl_3$) δ 198.0 (C=O), 174.6 (C=O), 161.0 (d, *J* = 246.2 Hz, Ar-C), 139.2 (Ar-C), 138.8 (Ar-C), 133.4 (Ar-CH), 132.6 (Ar-CH), 131.8 (d, *J* = 4.3 Hz, Ar-CH), 130.2 (2 × Ar-CH), 128.9 (d, *J* = 8.2 Hz, Ar-CH), 128.4 (Ar-CH), 125.8 (Ar-C), 124.9 (d, *J* = 14.6 Hz, Ar-C), 124.0 (d,

J = 3.6 Hz, Ar-CH), 122.4 (Ar-CH), 121.6 (Ar-CH), 115.1 (d, J = 22.1 Hz, Ar-CH), 68.1 (CH), 53.9 (CH_2), 52.2 (CH_2), 31.2 (CH_2), 24.4 (CH_2).

^{19}F NMR (376 MHz, $CDCl_3$) δ –117.6.

Mp 85–87 °C.

$[\alpha]_D^{25}$ = –129.0 (c 0.1 in MeOH), lit: –125.1.[33]

IR (υ_{max}/cm^{-1}, neat) 3277 (N-H), 1682 (C=O), 1647 (C=O), 1578 (C=C).

HRMS-EI (calcd for $C_{25}H_{23}FN_2O_2$ [M + Na]$^+$) 425.1636, found 425.1623 (Δ = –2.9 ppm).

The spectroscopic data is in good agreement with the literature.[33]

S4 ((*R*)-*N*-(2-Benzoylphenyl)-1-(2-fluorobenzyl)pyrrolidine-2-carboxamide)

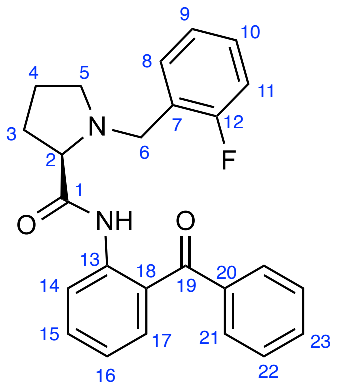

Method as **S3** using (*R*)-1-(2-Fluorobenzyl)pyrrolidine-2-carboxylic acid **S2**, yielding **S4** as a yellow solid (1.20 g, 63%).

^{1}H NMR (500 MHz, $CDCl_3$) δ 11.40 (s, 1H, NH), 8.54 (dd, J = 8.4, 1.1 Hz, 1H, Ar-H), 7.76 – 7.74 (m, 2H, Ar-H), 7.62 – 7.57 (m, 1H, Ar-H), 7.55 – 7.44 (m, 5H, 9-H + 4 × Ar-H), 7.14 – 7.07 (m, 2H, 8-H + Ar-H), 6.92 (td, J = 7.5, 1.2 Hz, 1H, 10-H), 6.81 – 6.76 (m, 1H, 11-H), 3.89 (d, J = 13.2 Hz, 1H, 6-H), 3.73 (d, J = 13.2 Hz, 1H, 6´-H), 3.35 (dd, J = 10.2, 4.6 Hz, 1H, 2-H), 3.25 – 3.20 (m, 1H, 5-H), 2.49 – 2.43 (m, 1H, 5´-H), 2.29 – 2.19 (m, 1H, 3-H), 1.98 – 1.91 (m, 1H, 3´-H), 1.85 – 1.73 (m, 2H, 4-H).

^{13}C NMR (101 MHz, $CDCl_3$) δ 198.0 (C=O), 174.5 (C=O), 161.0 (d, *J* = 246.2 Hz, Ar-C), 139.2 (Ar-C), 138.8 (Ar-C), 133.4 (Ar-CH), 132.6 (Ar-CH), 131.8 (d, *J* = 4.3 Hz, Ar-CH), 130.2 (2 × Ar-CH), 129.0 (d, *J* = 8.3 Hz, Ar-CH), 128.4 (Ar-CH), 125.8 (Ar-C), 125.1 (d, *J* = 14.6 Hz, Ar-C), 124.0 (d, *J* = 3.6 Hz, Ar-CH), 122.4 (Ar-CH), 121.6 (Ar-CH), 115.2 (d, *J* = 22.2 Hz, Ar-CH), 68.1 (CH), 53.9 (CH_2), 52.1 (CH_2), 31.2 (CH_2), 24.4 (CH_2).

^{19}F NMR (376 MHz, $CDCl_3$) δ –117.6.

Mp 85–87 °C.

$[\alpha]_D^{25}$ = 133.5 (c 0.1 in MeOH).

IR (υ_{max}/cm^{-1}, neat) 3275 (N-H), 1682 (C=O), 1645 (C=O), 1576 (C=C).

HRMS-EI (calcd for $C_{25}H_{23}FN_2O_2$ $[M + Na]^+$) 425.1636, found 425.1625 (Δ = - 2.5 ppm).

The spectroscopic data is in good agreement with the literature.[34]

S5 (7-Octyn-1-ol)

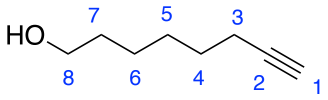

Ethylene diamine (45 mL) was added to a two-necked flask under N_2 and cooled to 0 °C. NaH (60%, 2.00 g, 55.6 mmol, 4 equiv.) was added in two portions and stirred at 0 °C for 15 mins, rt for 1 h and 60 °C for 1 h. The reaction mixture was cooled to 45 °C and 3-octyn-1-ol (1.76 g, 13.9 mmol, 1 equiv.) was added dropwise and left to stir for 1 h before stirring at 60 °C for 1 h. After quenching with H_2O (50 mL) at 0 °C, the mixture was acidified with 1 M $HCl_{(aq.)}$ (50 mL). The aqueous layer was extracted with EtOAc (3 × 200 mL) and Et_2O (3 × 200 mL) and combined organic layers washed with 1 M $HCl_{(aq.)}$ (3 × 200 mL), brine (3 × 200 mL), dried over $MgSO_4$ and concentrated to afford **S5** as a pale yellow oil (1.50 g, 85%).

^{1}**H NMR** (400 MHz, $CDCl_3$) δ 3.64 (t, *J* = 6.6 Hz, 2H, 8-H), 2.19 (td, *J* = 7.0 Hz, 2.7, 2H, 3-H), 1.94 (t, *J* = 2.7 Hz, 1H, 1-H), 1.61 – 1.33 (m, 8H, 4-H, 5-H, 6-H + 7-H).

^{13}**C NMR** (101 MHz, $CDCl_3$) δ 84.7 (C), 68.3 (CH), 63.0 (CH_2), 32.7 (CH_2), 28.6 (CH_2), 28.5 (CH_2), 25.4 (CH_2), 18.5 (CH_2).

IR (υ_{max}/cm^{-1}, neat) 3315 (OH), 2931 (C-H), 2854 (C-H).

The spectroscopic data is in good agreement with the literature.[35]

1 ((*S*)-((2-[1-(2-Fluorobenzyl)pyrrolidine-2-carboxamide]phenyl)phenylmethylene)-(*S*)-alaninato-*N*,*N'*,*N''*,O)nickel(II))

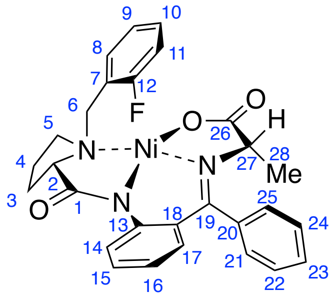

(*S*)-*N*-(2-benzoylphenyl)-1-(2-fluorobenzyl)pyrrolidine-2-carboxamide **S3** (1.00 g, 2.48 mmol, 1 equiv.), $Ni(NO_3)_2{\cdot}6H_2O$ (1.45 g, 4.97 mmol, 2 equiv.) and L-Ala (443 mg, 4.97 mmol, 2 equiv.) were dissolved in MeOH (25 mL) with heating at 50 °C. KOH (974 mg, 17.4 mmol, 7 equiv.) was added, the reaction mixture heated to 70 °C and left to stir for 1 h. After cooling and concentration *in vacuo,* the residue was taken up in H_2O (50 mL) and EtOAc (50 mL). The aqueous layer was extracted with EtOAc (3 × 100 mL), organic phases recombined and subsequently washed with brine (3 × 100 mL), dried with $MgSO_4$ and concentrated *in vacuo* to produce **1** as a red solid (1.30 g, 99%).

^{1}**H NMR** (400 MHz, $CDCl_3$) δ 8.31 (td, *J* = 7.5, 1.9 Hz, 1H, 9-H), 8.11 (d, *J* = 8.6 Hz, 1H, Ar-H), 7.52 – 7.43 (m, 3H, 3 × Ar-H), 7.25 – 7.14 (m, 4H, 8-H, 10-H + 2 × Ar-H), 7.07 – 7.03 (m, 1H, 11-H), 6.95 (d, *J* = 7.5 Hz, 1H, Ar-H), 6.69 – 6.63 (m, 2H, 2 × Ar-H), 4.40 (d, *J* = 12.9 Hz, 1H, 6-H), 3.91 (quin, *J* = 7.0 Hz, 1H, 27-H), 3.82 (d, *J* = 13.0 Hz, 1H, 6'-H), 3.75 – 3.65 (m, 1H, 5-H), 3.57

– 3.47 (m, 2H, 2-H + 4-H), 2.85 – 2.78 (m, 1H, 3-H), 2.61 – 2.52 (m, 1H, 3′-H), 2.24 – 2.19 (m, 1H, 5′-H), 2.09 – 2.03 (m, 1H, 4′-H), 1.58 (d, *J* = 7.0 Hz, 3H, 28-H).

^{13}C NMR (101 MHz, $CDCl_3$) δ 180.6 (C=O), 180.2 (C=O), 170.5 (C=N), 161.8 (d, *J* = 248.0 Hz, Ar-C), 142.2 (Ar-C), 134.3 (d, *J* = 3.4 Hz, Ar-CH), 133.6 (Ar-C), 133.3 (Ar-CH), 132.3 (Ar-CH), 131.4 (d, *J* = 8.5 Hz, Ar-CH), 129.8 (Ar-CH), 129.1 (2 × Ar-CH), 127.6 (Ar-CH), 127.4 (Ar-CH), 126.7 (Ar-C), 124.7 (d, *J* = 3.5 Hz, Ar-CH) 124.0 (Ar-CH), 121.0 (Ar-CH), 120.6 (d, *J* = 14.5 Hz, Ar-C), 116.3 (d, *J* = 22.4 Hz, Ar-CH), 70.5 (CH), 66.8 (C), 57.2 (CH_2), 55.7 (CH_2), 30.9 (CH_2), 24.3 (CH_2), 22.0 (CH_3).

^{19}F NMR (376 MHz, $CDCl_3$) δ –114.0.

Mp 280–282 °C.

$[\alpha]_D^{25}$ = +2904.3 (c 0.05 in $CHCl_3$) (lit: +3126.6).[36]

IR (υ_{max}/cm^{-1}, neat) 1680 (C=O), 1626 (C=N), 1549 (C=C), 1441 (C=C).

HRMS-EI (calcd for $C_{28}H_{26}FN_3NiO_3$ $[M + Na]^+$) 552.1204, found 552.1192 (Δ = - 2.2 ppm).

The spectroscopic data is in good agreement with the literature.[36]

2 ((*R*)-((2-[1-(2-Fluorobenzyl)pyrrolidine-2-carboxamide]phenyl)phenylmethylene)-(*R*)-alaninato-*N*,*N′*,*N′′*,O)nickel(II))

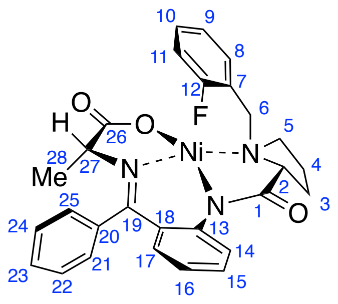

Method as **1** using (*R*)-*N*-(2-benzoylphenyl)-1-(2-fluorobenzyl)pyrrolidine-2-carboxamide **S4**, yielding **2** as a red solid (0.130 g, 99%).

^{1}H NMR (400 MHz, $CDCl_3$) δ 8.32 (td, *J* = 7.4, 1.9 Hz, 1H, 9-H), 8.12 (d, *J* = 8.8 Hz, 1H, Ar-H), 7.51 – 7.43 (m, 3H, 22-H, 23-H + Ar-H), 7.25 – 7.13 (m, 4H, 8-H, 10-H + 2 × Ar-H), 7.07 – 7.03 (m, 1H, 11-H), 6.96 – 6.94 (m, 1H, Ar-H), 6.69 – 6.62 (m, 2H, 2 × Ar-H), 4.40 (d, *J* = 13.8 Hz, 1H, 6-H), 3.90 (quin., *J* = 7.0 Hz, 1H, 27-H), 3.82 (d, *J* = 13.1 Hz, 1H, 6′-H), 3.76 – 3.63 (m, 1H, 5-H), 3.52 – 3.46 (m, 2H, 2-H + 4-H), 2.85 – 2.78 (m, 1H, 3-H), 2.61 – 2.51 (m, 1H, 3′-H), 2.28 – 2.18 (m, 1H, 5′-H), 2.10 – 2.03 (m, 1H, 4′-H), 1.57 (d, *J* = 7.0 Hz, 3H, 28-H).

^{13}C NMR (101 MHz, $CDCl_3$) δ 180.4 (C=O), 180.1 (C=O), 170.3 (C=N), 161.6 (d, *J* = 248.0 Hz, Ar-C), 142.1 (Ar-C), 134.2 (d, *J* = 3.4 Hz, Ar-CH), 133.5 (Ar-C), 133.2 (Ar-CH), 132.2 (Ar-CH), 131.2 (d, *J* = 8.5 Hz, Ar-CH), 129.7 (Ar-CH), 129.0 (2 × Ar-CH), 127.5 (Ar-CH), 127.2 (Ar-CH), 126.6 (Ar-C), 124.5 (d, *J* = 3.6 Hz, Ar-CH), 123.9 (Ar-CH), 120.8 (Ar-CH), 120.4 (d, *J* = 14.5 Hz, Ar-C), 116.1 (d, *J* = 22.3 Hz, Ar-CH), 70.4 (C), 66.6 (C), 57.1 (CH_2), 55.6 (CH_2), 30.7 (CH_2), 24.1 (CH_2), 21.8 (CH_3).

^{19}F NMR (376 MHz, $CDCl_3$) δ –114.0.

Mp 282–284 °C.

$[\alpha]_D^{25}$ = –2909.3 (c 0.05 in $CHCl_3$).

IR (υ_{max}/cm^{-1}, neat) 1670 (C=O), 1635 (C=N), 1550 (C=C).

HRMS-EI (calcd for $C_{28}H_{26}FN_3NiO_3$ $[M + Na]^+$) 552.1204, found 552.1180 (Δ = - 2.4 ppm).

3 ((*S*)-((2-[1-(2-Fluorobenzyl)pyrrolidine-2-carboxamide]phenyl)phenylmethylene)-(*S*)-glycinato-*N*,*N′*,*N′′*,O)nickel(II))

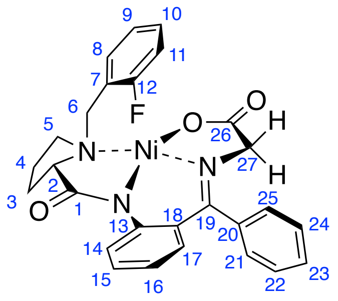

Method as **1** using *(R)*-*N*-(2-benzoylphenyl)-1-(2-fluorobenzyl)pyrrolidine-2-carboxamide **S3** and L-Gly, yielding **3** as a red solid (0.130 g, 94%).

^{1}H NMR (400 MHz, $CDCl_3$) δ 8.33 (dd, *J* = 7.5, 1.8 Hz, 1H, Ar-H), 8.29 (d, *J* = 8.7 Hz, 1H, Ar-H), 7.58 – 7.45 (m, 3H, 3 x Ar-H), 7.32 (m, 1H, Ar-H), 7.23 (m, 2H, 2 x Ar-H), 7.15 – 7.06 (m, 2H, 2 x Ar-H), 6.98 (m, 1H, Ar-H), 6.81 (d, *J* = 8.2 Hz, 1H, Ar-H), 6.71 (t, *J* = 7.6 Hz, 1H, Ar-H), 4.47 (d, *J* = 12.9 Hz, 1H), 3.93 (d, *J* = 12.9 Hz, 1H), 3.77 (d, *J* = 20.1 Hz, 1H), 3.67 (d, *J* = 20.1 Hz, 1H), 3.62 (m, 1H), 3.45 (dd, *J* = 10.8, 5.7 Hz, 1H), 3.42 – 3.27 (m, 1H), 2.72 – 2.59 (m, 1H), 2.54 – 2.39 (m, 1H), 2.18 – 2.04 (m, 2H, 27-H).

^{13}C NMR (101 MHz, $CDCl_3$) δ 181.1 (C=O), 177.4 (C=O), 171.8 (C=N), 161.9 (d, *J* = 248.0 Hz, Ar-CH), 142.6 (Ar-CH), 134.7 (Ar-CH), 134.3 (d, *J* = 3.3 Hz, (Ar-CH), 133.4 (Ar-CH), 132.4 (Ar-CH), 131.5 (d, *J* = 8.5 Hz, Ar-CH), 129.9 (Ar-CH), 129.8 (Ar-CH), 129.5 (Ar-CH), 126.4 (Ar-CH), 125.8 (Ar-CH), 125.4 (Ar-CH), 124.7 (d, *J* = 3.7 Hz, Ar-CH), 124.5 (Ar-CH), 121.1(Ar-CH), 120.6 (d, *J* = 14.5 Hz, Ar-CH), 116.3 (d, *J* = 22.4 Hz, Ar-CH), 70.2 (CH), 61.4 (C), 57.4 (CH_2), 55.9 (CH_2), 30.7 (CH_2), 23.8 (CH_2).

^{19}F NMR (376 MHz, $CDCl_3$) δ –113.7.

Mp 124-126 °C.

$[\alpha]_D^{25}$ = +1897.9 (c 0.05 in $CHCl_3$) (lit: +1300.0).[36]

IR (υ_{max}/cm^{-1}, neat) 2935, 2354, 1672, 1634, 1588, 1490, 1471, 1440, 1362, 1334, 1256, 1165, 1111, 1063, 962, 754, 723, 704.

HRMS-EI (calcd for $C_{28}H_{26}FN_3NiO_3$ $[M + H]^+$) 516.1228, found 516.1238 (Δ = - 2.0 ppm).

The spectroscopic data is in good agreement with the literature.[33, 36]

4 (5-Iodopent-1-yne)

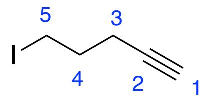

5-Chloro-pent-1-yne (775 µL, 7.31 mmol, 1 equiv.) was dissolved in acetone (15 mL) and NaI (5.49 g, 36.6 mmol, 5 equiv.) was added and left to stir for 16 h at 60 °C. After cooling and concentrating *in vacuo*, the residue was taken up in H_2O (50 mL) and EtOAc (50 mL). The aqueous layer was extracted with EtOAc (3 × 100 mL) and the organic layer was washed with $Na_2S_2O_{3(aq.)}$ (2 × 100 mL), dried over $MgSO_4$ and concentrated yielding **4** as a yellow oil (1.00 g, 70%).

^{1}H NMR: (400 MHz, $CDCl_3$) δ 3.31 (t, *J* = 6.7 Hz, 2H, 5-H), 2.34 (td, *J* = 6.7, 2.7 Hz, 2H, 3-H), 2.04-1.97 (m, 3H, 1-H + 4-H).

^{13}C NMR: (101 MHz, $CDCl_3$) δ 82.4 (C), 69.6 (CH), 32.0 (CH_2), 19.6 (CH_2), 5.1 (CH_2).

IR (υ_{max}/cm^{-1}, neat) 3250 (C≡C-H), 2910 (C-H), 2837 (C-H).

The spectroscopic data is in good agreement with the literature.[37]

5 (6-Iodohex-1-yne)

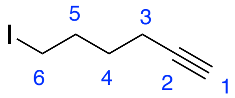

5-Hexyn-1-ol (1.00 g, 10.2 mmol, 1 equiv.) was dissolved in dry DCM (10 mL) and Et_3N (2.84 mL, 20.4 mmol, 2 equiv.) added. MsCl (1.75 mL, 15.3 mmol, 1.5 equiv.) was added dropwise at 0 °C and left to stir for 4 h at rt. After concentration, the residue was dissolved in H_2O, extracted with EtOAc (3 × 100 mL). The organic layer was washed with brine, dried over $MgSO_4$ and concentrated *in vacuo*. The crude mixture was dissolved in acetone (20 mL), NaI (7.63 g, 50.1 mmol, 5 equiv.) added and left to stir for 16 h at 60 °C. After cooling and concentrating, the residue was taken up in H_2O (100 mL) and EtOAc (100 mL). The aqueous

layer was extracted with EtOAc (3 × 100 mL) and the organic layer was washed with $Na_2S_2O_{3(aq.)}$ (2 × 100 mL), dried over $MgSO_4$ and concentrated yielding **5** as a yellow oil (1.82 g, 86%).

^{1}H NMR: (400 MHz, $CDCl_3$) δ 3.21 (t, *J* = 6.9 Hz, 2H, 6-H), 2.23 (td, *J* = 7.0, 2.7 Hz, 2H, 3-H), 1.99-1.92 (m, 3H, 1-H + 5-H), 1.68 – 1.61 (m, 2H, 4-H).

^{13}C NMR: (101 MHz, $CDCl_3$) δ 83.7 (C), 69.0 (CH), 32.4 (CH_2), 29.3 (CH_2), 17.6 (CH_2), 6.1 (CH_2).

IR (υ_{max}/cm^{-1}, neat) 3291 (C≡C-H), 2940 (C-H), 2860 (C-H).

The spectroscopic data is in good agreement with the literature.[38]

6 (7-Iodohept-1-yne)

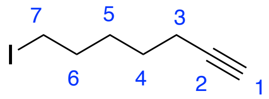

Method as **5**, using 6-Heptyn-1-ol (4.00 g, 35.7 mmol, 1 equiv.) yielding **6** as an orange oil (7.80 g, 99%).

^{1}H NMR: (400 MHz, $CDCl_3$) δ 3.19 (t, *J* = 7.0 Hz, 2H, 7-H), 2.21 (td, *J* = 6.8, 2.6 Hz, 2H, 3-H), 1.95 (t, *J* = 2.7 Hz, 1H, 1-H), 1.85 (quin, *J* = 7.1 Hz, 2H, 6-H), 1.60 – 1.48 (m, 4H, 4-H + 5-H).

^{13}C NMR: (101 MHz, $CDCl_3$) δ 84.3 (C), 68.7 (CH), 33.2 (CH_2), 29.7 (CH_2), 27.5 (CH_2), 18.4 (CH_2), 6.6 (CH_2).

IR (υ_{max}/cm^{-1}, neat) 3299 (C≡C-H), 2930 (C-H), 2855 (C-H).

HRMS-EI (calcd for $C_7H_{11}I$ $[M + H]^+$) 221.9906, found 221.9902 (Δ = -1.6 ppm).

The spectroscopic data is in good agreement with the literature.[39]

7 (8-Iodooct-1-yne)

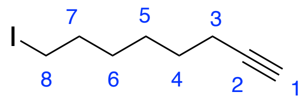

Method as **5** using 7-octyn-1-ol **S5** (1.32 g, 10.5 mmol, 1 equiv.), yielding **7** as a yellow oil (2.37 g, 96%).

^{1}H NMR (400 MHz, $CDCl_3$) δ 3.19 (t, *J* = 7.0 Hz, 2H, 8-H), 2.19 (td, *J* = 7.0 Hz, 2.7, 2H, 3-H), 1.94 (t, *J* = 2.6 Hz, 1H, 1-H), 1.87 – 1.80 (m, 2H, 4-H), 1.57 – 1.50 (m, 2H, 5-H), 1.47 – 1.38 (m, 4H, 4-H + 6-H).

^{13}C NMR (101 MHz, $CDCl_3$) δ 84.5 (C), 68.5 (CH), 33.5 (CH_2), 30.1 (CH_2), 28.3 (CH_2), 27.7 (CH_2), 18.5 (CH_2), 7.1 (CH_2).

IR (υ_{max}/cm^{-1}, neat) 3296 (C≡C-H), 2936 (C-H), 2860 (C-H).

The spectroscopic data is in good agreement with the literature.[35]

8 ((*S*)-((2-[1-(2-Fluorobenzyl)pyrrolidine-2-carboxamide]phenyl)phenylmethylene)-(*S*)-pentynylalaninato-*N*,*N'*,*N''*,O)nickel(II))

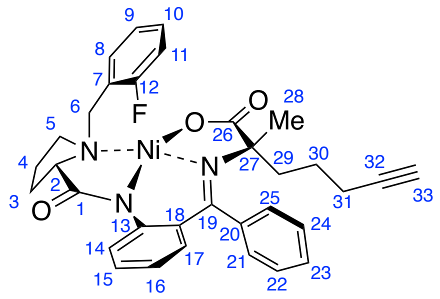

Sodium *t*-butoxide (0.750 g, 7.83 mmol, 1.2 equiv.) and L-Ala-Ni-(*S*)-FBPB **1** (3.45 g, 6.52 mmol, 1 equiv.) were added to a dry flask under N_2. The flask was cooled to 0 °C and dry DMF added (30 mL) before dropwise addition of 5-iodopent-1-yne **4** (1.52 g, 7.83 mmol, 1.2 equiv.). After 5 mins the ice bath was removed and the flask warmed to rt and stirred for 1 h. The reaction mixture was quenched by pouring into cold 5% acetic acid (50 mL) and concentrated *in vacuo*. The residue was dissolved in DCM (50 mL) and H_2O (50 mL) and the layers separated. The aqueous layer was extracted with DCM (50 mL) and the combined organic layers washed with 5% w/v $LiCl_{(aq.)}$ solution (3 × 50 mL), brine (3 × 50 mL), dried with $MgSO_4$ and concentrated *in vacuo*. Purification was carried out using normal phase flash column chromatography with a gradient of 50-100% EtOAc/Pet Ether to give **8** as a red solid (2.80 g, 72%).

^{1}H NMR (400 MHz, $CDCl_3$) δ 8.27 (td, *J* = 7.5, 1.8 Hz, 1H, 9-H), 8.03 (d, *J* = 8.2 Hz, 1H, Ar-H), 7.48 – 7.42 (m, 2H, 2 × Ar-H), 7.38 – 7.28 (m, 3H, 8-H + 2 × Ar-H), 7.18 (td, *J* = 7.5, 1.1 Hz, 1H, 10-H), 7.15 – 7.10 (m, 1H, 11-H), 7.07 – 7.04 (m, 2H, 2 × Ar-H), 6.66 – 6.59 (m, 2H, 2 × Ar-H), 4.48 (d, *J* = 13.1 Hz, 1H, 6-H), 3.92 (d, *J* = 13.1 Hz, 1H, 6′-H), 3.59 – 3.55 (m, 1H, 5-H), 3.40 (dd, *J* = 10.7, 6.4 Hz, 1H, 2-H), 3.31 – 3.18 (m, 1H, 4-H), 2.82 – 2.74 (m, 1H, 3-H), 2.60 – 2.46 (m, 2H, 3′-H +31-H), 2.38 – 2.29 (m, 1H, 30-H), 2.26 – 2.13 (m, 2H, 30′-H, 31′-H), 2.09 – 1.98 (m, 3H, 4′-H, 5′-H + 33-H), 1.94 – 1.86 (m, 1H, 29-H), 1.79 – 1.71 (m, 1H, 29′-H), 1.19 (s, 3H, 28-H).

^{13}C NMR (101 MHz, $CDCl_3$) δ 182.1 (C=O), 180.2 (C=O), 172.6 (C=N), 161.8 (d, *J*= 248.0 Hz, Ar-C), 141.6 (Ar-C), 136.5 (Ar-C), 134.2 (d, *J* = 3.4 Hz, Ar-CH), 133.6 (Ar-CH), 131.8 (Ar-CH), 131.3 (d, *J* = 8.5 Hz, Ar-CH), 130.4 (Ar-CH), 129.5 (Ar-CH), 128.6 (Ar-C), 128.1 (Ar-CH), 127.5 (Ar-CH), 127.0 (Ar-CH), 124.6 (d, *J* = 3.5 Hz, Ar-CH), 124.0 (Ar-CH), 120.8 (Ar-CH), 120.5 (d, *J* = 14.5 Hz, Ar-C), 116.2 (d, *J* = 22.3 Hz, Ar-CH), 83.6 (C), 77.8 (C), 70.3 (CH), 69.3 (CH), 56.8 (CH_2), 56.0 (CH_2), 39.8 (CH_2), 30.6 (CH_2), 29.4 (CH_3), 25.0 (CH_2), 23.4 (CH_2), 18.6 (CH_2).

^{19}F NMR (377 MHz, $CDCl_3$) δ –113.7.

Mp 170–172 °C.

$[\alpha]_D^{25}$ = +2050 (c 0.05 in MeOH).

IR (υ_{max}/cm^{-1}, neat) 3287 (C≡C-H), 2933 (C-H), 1665 (C=O), 1634 (C=N).

HRMS-EI (calcd for $C_{33}H_{32}FN_3NiO_3$ [M + Na]$^+$) 618.1673, found 618.1674 (Δ = 0.0 ppm).

9 ((*S*)-((2-[1-(2-Fluorobenzyl)pyrrolidine-2-carboxamide]phenyl)phenylmethylene)-(*S*)-hexynylalaninato-*N*,*N'*,*N''*,O)nickel(II))

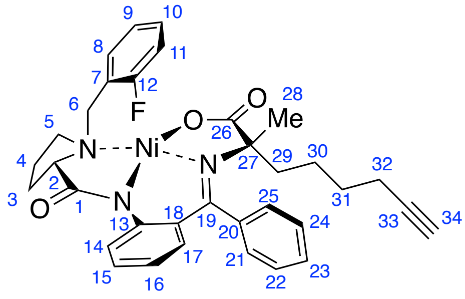

Method as **8** using L-Ala-Ni-(*S*)-FBPB **1** (3.18 g, 6.01 mmol, 1 equiv.) and 6-iodohex-1-yne **5** (1.50 g, 7.21 mmol, 1.2 equiv.) yielding **9** as a red solid (2.80 g, 76%).

^{1}H NMR (400 MHz, $CDCl_3$) δ 8.28 (td, *J* = 7.4, 1.8 Hz, 1H, 9-H), 8.00 (d, *J* = 8.7 Hz, 1H, Ar-H), 7.48 – 7.43 (m, 2H, 2 × Ar-H), 7.39 – 7.27 (m, 3H, 8-H + 2 × Ar-H), 7.21–7.05 (m, 3H, 10-H, 11-H + Ar-H), 7.01 – 6.98 (m, 1H, Ar-H), 6.66 – 6.60 (m, 2H, 2 × Ar-H), 4.49 (d, *J* = 13.0 Hz, 1H, 6-H), 3.93 (d, *J* = 13.0 Hz, 1H, 6'-H), 3.61 – 3.57 (m, 1H, 5-H), 3.39 (dd, *J* = 10.6, 6.4 Hz, 1H, 2-H), 3.34 – 3.21 (m, 1H, 4-H), 2.81 – 2.73 (m, 1H, 3-H), 2.57 – 2.45 (m, 2H, 3'-H + 30-H), 2.28 (td, *J* = 6.9, 2.6 Hz, 2H, 32-H), 2.13 – 1.99 (m, 3H, 4'-H, 5'-H, 30'-H), 1.96 (t, *J* = 2.6 Hz, 1H, 34-H), 1.68 (m, 1H, 29-H), 1.61 – 1.41 (m, 3H, 29**'**-H + 31-H), 1.25 (s, 3H, 28-H).

^{13}C NMR (101 MHz, $CDCl_3$) δ 182.5 (C=O), 180.2 (C=O), 172.7 (C=N), 161.8 (d, *J*= 247.9 Hz, Ar-C), 141.6 (Ar-C), 136.7 (Ar-C), 134.3 (d, *J* = 3.4 Hz, Ar-CH), 133.4 (Ar-CH), 131.6 (Ar-CH), 131.3 (d, *J* = 8.5 Hz, Ar-CH), 130.3 (Ar-CH), 129.5 (Ar-CH), 128.9 (Ar-C), 128.1 (Ar-CH), 127.4 (Ar-CH), 127.0 (Ar-CH), 124.6 (d, *J* = 3.6 Hz, Ar-CH), 124.1 (Ar-CH), 120.8 (Ar-CH), 120.6 (d, *J* = 14.5 Hz, Ar-C), 116.2 (d, *J* = 22.4 Hz, Ar-CH), 84.0 (C), 78.1 (C), 70.3 (CH), 69.2 (CH), 56.9 (CH_2), 56.0 (CH_2), 39.5 (CH_2), 30.7 (CH_2), 29.9 (CH_3), 28.4 (CH_2), 25.0 (CH_2), 23.4 (CH_2), 18.5 (CH_2).

^{19}F NMR (377 MHz, $CDCl_3$) δ –113.7.

Mp 119–121 °C.

[α]$_D^{25}$ = –1940 (c 0.05 in MeOH).

IR (υ_{max}/cm^{-1}, neat) 3294 (C≡C-H), 2934 (C-H), 1665 (C=O), 1638 (C=N).

HRMS-EI (calcd for $C_{34}H_{34}FN_3NiO_3$ [M + Na]$^+$) 632.1830, found 632.1821 (Δ = –1.4 ppm).

10 ((*S*)-((2-[1-(2-Fluorobenzyl)pyrrolidine-2-carboxamide]phenyl)phenylmethylene)-(*S*)-heptynylalaninato-*N*,*N'*,*N''*,O)nickel(II))

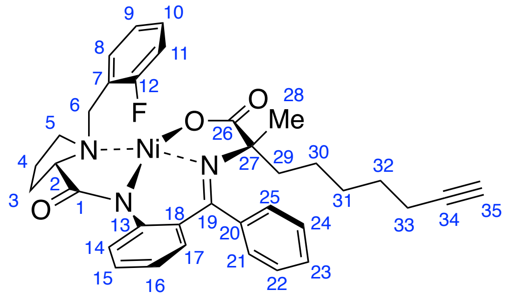

Method as **8** using L-Ala-Ni-(*S*)-FBPB **1** (4.76 g, 9.00 mmol, 1 equiv.) and 7-iodohept-1-yne **6** (3.00 g, 13.5 mmol, 1.2 equiv.) yielding **10** as a red solid (5.90 g, 72%).

^{1}H NMR (400 MHz, CDCl$_3$) δ 8.29 (td, *J* = 7.5, 1.8 Hz, 1H, 9-H), 8.02 (d, *J* = 8.2 Hz, 1H, Ar-H), 7.50 – 7.44 (m, 2H, 2 × Ar-H), 7.40 – 7.27 (m, 3H, 8-H + 2 × Ar-H), 7.20 (td, *J* = 7.5, 1.2 Hz, 1H, 10-H), 7.16 – 7.06 (m, 2H, 11-H + Ar-H), 6.98 – 6.96 (m, 1H, Ar-H), 6.65 – 6.61 (m, 2H, 2 × Ar-H), 4.51 (d, *J* = 13.1 Hz, 1H, 6-H), 3.94 (d, *J* = 13.1 Hz, 1H, 6'-H), 3.61 – 3.57 (m, 1H, 5-H), 3.41 (dd, *J* = 10.7, 6.3 Hz, 1H, 2-H), 3.32 – 3.20 (m, 1H, 4-H), 2.80 – 2.72 (m, 1H, 3-H), 2.57 – 2.47 (m, 1H, 3'-H), 2.39 – 2.29 (m, 1H, 30-H), 2.23 (td, *J* = 6.9, 2.4 Hz, 2H, 33-H), 2.13 – 1.97 (m, 3H, 4'-H, 5'-H + 30'-H), 1.95 (t, *J* = 2.6 Hz, 1H, 35-H), 1.74 – 1.55 (m, 4H, 29-H + 32-H), 1.47 – 1.37 (m, 2H, 31-H), 1.24 (s, 3H, 28-H).

^{13}C NMR (101 MHz, CDCl$_3$) δ 182.5 (C=O), 180.3 (C=O), 172.6 (C=N), 161.9 (d, *J* = 248.0 Hz, Ar-C), 141.7 (Ar-C), 136.7 (Ar-C), 134.4 (d, *J* = 3.3 Hz, Ar-CH), 133.6 (Ar-CH), 131.7 (Ar-CH), 131.4 (d, *J* = 8.5 Hz, Ar-CH), 130.4 (Ar-CH), 129.6 (Ar-CH), 128.9 (Ar-C), 128.1 (Ar-CH), 127.4 (Ar-CH), 127.1 (Ar-CH), 124.7 (d, *J* = 3.6 Hz, Ar-CH), 124.1 (Ar-CH), 120.9 (Ar-CH), 120.6 (d, *J* = 14.4 Hz,

Ar-C), 116.3 (d, J = 22.4 Hz, Ar-CH), 84.4 (C), 78.3 (C), 70.3 (CH), 68.7 (CH), 56.9 (CH_2), 56.0 (CH_2), 40.1 (CH_2), 30.7 (CH_2), 29.8 (CH_3), 28.9 (CH_2), 28.4 (CH_2), 25.5 (CH_2), 23.4 (CH_2), 18.6 (CH_2).

^{19}F NMR (377 MHz, $CDCl_3$) δ –113.7.

Mp 128–130 °C.

$[\alpha]_D^{25}$ = +3470 (c 0.05 in MeOH).

IR (υ_{max}/cm^{-1}, neat) 3300 (C≡C-H), 2930 (C-H), 1719 (C=O), 1670 (C=N).

HRMS-EI (calcd for $C_{35}H_{36}FN_3NiO_3$ $[M + Na]^+$) 646.1986, found 646.1963 (Δ = –3.7 ppm).

11 ((*S*)-((2-[1-(2-Fluorobenzyl)pyrrolidine-2-carboxamide]phenyl)phenylmethylene)-(*S*)-octynylalaninato-*N*,*N'*,*N''*,O)nickel(II))

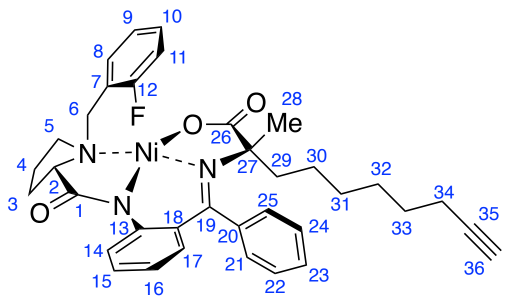

Method as **8** using L-Ala-Ni-(*S*)-FBPB **1** (6.50 g, 12.3 mmol, 1 equiv.) and 8-iodooct-1-yne **7** (3.50 g, 14.8 mmol, 1.2 equiv.) yielding **11** as a red solid (5.90 g, 75%).

^{1}H NMR (400 MHz, $CDCl_3$) δ 8.29 (td, J = 7.5, 1.7 Hz, 1H, 9-H), 8.03 (d, J = 8.3 Hz, 1H, Ar-H), 7.50 – 7.44 (m, 2H, 2 × Ar-H), 7.41 – 7.28 (m, 3H, 8-H + 2 × Ar-H), 7.20 (td, J = 7.5, 1.3 Hz, 1H, 10-H), 7.17 – 7.07 (m, 2H, 11-H + Ar-H), 6.97 – 6.95 (m, 1H, Ar-H), 6.67 – 6.62 (m, 2H, 2 × Ar-H), 4.52 (d, J = 13.3 Hz, 1H, 6-H), 3.95 (d, J = 13.3 Hz, 1H, 6′-H), 3.62 – 3.58 (m, 1H, 5-H), 3.41 (dd, J = 10.7, 6.4 Hz, 1H, 2-H), 3.34 – 3.22 (m, 1H, 4-H), 2.82 – 2.73 (m, 1H, 3-H), 2.58 – 2.47 (m, 1H, 3′-H), 2.40 – 2.29 (m, 1H, 30-H), 2.21 (td, J = 6.8, 2.6 Hz, 2H, 34-H), 2.14 – 1.97 (m, 3H,

4′-H, 5′-H, 30′-H), 1.95 (t, J = 2.6 Hz, 1H, 36-H), 1.70 (m, 1H, 29-H) 1.62 – 1.49 (m, 5H, 29′-H, 32-H + 33-H), 1.37 – 1.28 (m, 2H, 31-H), 1.24 (s, 3H, 28-H).

^{13}C NMR (101 MHz, $CDCl_3$) δ 182.6 (C=O), 180.3 (C=O), 172.5 (C=N), 161.9 (d, J = 247.9 Hz, Ar-C), 141.6 (Ar-C), 136.7 (Ar-C), 134.4 (d, J = 3.3 Hz, Ar-CH), 133.6 (Ar-CH), 131.7 (Ar-CH), 131.4 (d, J = 8.4 Hz, Ar-CH), 130.5 (Ar-CH), 129.5 (Ar-CH), 129.0 (Ar-C), 128.1 (Ar-CH), 127.4 (Ar-CH), 127.1 (Ar-CH), 124.7 (d, J = 3.5 Hz, Ar-CH), 124.1 (Ar-CH), 120.9 (Ar-CH), 120.6 (d, J = 14.5 Hz, Ar-C), 116.3 (d, J = 22.3 Hz, Ar-CH), 84.6 (C), 78.4 (C), 70.3 (CH), 68.5 (CH), 56.8 (CH_2), 56.0 (CH_2), 40.3 (CH_2), 30.7 (CH_2), 29.9 (28-CH_3), 29.4 (CH_2), 28.9 (CH_2), 28.6 (CH_2), 26.1 (CH_2), 23.4 (CH_2), 18.5 (CH_2).

^{19}F NMR (377 MHz, $CDCl_3$) δ –113.7.

Mp 107–109 °C.

$[\alpha]_D^{25}$ = +3000 (c 0.05 in MeOH).

IR (υ_{max}/cm^{-1}, neat) 3305 (C≡C-H) 2928 (C-H), 1736 (C=O), 1670 (C=N).

HRMS-EI (calcd for $C_{36}H_{38}FN_3NiO_3$ $[M + Na]^+$) 660.2143, found 660.2123 (Δ = –3.0 ppm).

12 ((*S*)-((2-[1-(2-Fluorobenzyl)pyrrolidine-2-carboxamide]phenyl)phenylmethylene)-(*S*)-heptynylglycinato-*N*,*N′*,*N″*,O)nickel(II))

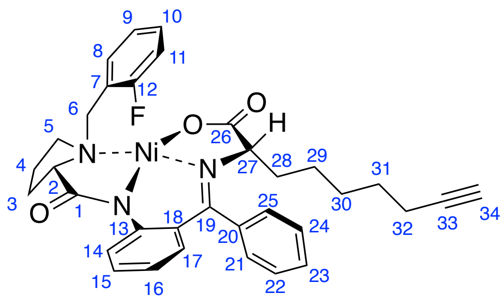

To ground NaOH (1.20 g, 30.0 mmol, 5.0 equiv.) was added dry DMF (15 mL) under N_2 and the flask cooled to 0 °C. A solution of L-Gly-Ni-(*S*)-FBPB **116** complex (3.10 g, 6.00 mmol, 1 equiv.) in dry DMF (10 mL) was added and the flask removed from the ice bath. 8-Iodooct-1-

yne **7** (1.47 g, 6.60 mmol, 1.1 equiv.) was added dropwise and the solution left to stir for 30 mins at rt. The reaction mixture was quenched by pouring into H_2O (50 mL) and concentrated *in vacuo*. The residue was dissolved in DCM (100 mL) and H_2O (100 mL) and the layers separated. The aqueous layer was extracted with DCM (100 mL) and the combined organic layers washed with 5% w/v $LiCl_{(aq.)}$ solution (3 × 100 mL), brine (3 × 100 mL), dried with $MgSO_4$ and concentrated *in vacuo*. Purification was carried out using normal phase flash column chromatography with a gradient of 50-100% EtOAc/Pet Ether to give **12** as a red solid (2.89 g, 79%).

^{1}H NMR (400 MHz, $CDCl_3$) δ 8.28 (td, *J* = 7.4, 1.8 Hz, 1H, 9-H), 8.16 – 8.14 (m, 1H, Ar-H), 7.52 – 7.44 (m, 3H, 3 × Ar-H), 7.24 – 7.14 (m, 4H, 8-H, 10-H + 2 × Ar-H), 7.07 – 7.02 (m, 1H, 11-H), 6.94 – 6.92 (m, 1H, Ar-H), 6.70 – 6.63 (m, 2H, 2 × Ar-H), 4.45 (d, *J* = 13.6 Hz, 1H, 6-H), 3.90 (dd, *J* = 8.3, 3.4 Hz, 1H, 27-H), 3.85 (d, *J* = 13.6 Hz, 1H, 6′-H), 3.61 – 3.52 (m, 1H, 5-H), 3.49 – 3.45 (m, 2H, 2-H + 4-H), 2.87 – 2.80 (m, 1H, 3-H), 2.62– 2.51 (m, 1H, 3′-H), 2.19– 1.96 (m, 5H, 4′-H, 5′-H, 28-H + 32-H), 1.93 (t, *J* = 2.6 Hz, 1H, 34-H), 1.66 – 1.55 (m, 3H, 28′-H + 30-H), 1.51– 1.44 (m, 2H, 31-H), 1.33– 1.18 (m, 2H, 29-H).

^{13}C NMR (101 MHz, $CDCl_3$) δ 180.0 (C=O), 179.3 (C=O), 170.3 (C=N), 161.6 (d, *J* = 247.9 Hz, Ar-C), 142.2 (Ar-C), 134.2 (d, *J* = 3.1 Hz Ar-C), 133.8 (Ar-CH), 133.3 (Ar-CH), 132.2 (Ar-CH), 131.2 (d, *J* = 8.4 Hz, Ar-CH), 129.7 (Ar-CH), 129.0 (Ar-C + Ar-CH), 127.5 (Ar-CH), 127.2 (Ar-CH), 126.6 (Ar-CH), 124.5 (d, *J* = 3.4 Hz, Ar-CH), 123.7 (Ar-CH), 120.8 (Ar-C), 120.4 (d, *J* = 14.5 Hz, Ar-CH), 116.1 (d, *J* = 22.1 Hz, Ar-CH), 84.3 (C), 70.4 (CH), 70.3 (CH), 68.4 (C), 56.8 (CH_2), 55.6 (CH_2), 35.2 (CH_2), 30.7 (3-CH_2), 28.4 (CH_3), 28.1 (CH_2), 24.9 (CH_2), 23.7 (CH_2), 18.3 (CH_2).

^{19}F NMR (377 MHz, $CDCl_3$) δ –113.9.

Mp 168–170 °C.

$[\alpha]_D^{25}$ = +2494 (c 0.05 in MeOH).

IR (υ_{max}/cm^{-1}, neat) 3240 (C≡C-H) 2934 (C-H), 1670 (C=O), 1634 (C=N).

HRMS-EI (calcd for $C_{34}H_{34}FN_3NiO_3$ $[M + Na]^+$) 632.1835, found 632.1840 (Δ = 0.8 ppm).

13 ((*R*)-((2-[1-(2-Fluorobenzyl)pyrrolidine-2-carboxamide]phenyl)phenylmethylene)-(*R*)-pentynylalaninato-*N*,*N'*,*N''*,O)nickel(II))

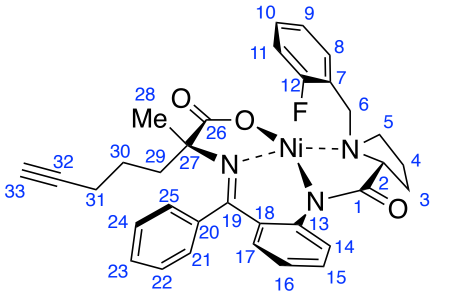

Method as **8** using D-Ala-Ni-(*R*)-FBPB **2** (1.59 g, 3.00 mmol, 1 equiv.) and 5-iodopent-1-yne **4** (0.700 g, 3.61 mmol, 1.2 equiv.) to give **13** as a red solid (1.25 g, 70%).

^{1}H NMR (400 MHz, $CDCl_3$) δ 8.29 (td, *J* = 7.5, 1.8 Hz, 1H, 9-H), 8.04 (d, *J* = 8.4 Hz, 1H, Ar-H), 7.48 – 7.43 (m, 2H, 2 × Ar-H), 7.39 – 7.27 (m, 3H, 8-H + 2 × Ar-H), 7.19 (td, *J* = 7.5, 1.2 Hz, 1H, 10-H), 7.16 – 7.12 (m, 1H, 11-H), 7.11 – 7.05 (m, 2H, 2 × Ar-H), 6.67 – 6.60 (m, 2H, 2 × Ar-H), 4.49 (d, *J* = 13.1 Hz, 1H, 6-H), 3.94 (d, *J* = 13.1 Hz, 1H, 6'-H), 3.61 – 3.57 (m, 1H, 5-H), 3.41 (dd, *J* = 10.7, 6.4 Hz, 1H, 2-H), 3.32 – 3.19 (m, 1H, 4-H), 2.83 – 2.76 (m, 1H, 3-H), 2.60 – 2.47 (m, 2H, 3'-H +31-H), 2.39 – 2.30 (m, 1H, 30-H), 2.27 – 2.14 (m, 2H, 30'-H, 31'-H), 2.10 – 1.99 (m, 3H, 4'-H, 5'-H + 33-H), 1.95 – 1.87 (m, 1H, 29-H), 1.72 – 1.62 (m, 1H, 29'-H), 1.20 (s, 3H, 28-H).

^{13}C NMR (101 MHz, $CDCl_3$) δ 182.1 (C=O), 180.2 (C=O), 172.7 (C=N), 161.8 (d, *J*= 248.0 Hz, Ar-C), 141.7 (Ar-C), 136.5 (Ar-C), 134.3 (d, *J* = 3.3 Hz, Ar-CH), 133.6 (Ar-CH), 131.8 (Ar-CH), 131.4 (d, *J* = 8.5 Hz, Ar-CH), 130.5 (Ar-CH), 129.5 (Ar-CH), 128.7 (Ar-C), 128.2 (Ar-CH), 127.6 (Ar-CH), 127.0 (Ar-CH), 124.6 (d, *J* = 3.6 Hz, Ar-CH), 124.0 (Ar-CH), 120.9 (Ar-CH), 120.6 (d, *J* = 14.4 Hz, Ar-C), 116.3 (d, *J* = 22.3 Hz, Ar-CH), 83.7 (C), 77.9 (C), 70.3 (CH), 69.3 (CH), 56.9 (CH_2), 56.1 (CH_2), 39.9 (CH_2), 30.7 (CH_2), 29.5 (CH_3), 25.0 (CH_2), 23.4 (CH_2), 18.7 (CH_2).

^{19}F NMR (377 MHz, $CDCl_3$) δ –113.7.

Mp 171–173 °C.

[α]$_D^{25}$ = –2190 (c 0.05 in MeOH).

IR (υ_{max}/cm^{-1}, neat) 3288 (C≡C-H), 2936 (C-H), 1662 (C=O), 1631 (C=N).

HRMS-EI (calcd for $C_{33}H_{32}FN_3NiO_3$ [M + Na]$^+$) 618.1673, found 618.1690 (Δ = 2.8 ppm).

14 ((*R*)-((2-[1-(2-Fluorobenzyl)pyrrolidine-2-carboxamide]phenyl)phenylmethylene)-(*R*)-hexynylalaninato-*N*,*N'*,*N''*,O)nickel(II))

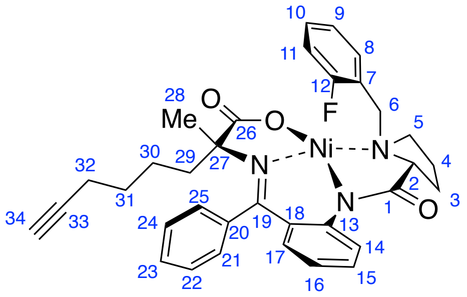

Method as **8** using D-Ala-Ni-(*R*)-FBPB **2** (1.59 g, 3.00 mmol, 1 equiv.) and 6-iodohex-1-yne **5** (715 mg, 3.61 mmol, 1.2 equiv.) yielding **14** as a red solid (1.23 g, 69%).

^{1}H NMR (400 MHz, $CDCl_3$) δ 8.27 (td, *J* = 7.5, 1.8 Hz, 1H, 9-H), 7.99 (d, *J* = 8.7 Hz, 1H, Ar-H), 7.49 – 7.44 (m, 2H, 2 × Ar-H), 7.40 – 7.27 (m, 3H, 8-H + 2 × Ar-H), 7.21–7.06 (m, 3H, 10-H, 11-H + Ar-H), 7.01 – 6.98 (m, 1H, Ar-H), 6.67–6.60 (m, 2H, 2 × Ar-H), 4.50 (d, *J* = 13.1 Hz, 1H, 6-H), 3.93 (d, *J* = 13.1 Hz, 1H, 6'-H), 3.60 – 3.55 (m, 1H, 5-H), 3.39 (dd, *J* = 10.7, 6.3 Hz, 1H, 2-H), 3.34 – 3.22 (m, 1H, 4-H), 2.81 – 2.73 (m, 1H, 3-H), 2.58 – 2.45 (m, 2H, 3'-H + 30-H), 2.28 (td, *J* = 6.9, 2.7 Hz, 2H, 32-H), 2.13 – 1.98 (m, 3H, 4'-H, 5'-H, 30'-H), 1.97 (t, *J* = 2.6 Hz, 1H, 34-H), 1.72 – 1.62 (m, 1H, 29-H), 1.61 – 1.41 (m, 3H, 29**'**-H + 31-H), 1.25 (s, 3H, 28-H).

^{13}C NMR (101 MHz, $CDCl_3$) δ 182.6 (C=O), 180.2 (C=O), 172.7 (C=N), 161.8 (d, *J*= 248.0 Hz, Ar-C), 141.5 (Ar-C), 136.6 (Ar-C), 134.3 (d, *J* = 3.3 Hz, Ar-CH), 133.4 (Ar-CH), 131.7 (Ar-CH), 131.3 (d, *J* = 8.4 Hz, Ar-CH), 130.3 (Ar-CH), 129.5 (Ar-CH), 128.9 (Ar-C), 128.1 (Ar-CH), 127.3 (Ar-CH), 127.0 (Ar-CH), 124.6 (d, *J* = 3.6 Hz, Ar-CH), 124.1 (Ar-CH), 120.9 (Ar-CH), 120.5 (d, *J* = 14.5 Hz, Ar-C), 116.2 (d, *J* = 22.3 Hz, Ar-CH), 84.0 (C), 78.1 (C), 70.2 (CH), 69.2 (CH), 56.9 (CH_2), 56.0 (CH_2), 39.5 (CH_2), 30.6 (CH_2), 29.9 (CH_3), 28.4 (CH_2), 25.0 (CH_2), 23.4 (CH_2), 18.5 (CH_2).

^{19}F NMR (377 MHz, $CDCl_3$) δ –113.7.

Mp 120–122 °C.

[*α*]$_D^{25}$ = +1838 (c 0.05 in MeOH).

IR (υ_{max}/cm^{-1}, neat) 3292 (C≡C-H), 2933 (C-H), 1665 (C=O), 1636 (C=N).

HRMS-EI (calcd for $C_{34}H_{34}FN_3NiO_3$ $[M + Na]^+$) 632.1830, found 632.1822 (Δ = –1.3 ppm).

15 ((*R*)-((2-[1-(2-Fluorobenzyl)pyrrolidine-2-carboxamide]phenyl)phenylmethylene)-(*R*)-heptynylalaninato-*N*,*N'*,*N''*,O)nickel(II))

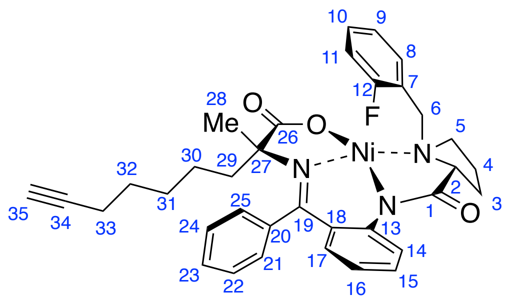

Method as **8** using D-Ala-Ni-(*R*)-FBPB **2** (4.76 g, 9.00 mmol, 1 equiv.) and 7-iodohept-1-yne **6** (3.00 g, 13.5 mmol, 1.2 equiv.) yielding **15** as a red solid (4.06 g, 72%).

^{1}H NMR (400 MHz, $CDCl_3$) δ 8.29 (td, *J* = 7.5, 1.8 Hz, 1H, 9-H), 8.02 (d, *J* = 8.2 Hz, 1H, Ar-H), 7.49 – 7.44 (m, 2H, 2 × Ar-H), 7.40 – 7.27 (m, 3H, 8-H + 2 × Ar-H), 7.20 (td, *J* = 7.5, 1.3 Hz, 1H, 10-H), 7.15 – 7.06 (m, 2H, 11-H + Ar-H), 6.98 – 6.95 (m, 1H, Ar-H), 6.65 – 6.66 (m, 2H, 2 × Ar-H), 4.50 (d, *J* = 13.1 Hz, 1H, 6-H), 3.93 (d, *J* = 13.1 Hz, 1H, 6'-H), 3.61 – 3.57 (m, 1H, 5-H), 3.40 (dd, *J* = 10.7, 6.3 Hz, 1H, 2-H), 3.32 – 3.19 (m, 1H, 4-H), 2.78 – 2.71 (m, 1H, 3-H), 2.57 – 2.46 (m, 1H, 3'-H) 2.39 – 2.29 (m, 1H, 30-H), 2.23 (td, *J* = 6.9, 2.4 Hz, 2H, 33-H), 2.12 – 1.97 (m, 3H, 4'-H, 5'-H + 30'-H), 1.94 (t, *J* = 2.6 Hz, 1H, 35-H), 1.74 – 1.53 (m, 4H, 29-H + 32-H), 1.46 – 1.37 (m, 2H, 31-H), 1.23 (s, 3H, 28-H).

^{13}C NMR (101 MHz, $CDCl_3$) δ 182.5 (C=O), 180.2 (C=O), 172.5 (C=N), 161.8 (d, *J* = 248.0 Hz, Ar-C), 141.6 (Ar-C), 136.7 (Ar-C), 134.3 (d, *J* = 3.3 Hz, Ar-CH), 133.5 (Ar-CH), 131.7 (Ar-CH), 131.3

(d, J = 8.4 Hz, Ar-CH), 130.4 (Ar-CH), 129.5 (Ar-CH), 128.9 (Ar-C), 128.1 (Ar-CH), 127.3 (Ar-CH), 127.0 (Ar-CH), 124.6 (d, J = 3.6 Hz, Ar-CH), 124.1 (Ar-CH), 120.9 (Ar-CH), 120.5 (d, J = 14.5 Hz, Ar-C), 116.2 (d, J = 22.3Hz, Ar-CH), 84.3 (C), 78.2 (C), 70.2 (CH), 68.6 (CH), 56.8 (CH_2), 56.0 (CH_2), 40.1 (CH_2), 30.7 (CH_2), 29.8 (CH_3), 28.8 (CH_2), 28.4 (CH_2), 25.5 (CH_2), 23.4 (CH_2), 18.5 (CH_2).

^{19}F NMR (377 MHz, $CDCl_3$) δ –113.7.

Mp 127–129 °C.

$[\alpha]_D^{25}$ = –2994 (c 0.05 in MeOH).

IR (υ_{max}/cm^{-1}, neat) 3310 (C≡C-H), 2930 (C-H), 1719 (C=O), 1673 (C=N).

HRMS-EI (calcd for $C_{35}H_{36}FN_3NiO_3$ $[M + Na]^+$) 646.1986, found 646.1954 (Δ = –5.0 ppm).

16 ((*R*)-((2-[1-(2-Fluorobenzyl)pyrrolidine-2-carboxamide]phenyl)phenylmethylene)- (*R*)-octynylalaninato-*N*,*N'*,*N''*,O)nickel(II))

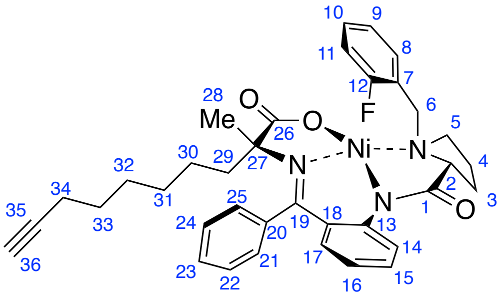

Method as **8** using D-Ala-Ni-(*R*)-FBPB **2** (4.35 g, 8.22 mmol, 1 equiv.) and 8-iodooct-1-yne **7** (2.33 g, 9.87 mmol, 1.2 equiv.) yielding **16** as a red solid (3.87 g, 75%).

^{1}H NMR (400 MHz, $CDCl_3$) δ 8.29 (td, J = 7.4, 1.4 Hz, 1H, 9-H), 8.03 (d, J = 8.5 Hz, 1H, Ar-H), 7.50 – 7.45 (m, 2H, 2 × Ar-H), 7.41 – 7.28 (m, 3H, 8-H + 2 × Ar-H), 7.20 (td, J = 7.5, 1.2 Hz, 1H, 10-H), 7.17 – 7.07 (m, 2H, 11-H + Ar-H), 6.97 – 6.95 (m, 1H, Ar-H), 6.67 – 6.62 (m, 2H, 2 × Ar-

H), 4.52 (d, *J* = 13.1 Hz, 1H, 6-H), 3.95 (d, *J* = 13.1 Hz, 1H, 6′-H), 3.62 – 3.58 (m, 1H, 5-H), 3.41 (dd, *J* = 10.8, 6.4 Hz, 1H, 2-H), 3.34 – 3.21 (m, 1H, 4-H), 2.81 – 2.73 (m, 1H, 3-H), 2.58 – 2.47 (m, 1H, 3′-H), 2.40 – 2.29 (m, 1H, 30-H), 2.21 (td, *J* = 6.7, 2.6 Hz, 2H, 34-H), 2.13 – 1.97 (m, 3H, 4′-H, 5′-H, 30′-H), 1.95 (t, *J* = 2.6 Hz, 1H, 36-H), 1.70 (td, *J* = 13.5, 13.0, 4.7 Hz, 1H, 29-H) 1.62 – 1.48 (m, 5H, 29′-H, 32-H + 33-H), 1.37 – 1.28 (m, 2H, 31-H), 1.24 (s, 3H, 28-H).

^{13}C NMR (101 MHz, $CDCl_3$) δ 182.6 (C=O), 180.3 (C=O), 172.5 (C=N), 161.9 (d, *J* = 247.9 Hz, Ar-C), 141.6 (Ar-C), 136.7 (Ar-C), 134.4 (d, *J* = 3.3 Hz, Ar-CH), 133.6 (Ar-CH), 131.7 (Ar-CH), 131.4 (d, *J* = 8.4 Hz, Ar-CH), 130.5 (Ar-CH), 129.5 (Ar-CH), 129.0 (Ar-C), 128.1 (Ar-CH), 127.4 (Ar-CH), 127.1 (Ar-CH), 124.7 (d, *J* = 3.6 Hz, Ar-CH), 124.1 (Ar-CH), 120.9 (Ar-CH), 120.6 (d, *J* = 14.5 Hz, Ar-C), 116.3 (d, *J* = 22.3 Hz, Ar-CH), 84.6 (C), 78.4 (C), 70.3 (CH), 68.5 (CH), 56.8 (CH_2), 56.0 (CH_2), 40.3 (CH_2), 30.7 (CH_2), 29.9 (CH_3), 29.4 (CH_2), 28.9 (CH_2), 28.6 (CH_2), 26.1 (CH_2), 23.4 (CH_2), 18.5 (CH_2).

^{19}F NMR (377 MHz, $CDCl_3$) δ –113.7.

Mp 104–106 °C.

$[\alpha]_D^{25}$ = –3040 (c 0.05 in MeOH).

IR (υ_{max}/cm^{-1}, neat) 3303 (C≡C-H) 2936 (C-H), 1736 (C=O), 1670 (C=N).

HRMS-EI (calcd for $C_{36}H_{38}FN_3NiO_3$ $[M + Na]^+$) 660.2143, found 660.2116 (Δ = –4.1 ppm).

17 ((*S*)-2-((((9*H*-fluoren-9-yl)methoxy)carbonyl)amino)-2-methylhept-6-ynoic acid)

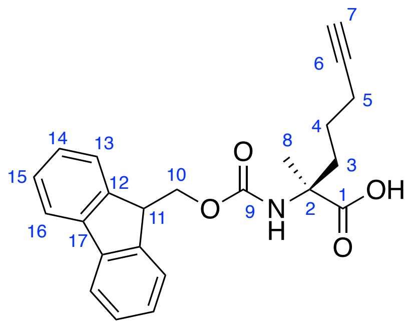

(*S*)-((2-[1-(2-Fluorobenzyl)pyrrolidine-2-carboxamide]phenyl)phenylmethylene)-(*S*)-pentynylalaninato-*N*,*N′*,*N′′*,O)nickel(II) **8** (2.00 g, 3.35 mmol, 1 equiv.) was dissolved in MeOH (40 mL), added dropwise to 3 M $HCl_{(aq.)}$ (20 mL) at 70 °C and left to stir for 30 mins. The solution was cooled to rt and concentrated *in vacuo.* The residue was dissolved in H_2O and DCM and the aqueous layer extracted with DCM (5 × 100 mL) to recover the chiral auxiliary. After concentrating the aqueous layer, 6% $Na_2CO_{3(aq.)}$ (40.0 mL) was added until the pH reached 9-10. EDTA (916 mg, 2.46 mmol, 1 equiv.) was added and left to stir for 15 mins at rt. Afterwards, Fmoc-OSu (1.25 g, 3.35 mmol, 1.1 equiv.) was dissolved in MeCN (40.0 mL) and added dropwise at 0 °C. The reaction mixture was warmed to rt and left to stir for 16 h. MeCN was then removed *in vacuo*, the aqueous layer acidified to pH 1 with 1 M $HCl_{(aq.)}$ and then extracted with EtOAc (3 × 100 mL). The organic layer was washed with brine (3 × 100 mL), dried with $MgSO_4$ and concentrated *in vacuo*. Purification was carried out using normal phase flash column chromatography with a gradient of 0-10% DCM/10% MeOH in DCM yielding **17** as a pale yellow solid (0.750 g, 60%).

^{1}H NMR (400 MHz, MeOD) δ 7.82 (d, *J* = 7.5 Hz, 2H, 16-H), 7.69 (d, *J* = 7.4 Hz, 2H, 13-H), 7.41 (t, *J* = 7.4 Hz, 2H, 15-H), 7.34 (td, *J* = 7.5, 1.2 Hz, 2H, 14-H), 4.37 – 4.36 (m, 2H, 10-H), 4.24 (t, *J* = 6.5 Hz, 1H, 11-H), 2.23 – 2.19 (m, 3H, 5-H + 7-H), 2.02 – 1.98 (m, 2H, 3-H), 1.49 – 1.31 (m, 5H, 4-H + 8-H).

^{13}C NMR (101 MHz, MeOD) δ 177.5 (C=O), 157.1 (C=O), 145.3 (2 × Ar-C), 142.6 (2 × Ar-C), 128.7 (2 × Ar-CH), 128.1 (2 × Ar-CH), 126.2 (2 × Ar-CH), 120.9 (2 × Ar-CH), 84.6 (C), 69.8 (CH), 67.4 (CH_2), 60.1 (C), 48.5 (CH), 37.1 (CH_2), 24.3 (CH_2), 23.3 (CH_3), 19.2 (CH_2).

[α]$_D$^{25} = +2.0 (c 0.1 in MeOH).

IR ($υ_{max}$/cm^{-1}, neat) 3294 (C≡C-H) 2941 (C-H), 1709 (C=O).

HRMS-EI (calcd for $C_{23}H_{23}NO_4$ [M + Na]$^+$) 400.1519, found 400.1513 (Δ = –1.7 ppm).

RP-HPLC (20 min gradient) t_R = 19.4 min, 97% purity.

18 ((*S*)-2-((((9H-fluoren-9-yl)methoxy)carbonyl)amino)-2-methyloct-7-ynoic acid)

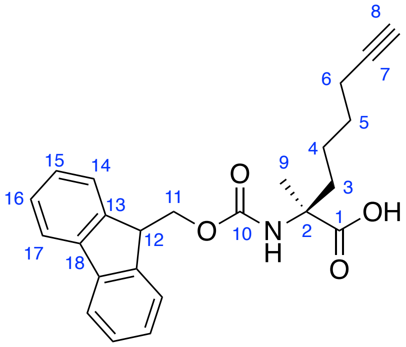

Method as **17** using (*S*)-((2-[1-(2-Fluorobenzyl)pyrrolidine-2-carboxamide]phenyl)phenylmethylene)-(*S*)-hexynylalaninato-*N*,*N′*,*N′′*,O)nickel(II) **9** (1.50 g, 2.46 mmol, 1 equiv.) yielding **18** as a pale yellow solid (0.580 g, 60%).

^{1}H NMR (400 MHz, MeOD) δ 7.79 (d, *J* = 7.5 Hz, 2H, 17-H), 7.66 (d, *J* = 7.3 Hz, 2H, 14-H), 7.38 (t, *J* = 7.4 Hz, 2H, 16-H), 7.31 (td, *J* = 7.4, 1.2 Hz, 2H, 15-H), 4.33 – 4.31 (m, 2H, 11-H), 4.21 (t, *J* = 6.5 Hz, 1H, 12-H), 2.18 – 2.15 (m, 3H, 6-H + 8-H), 1.90 – 1.86 (m, 2H, 3-H), 1.51 – 1.33 (m, 7H, 4-H, 5-H + 9-H).

^{13}C NMR (101 MHz, MeOD) δ 177.7 (C=O), 157.1 (C=O), 145.3 (2 × Ar-C), 142.6 (2 × Ar-C), 128.7 (2 × Ar-CH), 128.1 (2 × Ar-CH), 126.2 (2 × Ar-CH), 120.9 (2 × Ar-CH), 84.8 (C), 69.6 (CH), 67.5 (CH_2), 60.3 (C), 48.5 (CH), 37.3 (CH_2), 29.7 (CH_2), 24.0 (CH_2), 23.3 (CH_3), 18.9 (CH_2).

[α]$_D$^{25} = +5.1 (c 0.1 in MeOH).

IR ($υ_{max}$/cm^{-1}, neat) 3298 (C≡C-H) 2938 (C-H), 2864 (C-H), 1701 (C=O), 1449 (OH).

HRMS-EI (calcd for $C_{24}H_{25}NO_4$ [M + Na]$^+$) 414.1676, found 414.1671 (Δ = –1.3 ppm).

RP-HPLC (20 min gradient) t_R = 20.0 min, 98% purity.

19 ((*S*)-2-((((9*H*-Fluoren-9-yl)methoxy)carbonyl)amino)-2-methylnon-8-ynoic acid)

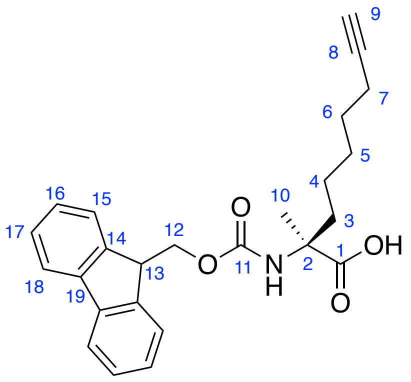

Method as **17** using (*S*)-((2-[1-(2-Fluorobenzyl)pyrrolidine-2-carboxamide]phenyl)phenylmethylene)-(*S*)-heptynylalaninato-*N*,*N′*,*N′′*,O)nickel(II) **10** (4.02 g, 6.44 mmol, 1 equiv.) yielding **19** as a pale yellow solid (1.40 g, 54%).

^{1}H NMR (400 MHz, MeOD) δ 7.77 (d, *J* = 7.5 Hz, 2H, 18-H), 7.65 (d, *J* = 7.5 Hz, 2H, 15-H), 7.37 (t, *J* = 7.5 Hz, 2H, 17-H), 7.30 (td, *J* = 7.4, 1.1 Hz, 2H, 16-H), 4.33 – 4.31 (m, 2H, 12-H), 4.19 (t, *J* = 6.6 Hz, 1H, 13-H), 2.17 – 2.11 (m, 3H, 7-H + 9-H), 1.89 – 1.84 (m, 2H, 3-H), 1.52 – 1.24 (m, 9H, 4-H, 5-H, 6-H, and 10-H).

^{13}C NMR (101 MHz, MeOD) δ 177.7 (C=O), 157.1 (C=O), 145.3 (2 × Ar-C), 142.6 (2 × Ar-C), 128.7 (2 × Ar-CH), 128.1 (2 × Ar-CH), 126.2 (2 × Ar-CH), 120.9 (2 × Ar-CH), 85.0 (C), 69.5 (CH), 67.4 (CH_2), 60.4 (C), 48.5 (CH), 37.8 (CH_2), 29.8 (CH_2), 29.5 (CH_2), 24.4 (CH_2), 23.2 (CH_3), 18.9 (CH_2).

$[\alpha]_D^{25}$ = +3.0 (c 0.1 in MeOH).

IR ($υ_{max}$/cm^{-1}, neat) 3296 (C≡C-H) 2940 (C-H), 2862 (C-H), 1717 (C=O).

HRMS-EI (calcd for $C_{25}H_{27}NO_4$ [M + Na]$^+$) 428.1832, found 428.1827 (Δ = –1.3 ppm).

RP-HPLC (20 min gradient) t_R = 20.8 min, 96% purity.

20 ((*S*)-2-((((9*H*-Fluoren-9-yl)methoxy)carbonyl)amino)-2-methyldec-9-ynoic acid)

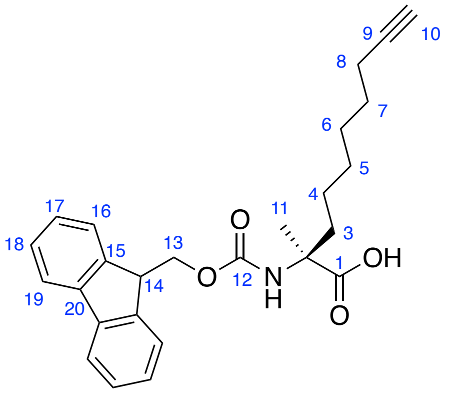

Method as **17** using (*S*)-((2-[1-(2-Fluorobenzyl)pyrrolidine-2-carboxamide]phenyl)phenylmethylene)-(*S*)-octynylalaninato-*N*,*N'*,*N''*,O)nickel(II) **11** (4.10 g, 6.57 mmol, 1 equiv.) yielding **20** as a yellow gum (1.60 g, 58%).

^{1}H NMR (400 MHz, MeOD) δ 7.77 (d, *J* = 7.5 Hz, 2H, 19-H), 7.64 (d, *J* = 7.5 Hz, 2H, 16-H), 7.37 (t, *J* = 7.4 Hz, 2H, 18-H), 7.29 (td, *J* = 7.4, 1.1 Hz, 2H, 17-H), 4.31 – 4.30 (m, 2H, 13-H), 4.19 (t, *J* = 6.6 Hz, 1H, 14-H), 2.14 – 2.10 (m, 3H, 8-H + 10-H), 1.85 – 1.81 (m, 2H, 3-H), 1.45 – 1.28 (m, 11H, 4-H, 5-H, 6-H, 7-H and 11-H).

^{13}C NMR (101 MHz, MeOD) δ 177.7 (C=O), 157.1 (C=O), 145.3 (2 × Ar-C), 142.6 (2 × Ar-C), 128.7 (2 × Ar-CH), 128.1 (2 × Ar-CH), 126.2 (2 × Ar-CH), 120.9 (2 × Ar-CH), 85.0 (C), 69.4 (CH), 67.5 (CH_2), 60.4 (C), 48.5 (CH), 37.9 (CH_2), 30.2 (CH_2), 29.6 (2 × CH_2), 24.8 (CH_2), 23.3 CH_3), 19.0 (CH_2).

$[\alpha]_D^{25}$ = +3.0 (c 0.1 in MeOH).

IR (υ_{max}/cm^{-1}, neat) 3303 (C≡C-H) 2934 (C-H), 2860 (C-H), 1701 (C=O).

HRMS-EI (calcd for $C_{26}H_{29}NO_4$ $[M + Na]^+$) 442.1989, found 442.1976 (Δ = –3.0 ppm).

RP-HPLC (20 min gradient) t_R = 21.5 min, 98% purity.

21 ((*S*)-2-((((9*H*-Fluoren-9-yl)methoxy)carbonyl)amino)-non-8-ynoic acid)

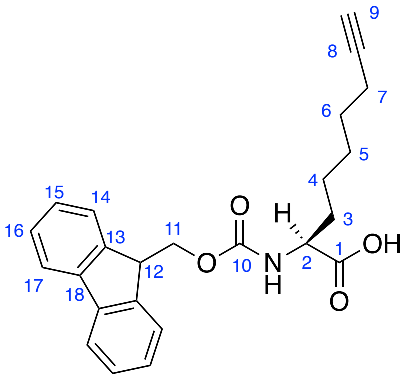

Method as **17** using (*S*)-((2-[1-(2-Fluorobenzyl)pyrrolidine-2-carboxamide]phenyl)phenylmethylene)-(*S*)-heptynylglycinato-*N*,*N'*,*N''*,O)nickel(II) **12** (2.89 g, 4.73 mmol, 1 equiv.) yielding **21** as a yellow gum (1.11 g, 60%).

^{1}H NMR (400 MHz, MeOD) δ 7.82 (d, *J* = 7.6 Hz, 2H, 17-H), 7.70 (t, *J* = 7.0 Hz, 2H, 14-H), 7.41 (t, *J* = 7.4 Hz, 2H, 16-H), 7.33 (t, *J* = 7.5 Hz, 2H, 15-H), 4.39 (d, *J* = 6.9 Hz, 2H, 11-H), 4.26 (t, *J* = 6.9 Hz, 1H, 12-H), 4.19 – 4.15 (d, 1H, 2-H), 2.21 – 2.18 (m, 3H, 7-H + 9-H), 1.92 – 1.82 (m, 1H, 3-H), 1.75 – 1.68 (m, 1H, 3'-H), 1.59 – 1.32 (m, 6H, 4-H, 5-H + 6-H).

^{13}C NMR (101 MHz, MeOD) δ 176.0 (C=O), 158.6 (C=O), 145.3 (Ar-C), 145.1 (Ar-C), 142.5 (2 $\times$ Ar-C), 128.7 (2 $\times$ Ar-CH), 128.1 (2 $\times$ Ar-CH), 126.2 (2 $\times$ Ar-CH), 120.9 (2 $\times$ Ar-CH), 85.0 (C), 69.5 (CH), 67.9 (13-CH_2), 55.2 (C), 48.4 (CH), 32.6 (CH_2), 29.4 (CH_2), 29.2 (CH_2), 26.4 (CH_2), 18.9 (CH_3).

$[\alpha]_D^{25}$ = +6.0 (c 0.1 in MeOH).

IR (υ_{max}/cm^{-1}, neat) 3293 (C≡C-H), 2941 (C-H), 2864 (C-H), 1701 (C=O).

HRMS-EI (calcd for $C_{24}H_{25}NO_4$ $[M + Na]^+$) 414.1676, found 414.1666 (Δ = –2.3 ppm).

RP-HPLC (20 min gradient) t_R = 20.4 min, 96% purity.

22 ((*R*)-2-((((9*H*-fluoren-9-yl)methoxy)carbonyl)amino)-2-methylhept-6-ynoic acid)

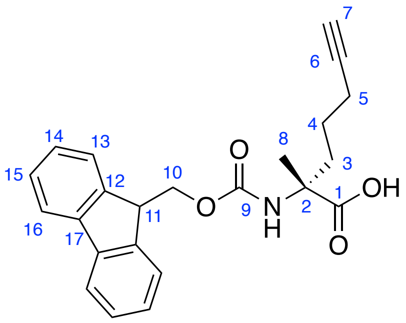

Method as **17** using (*R*)-((2-[1-(2-Fluorobenzyl)pyrrolidine-2-carboxamide]phenyl)phenylmethylene)-(*R*)-pentynylalaninato-*N*,*N'*,*N''*,O)nickel(II) **13** (0.460 g, 0.810 mmol, 1 equiv.) yielding **100d** as a pale yellow solid (190 mg, 62%).

^{1}H NMR (400 MHz, MeOD) δ 7.82 (d, *J* = 7.5 Hz, 2H, 16-H), 7.69 (d, *J* = 7.4 Hz, 2H, 13-H), 7.41 (t, *J* = 7.4 Hz, 2H, 15-H), 7.34 (td, *J* = 7.4, 1.2 Hz, 2H, 14-H), 4.37 – 4.36 (m, 2H, 10-H), 4.24 (t, *J* = 6.5 Hz, 1H, 11-H), 2.24 – 2.18 (m, 3H, 5-H + 7-H), 2.02 – 1.98 (m, 2H, 3-H), 1.49 – 1.31 (m, 5H, 4-H + 8-H).

^{13}C NMR (101 MHz, MeOD) δ 177.5 (C=O), 157.0 (C=O), 145.2 (2 × Ar-C), 142.5 (2 × Ar-C), 128.7 (2 × Ar-CH), 128.1 (2 × Ar-CH), 126.2 (2 × Ar-CH), 120.9 (2 × Ar-CH), 84.6 (C), 69.9 (CH), 67.4 (CH_2), 60.1 (C), 48.4 (CH), 37.1 (CH_2), 24.3 (CH_2), 23.3 (CH_3), 19.2 (CH_2).

$[\alpha]_D^{25}$ = –2.0 (c 0.1 in MeOH).

IR (υ_{max}/cm^{-1}, neat) 3292 (C≡C-H), 2936 (C-H), 1701 (C=O).

HRMS-EI (calcd for $C_{23}H_{23}NO_4$ [M + Na]$^+$) 400.151, found 400.1510 (Δ = –2.3 ppm).

RP-HPLC (20 min gradient) t_R = 19.4 min, 96% purity.

23 ((*R*)-2-((((9H-fluoren-9-yl)methoxy)carbonyl)amino)-2-methyloct-7-ynoic acid)

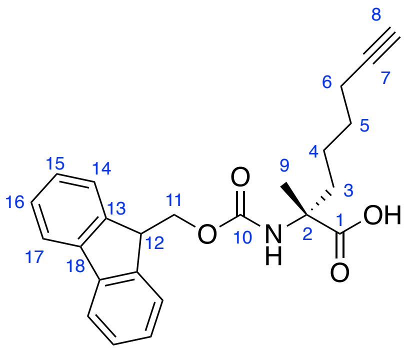

Method as **17** using (*R*)-((2-[1-(2-Fluorobenzyl)pyrrolidine-2-carboxamide]phenyl)phenylmethylene)-(*R*)-hexynylalaninato-*N*,*N'*,*N''*,O)nickel(II) **14** (458 mg, 0.750 mmol, 1 equiv.) yielding **23** as a pale yellow solid (179 mg, 61%).

^{1}H NMR (500 MHz, MeOD) δ 7.80 (d, *J* = 7.5 Hz, 2H, 17-H), 7.67 (d, *J* = 7.3 Hz, 2H, 14-H), 7.40 (t, *J* = 7.5 Hz, 2H, 16-H), 7.32 (t, *J* = 7.3 Hz, 2H, 15-H), 4.34 – 4.33 (m, 2H, 11-H), 4.22 (t, *J* = 6.5 Hz, 1H, 12-H), 2.17 – 2.16 (m, 3H, 6-H + 8-H), 1.89-1.88 (m, 2H, 3-H), 1.48 – 1.30 (m, 7H, 4-H, 5-H + 9-H).

^{13}C NMR (101 MHz, MeOD) δ 177.6 (C=O), 157.0 (C=O), 145.3 (2 × Ar-C), 142.5 (2 × Ar-C), 128.7 (2 × Ar-CH), 128.1 (2 × Ar-CH), 126.2 (2 × Ar-CH), 120.9 (2 × Ar-CH), 84.8 (C), 69.6 (CH), 67.4 (CH_2), 60.3 (C), 48.4 (CH), 37.3 (CH_2), 29.7 (CH_2), 24.0 (CH_2), 23.3 (CH_3), 18.9 (CH_2).

$[\alpha]_D^{25}$ = –5.4 (c 0.1 in MeOH).

IR (υ_{max}/cm^{-1}, neat) 3293 (C≡C-H) 2940 (C-H), 2864 (C-H), 1705 (C=O).

HRMS-EI (calcd for $C_{24}H_{25}NO_4$ [M + Na]$^+$) 414.1676, found 414.1670 (Δ = –1.4 ppm).

RP-HPLC (20 min gradient) t_R = 20.0 min, 99% purity.

24 ((*R*)-2-((((9*H*-Fluoren-9-yl)methoxy)carbonyl)amino)-2-methylnon-8-ynoic acid)

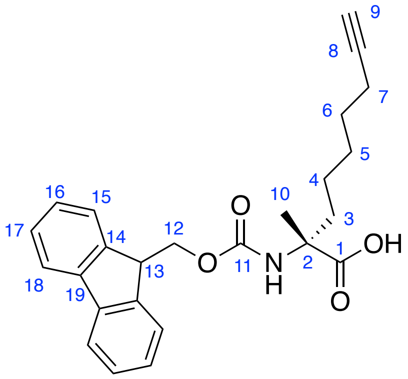

Method as **17** using (*R*)-((2-[1-(2-Fluorobenzyl)pyrrolidine-2-carboxamide]phenyl)phenylmethylene)-(*R*)-heptynylalaninato-*N*,*N'*,*N''*,O)nickel(II) **15** (4.06 g, 6.50 mmol, 1 equiv.) yielding **24** as a pale yellow solid (2.00 g, 77%).

^{1}H NMR (400 MHz, MeOD) δ 7.77 (d, *J* = 7.5 Hz, 2H, 18-H), 7.64 (d, *J* = 7.5 Hz, 2H, 15-H), 7.37 (t, *J* = 7.4 Hz, 2H, 17-H), 7.29 (td, *J* = 7.5, 1.1 Hz, 2H, 16-H), 4.32 – 4.31 (m, 2H, 12-H), 4.19 (t, *J* = 6.6 Hz, 1H, 13-H), 2.16 – 2.11 (m, 3H, 7-H + 9-H), 1.87 – 1.85 (m, 2H, 3-H), 1.50 – 1.24 (m, 9H, 4-H, 5-H, 6-H, and 10-H).

^{13}C NMR (101 MHz, MeOD) δ 177.8 (C=O), 157.1 (C=O), 145.3 (2 × Ar-C), 142.6 (2 × Ar-C), 128.7 (2 × Ar-CH), 128.1 (2 × Ar-CH), 126.2 (2 × Ar-CH), 120.9 (2 × Ar-CH), 85.0 (C), 69.5 (CH), 67.4 (CH_2), 60.4 (C), 48.5 (CH), 37.8 (CH_2), 29.8 (CH_2), 29.5 (CH_2), 24.4 (CH_2), 23.3 (CH_3), 18.9 (CH_2).

$[\alpha]_D^{25}$ = –3.3 (c 0.1 in MeOH).

IR (υ_{max}/cm^{-1}, neat) 3304 (C≡C-H) 2934 (C-H), 2855 (C-H), 1719 (C=O).

HRMS-EI (calcd for $C_{25}H_{27}NO_4$ $[M + Na]^+$) 428.1832, found 428.1820 (Δ = –2.8 ppm).

RP-HPLC (20 min gradient) t_R = 20.8 min, 97% purity.

25 ((*R*)-2-((((9*H*-Fluoren-9-yl)methoxy)carbonyl)amino)-2-methyldec-9-ynoic acid)

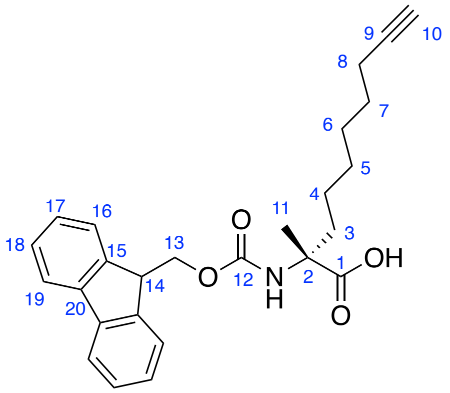

Method as **17** using (*R*)-((2-[1-(2-Fluorobenzyl)pyrrolidine-2-carboxamide]phenyl)phenylmethylene)-(*R*)-octynylalaninato-*N*,*N′*,*N′′*,O)nickel(II) **16** (2.66 g, 4.16 mmol, 1 equiv.) yielding **25** as a yellow gum (1.20 g, 69%).

^{1}H NMR (400 MHz, MeOD) δ 7.78 (d, *J* = 7.5 Hz, 2H, 19-H), 7.65 (d, *J* = 7.5 Hz, 2H, 16-H), 7.38 (t, *J* = 7.4 Hz, 2H, 18-H), 7.30 (td, *J* = 7.5, 1.2 Hz, 2H, 17-H), 4.33 – 4.31 (m, 2H, 13-H), 4.20 (t, *J* = 6.6 Hz, 1H, 14-H), 2.15 – 2.12 (m, 3H, 8-H + 10-H), 1.85 – 1.81 (m, 2H, 3-H), 1.46 – 1.29 (m, 11H, 4-H, 5-H, 6-H, 7-H and 11-H).

^{13}C NMR (101 MHz, MeOD) δ 177.7 (C=O), 157.1 (C=O), 145.3 (2 × Ar-C), 142.6 (2 × Ar-C), 128.7 (2 × Ar-CH), 128.1 (2 × Ar-CH), 126.2 (2 × Ar-CH), 120.9 (2 × Ar-CH), 85.0 (C), 69.5 (CH), 67.4 (CH_2), 60.4 (C), 48.5 (CH), 37.9 (CH_2), 30.2 (CH_2), 29.6 (2 × CH_2), 24.4 (CH_2), 23.3 (CH_3), 19.0 (CH_2).

$[\alpha]_D^{25}$ = –3.1 (c 0.1 in MeOH).

IR (υ_{max}/cm^{-1}, neat) 3305 (C≡C-H) 2934 (C-H), 2860 (C-H) 1705 (C=O).

HRMS-EI (calcd for $C_{25}H_{27}NO_4$ [M + Na]$^+$) 442.1989, found 442.1973 (Δ = –3.5 ppm).

RP-HPLC (20 min gradient) t_R = 21.4 min, 98% purity.

NMR Data

S1 ((2-Fluorobenzyl)-L-proline hydrochloride)

^{1}H NMR

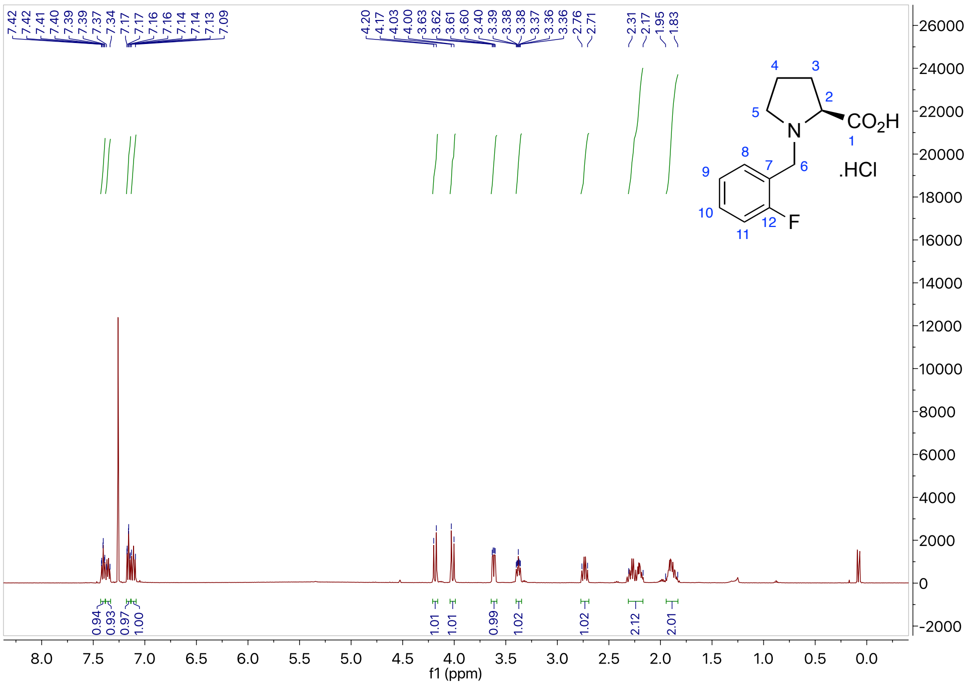

^{13}C NMR

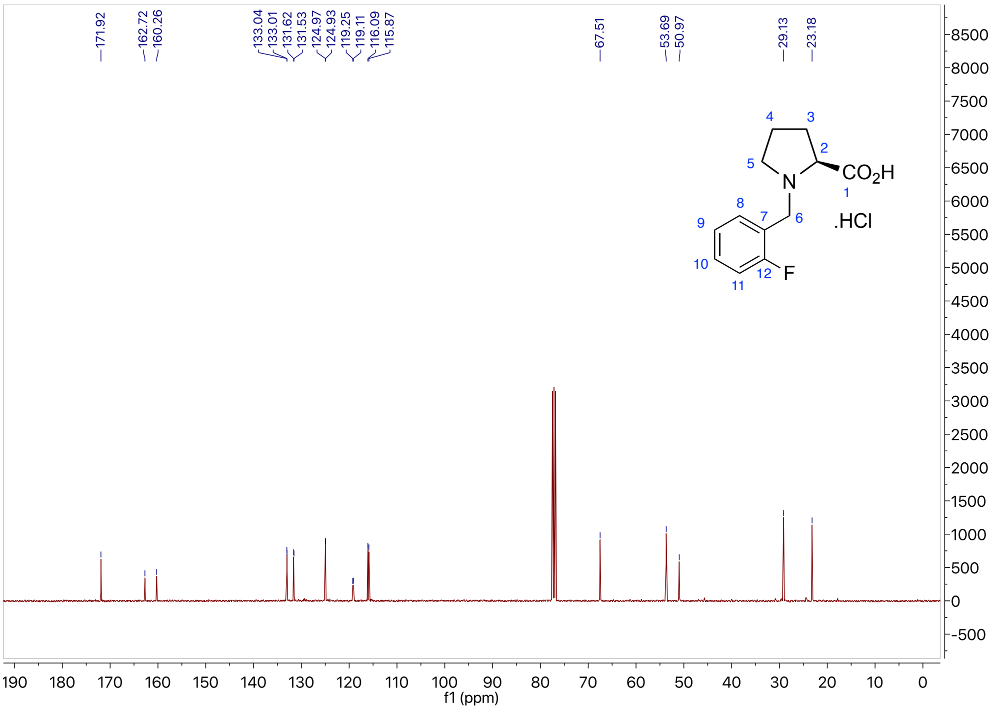

^{19}F NMR

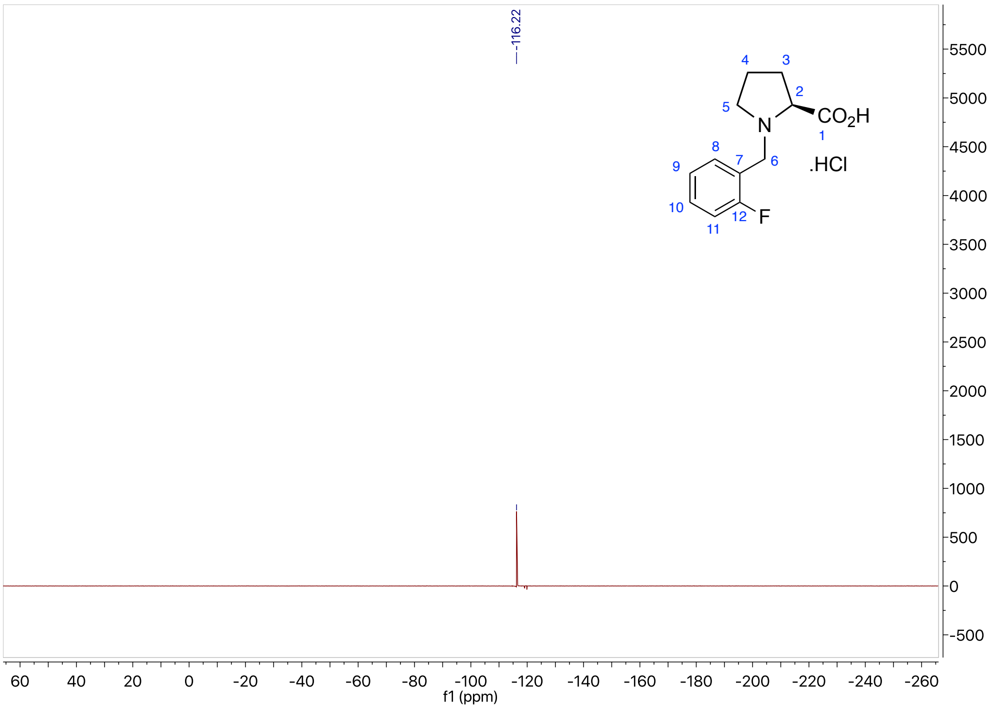

S2 ((2-Fluorobenzyl)-D-proline hydrochloride)

^{1}H NMR

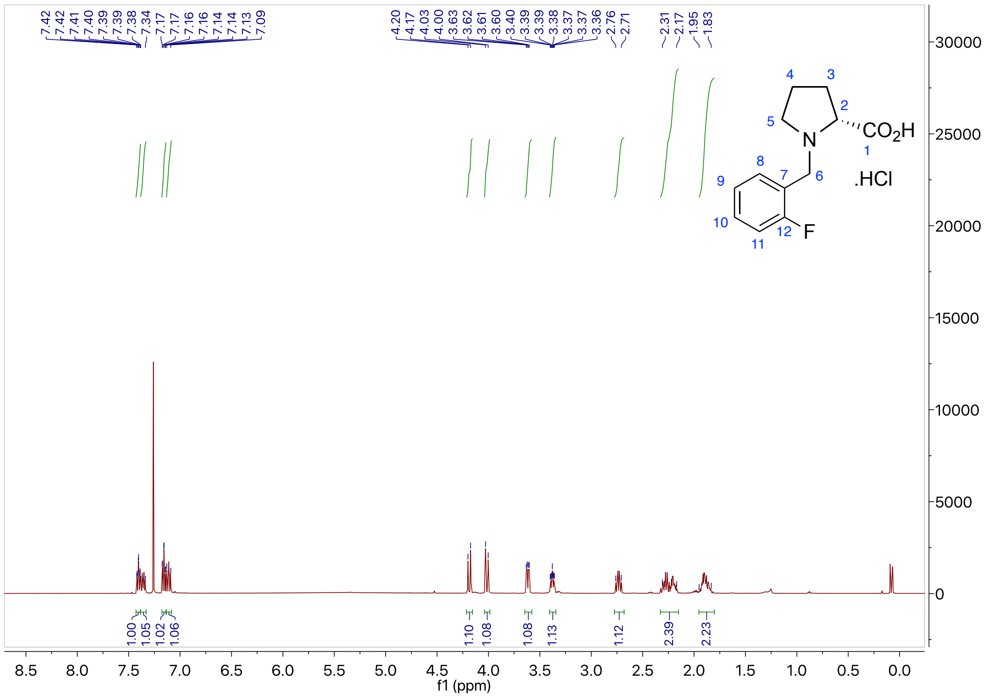

^{13}C NMR

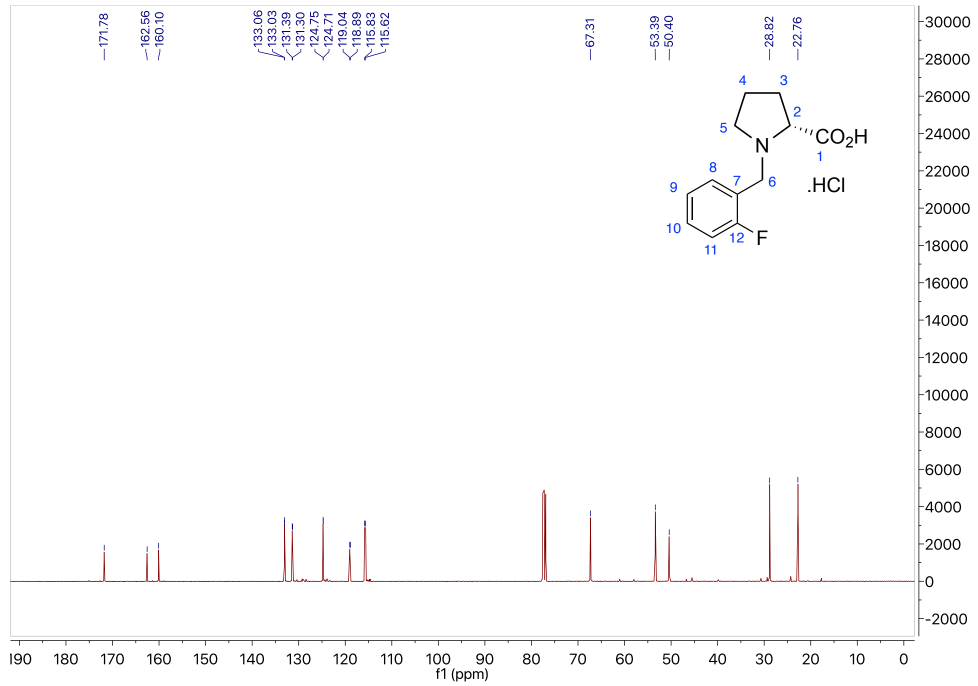

^{19}F NMR

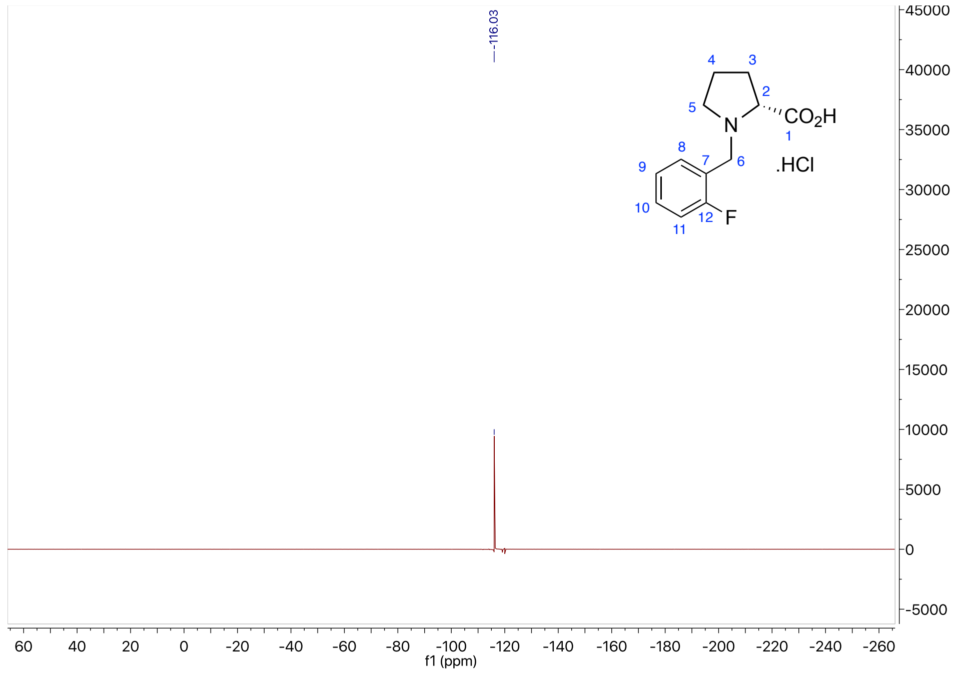

S3 ((*S*)-*N*-(2-Benzoylphenyl)-1-(2-fluorobenzyl)pyrrolidine-2-carboxamide)

^{1}H NMR

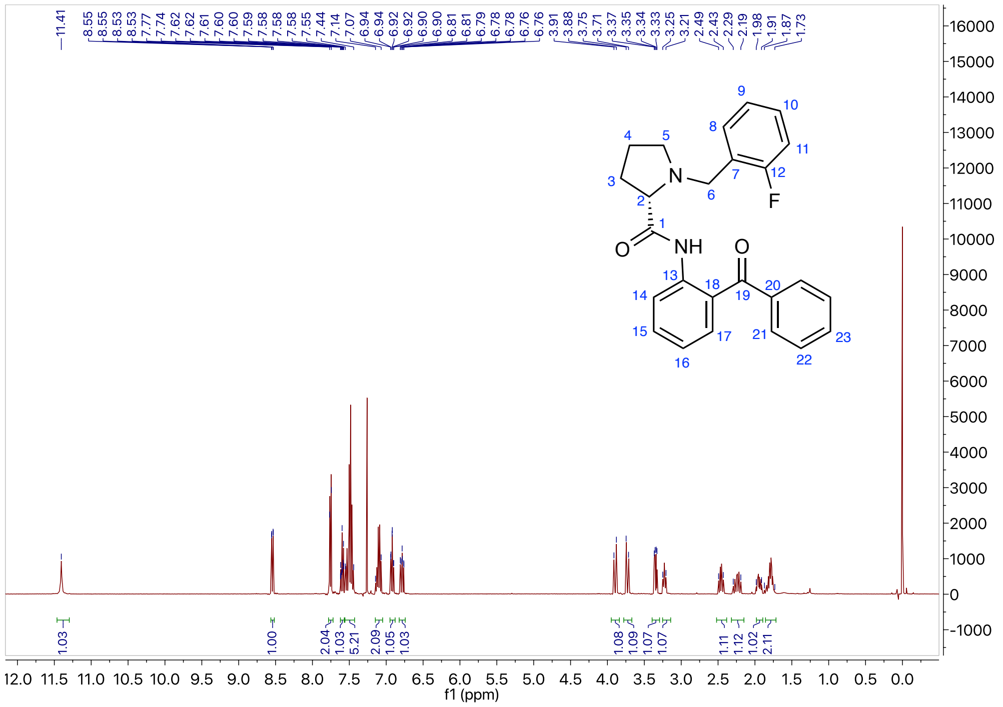

^{13}C NMR

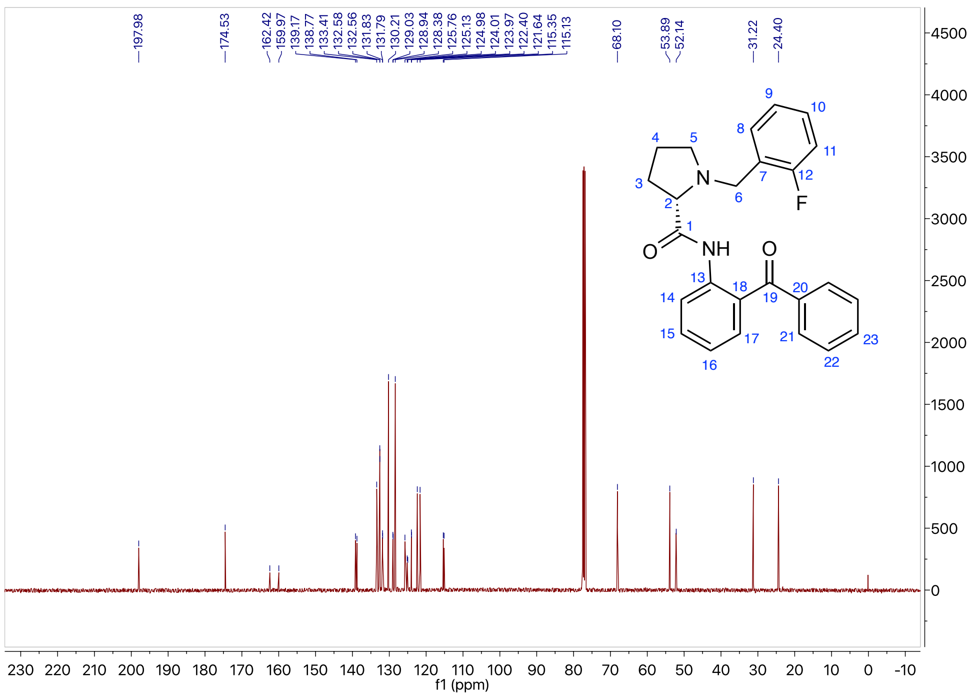

^{19}F NMR

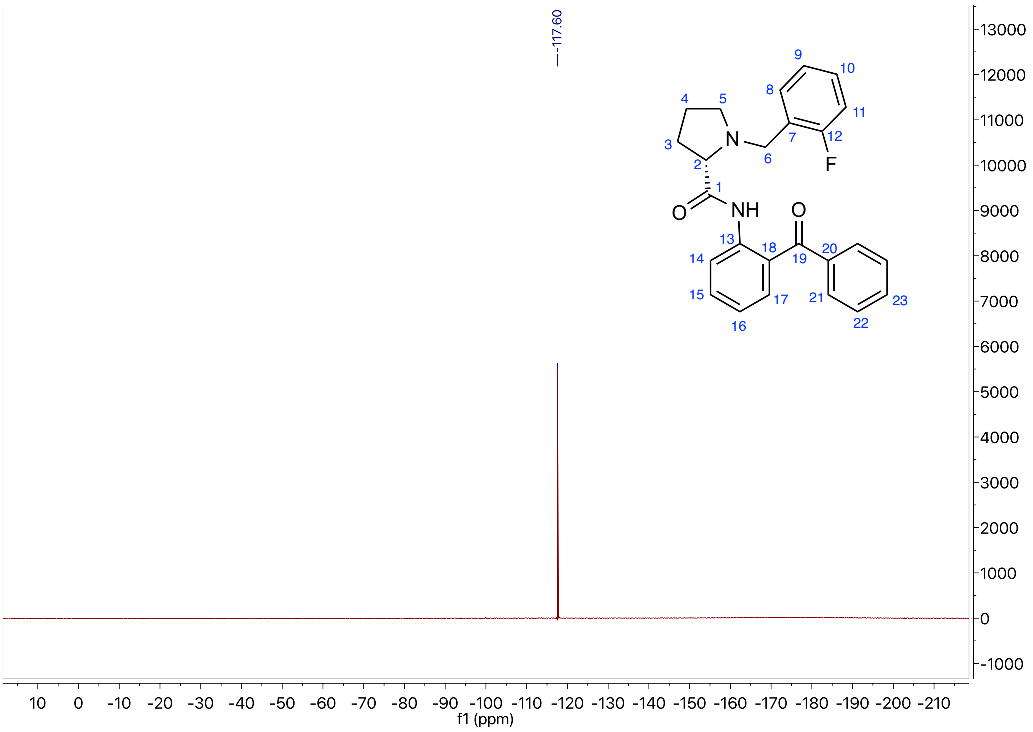
-117.60
13000
12000
11000
10000
9000
8000
7000
6000
5000
4000
3000
2000
1000
0
-1000
10 0 -10 -20 -30 -40 -50 -60 -70 -80 -90 -100 -110 -120 -130 -140 -150 -160 -170 -180 -190 -200 -210
f1 (ppm)
N
F
O
NH
O

S4 ((*R*)-*N*-(2-Benzoylphenyl)-1-(2-fluorobenzyl)pyrrolidine-2-carboxamide)

^{1}H NMR

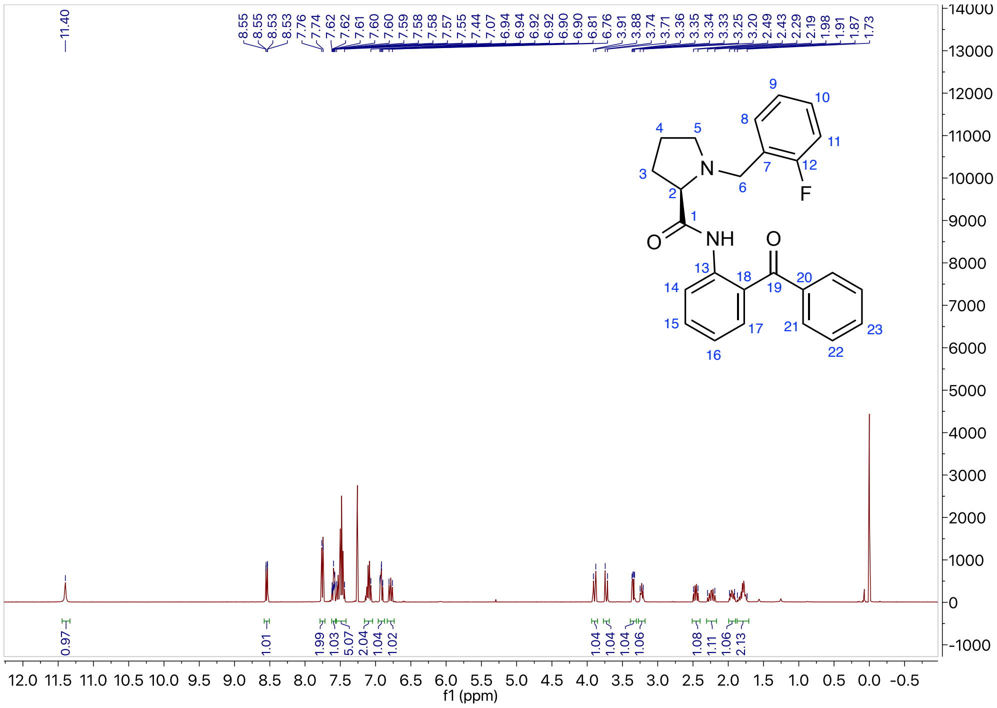

^{13}C NMR

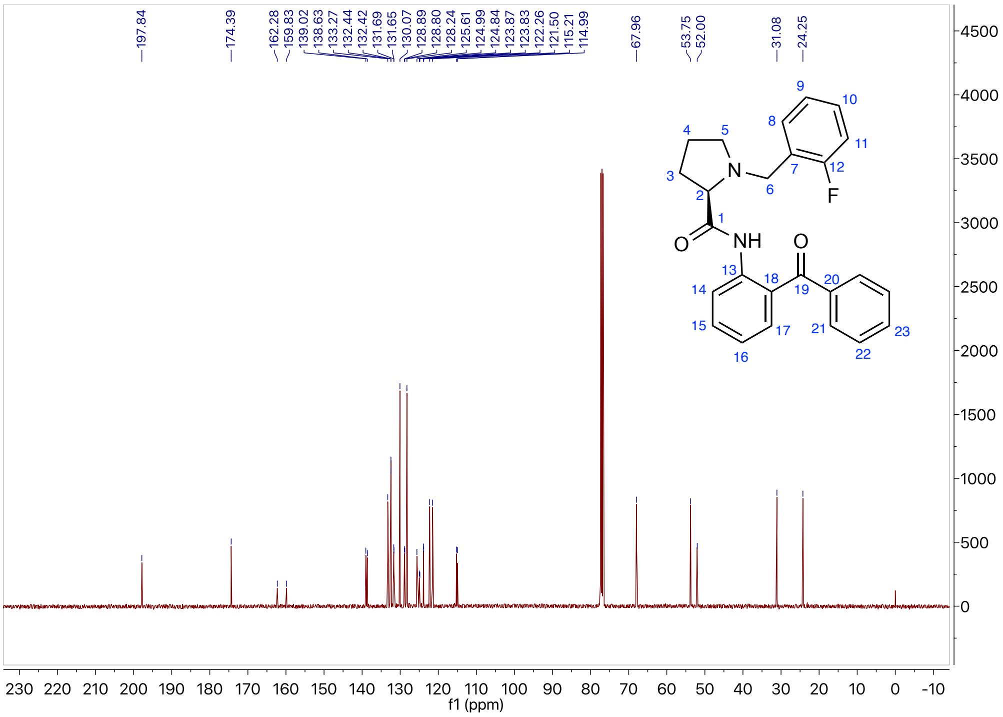

^{19}F NMR

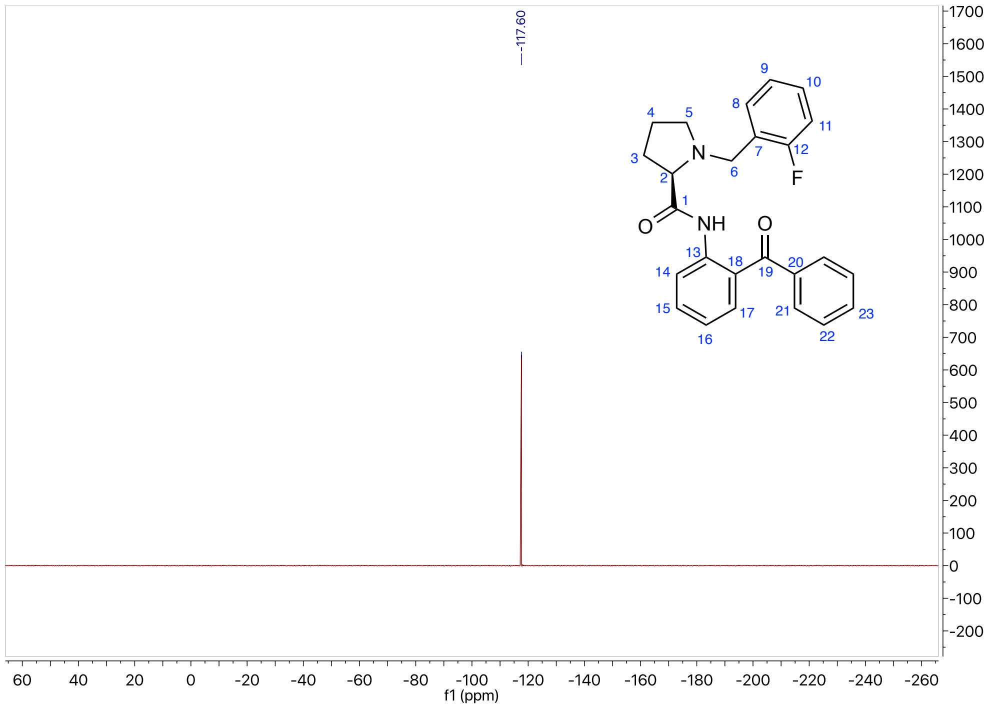

S5 (Oct-7-yn-1-ol)

^{1}H NMR

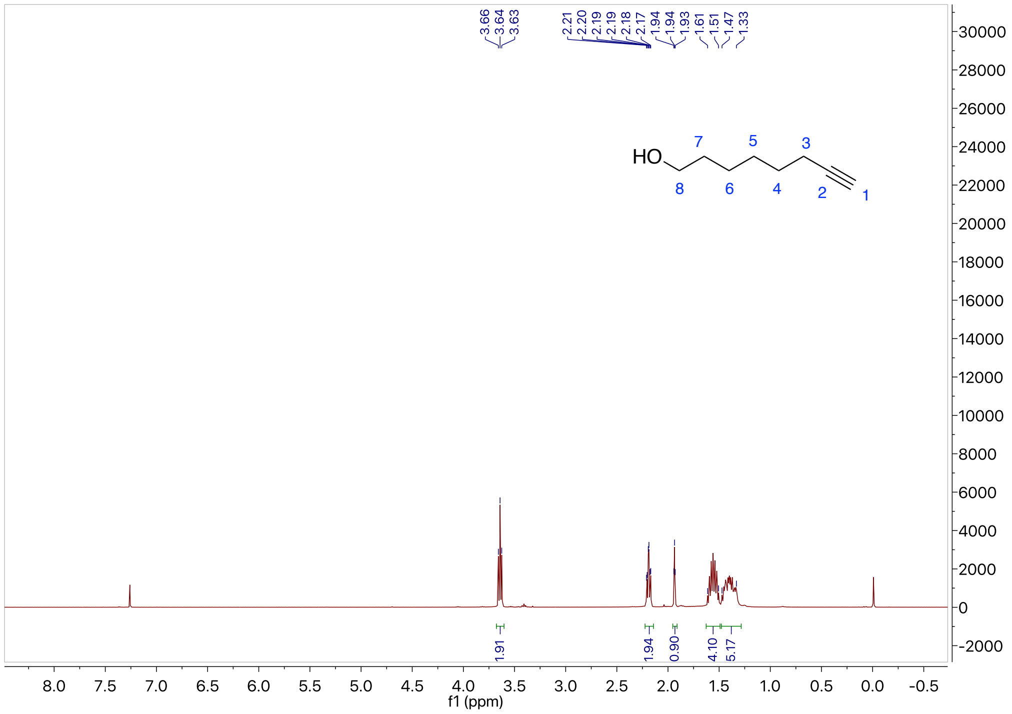

^{13}C NMR

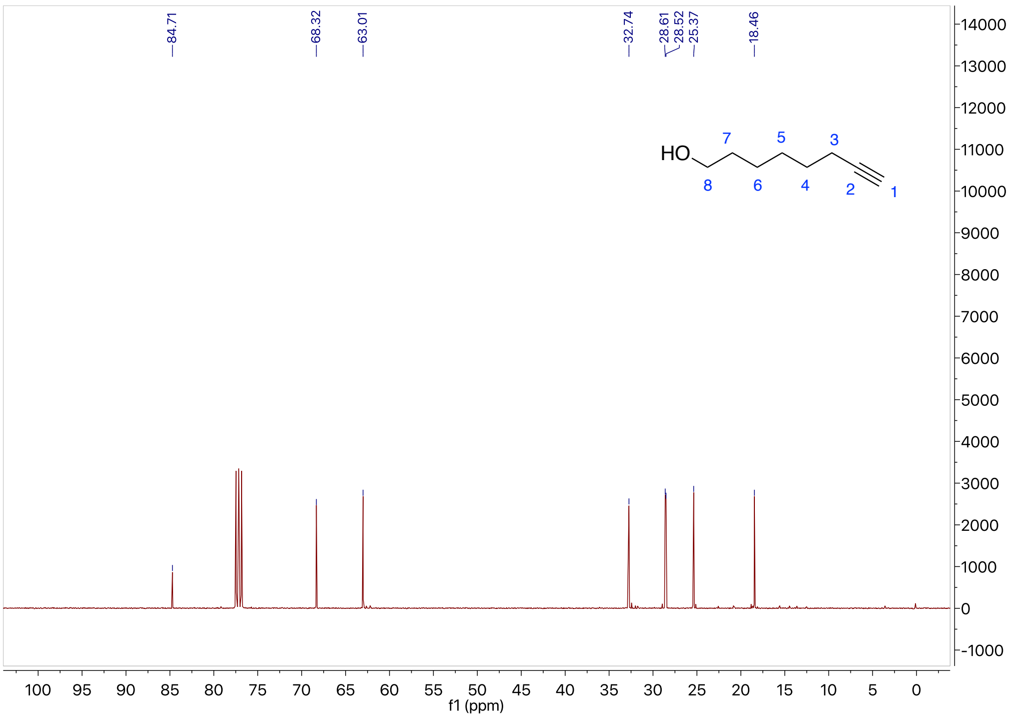
84.71
68.32
63.01
32.74
28.61
28.52
25.37
18.46
HO
7
5
3
8
6
4
2
1
14000
13000
12000
11000
10000
9000
8000
7000
6000
5000
4000
3000
2000
1000
0
-1000
100 95 90 85 80 75 70 65 60 55 50 45 40 35 30 25 20 15 10 5 0
f1 (ppm)

1 ((*S*)-((2-[1-(2-Fluorobenzyl)pyrrolidine-2-carboxamide]phenyl)phenylmethylene)-(*S*)-alaninato-*N*,*N'*,*N''*,O)nickel(II))

^{1}H NMR

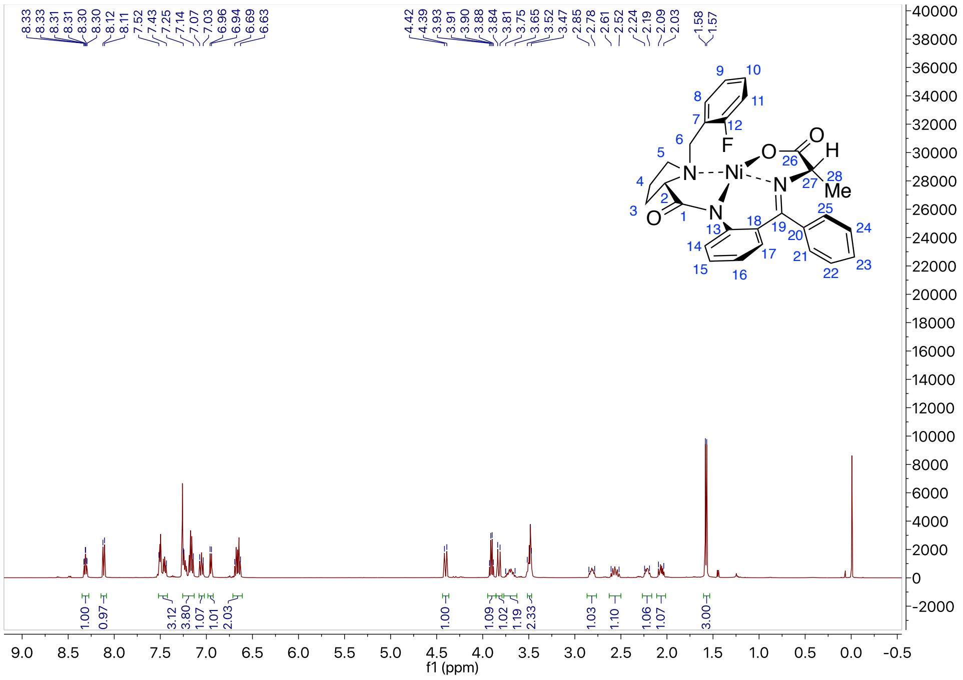

^{13}C NMR

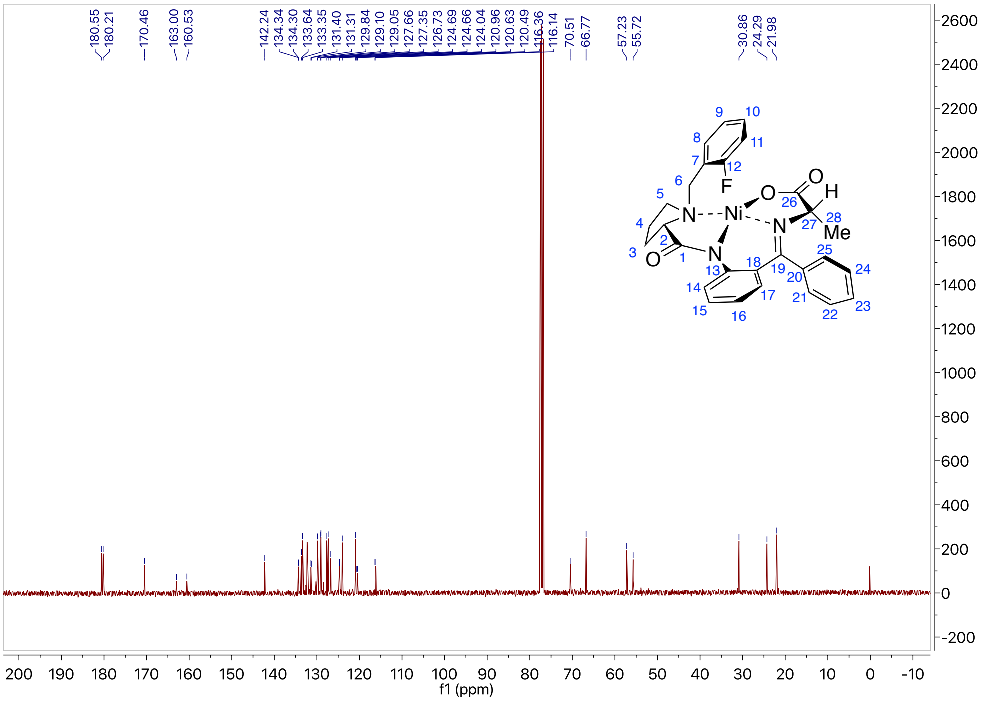

^{19}F NMR

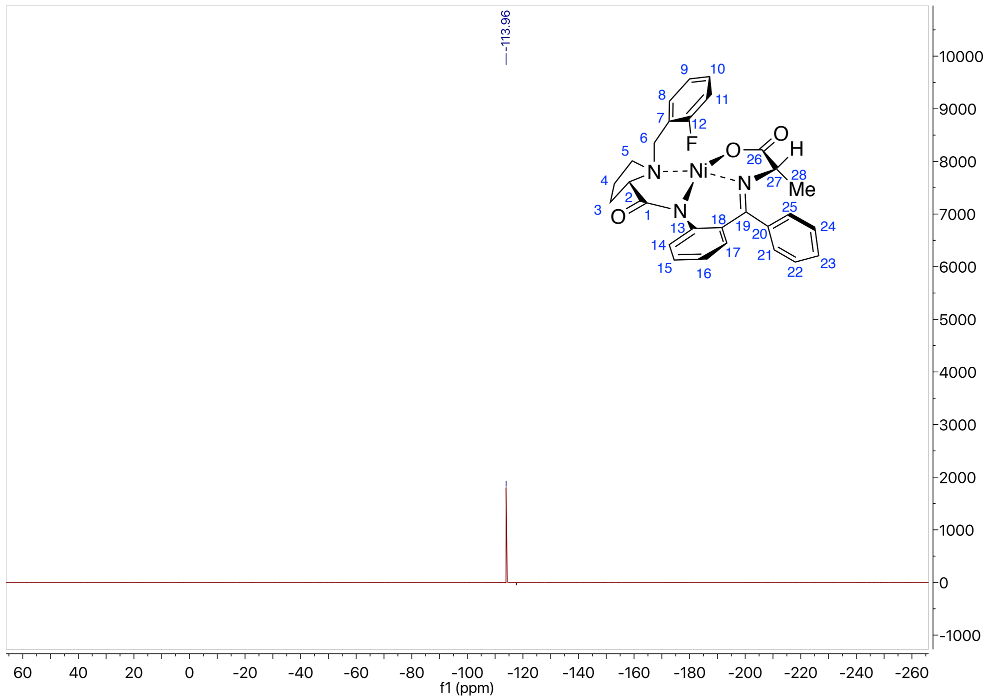

2 ((*R*)-((2-[1-(2-Fluorobenzyl)pyrrolidine-2-carboxamide]phenyl)phenylmethylene)-(*R*)-alaninato-*N*,*N'*,*N''*,O)nickel(II))

^{1}H NMR

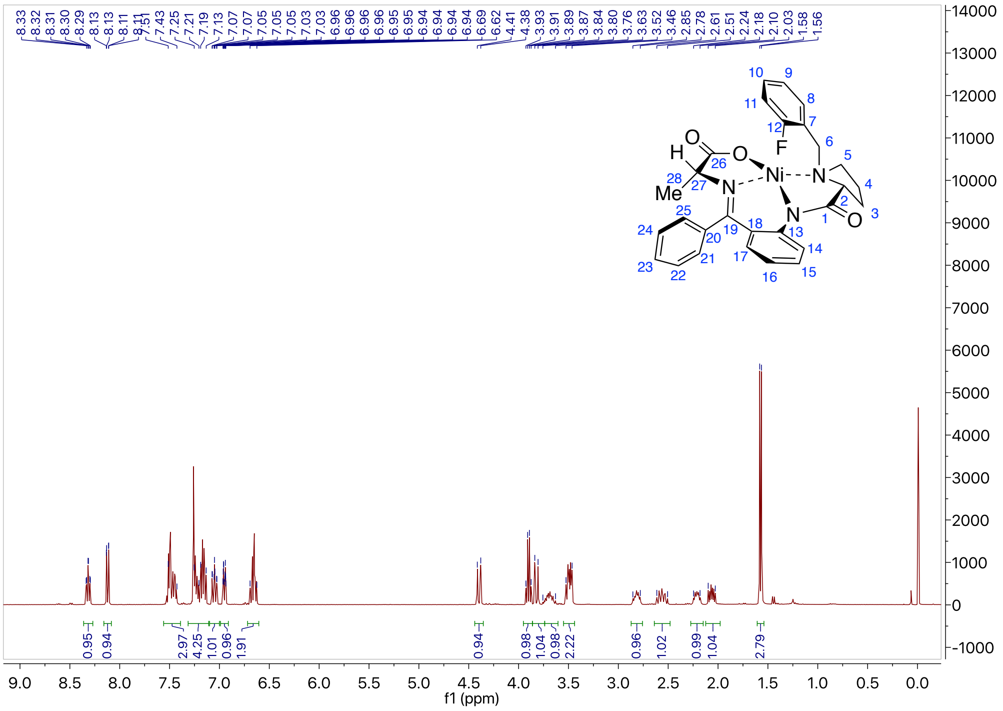

^{13}C NMR

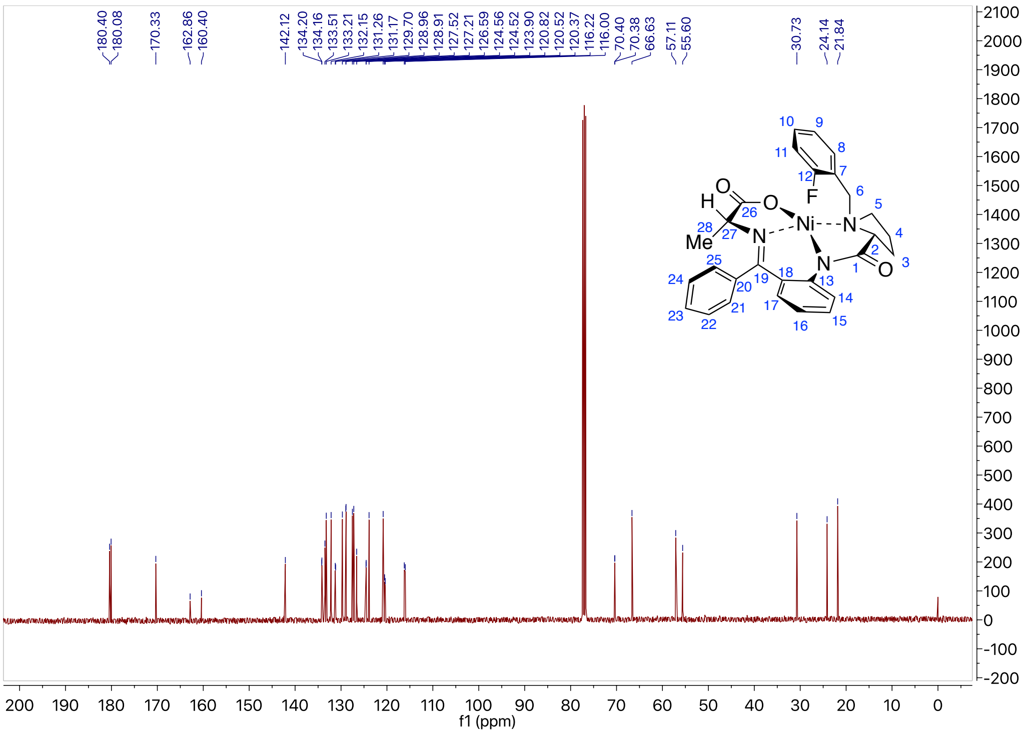

^{19}F NMR

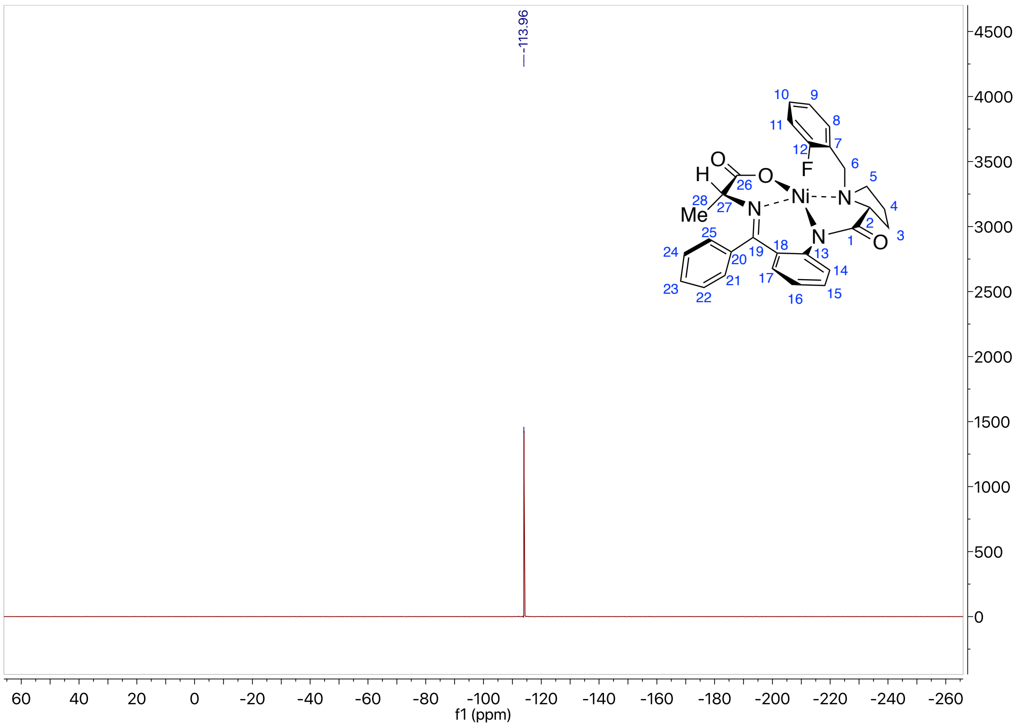

3 ((*S*)-((2-[1-(2-Fluorobenzyl)pyrrolidine-2-carboxamide]phenyl)phenylmethylene)-glycinato-*N*,*N'*,*N''*, O)nickel(II))

^{1}H NMR

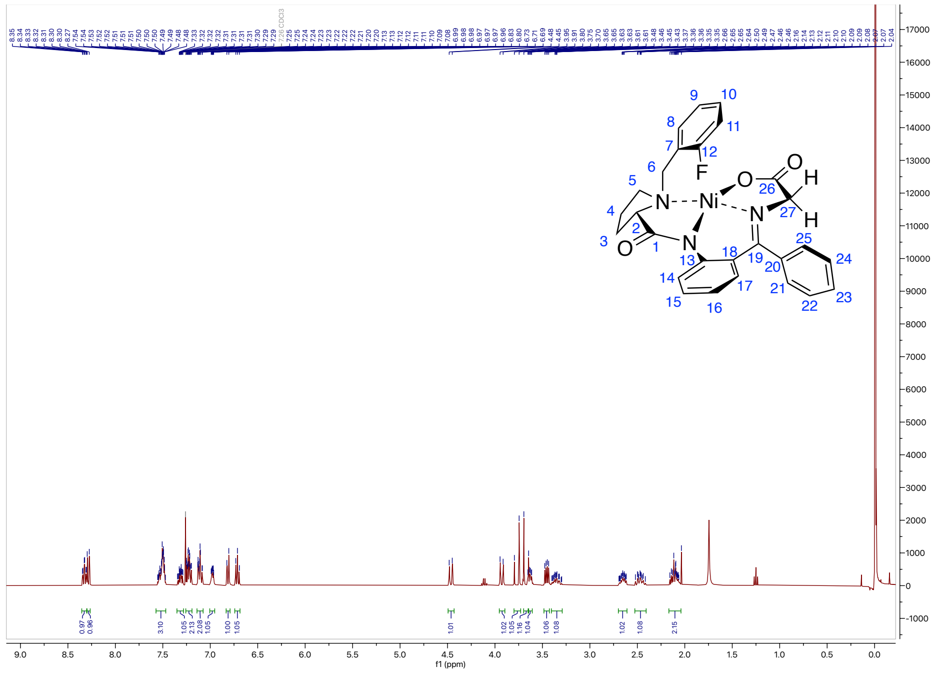

^{13}C NMR

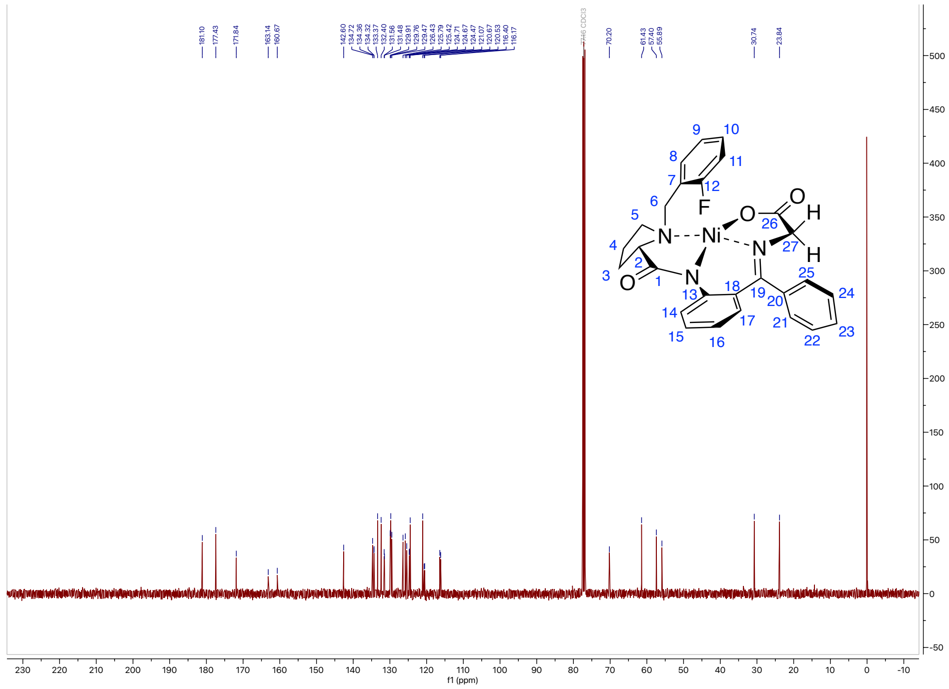

^{19}F NMR

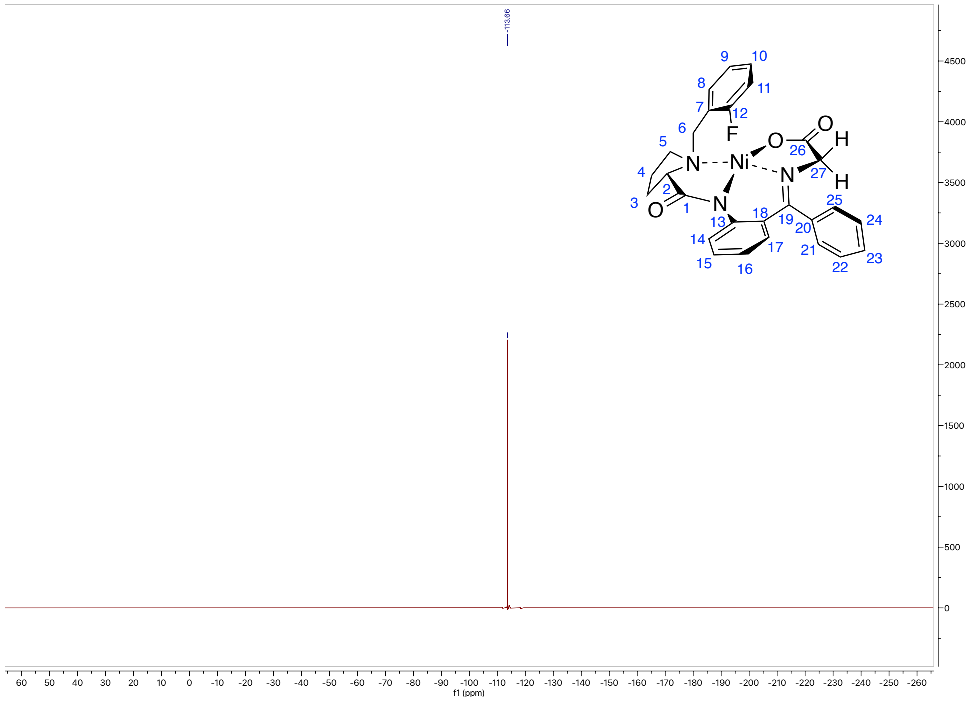

4 (5-Iodopent-1-yne)

^{1}H NMR

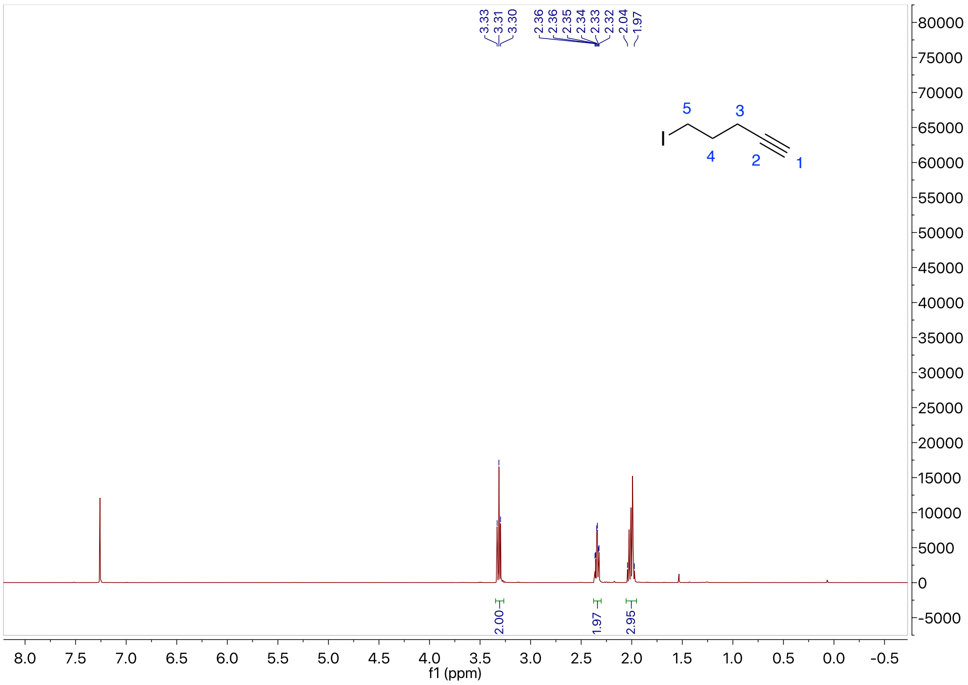

^{13}C NMR

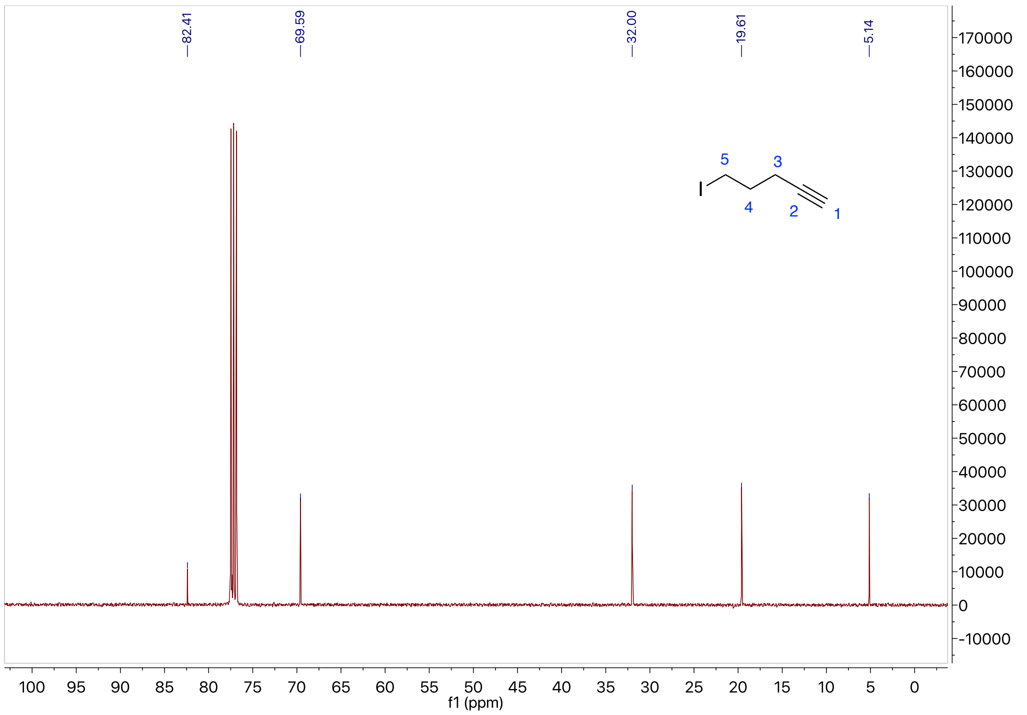

5 (6-Iodohex-1-yne)

^{1}H NMR

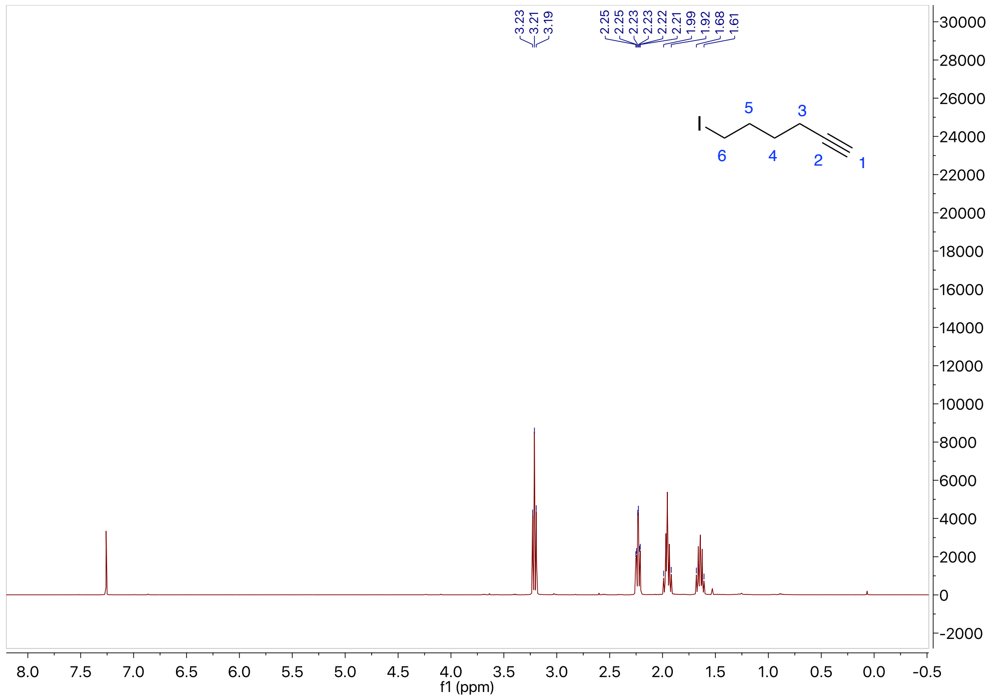

^{13}C NMR

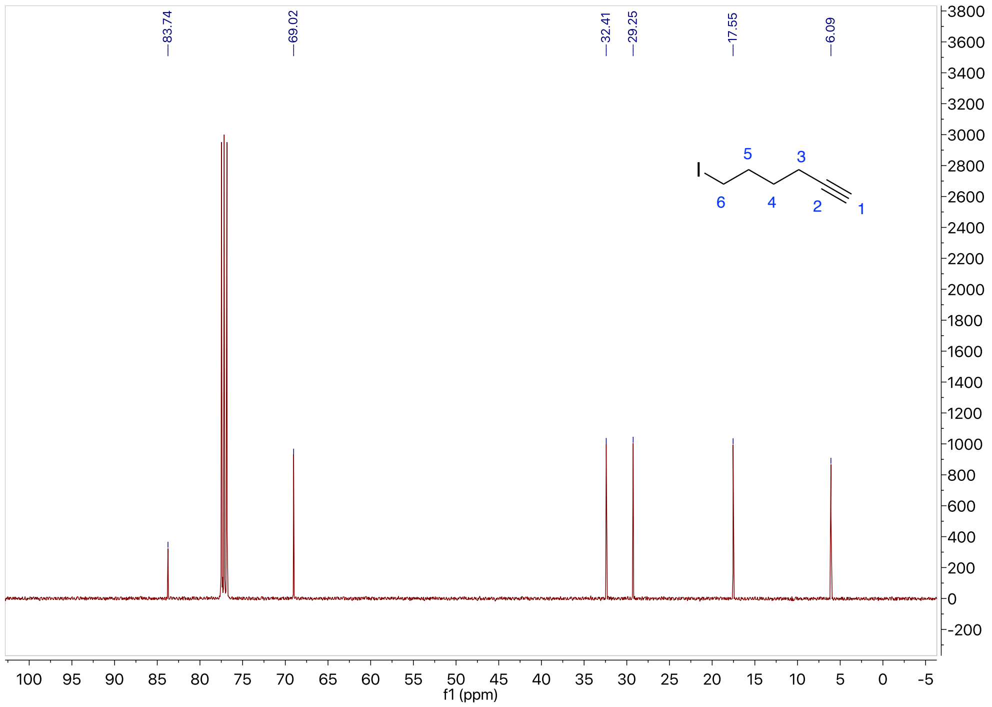

6 (7-Iodohept-1-yne)

^{1}H NMR

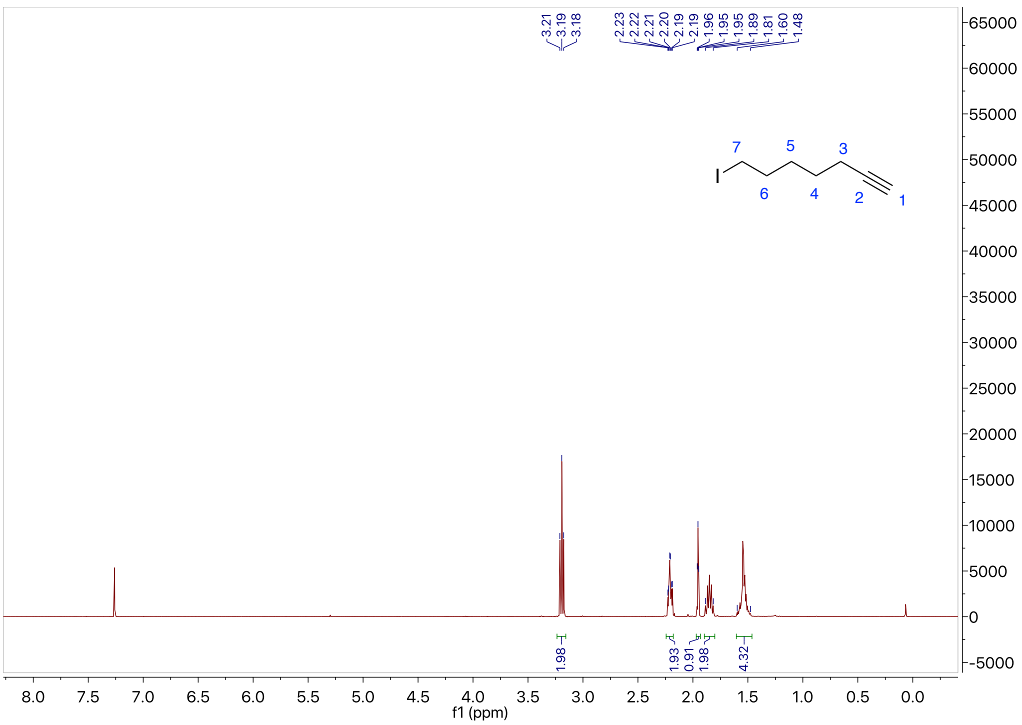

^{13}C NMR

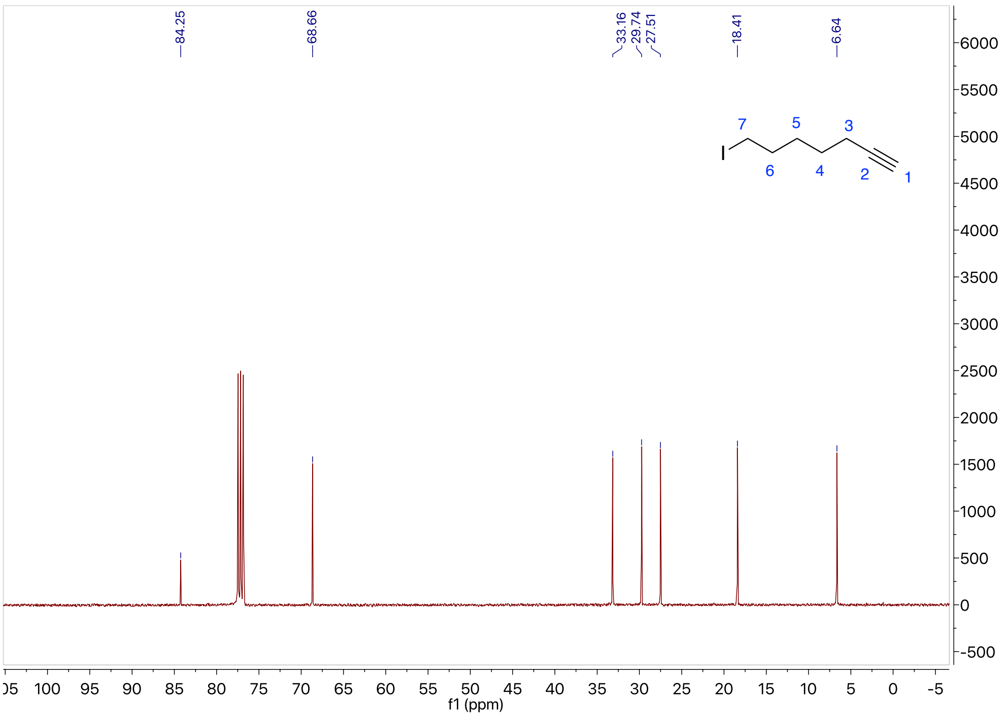

7 (8-Iodooct-1-yne)

^{1}H NMR

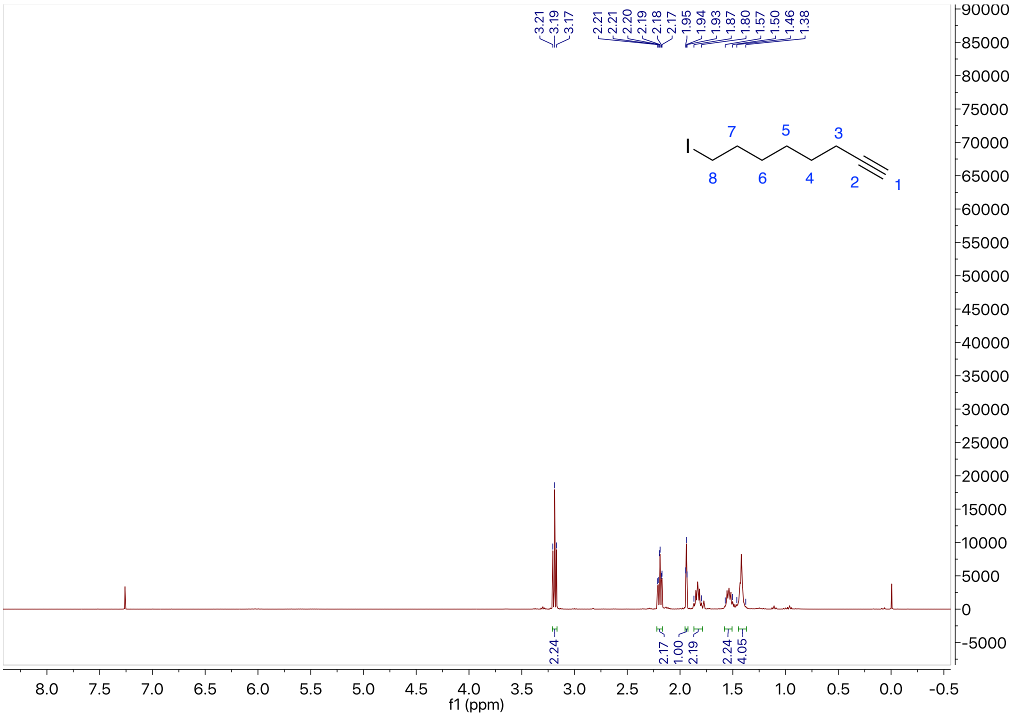

^{13}C NMR

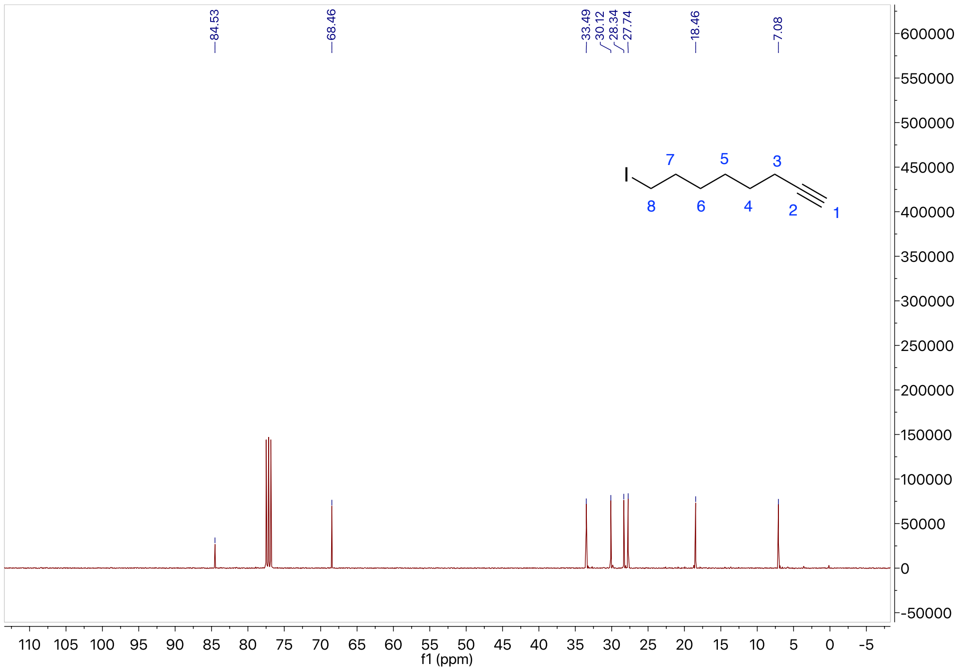
84.53
68.46
33.49
30.12
28.34
27.74
18.46
7.08
I
7
5
3
8
6
4
2
1
600000
550000
500000
450000
400000
350000
300000
250000
200000
150000
100000
50000
0
-50000
110 105 100 95 90 85 80 75 70 65 60 55 50 45 40 35 30 25 20 15 10 5 0 -5
f1 (ppm)

8 (C5 *S* Alkylated Complex - (*S*)-((2-[1-(2-Fluorobenzyl)pyrrolidine-2-carboxamide]phenyl)phenylmethylene)-(*S*)-pentynylalaninato-*N*,*N'*,*N''*,O)nickel(II))

^{1}H NMR

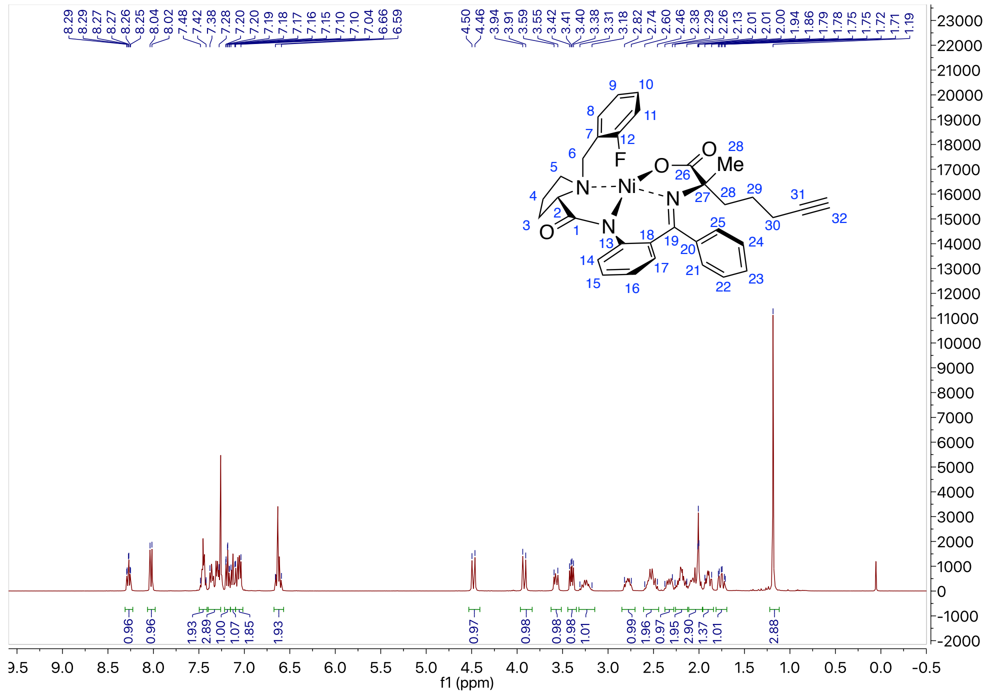

^{13}C NMR

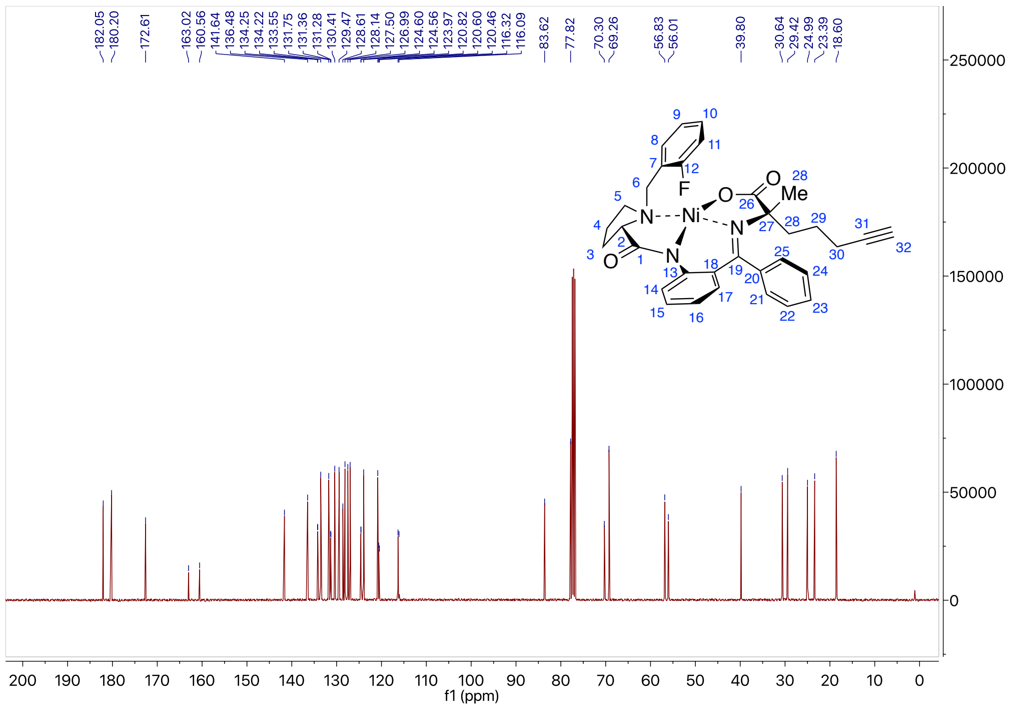

^{19}F NMR

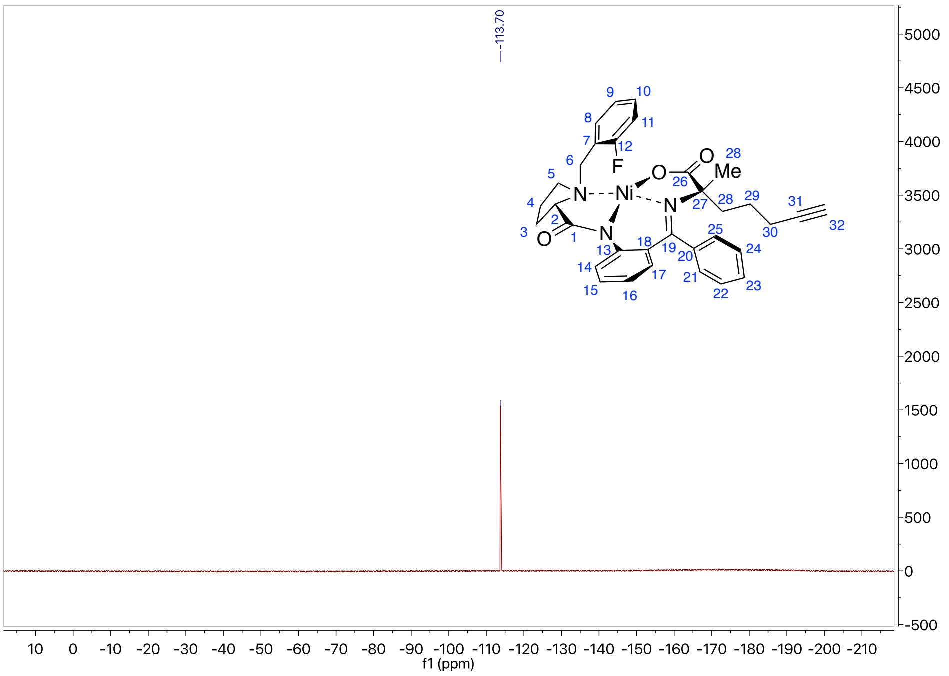

9 (C6 *S* Alkylated Complex - (*S*)-((2-[1-(2-Fluorobenzyl)pyrrolidine-2-carboxamide]phenyl)phenylmethylene)-(*S*)-hexynylalaninato-*N*,*N'*,*N''*,O)nickel(II))

^{1}H NMR

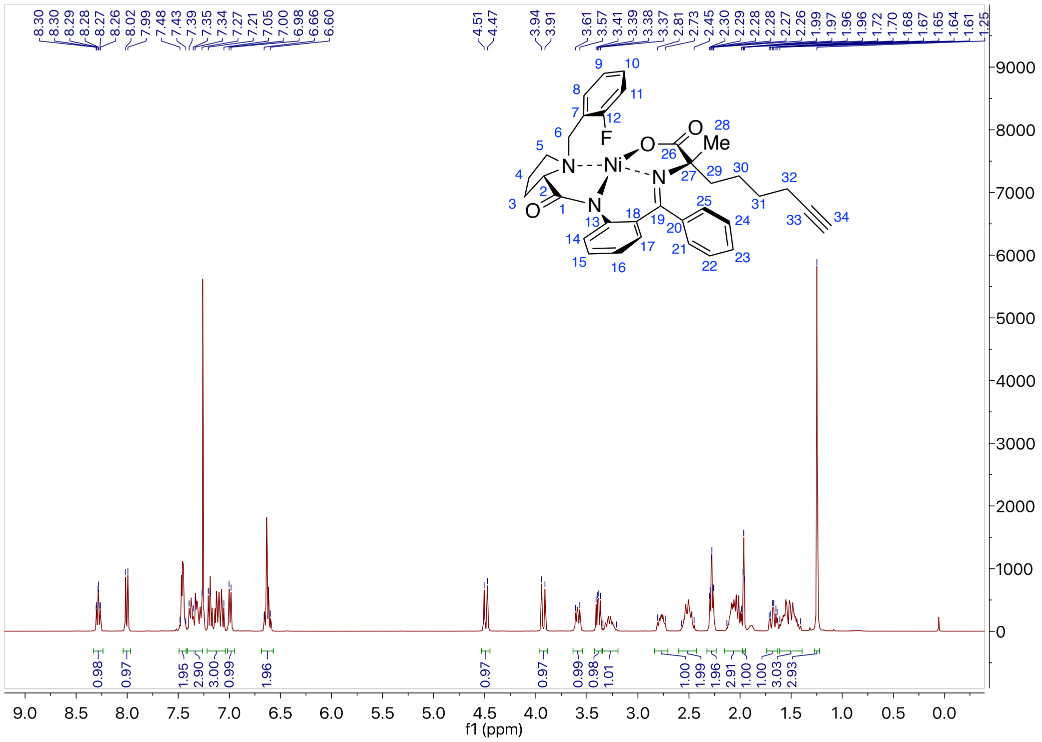

^{13}C NMR

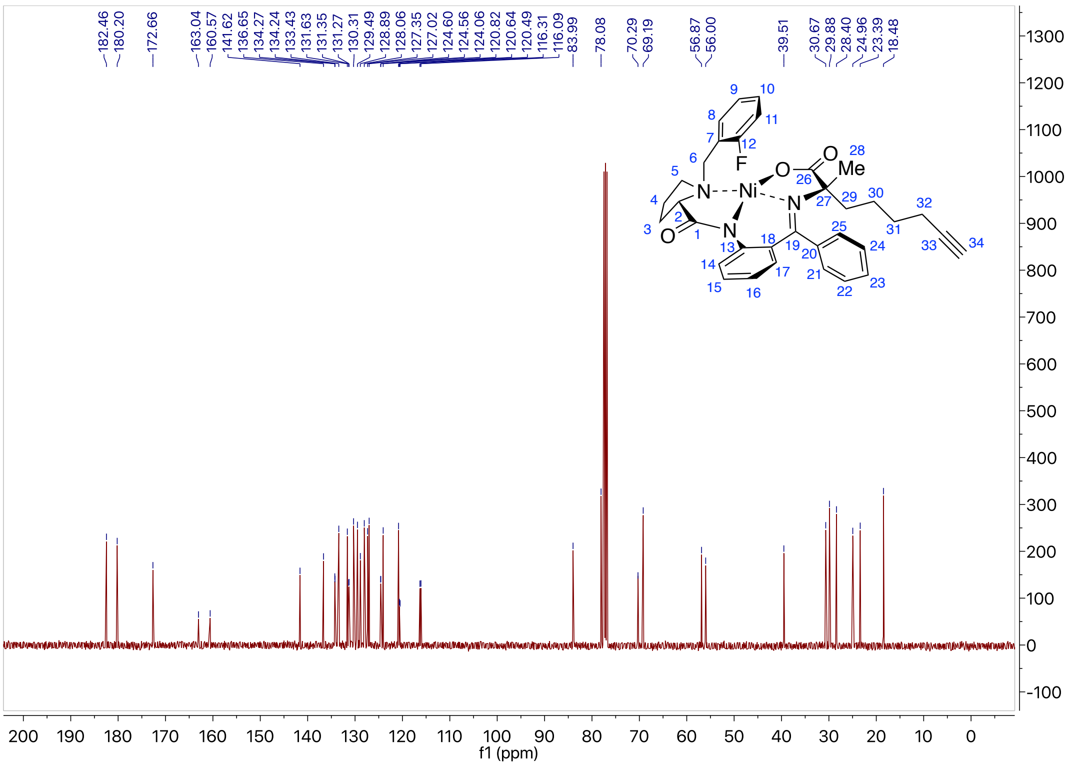

^{19}F NMR

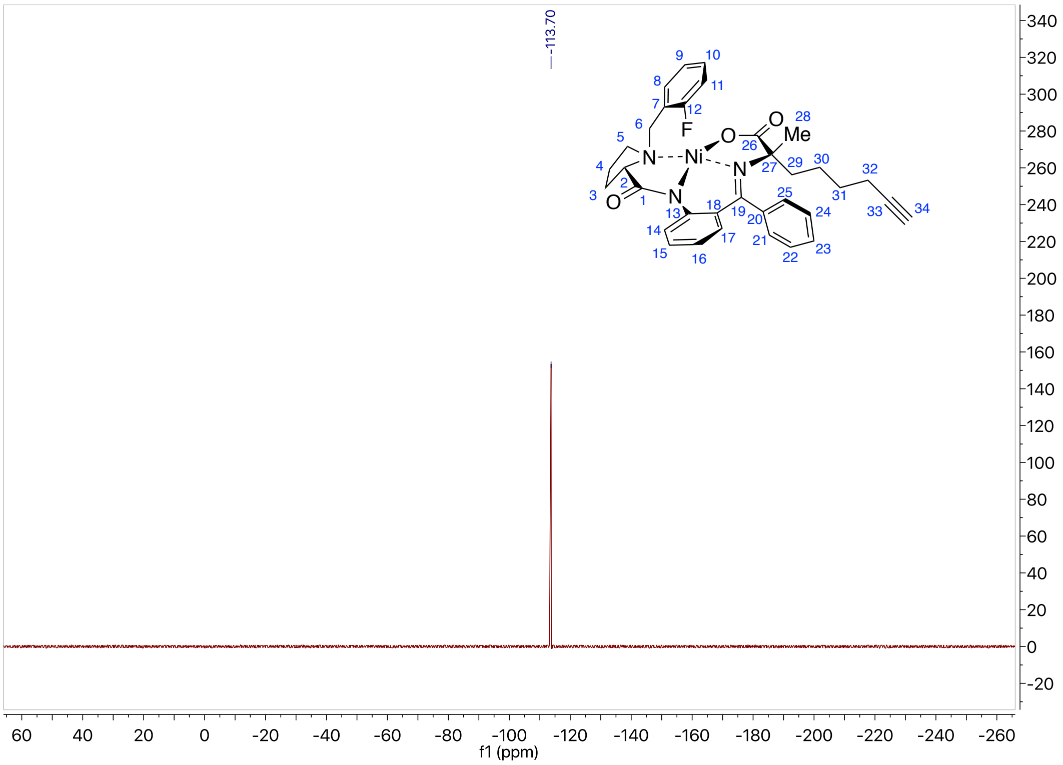

10 (C7 *S* Alkylated Complex - (*S*)-((2-[1-(2-Fluorobenzyl)pyrrolidine-2-carboxamide]phenyl)phenylmethylene)-(*S*)-heptynylalaninato-*N*,*N'*,*N''*,O)nickel(II))

^{1}H NMR

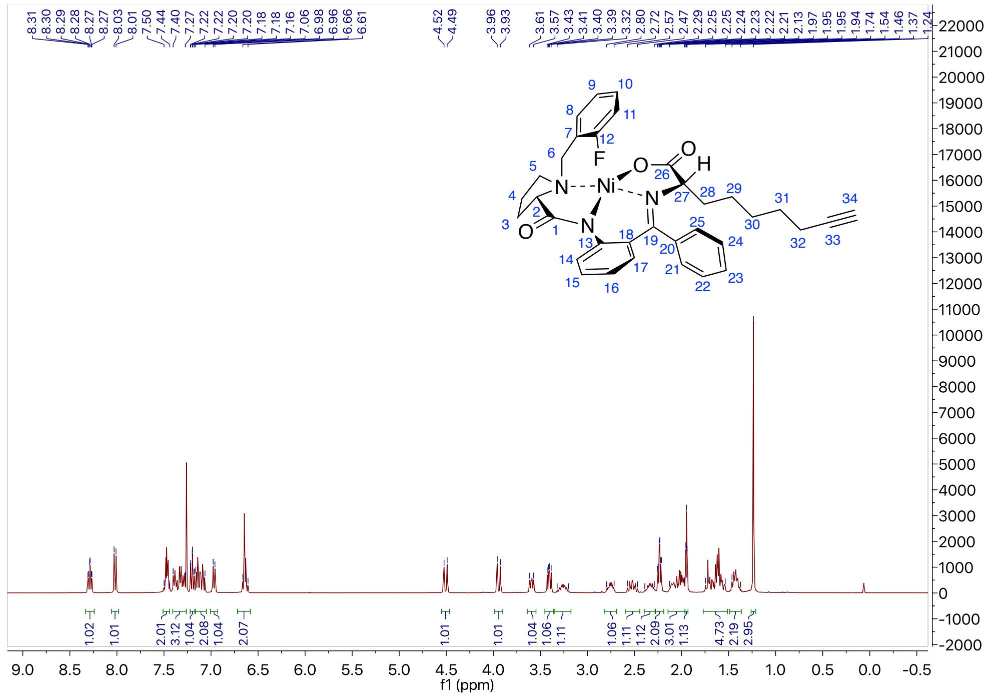

^{13}C NMR

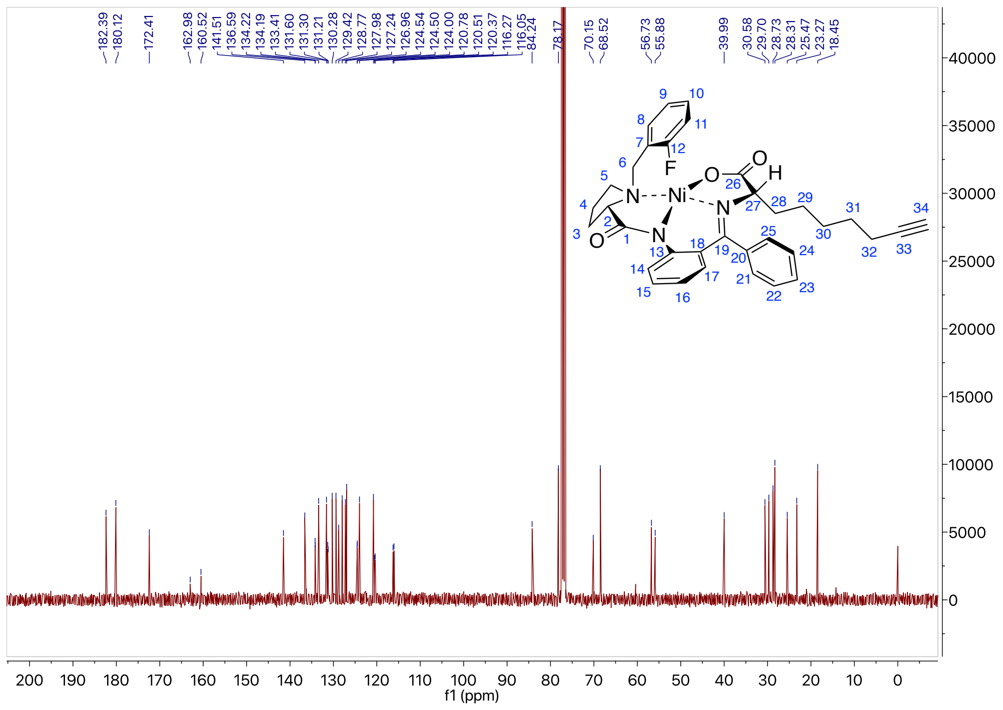

^{19}F NMR

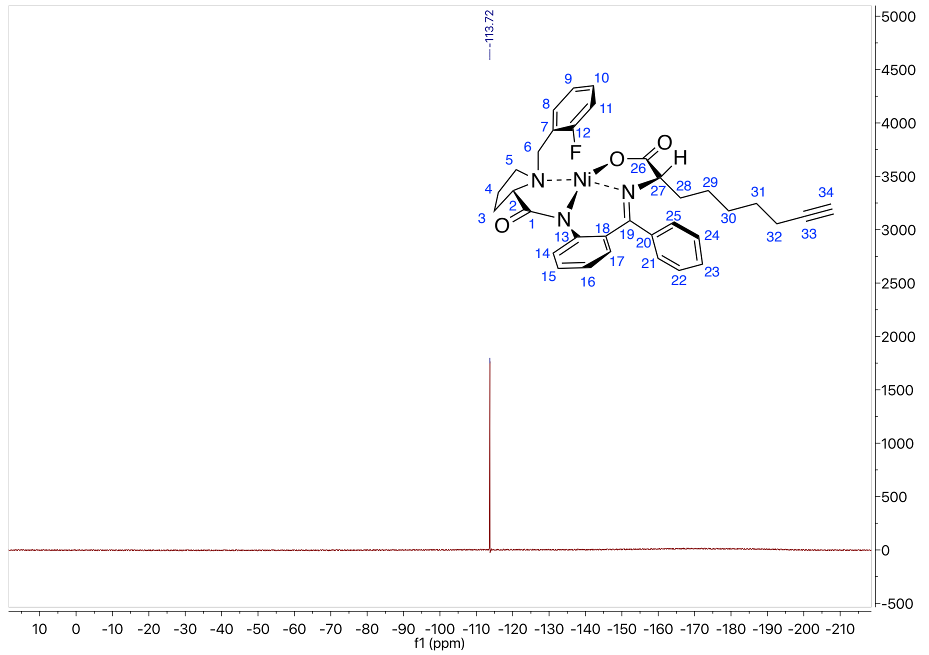

11 (C8 *S* Alkylated Complex - (*S*)-((2-[1-(2-Fluorobenzyl)pyrrolidine-2-carboxamide]phenyl)phenylmethylene)-(*S*)-octynylalaninato-*N*,*N'*,*N''*,O)nickel(II))

^{1}H NMR

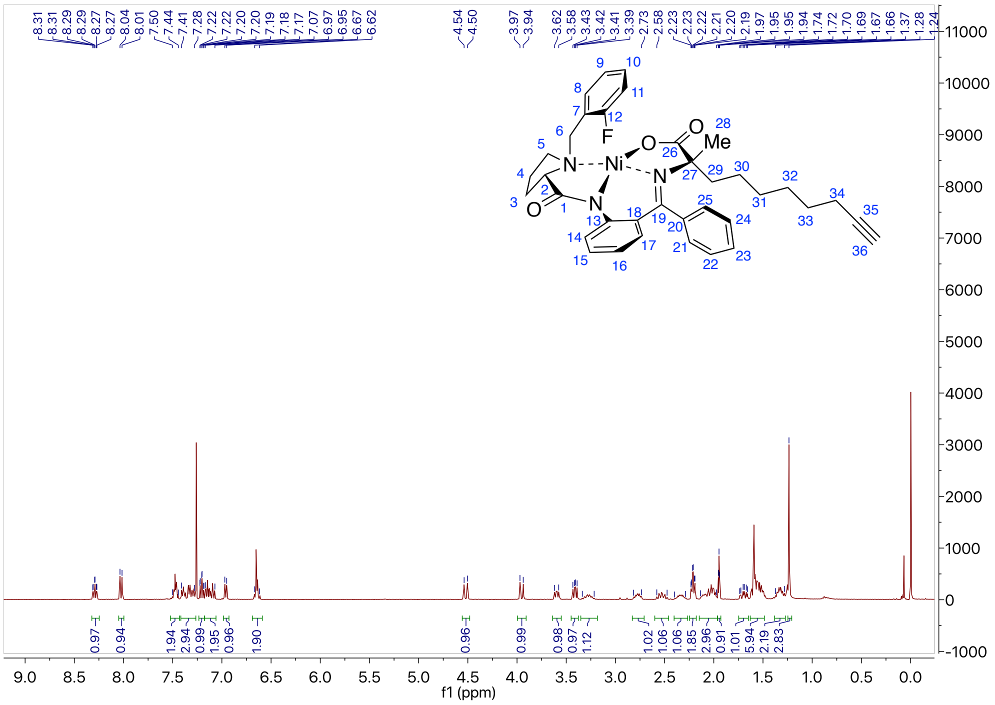

^{13}C NMR

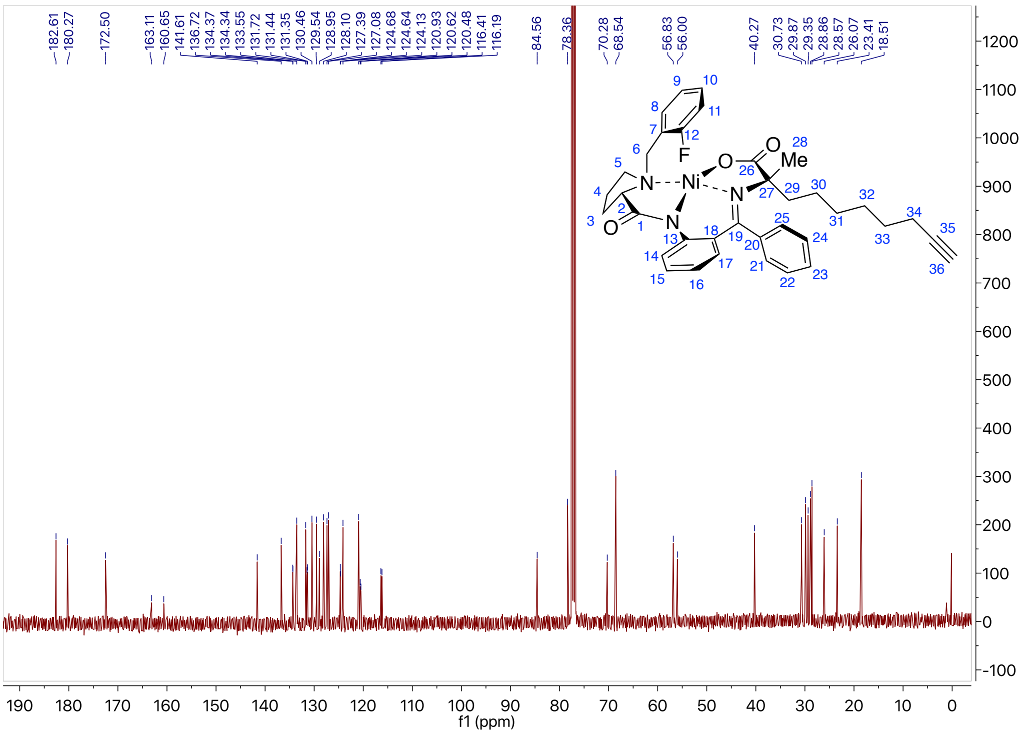

^{19}F NMR

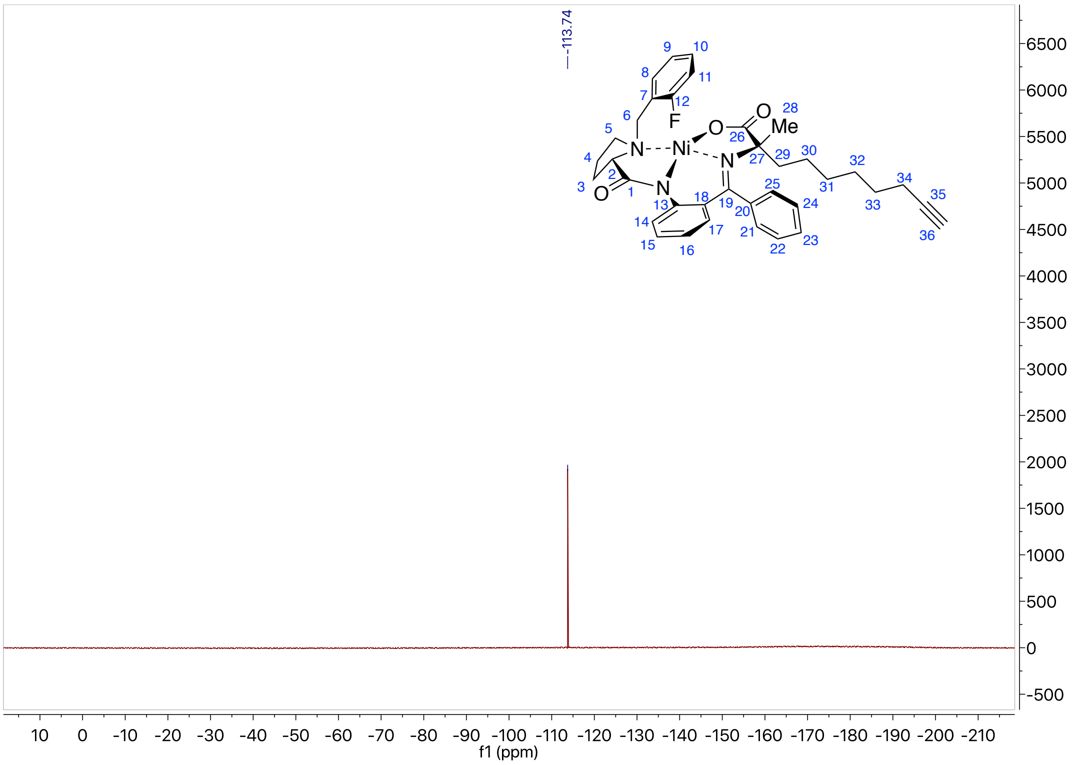

12 (S7 Gly Alkylated Complex - (*S*)-((2-[1-(2-Fluorobenzyl)pyrrolidine-2-carboxamide]phenyl)phenylmethylene)-(*S*)-heptynylglycinato-*N*,*N'*,*N''*,O)nickel(II))

^{1}H NMR

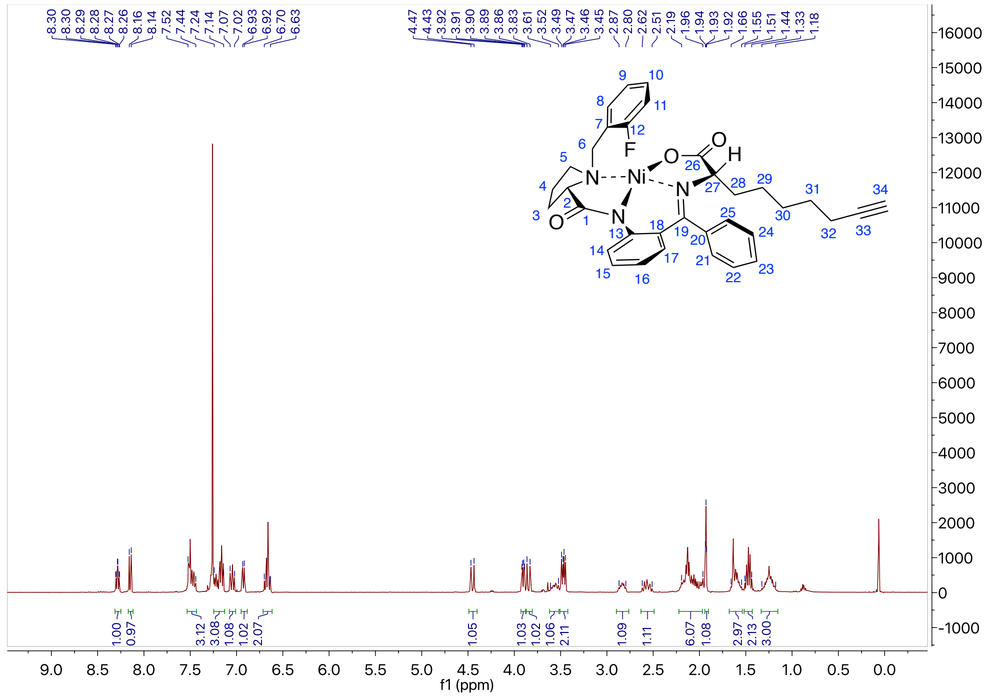

^{13}C NMR

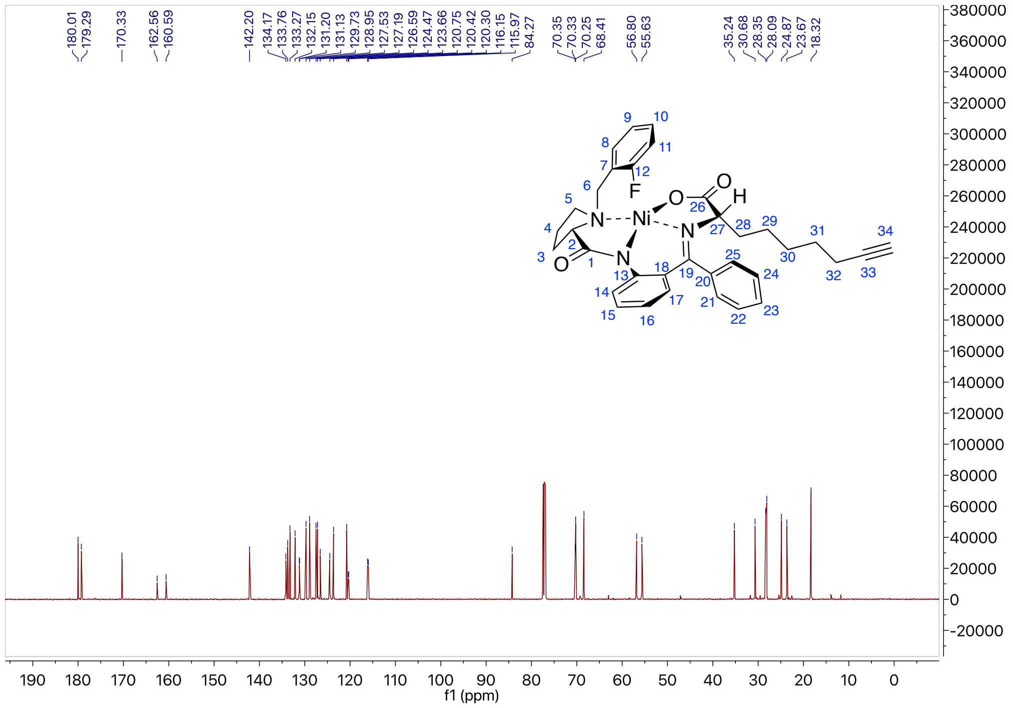

^{19}F NMR

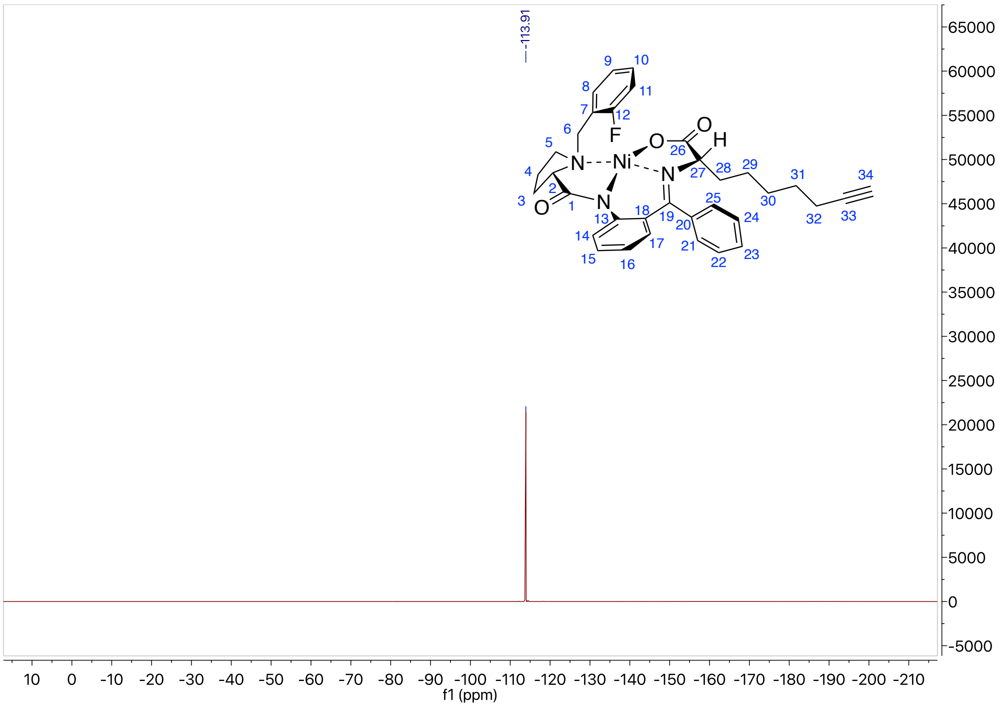

13 (C5 *R* Alkylated Complex - (*R*)-((2-[1-(2-Fluorobenzyl)pyrrolidine-2-carboxamide]phenyl)phenylmethylene)-(*R*)-pentynylalaninato-*N*,*N'*,*N''*,O)nickel(II))

^{1}H NMR

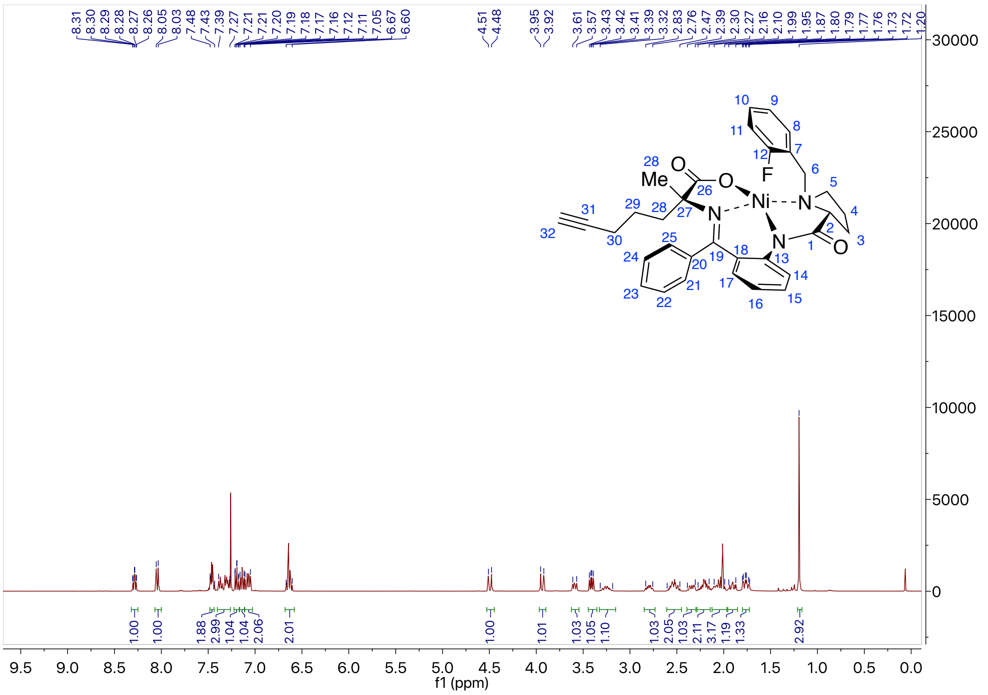

^{13}C NMR

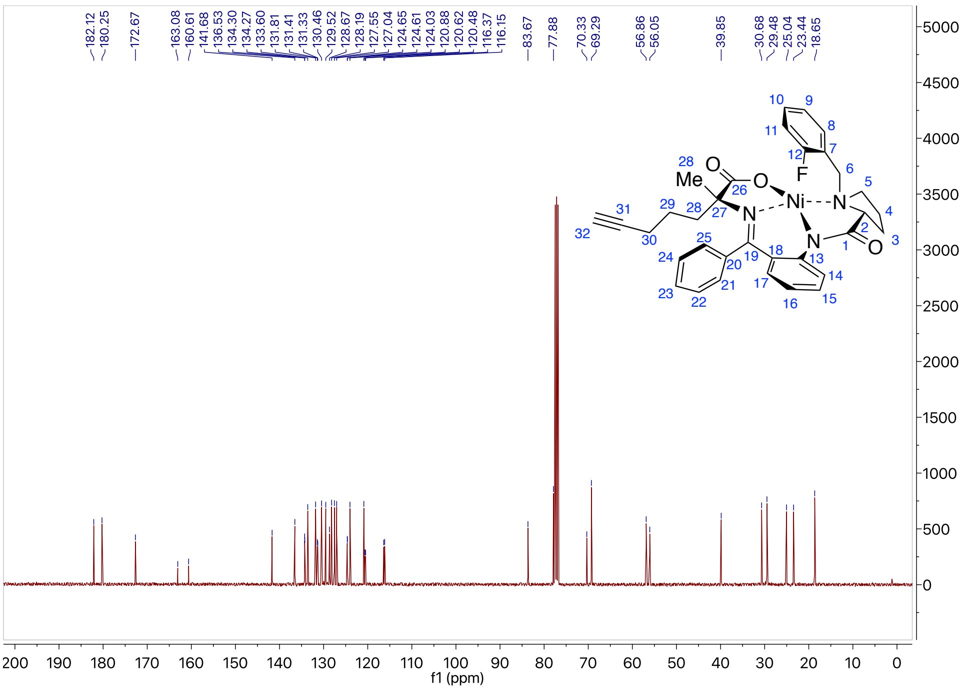

^{19}F NMR

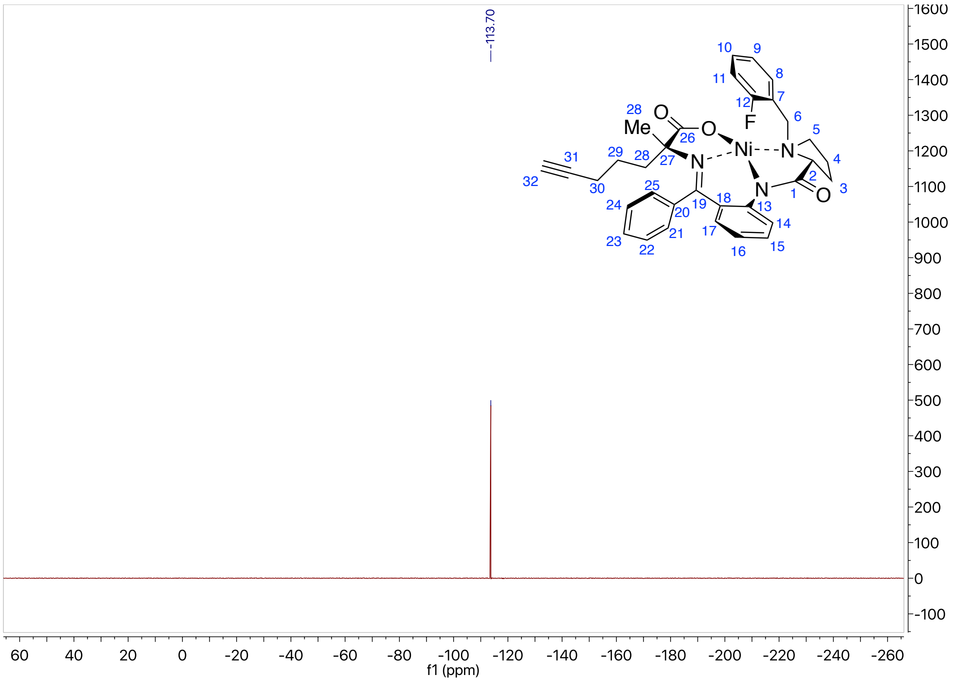

14 (C6 *R* Alkylated Complex - (*R*)-((2-[1-(2-Fluorobenzyl)pyrrolidine-2-carboxamide]phenyl)phenylmethylene)-(*R*)-hexynylalaninato-*N*,*N'*,*N''*,O)nickel(II))

^{1}H NMR

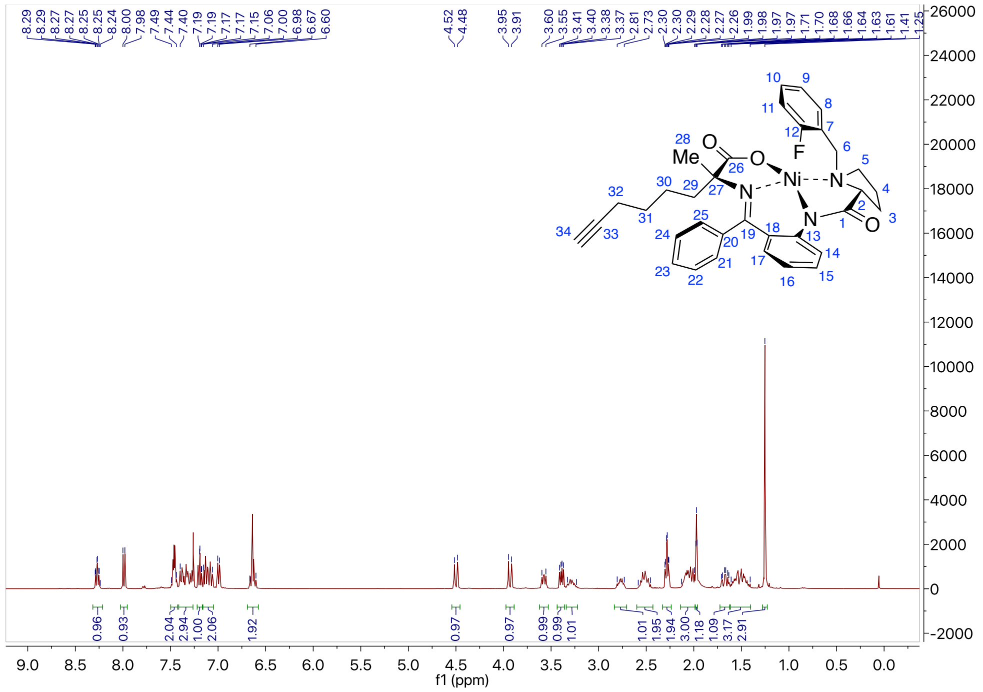

^{13}C NMR

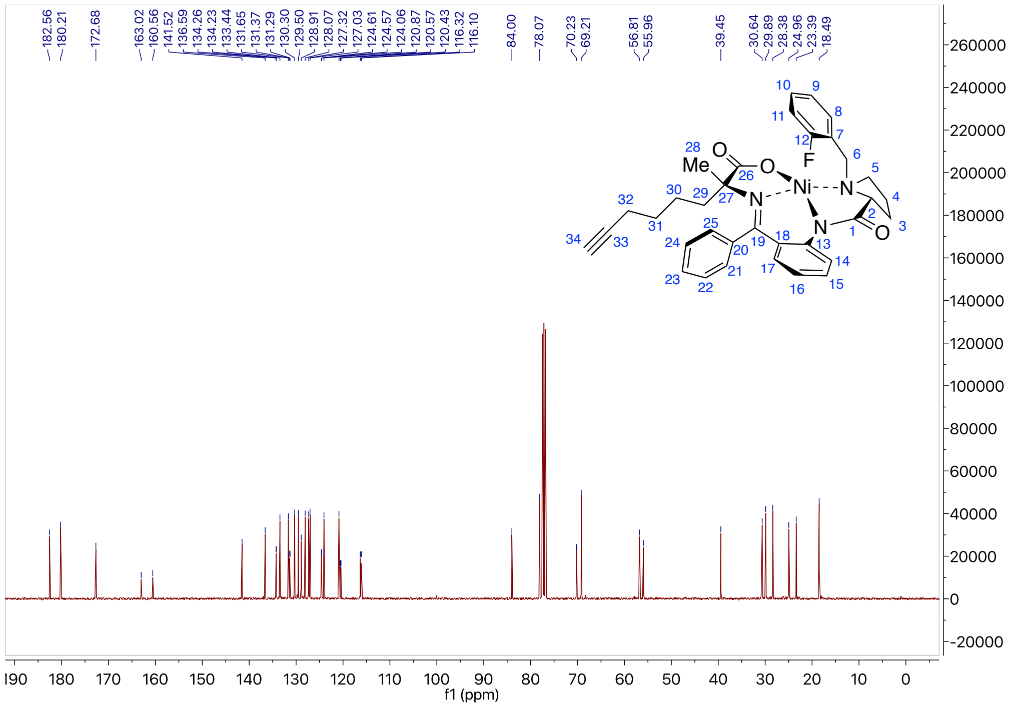

^{19}F NMR

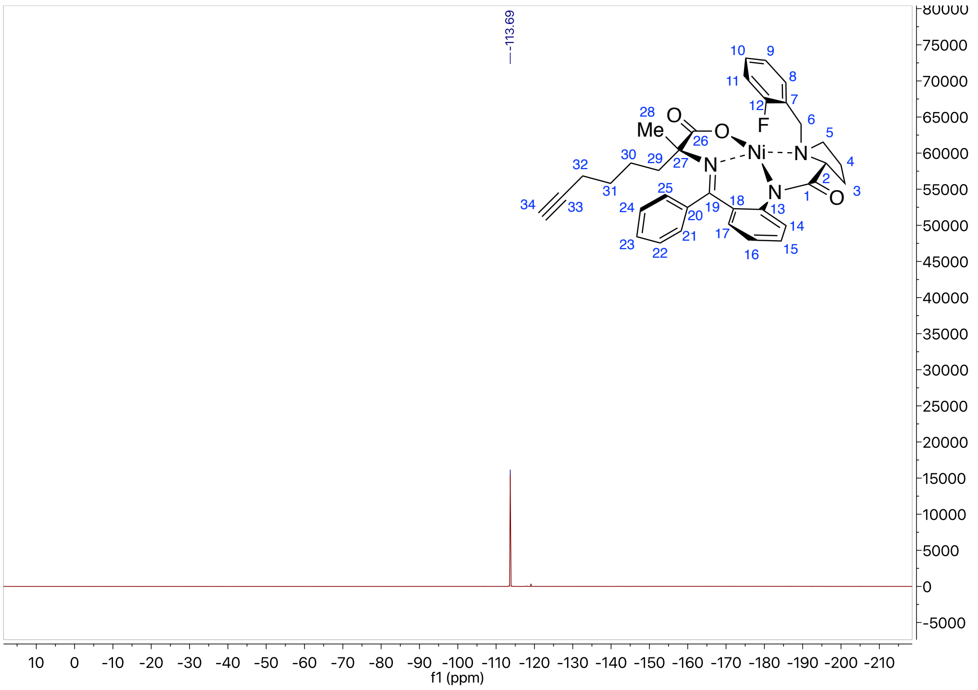

15 (C7 *R* Alkylated Complex - (*R*)-((2-[1-(2-Fluorobenzyl)pyrrolidine-2-carboxamide]phenyl)phenylmethylene)-(*R*)-heptynylalaninato-*N*,*N'*,*N''*,O)nickel(II))

^{1}H NMR

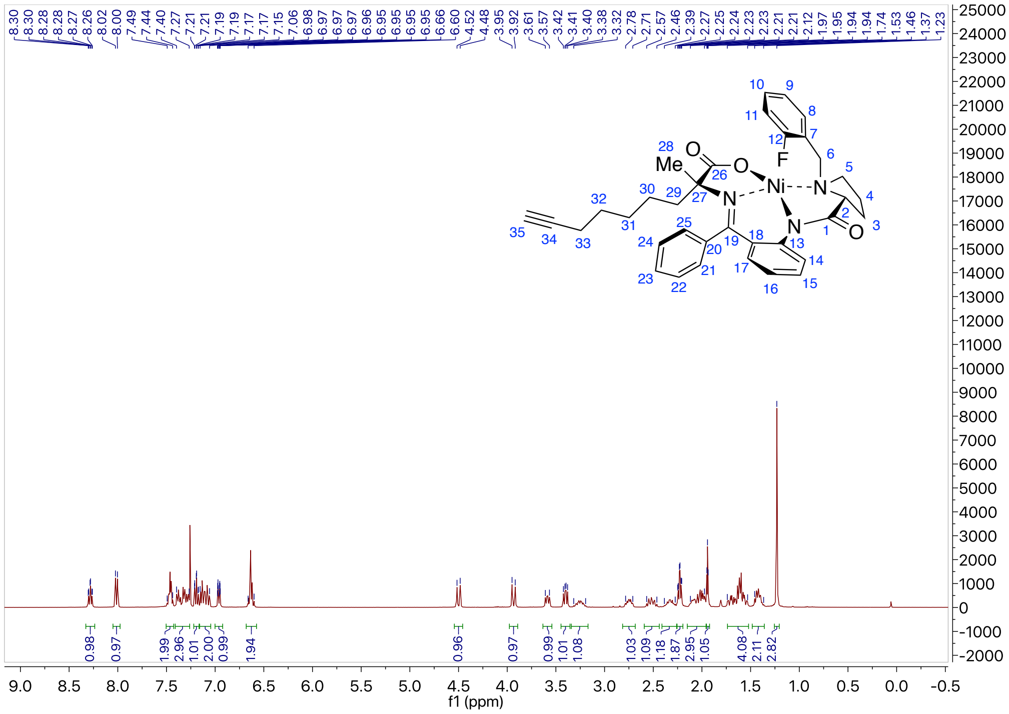

^{13}C NMR

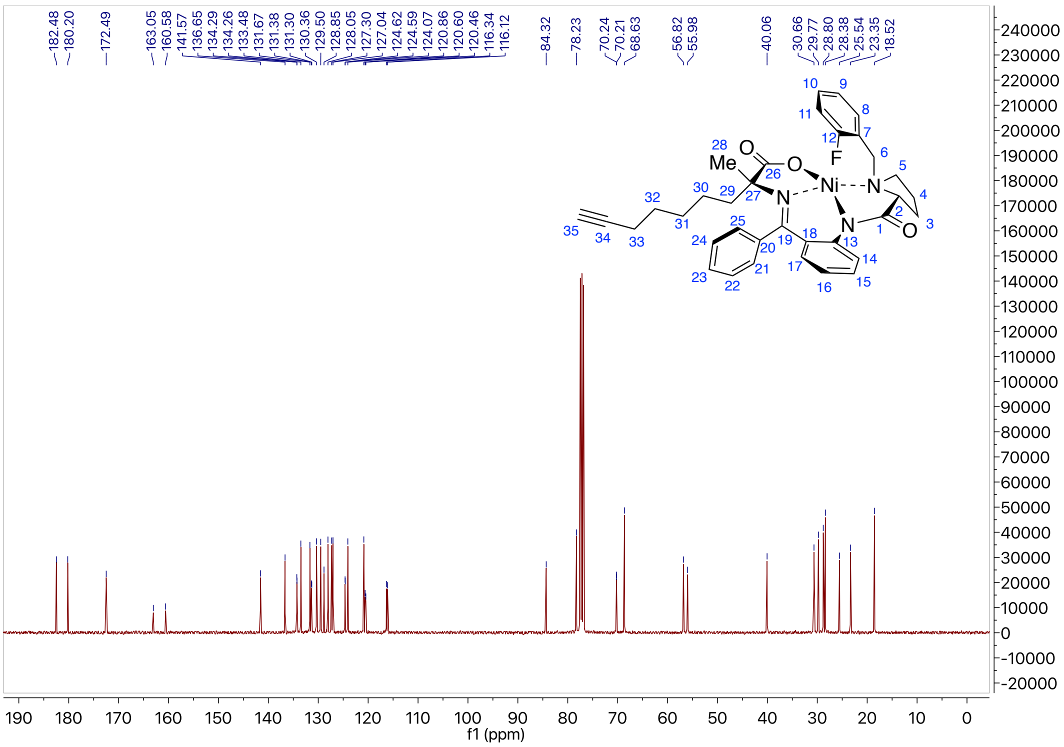

^{19}F NMR

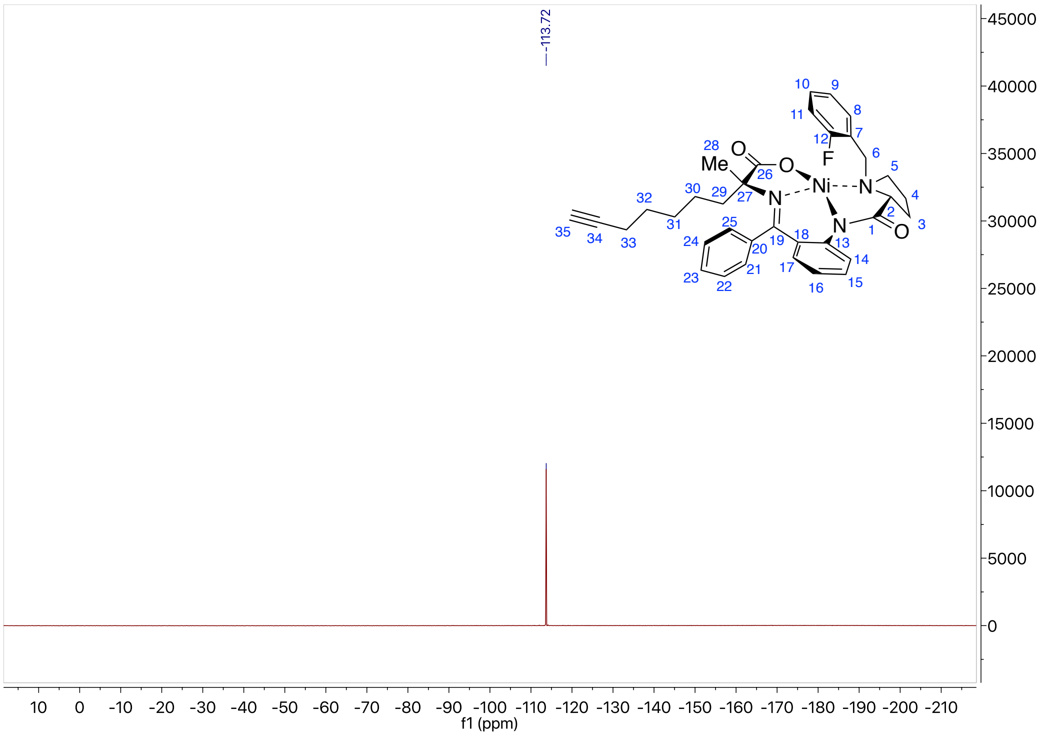

16 (C8 *R* Alkylated Complex - (*R*)-((2-[1-(2-Fluorobenzyl)pyrrolidine-2-carboxamide]phenyl)phenylmethylene)- (*R*)-octynylalaninato-*N*,*N'*,*N''*,O)nickel(II))

^{1}H NMR

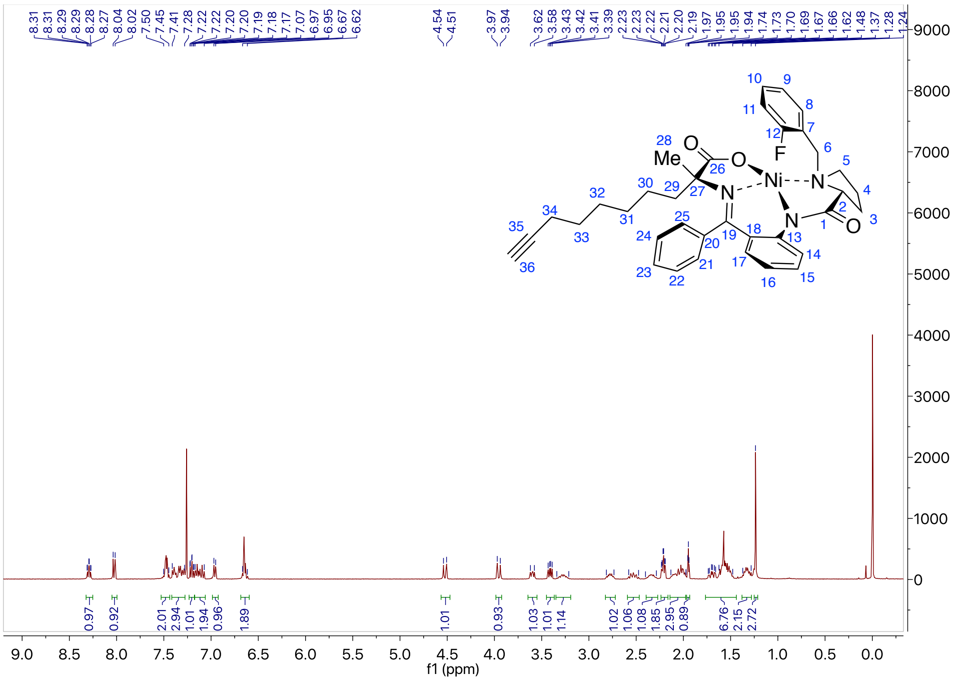

^{13}C NMR

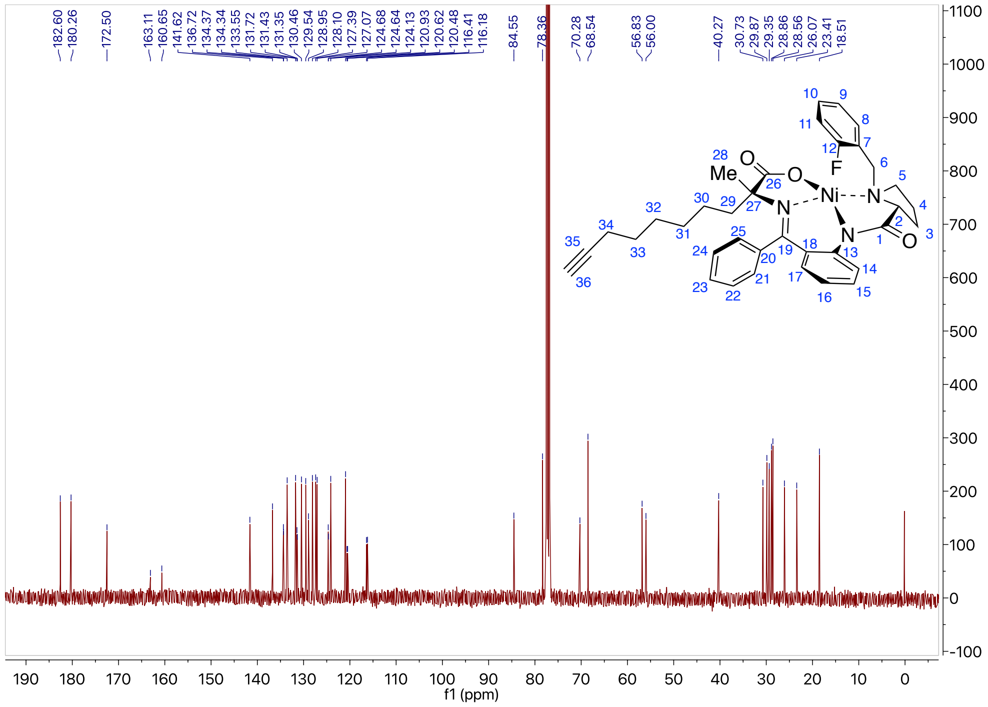

^{19}F NMR

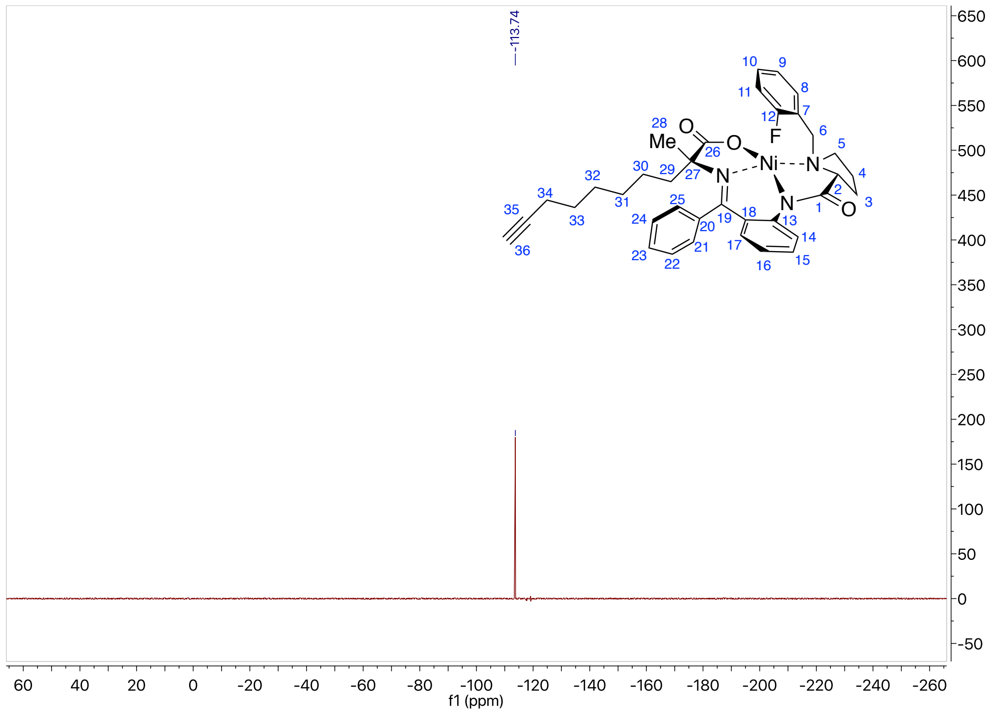

17 (C5 *S* AA - (*S*)-2-((((9*H*-fluoren-9-yl)methoxy)carbonyl)amino)-2-methylhept-6-ynoic acid)

^{1}H NMR

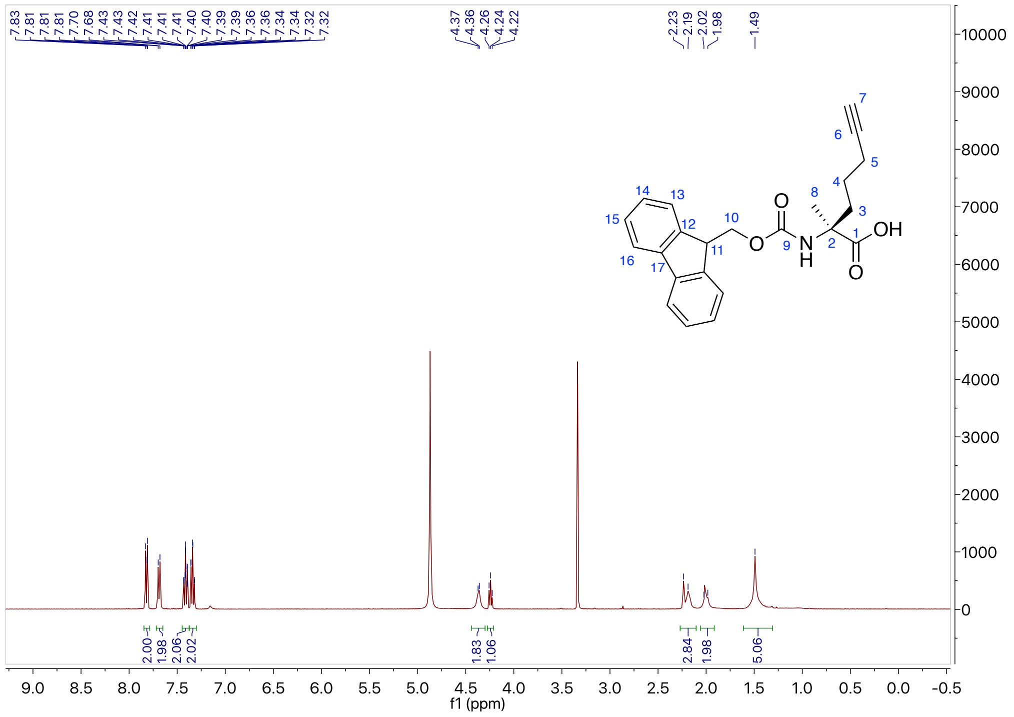

^{13}C NMR

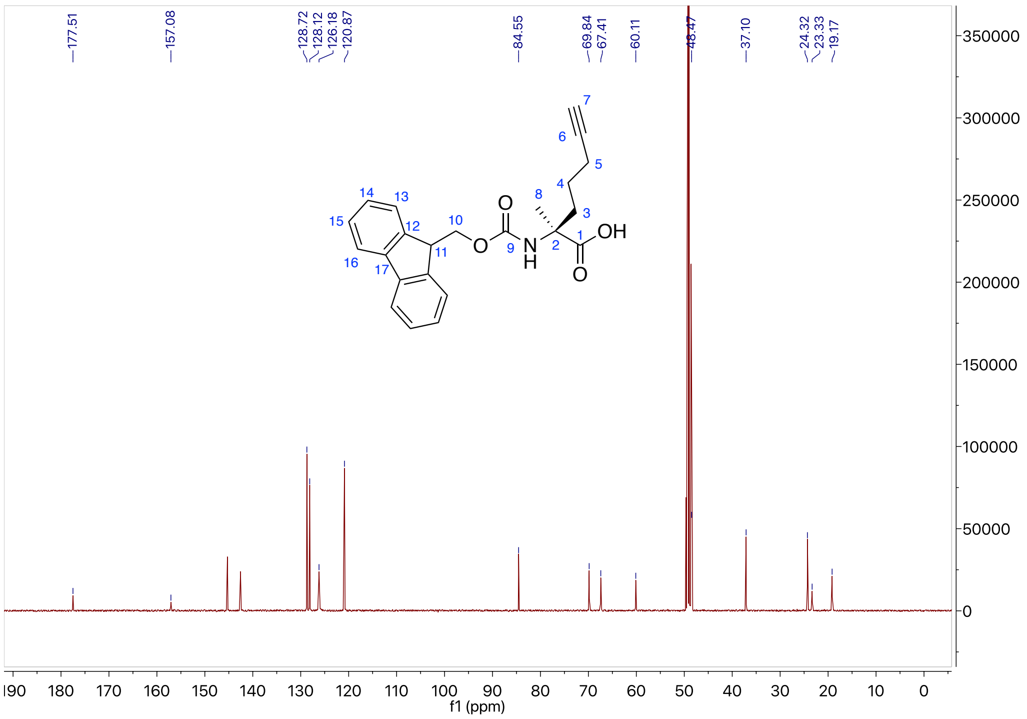

18 (C6 *S* AA - (*S*)-2-((((9H-fluoren-9-yl)methoxy)carbonyl)amino)-2-methyloct-7-ynoic acid)

^{1}H NMR

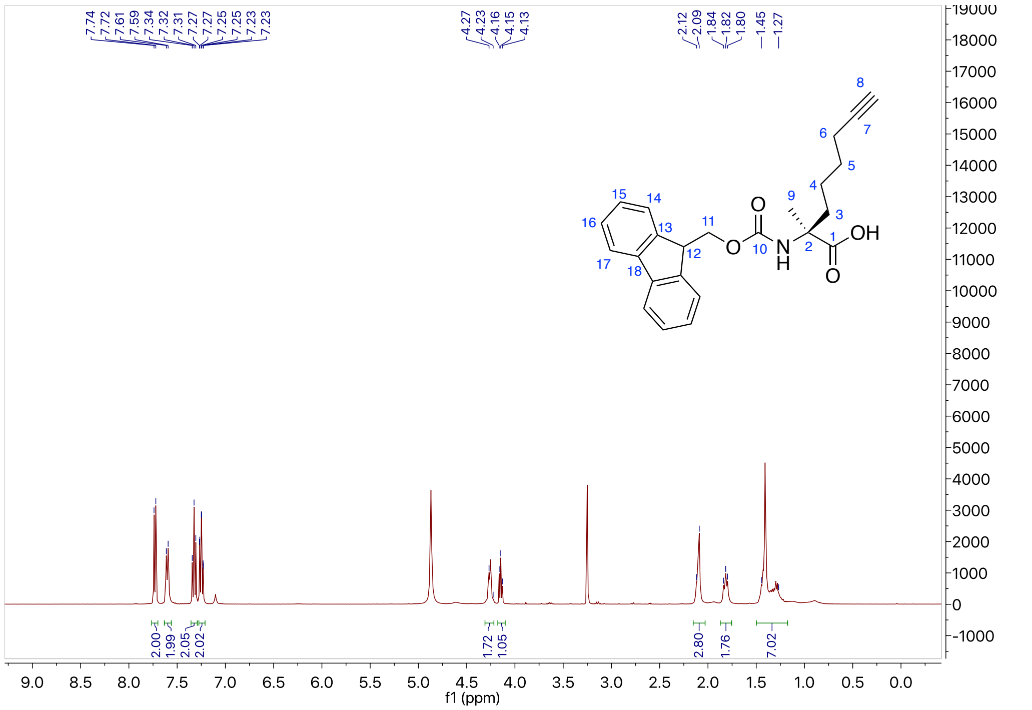

^{13}C NMR

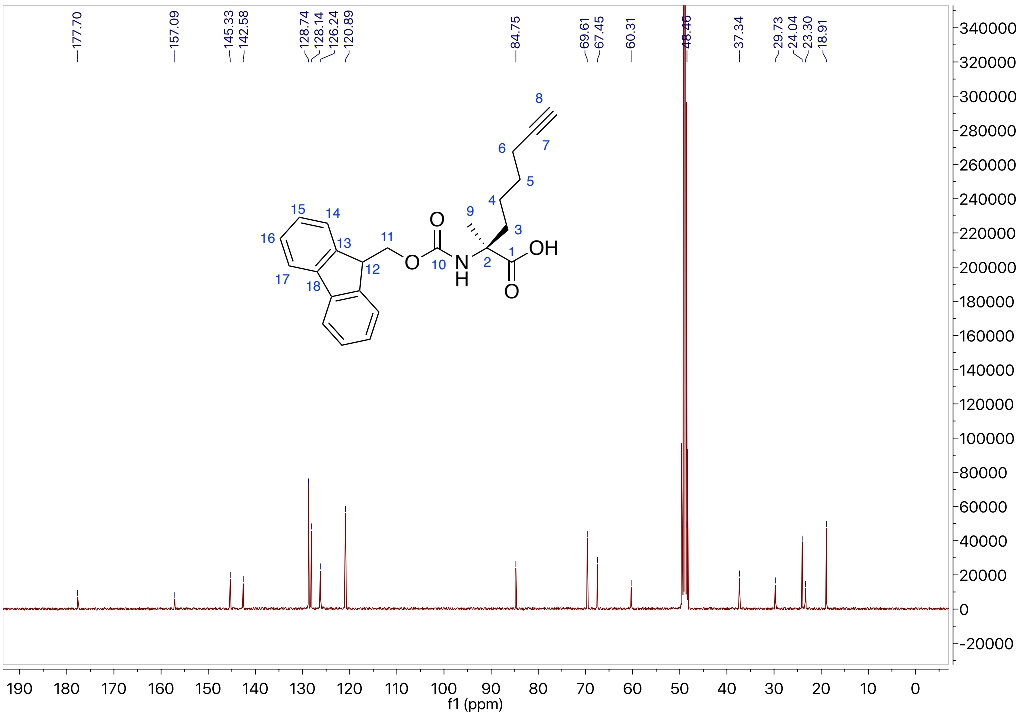

19 (C7 *S* AA - (*S*)-2-((((9*H*-Fluoren-9-yl)methoxy)carbonyl)amino)-2-methylnon-8-ynoic acid)

^{1}H NMR

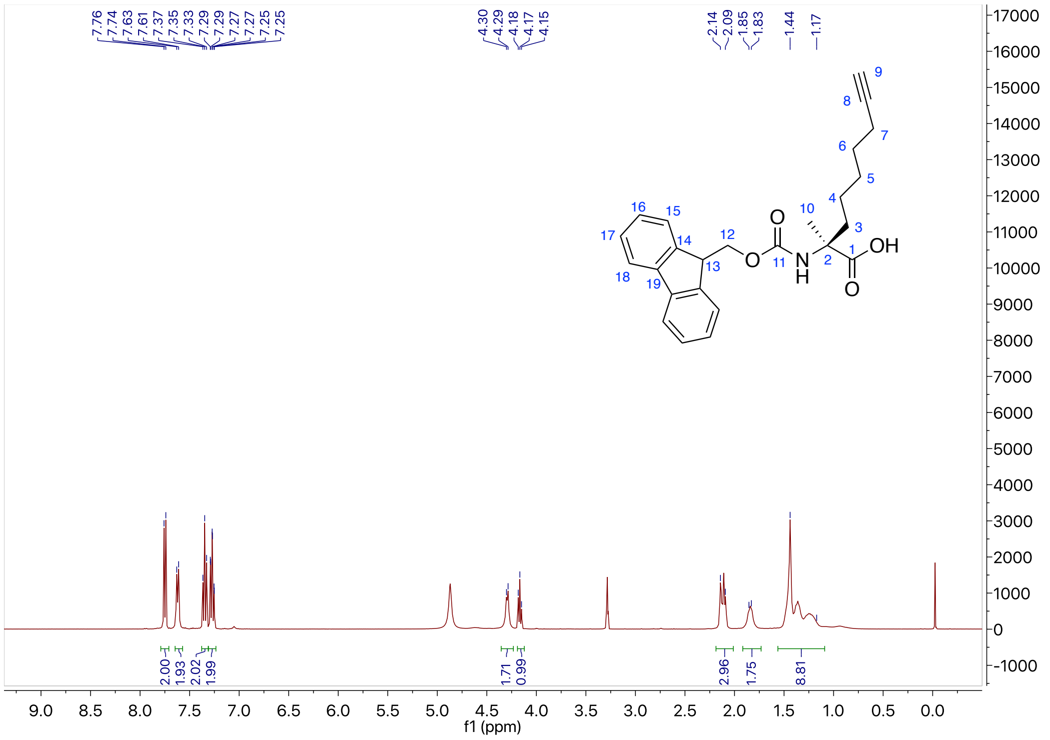

^{13}C NMR

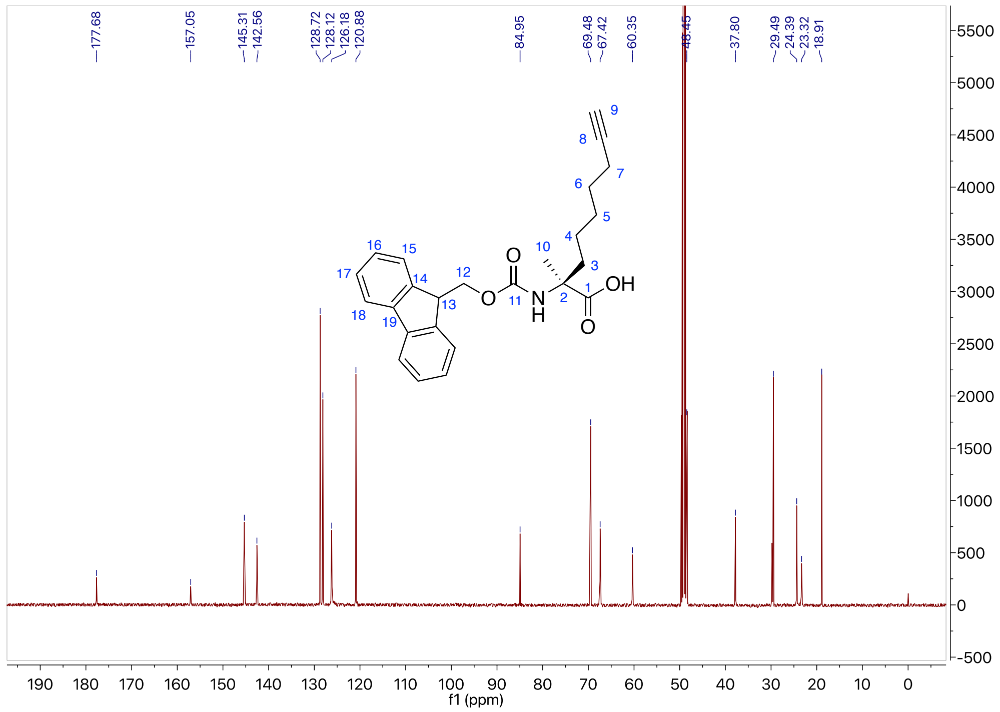

20 (C8 *S* AA - (*S*)-2-((((9*H*-Fluoren-9-yl)methoxy)carbonyl)amino)-2-methyldec-9-ynoic acid)

^{1}H NMR

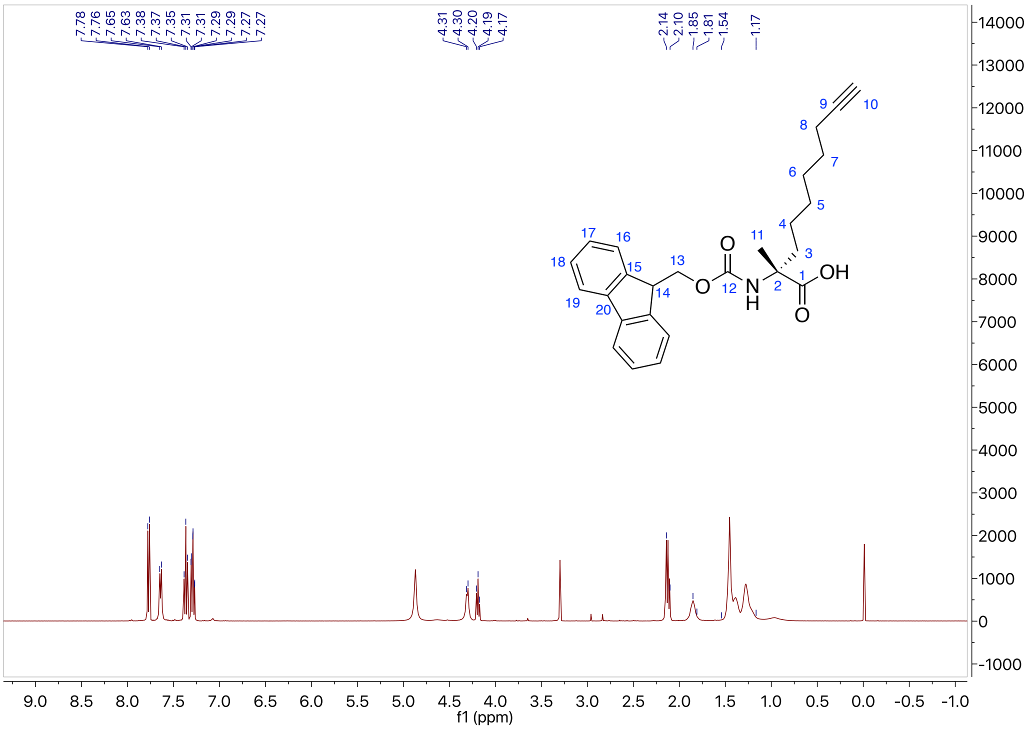

^{13}C NMR

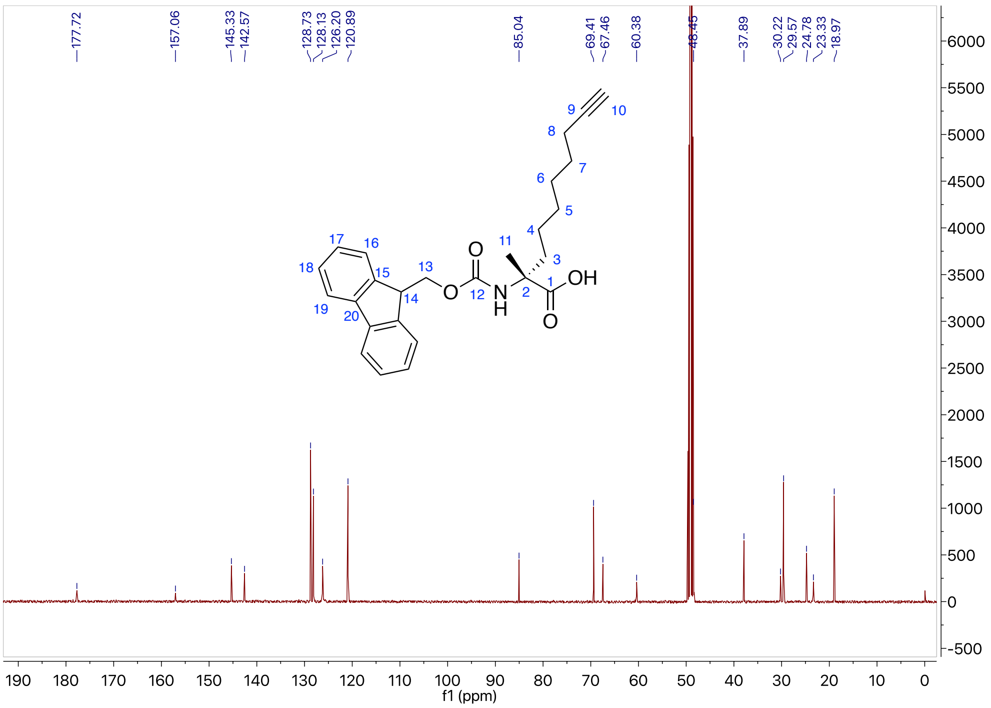

21 (C7 *S* H AA - (*S*)-2-((((9*H*-Fluoren-9-yl)methoxy)carbonyl)amino)-non-8-ynoic acid)

^{1}H NMR

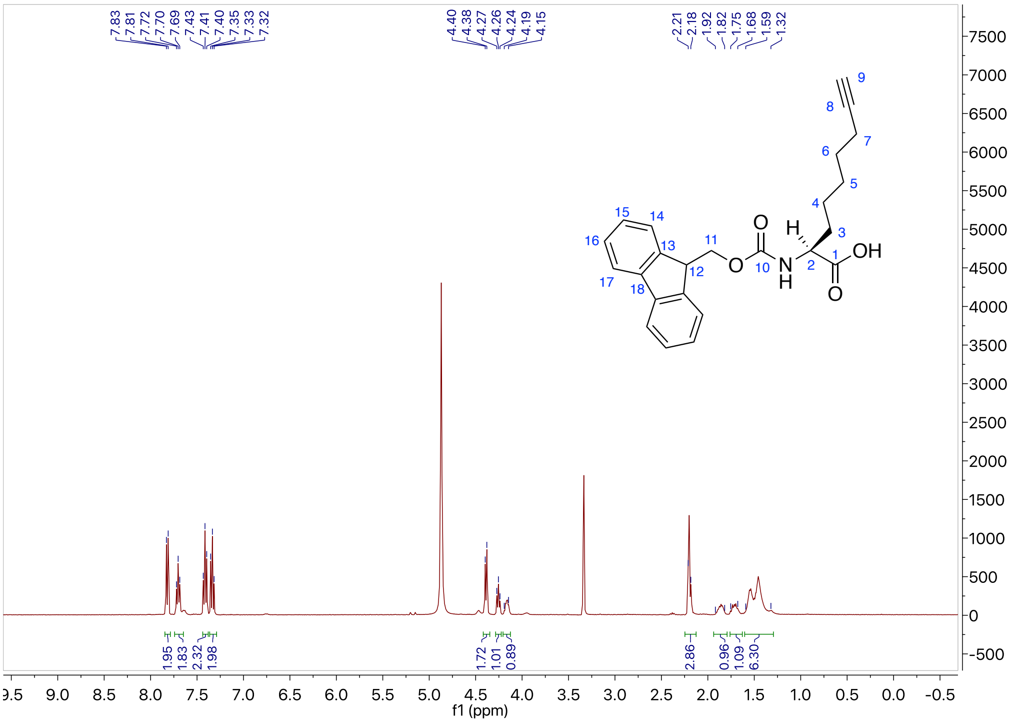

^{13}C NMR

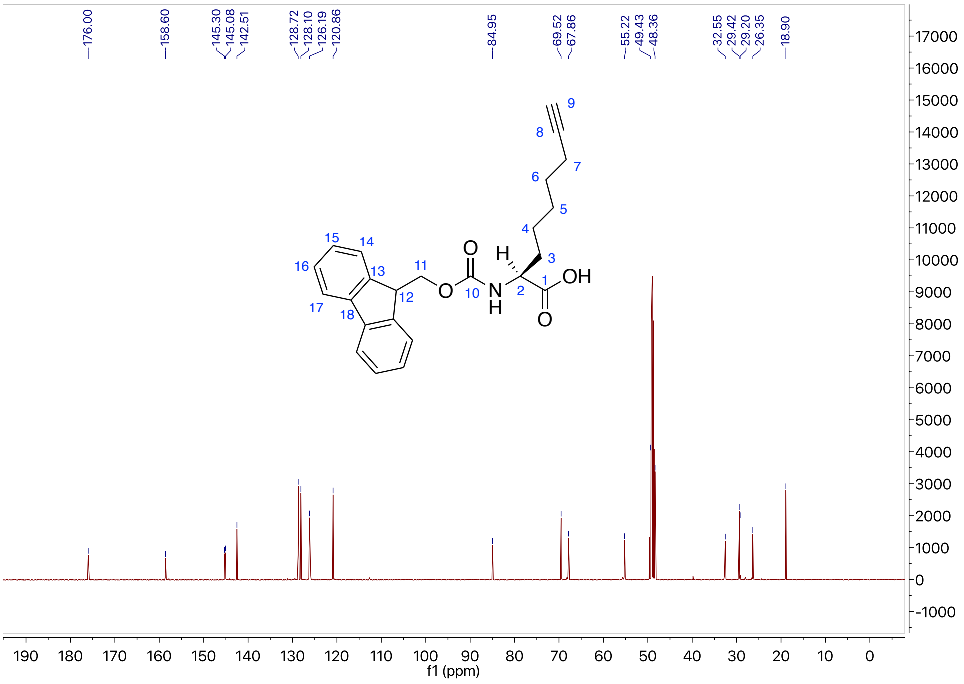

22 (C5 *R* AA - (*R*)-2-((((9*H*-fluoren-9-yl)methoxy)carbonyl)amino)-2-methylhept-6-ynoic acid)

^{1}H NMR

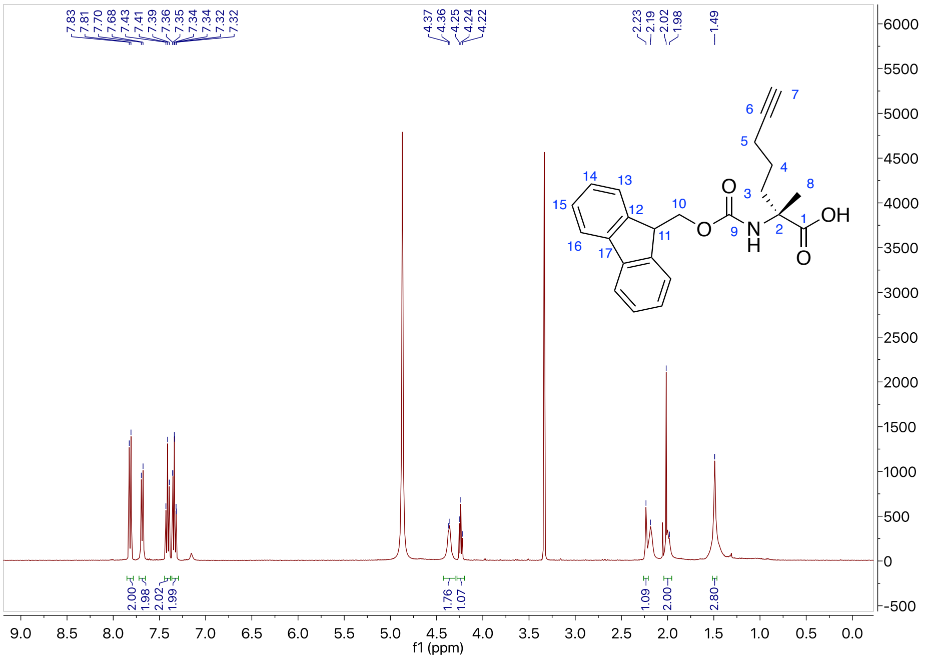

^{13}C NMR

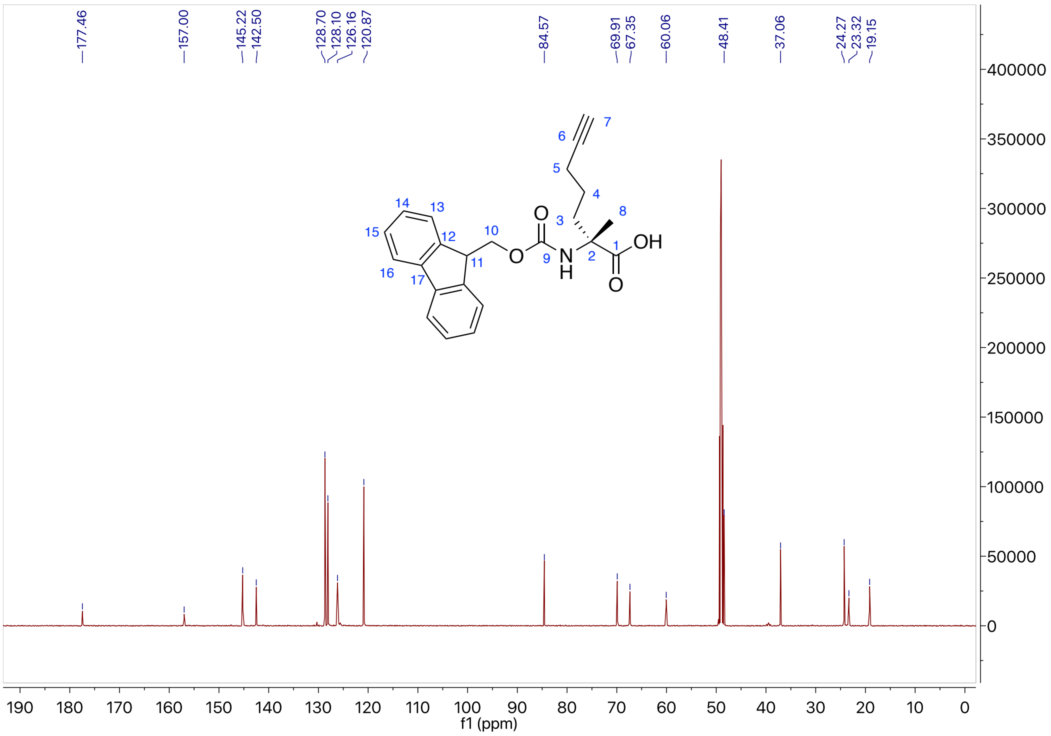

23 (C6 *R* AA - ((*R*)-2-((((9H-fluoren-9-yl)methoxy)carbonyl)amino)-2-methyloct-7-ynoic acid)

^{1}H NMR

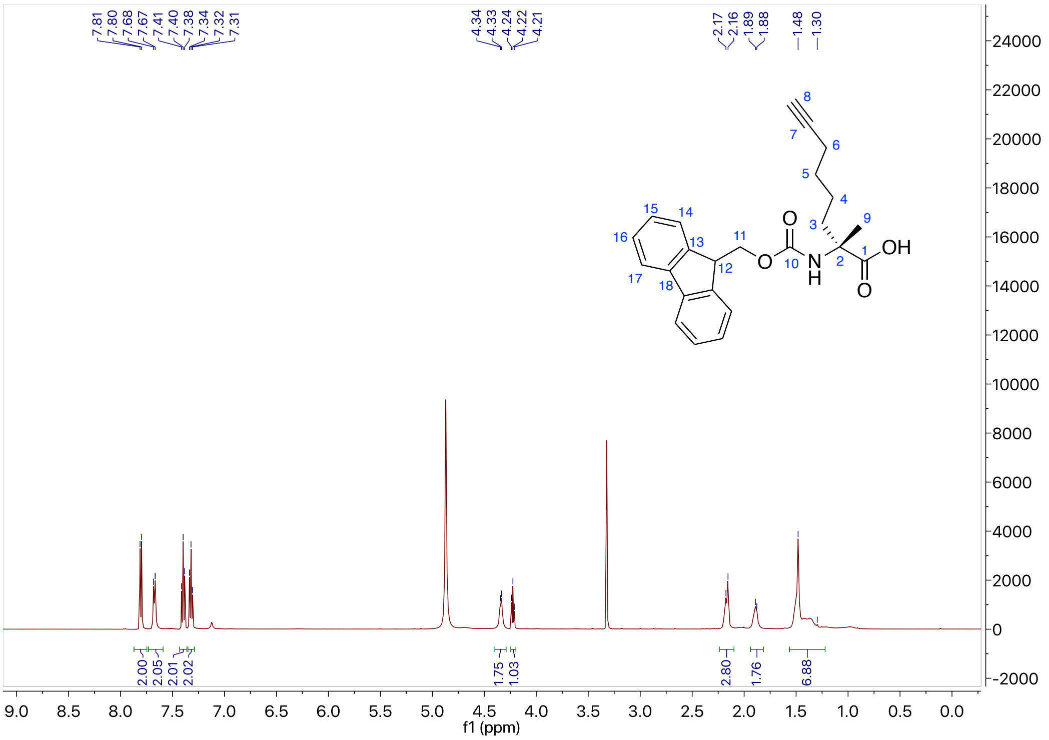

^{13}C NMR

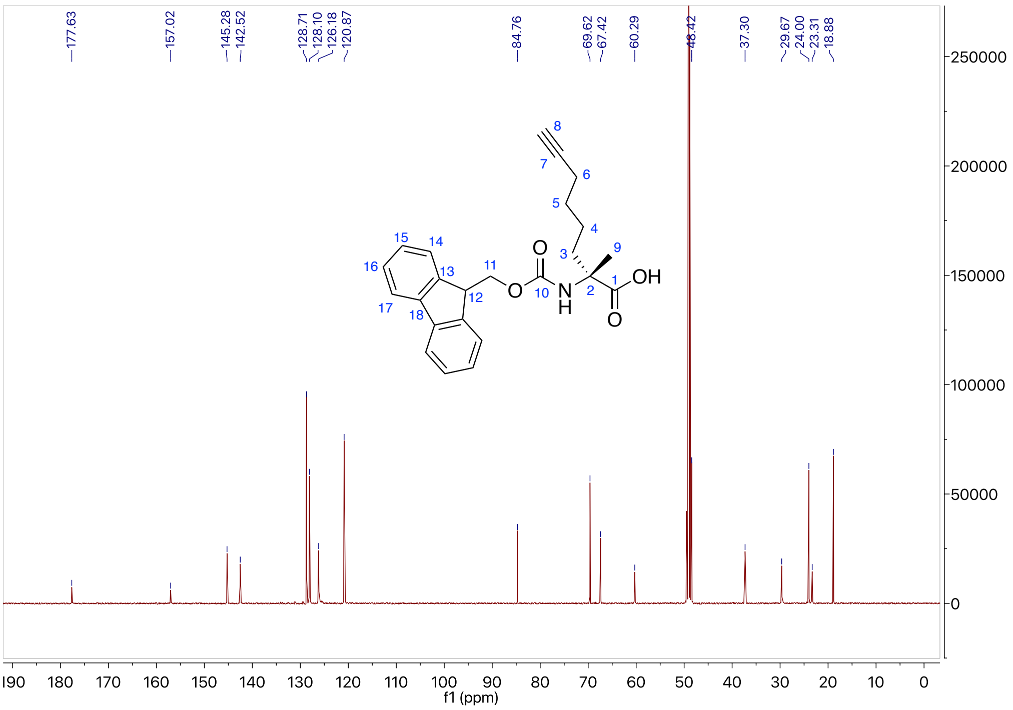

24 (C7 *R* AA - (*R*)-2-((((9*H*-Fluoren-9-yl)methoxy)carbonyl)amino)-2-methylnon-8-ynoic acid)

^{1}H NMR

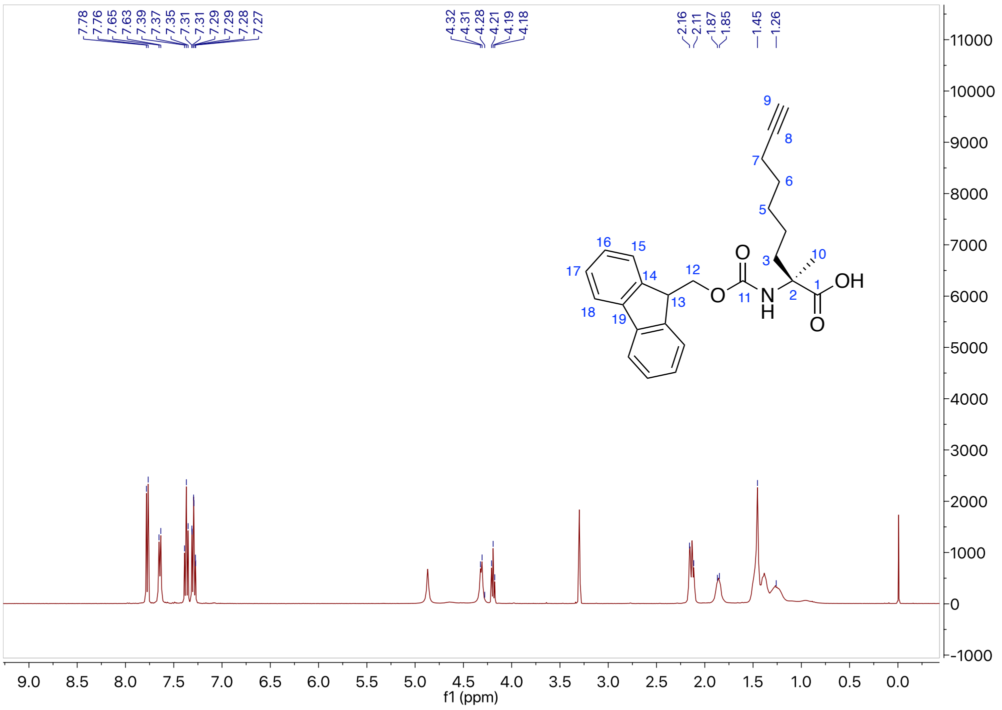

^{13}C NMR

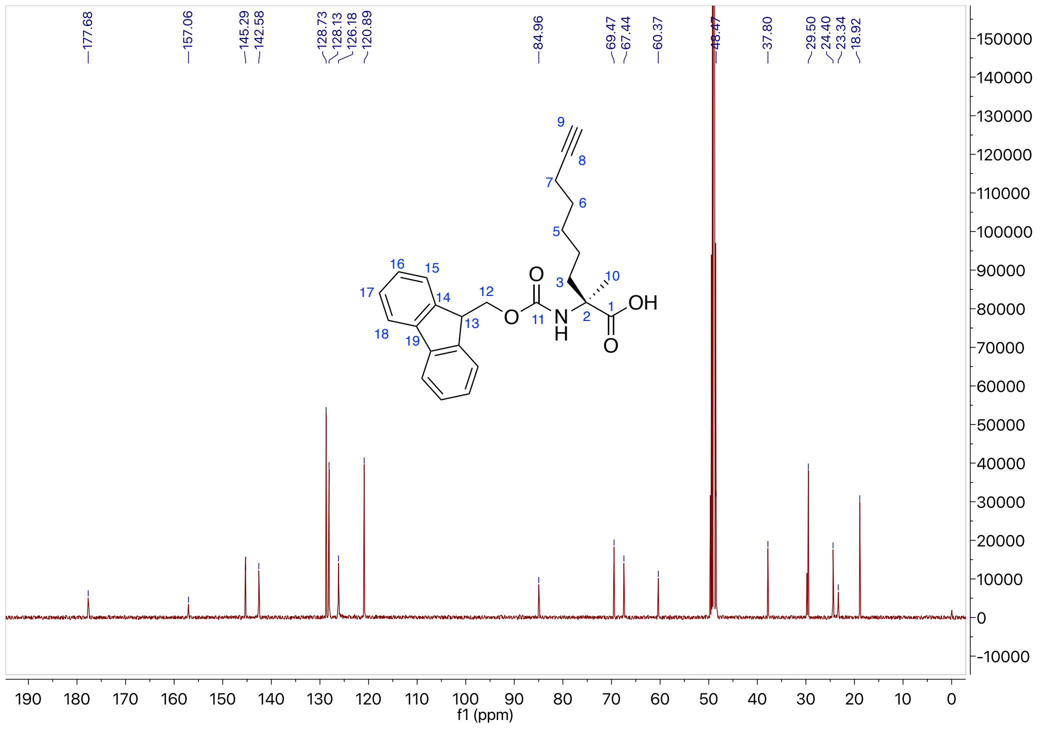

25 (C8 *R* AA - (*R*)-2-((((9*H*-Fluoren-9-yl)methoxy)carbonyl)amino)-2-methyldec-9-ynoic acid)

^{1}H NMR

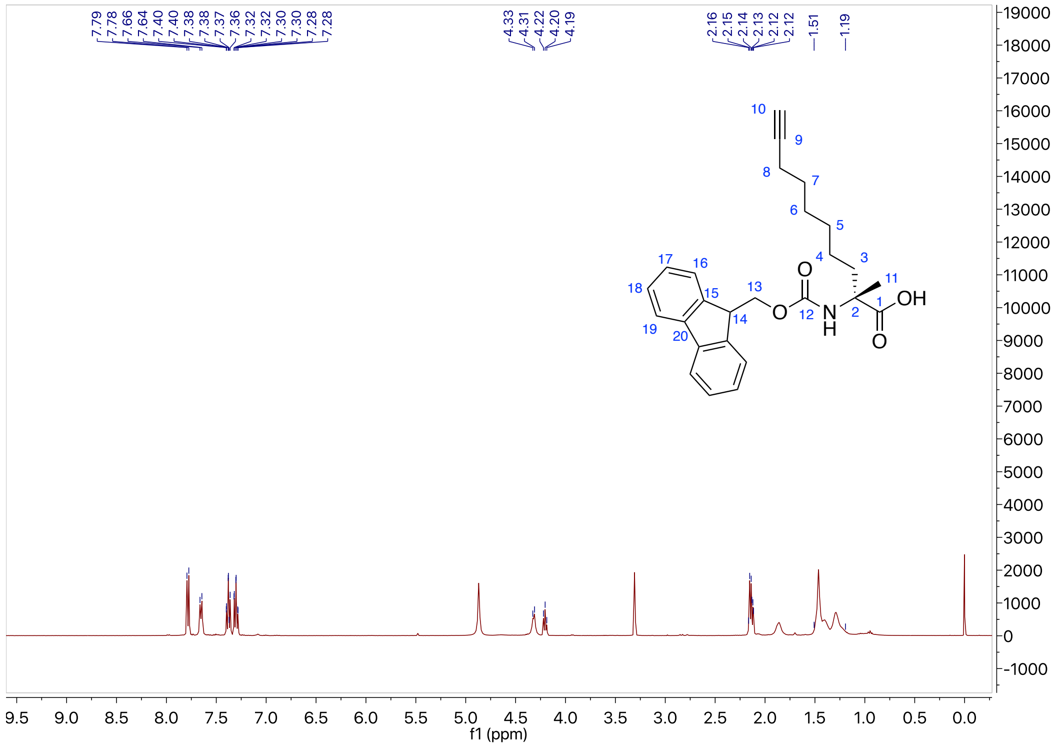

^{13}C NMR

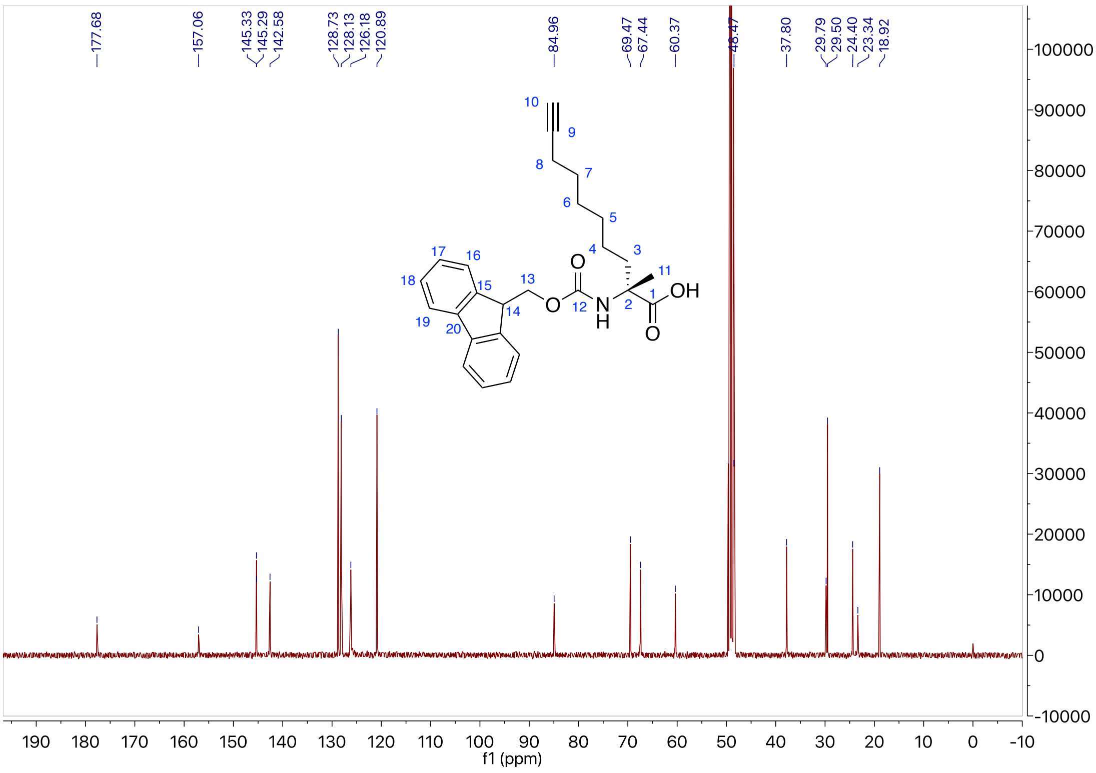
177.68
157.06
145.33
145.29
142.58
128.73
128.13
126.18
120.89
84.96
69.47
67.44
60.37
48.47
37.80
29.79
29.50
24.40
23.34
18.92
10
9
8
7
6
5
4
3
11
1
OH
O
N
H
2
12
O
13
14
15
16
17
18
19
20
f1 (ppm)

Peptide Characterisation Data

Peptide Characterisation Table

Peptide Sequence	Purity by RP-HPLC (%)	Yield (%)	t_R (20 min, 50 min gradient)	Calculated m/z	Measured m/z	Err (ppm)
**26 (T-STAR)**<br>FTU-Ahx-AKAYAR(Nle)GRALKEIAK-NH_2	99	10	14.2, 27.3	$[M+3H]^{3+}$ = 754.0735	$[M+3H]^{3+}$ = 754.0709	-3.5
**27 (T-STAR-diyne-S_5S_5)**<br>FTU-Ahx-AKS_5YAR(Nle)GRS_5LKEIAK-NH_2	98	2	15.1, 29.7	$[M+3H]^{3+}$ = 797.4329	$[M+3H]^{3+}$ = 797.4344	1.8
**28 (T-STAR-diyne-S_6S_6)**<br>FTU-Ahx-AKS_6YAR(Nle)GRS_6LKEIAK-NH_2	95	3	14.6, 28.4	$[M+3H]^{3+}$ = 806.7767	$[M+3H]^{3+}$ = 806.7757	-1.3

**29 (T-STAR-diyne-*S*$_7$*S*$_7$)**<br><br>FTU-Ahx-AK*S*$_7$YAR(Nle)GR*S*$_7$LKEIAK-NH_2	99	6	15.5, 30.7	$[M+3H]^{3+}$ = 816.1204	$[M+3H]^{3+}$ = 816.1221	2.1
**30 (T-STAR-diyne-*R*$_7$*R*$_7$)**<br><br>FTU-Ahx-AK*R*$_7$YAR(Nle)GR*R*$_7$LKEIAK-NH_2	96	4	15.9, 31.5	$[M+4H]^{4+}$ = 619.3422	$[M+4H]^{4+}$ = 619.3434	2.1
**31 (T-STAR-diyne-*R*$_7$*S*$_7$)**<br><br>FTU-Ahx-AK*R*$_7$YAR(Nle)GR*S*$_7$LKEIAK-NH_2	98	4	15.9, 31.4	$[M+4H]^{4+}$ = 612.3422	$[M+4H]^{4+}$ = 612.3444	3.7
**32 (T-STAR-diyne-*S*$_7$*R*$_7$)**<br><br>FTU-Ahx-AK*S*$_7$YAR(Nle)GR*S*$_7$LKEIAK-NH_2	99	2	14.9, 29.2	$[M+3H]^{3+}$ = 816.1204	$[M+3H]^{3+}$ = 816.1229	2.9
**33 (T-STAR-diyne-*S*$_8$*S*$_8$)**<br><br>FTU-Ahx-AK*S*$_8$YAR(Nle)GR*S*$_8$LKEIAK-NH_2	95	5	15.4, 30.3	$[M+4H]^{4+}$ = 619.3500	$[M+4H]^{4+}$ = 619.3525	4.1

<table>
<tr><td>34 (T-STAR-diyne-R_8R_8)

FTU-Ahx-AKR_8YAR(Nle)GRR_8LKEIAK-NH_2</td><td>96</td><td>3</td><td>16.3, 32.6</td><td>$[M+4H]^{4+}$ =
619.3500</td><td>$[M+4H]^{4+}$ =
619.3525</td><td>4.0</td></tr>
<tr><td>35 (T-STAR-diyne-R_8S_8)

FTU-Ahx-AKR_8YAR(Nle)GRS_8LKEIAK-NH_2</td><td>97</td><td>5</td><td>16.2, 32.2</td><td>$[M+4H]^{4+}$ =
619.3500</td><td>$[M+4H]^{4+}$ =
619.3511</td><td>1.8</td></tr>
<tr><td>36 (T-STAR-diyne-S_8R_8)

FTU-Ahx-AKS_8YAR(Nle)GRR_8LKEIAK-NH_2</td><td>97</td><td>3</td><td>15.7, 31.0</td><td>$[M+3H]^{3+}$ =
825.4642</td><td>$[M+3H]^{3+}$ =
825.4680</td><td>4.6</td></tr>
<tr><td>37 (T-STAR-HCS-2-cis)

FTU-Ahx-AKX_8YAR(Nle)GRX_5LKEIAK-NH_2</td><td>95</td><td rowspan="2">5</td><td>16.1, 32.1</td><td>$[M+3H]^{3+}$ =
804.1204</td><td>$[M+3H]^{3+}$ =
804.1186</td><td>-2.3</td></tr>
<tr><td>38 (T-STAR-HCS-2-trans)

FTU-Ahx-AKX_8YAR(Nle)GRX_5LKEIAK-NH_2</td><td>96</td><td>16.3, 32.6</td><td>$[M+3H]^{3+}$ =
804.1204</td><td>$[M+3H]^{3+}$ =
804.1168</td><td>-4.5</td></tr>
</table>

**39 (T-STAR-diyne-*S*$_{7H}$*S*$_{7H}$)** FTU-Ahx-AK*S*$_{7H}$YAR(Nle)GR*S*$_{7H}$LKEIAK-NH_2	98	2	16.9, 31.0	$[M+3H]^{3+}$ = 806.7767	$[M+3H]^{3+}$ = 806.7734	-4.1
**40 (Acetylated T-STAR)** Ac-AKAYAR(Nle)GRALKEIAK-NH_2	99	12	13.0, 24.2	$[M+3H]^{3+}$ = 601.0380	$[M+3H]^{3+}$ = 601.0385	0.82
**41 (Acetylated T-STAR-diyne-*S*$_7$*S*$_7$)** Ac-AK*S*$_7$YAR(Nle)GR*S*$_7$LKEIAK-NH_2	98	3	15.1, 29.6	$[M+3H]^{3+}$ = 663.0850	$[M+3H]^{3+}$ = 663.0844	-0.82

Table S1: Peptide sequence, % purity, % yield, m/z and retention time of peptides. Abbreviations: FTU = Fluorescein thiourea, Ahx = Aminohexanoic acid, Ac = Acetylated, NH_2 = *C*-terminal amide, Nle = norleucine, S_5 = (S)-2-((((9H-fluoren-9-yl)methoxy)carbonyl)amino)-2-methylhept-6-ynoic acid, S_6 = (S)-2-((((9H-fluoren-9-yl)methoxy)carbonyl)amino)-2-methyloct-7-ynoic acid, S_7 = (S)-2-((((9H-Fluoren-9-yl)methoxy)carbonyl)amino)-2-methylnon-8-ynoic acid, S_8 = (S)-2-((((9H-Fluoren-9-yl)methoxy)carbonyl)amino)-2-methyldec-9-ynoic acid, R_5 = (R)-2-((((9H-fluoren-9-yl)methoxy)carbonyl)amino)-2-methylhept-6-ynoic acid, R_6 = (R)-2-((((9H-fluoren-9-yl)methoxy)carbonyl)amino)-2-methyloct-7-ynoic acid, R_7 = (R)-2-((((9H-Fluoren-9-yl)methoxy)carbonyl)amino)-2-methylnon-8-ynoic acid, R_8 = ((R)-2-((((9H-Fluoren-9-yl)methoxy)carbonyl)amino)-2-methyldec-9-ynoic acid**,** S_{7H} = (S)-2-((((9H-Fluoren-9-yl)methoxy)carbonyl)amino)-non-8-ynoic acid, X_8 = (R)-2-((((9H-Fluoren-9-yl)methoxy)carbonyl)amino)-(7-Octenyl)alanine, X_5 = (S)-2-((((9H-Fluoren-9-yl)methoxy)carbonyl)amino)-α-(4-pentenyl)alanine.

26 (Native T-STAR)

FTU-Ahx-AKAYAR(Nle)GRALKEIAK-NH_2

20 min HPLC

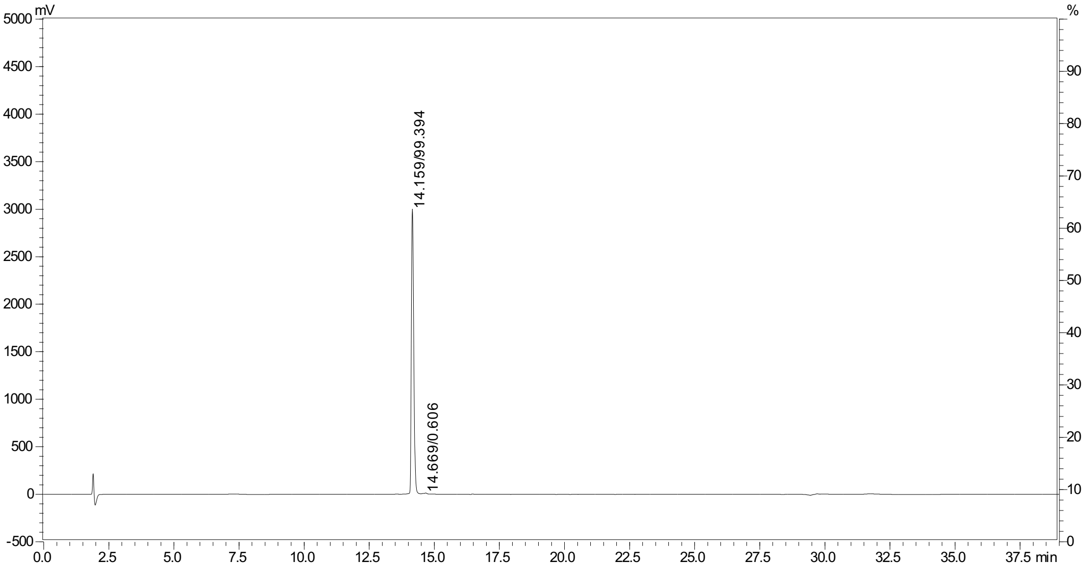

50 min HPLC

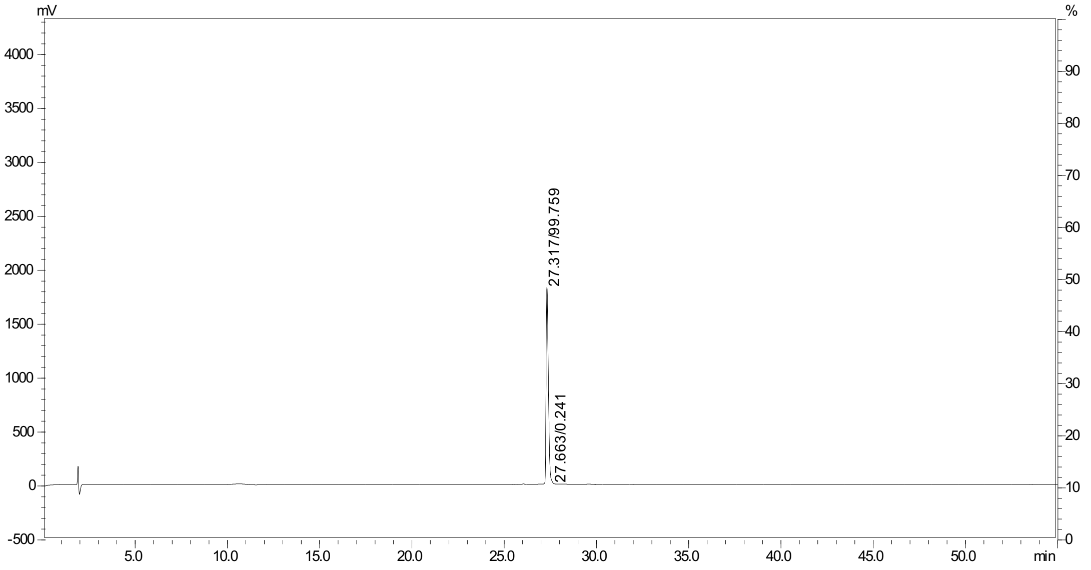

HR-MS

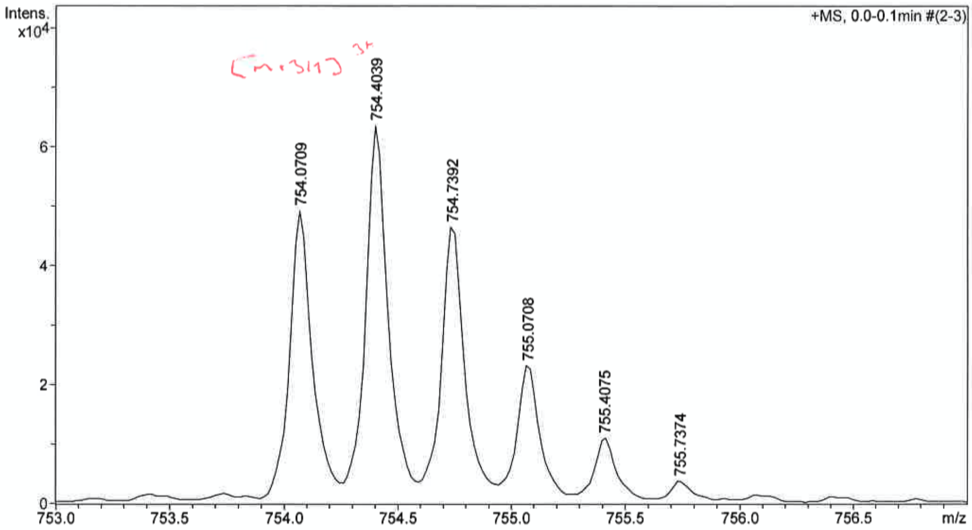

Formula	z	m/z	Meas. m/z	err [ppm]	err [mDa]
	3+		754.0709		
C 106 H 165 N 28 O 25 S		754.0735		3.5	2.6

27 (T-STAR-diyne-S_5S_5)

FTU-Ahx-AKS_5YAR(Nle)GRS_5LKEIAK-NH_2

20 min HPLC

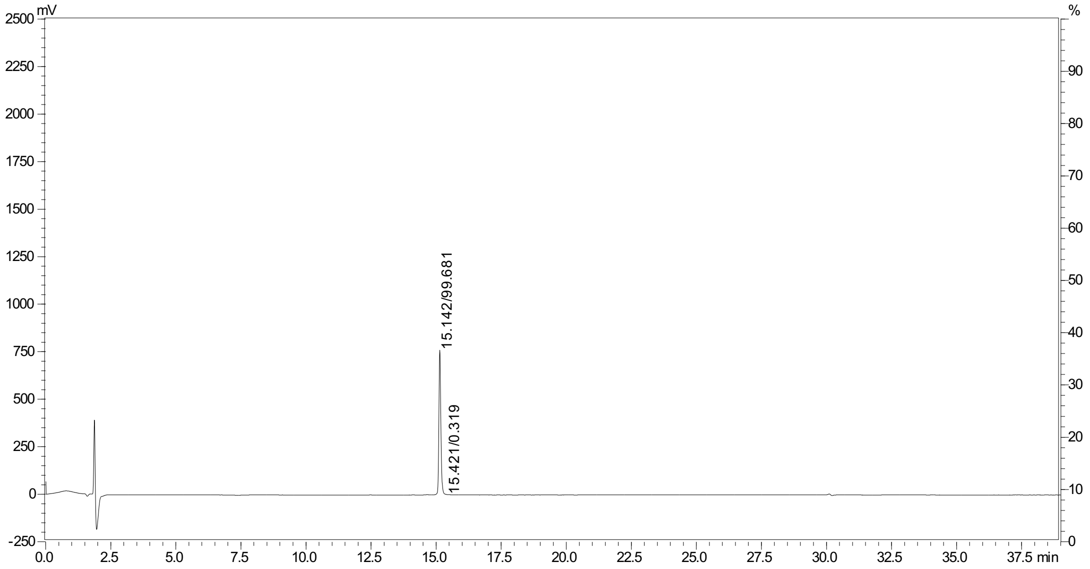

50 min HPLC

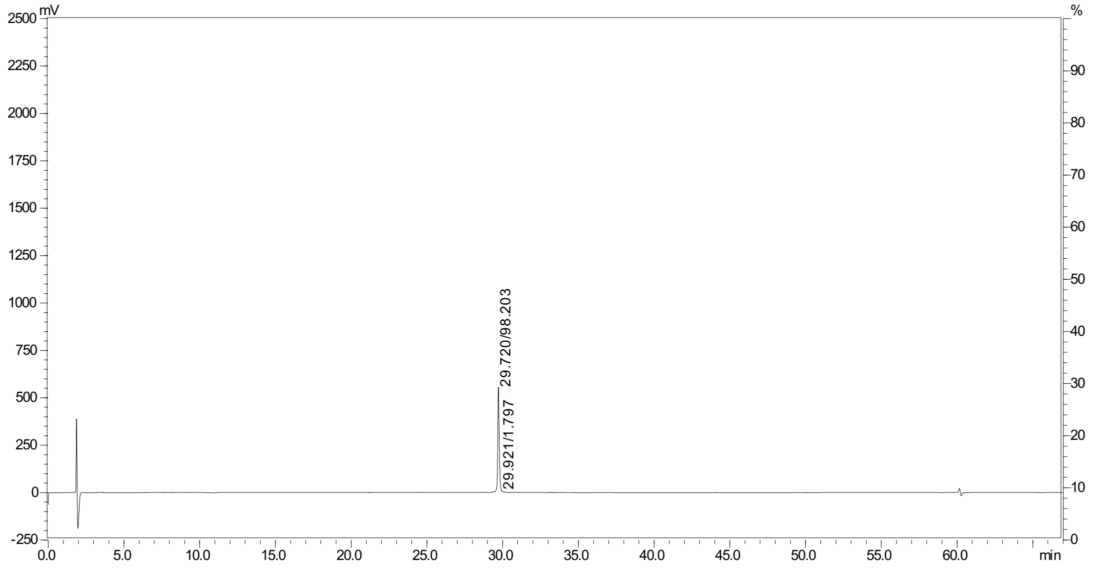

HR-MS

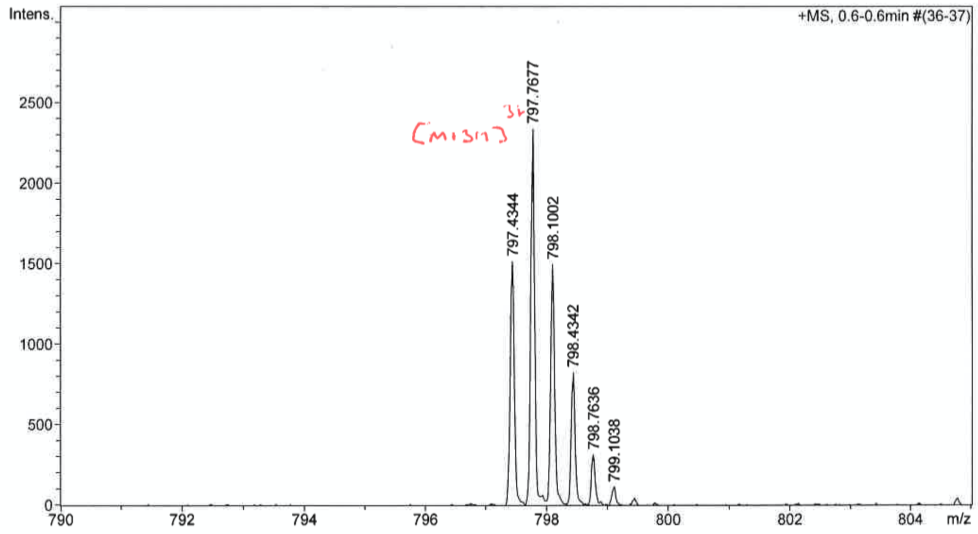

Formula	z	m/z	Meas. m/z	err [ppm]	err [mDa]
C 116 H 175 N 28 O 25 S	3+	797.4329	797.4344	-1.8	-1.4

28 (T-STAR-diyne-S_6S_6)

FTU-Ahx-AKS_6YAR(Nle)GRS_6LKEIAK-NH_2

20 min HPLC

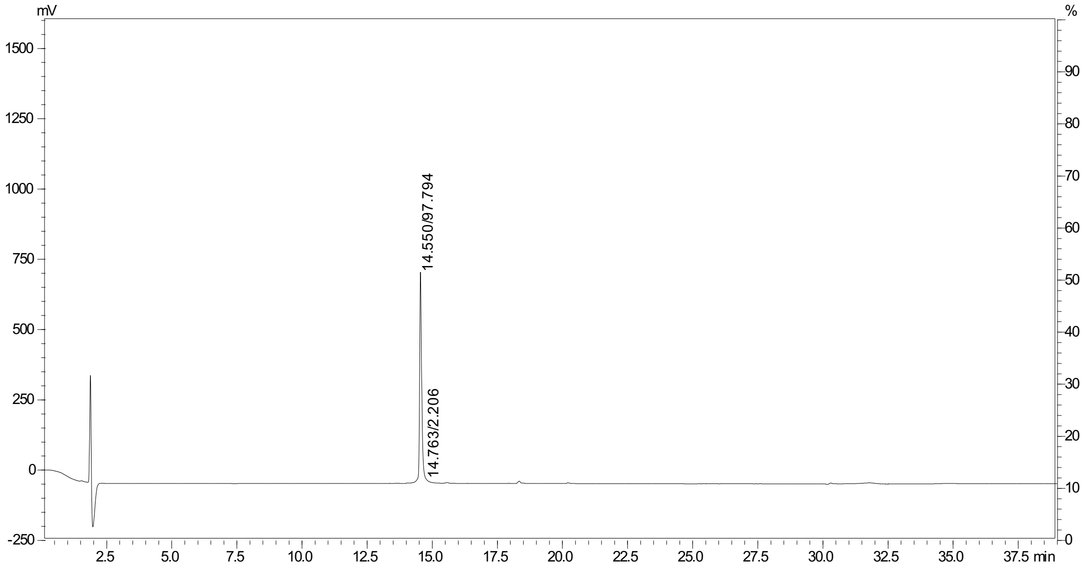

50 min HPLC

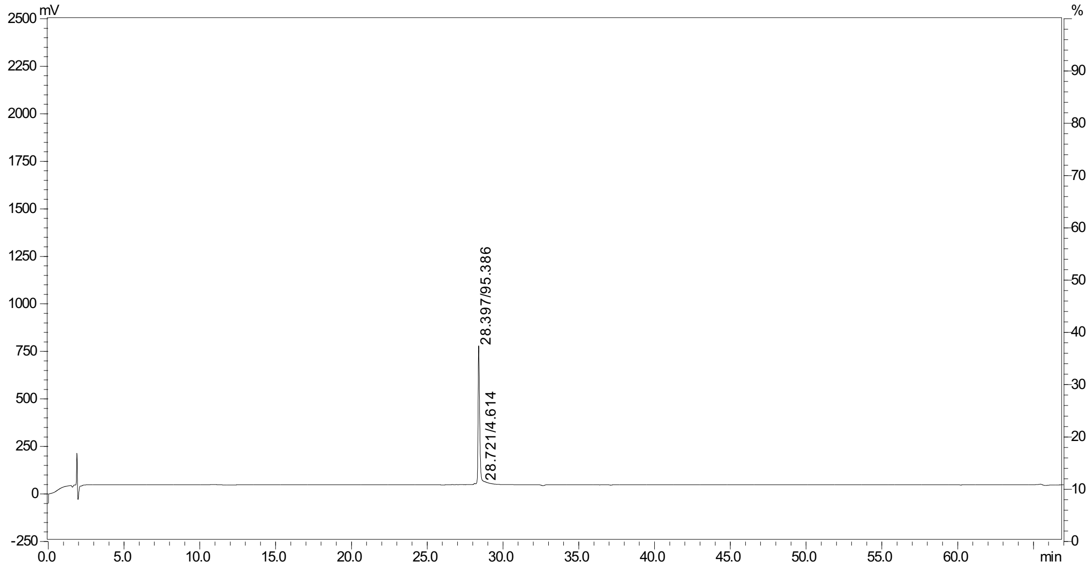

HR-MS

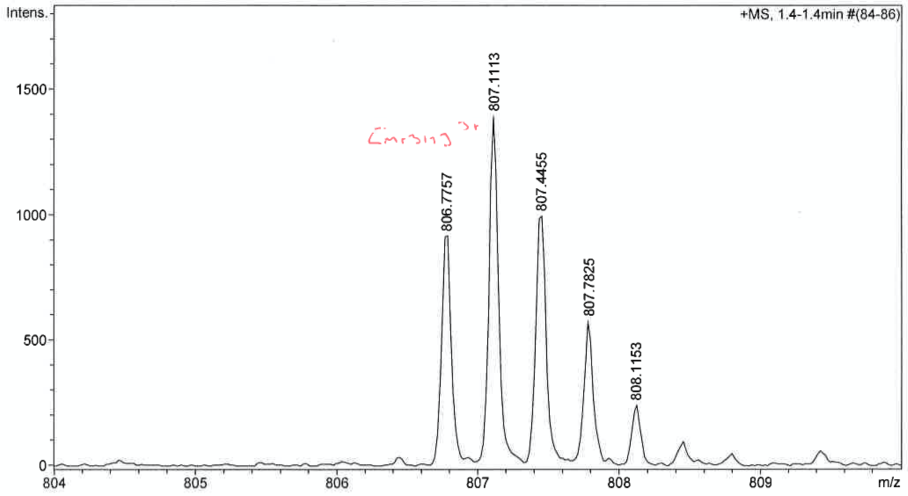

Formula	z	m/z	Meas. m/z	err [ppm]	err [mDa]
C 118 H 179 N 28 O 25 S	3+	806.7767	806.7757	1.3	1.0

29 (T-STAR-diyne-S_7S_7)

FTU-Ahx-AKS_7YAR(Nle)GRS_7LKEIAK-NH_2

20 min HPLC

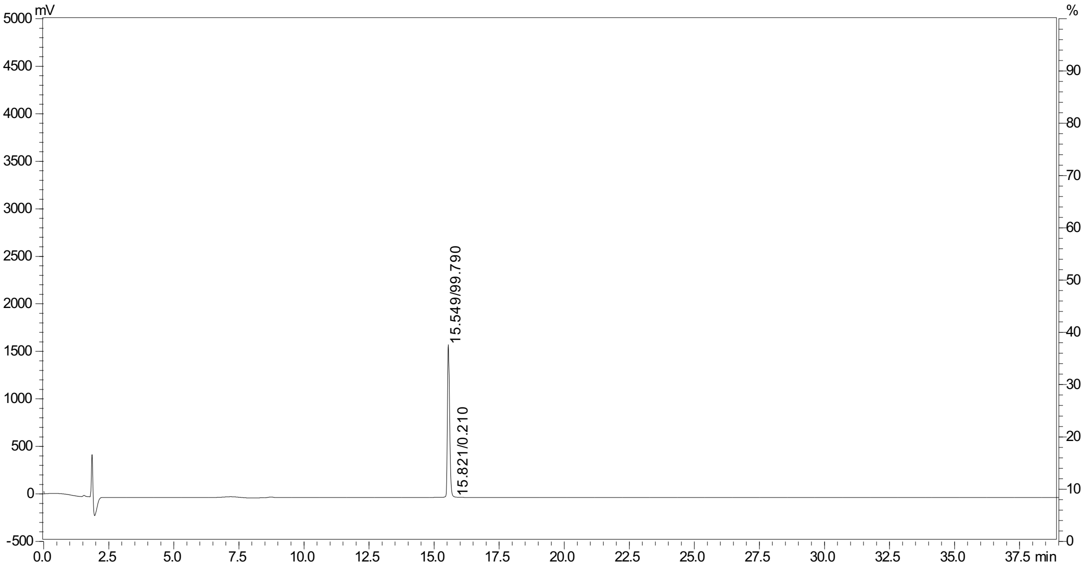

50 min HPLC

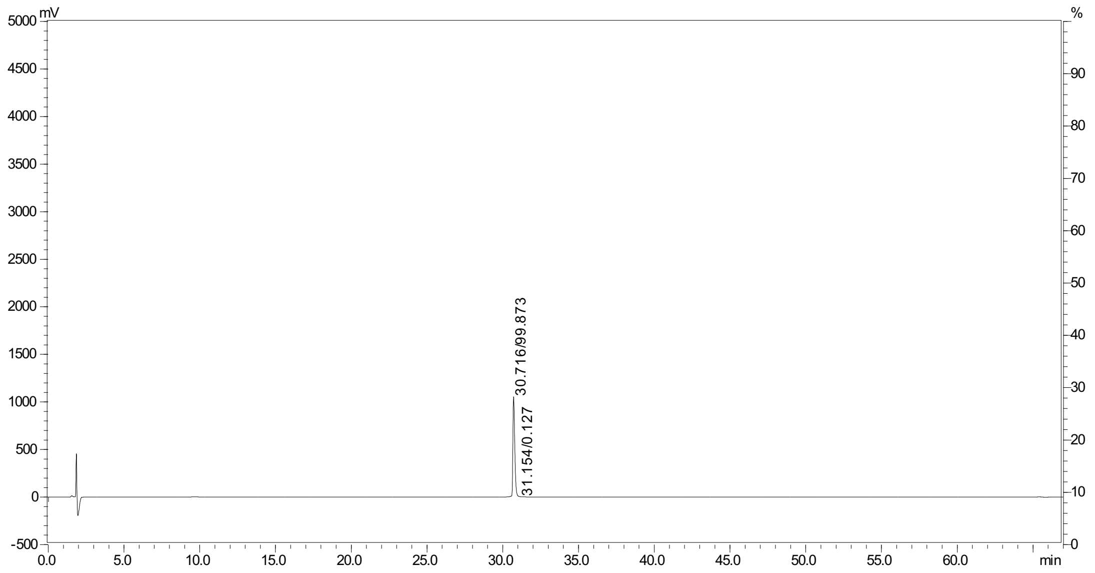

HR-MS

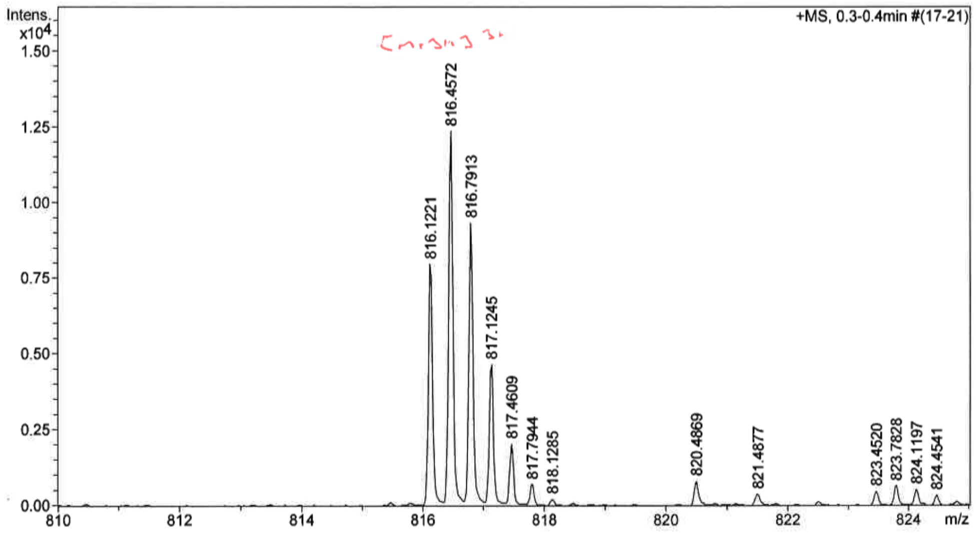

Formula	z	m/z	Meas. m/z	err [ppm]	err [mDa]
C 120 H 183 N 28 O 25 S	3+	816.1204	816.1221	-2.1	-1.7

30 (T-STAR-diyne-*R*$_7$*R*$_7$)

FTU-Ahx-AK*R*$_7$YAR(Nle)GR*R*$_7$LKEIAK-NH$_2$

20 min HPLC

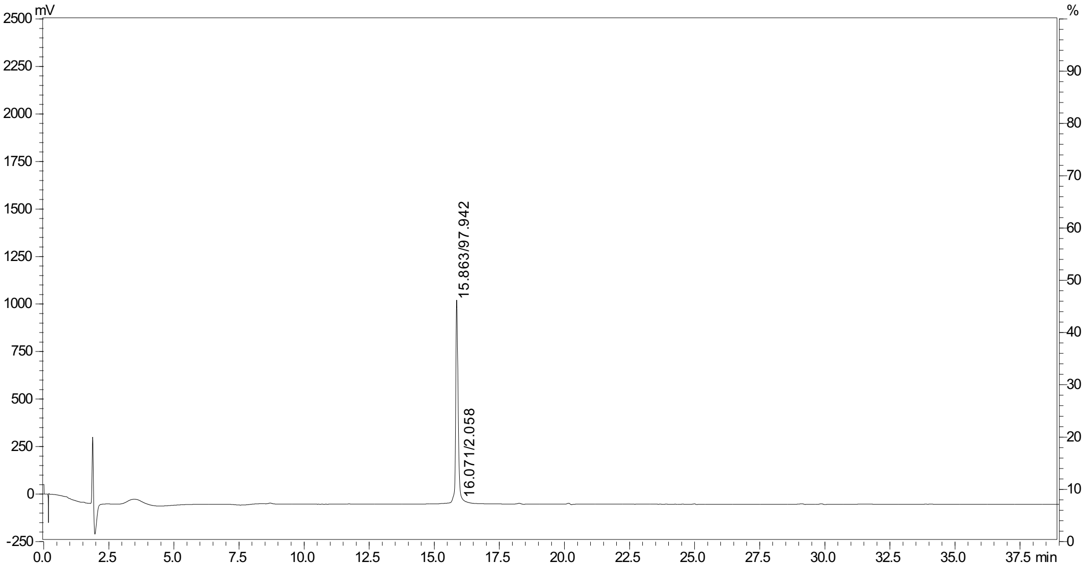

50 min HPLC

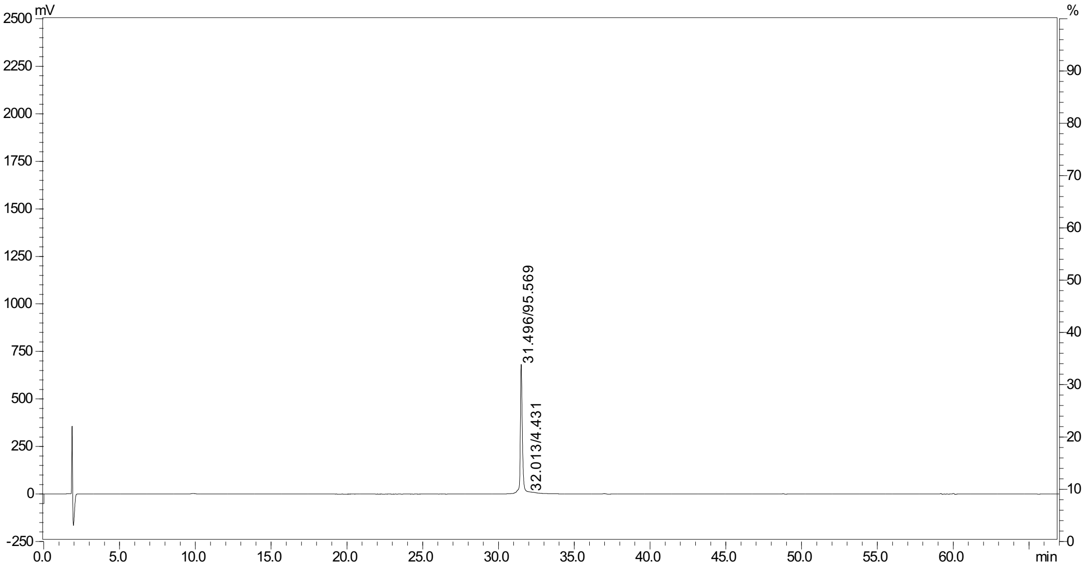

HR-MS

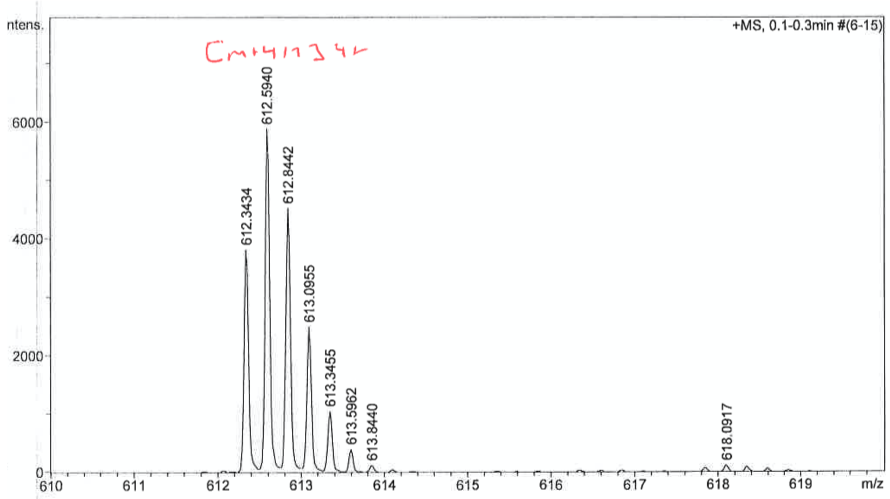

Formula	z	m/z	Meas. m/z	err [ppm]	err [mDa]
C 120 H 184 N 28 O 25 S	4+	612.3422	612.3434	-2.1	-1.3

31 (T-STAR-diyne-R_7S_7)

FTU-Ahx-AKR_7YAR(Nle)GRS_7LKEIAK-NH_2

20 min HPLC

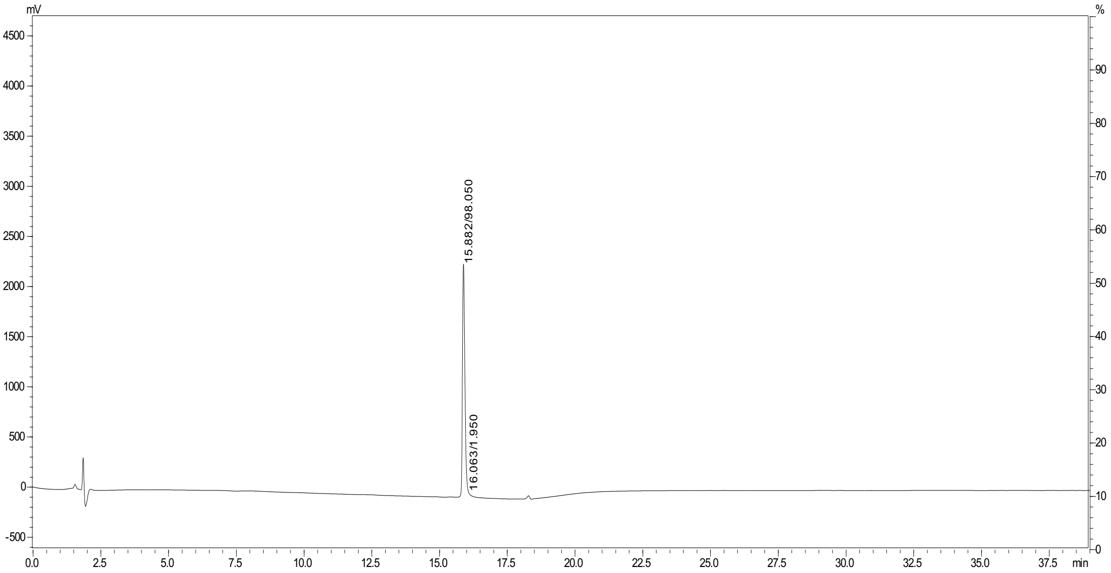

50 min HPLC

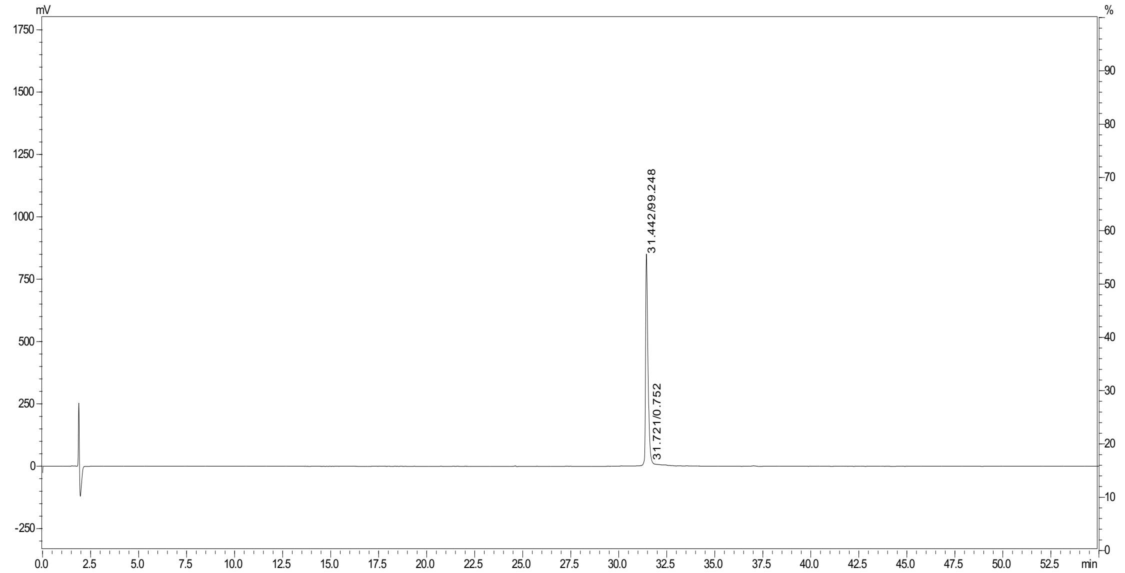

HR-MS

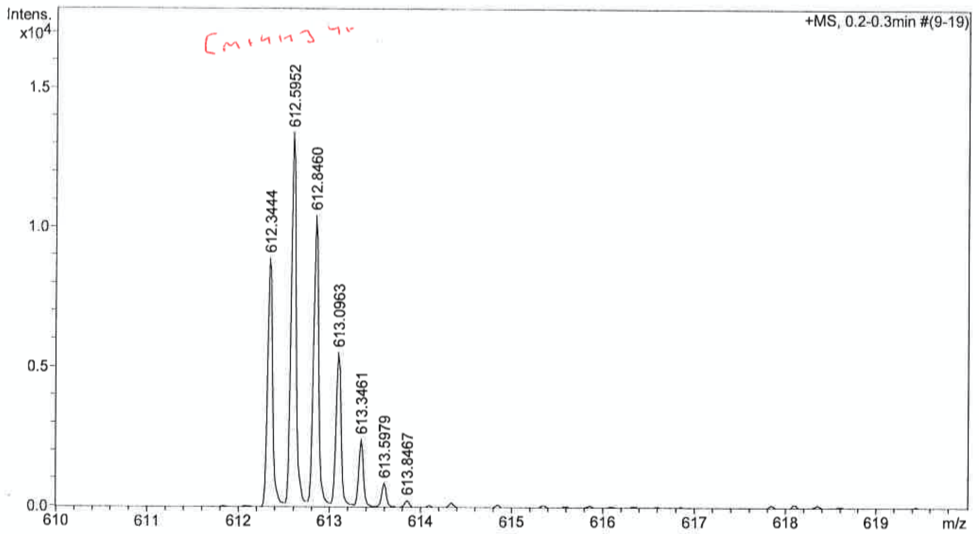

Formula	z	m/z	Meas. m/z	err [ppm]	err [mDa]
C 120 H 184 N 28 O 25 S	4+	612.3422	612.3444	-3.7	-2.3

32 (T-STAR-diyne-S_7R_7)

FTU-Ahx-AKS_7YAR(Nle)GRR_7LKEIAK-NH$_2$

20 min HPLC

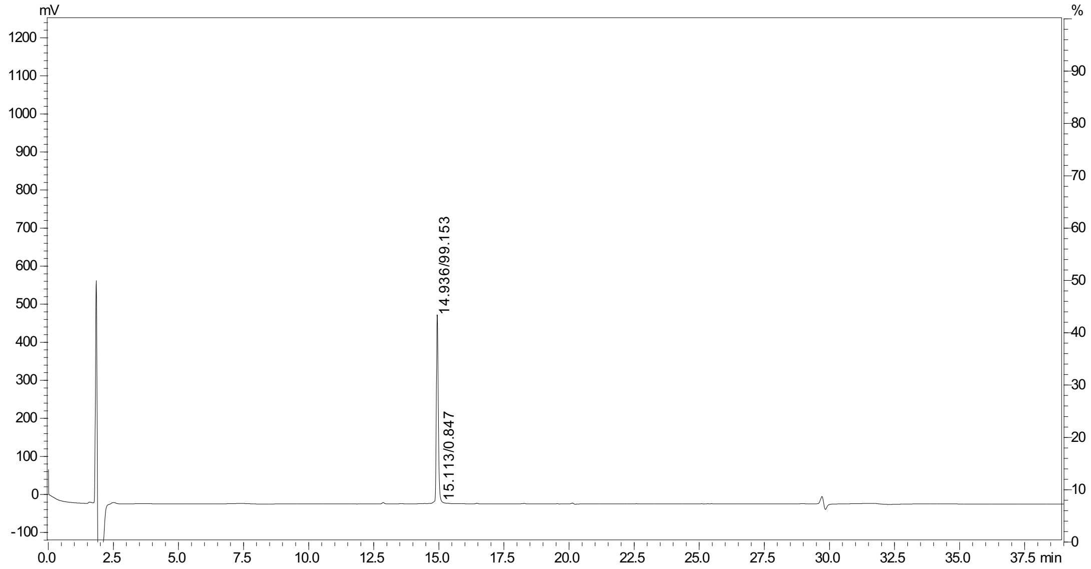

50 min HPLC

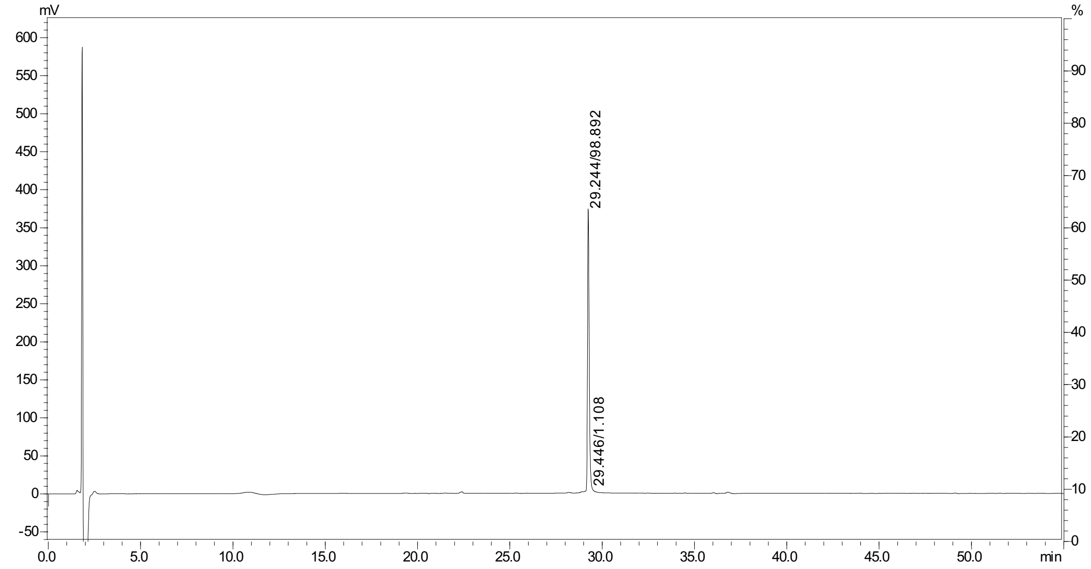

HR-MS

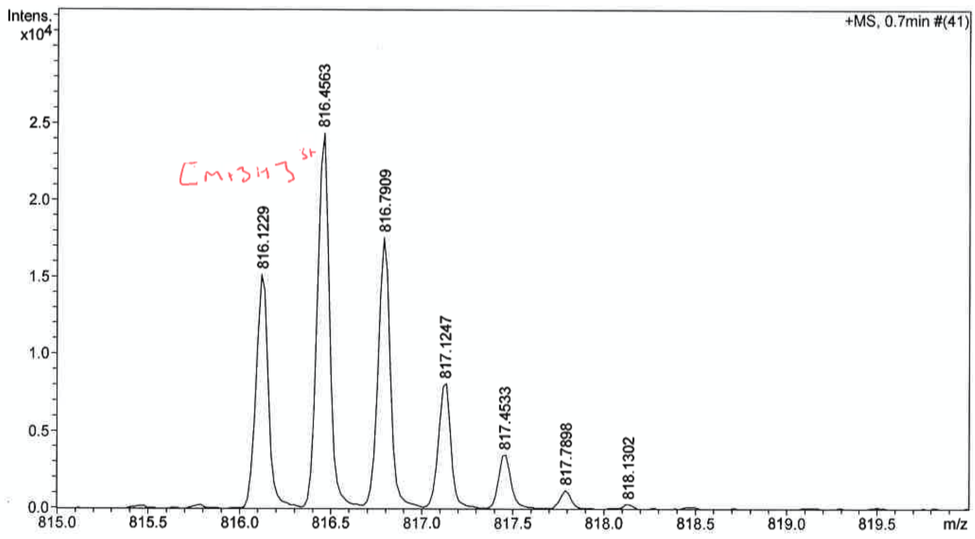

Formula	z	m/z	Meas. m/z	err [ppm]	err [mDa]
C 120 H 183 N 28 O 25 S	3+	816.1204	816.1229	-2.9	-2.4

33 (T-STAR-diyne-S_8S_8)

FTU-Ahx-AKS_8YAR(Nle)GRS_8LKEIAK-NH_2

20 min HPLC

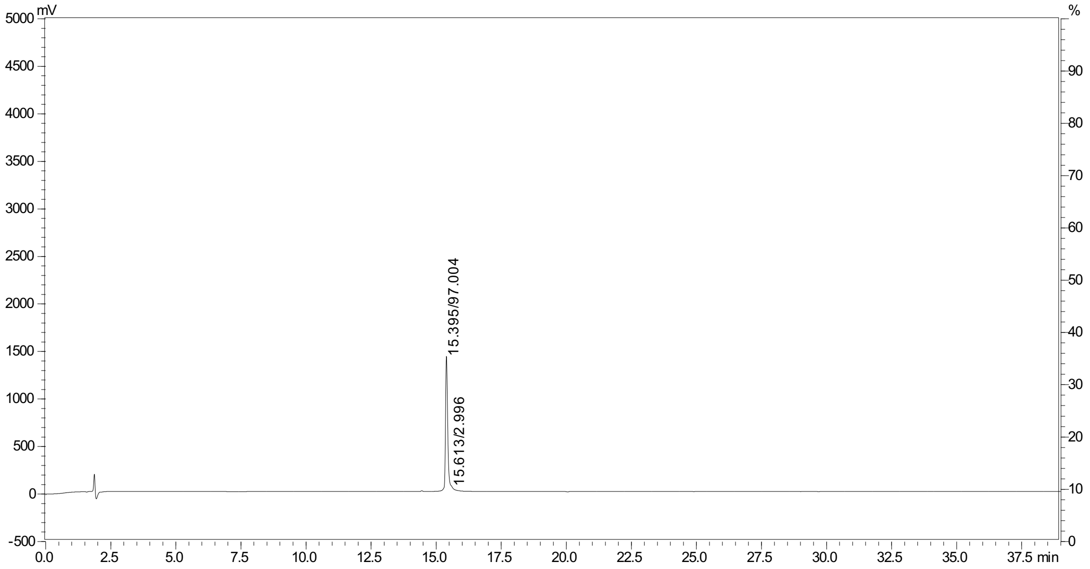

50 min HPLC

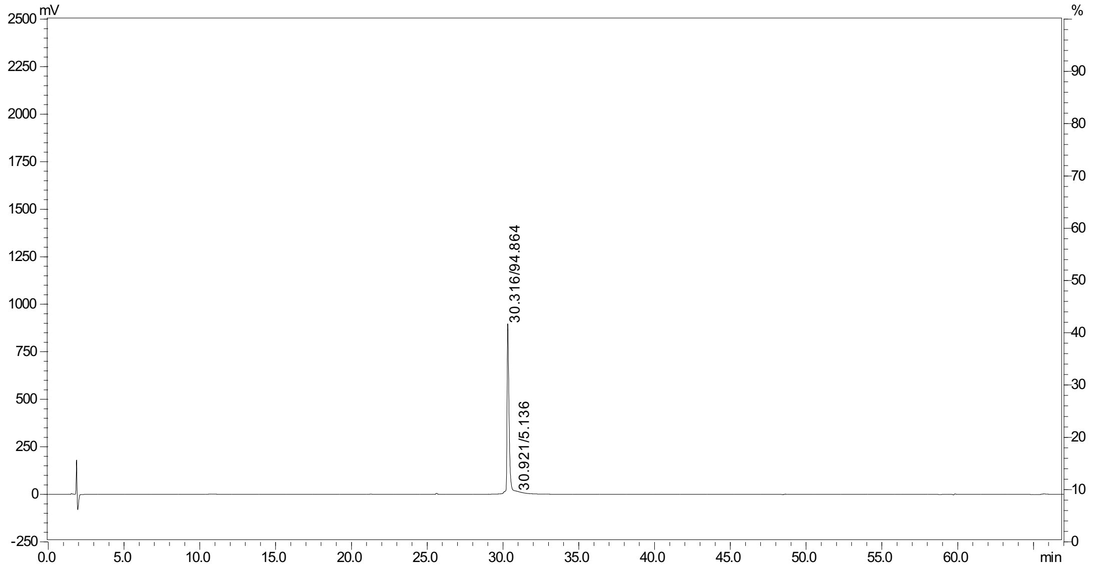

HR-MS

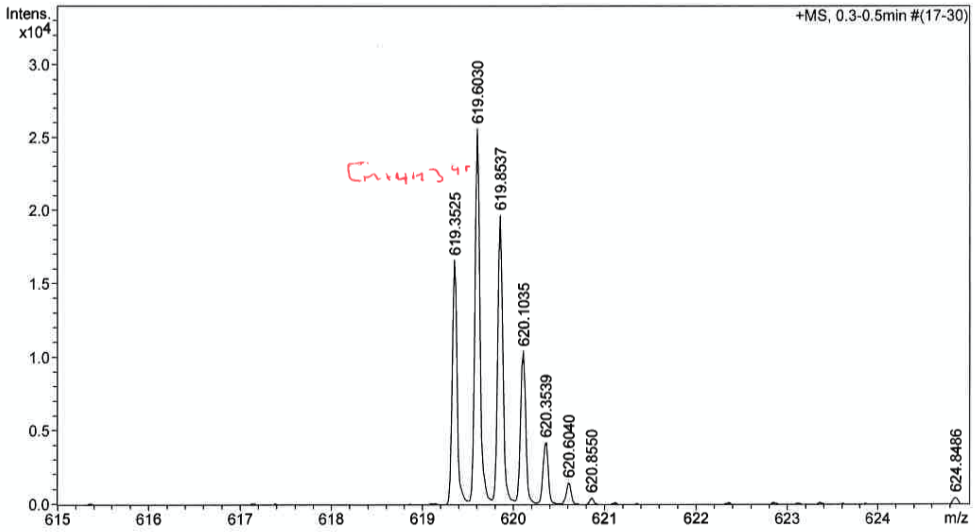

Formula	z	m/z	Meas. m/z	err [ppm]	err [mDa]
	4+		619.3525		
C 122 H 188 N 28 O 25 S		619.3500		-4.1	-2.5

34 (T-STAR-diyne-R_8R_8)

FTU-Ahx-AKR_8YAR(Nle)GRR_8LKEIAK-NH_2

20 min HPLC

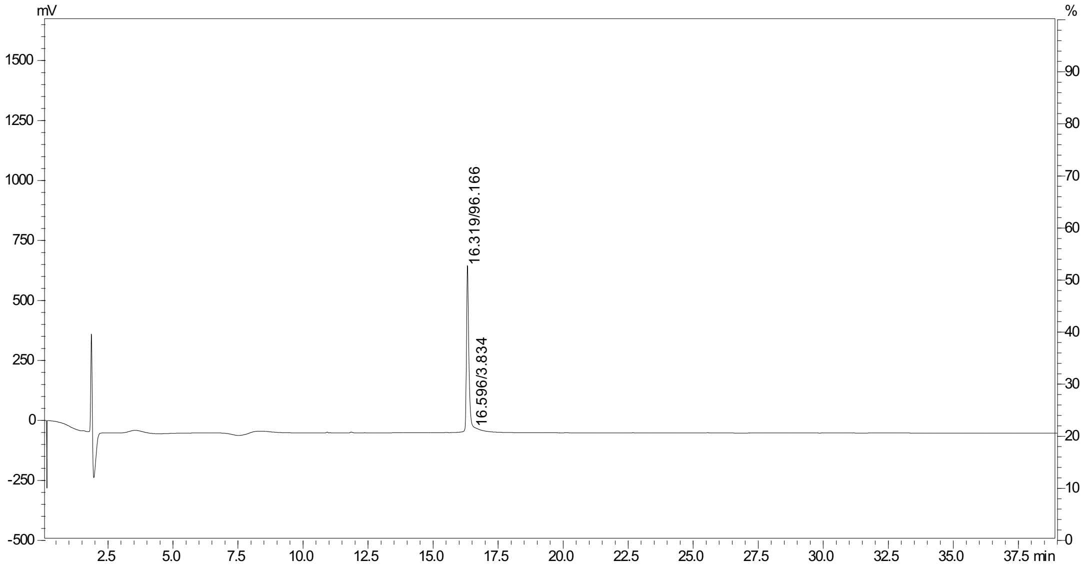

50 min HPLC

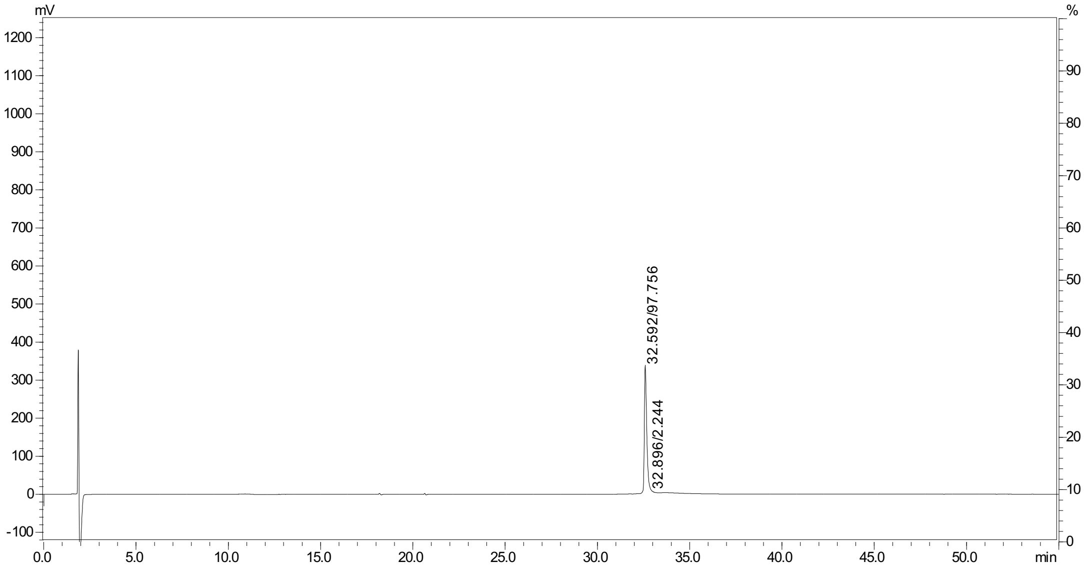

HR-MS

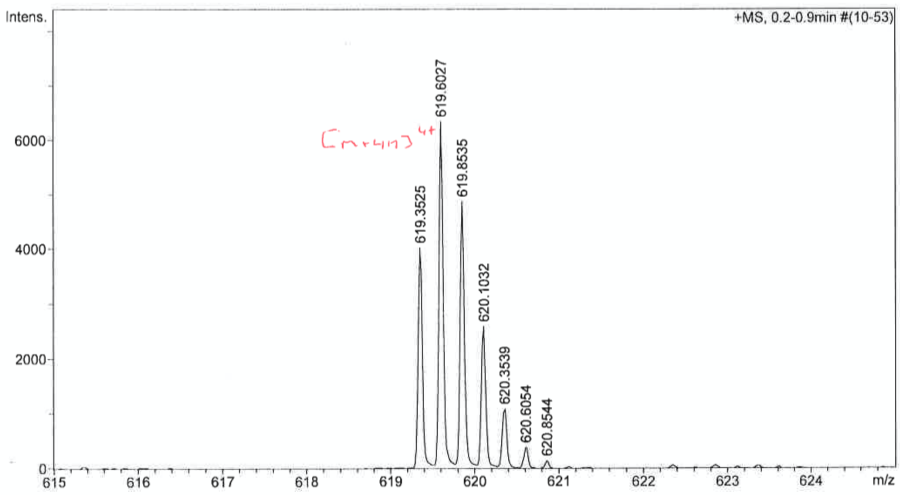

Formula	z	m/z	Meas. m/z	err [ppm]	err [mDa]
C 122 H 188 N 28 O 25 S	4+	619.3500	619.3525	-4.0	-2.5

35 (T-STAR-diyne-*R*$_8$*S*$_8$)

FTU-Ahx-AK*R*$_8$YAR(Nle)GR*S*$_8$LKEIAK-NH_2

20 min HPLC

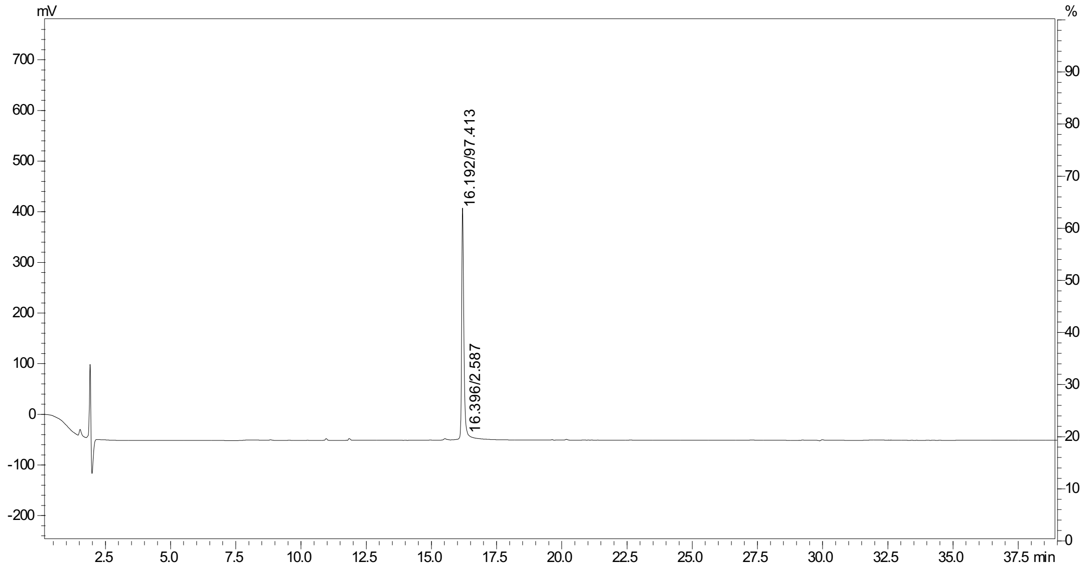

50 min HPLC

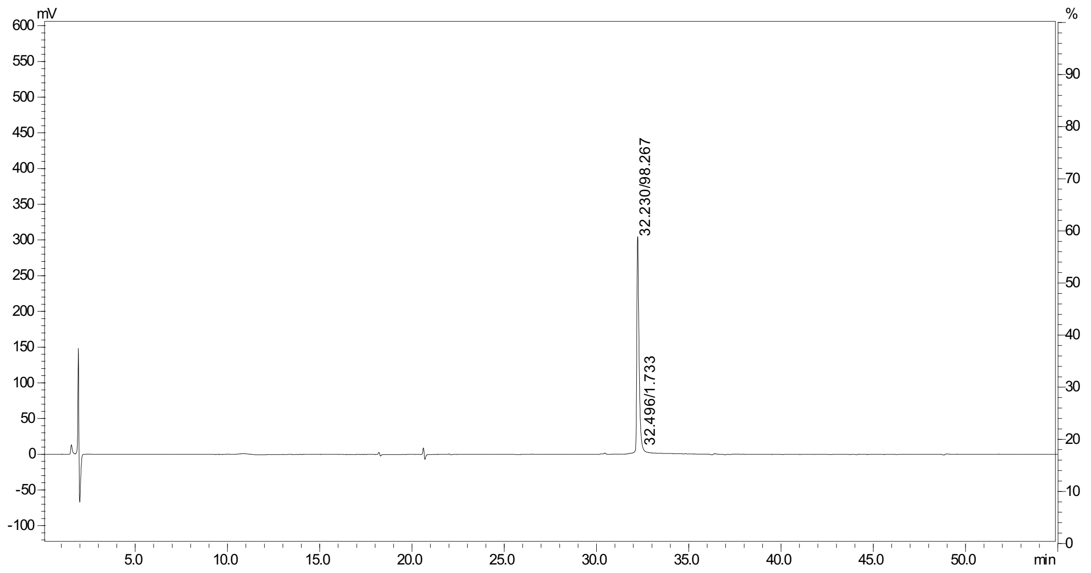

HR-MS

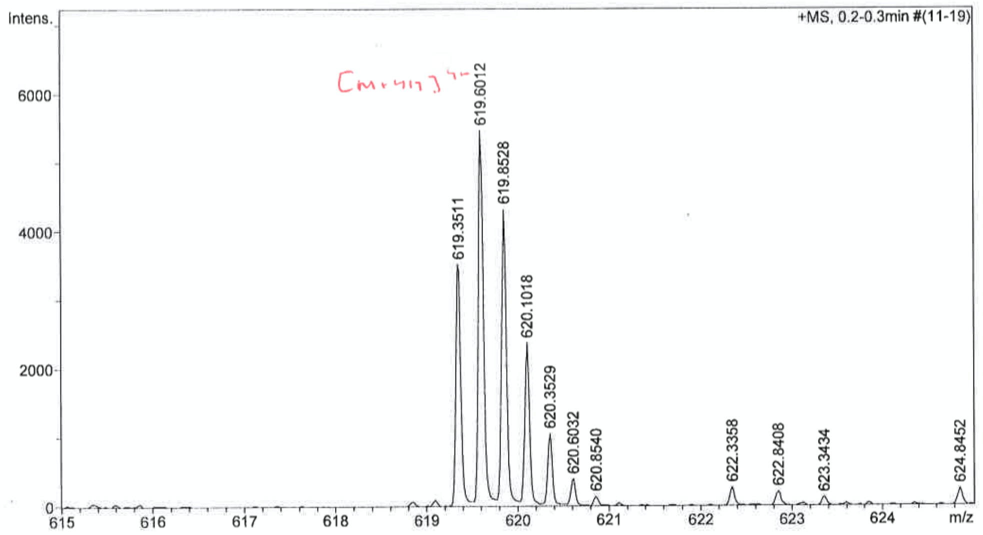

Formula	z	m/z	Meas. m/z	err [ppm]	err [mDa]
C 122 H 188 N 28 O 25 S	4+	619.3500	619.3511	-1.8	-1.1

36 (T-STAR-diyne-S_8R_8)

FTU-Ahx-AKS_8YAR(Nle)GRR_8LKEIAK-NH_2

20 min HPLC

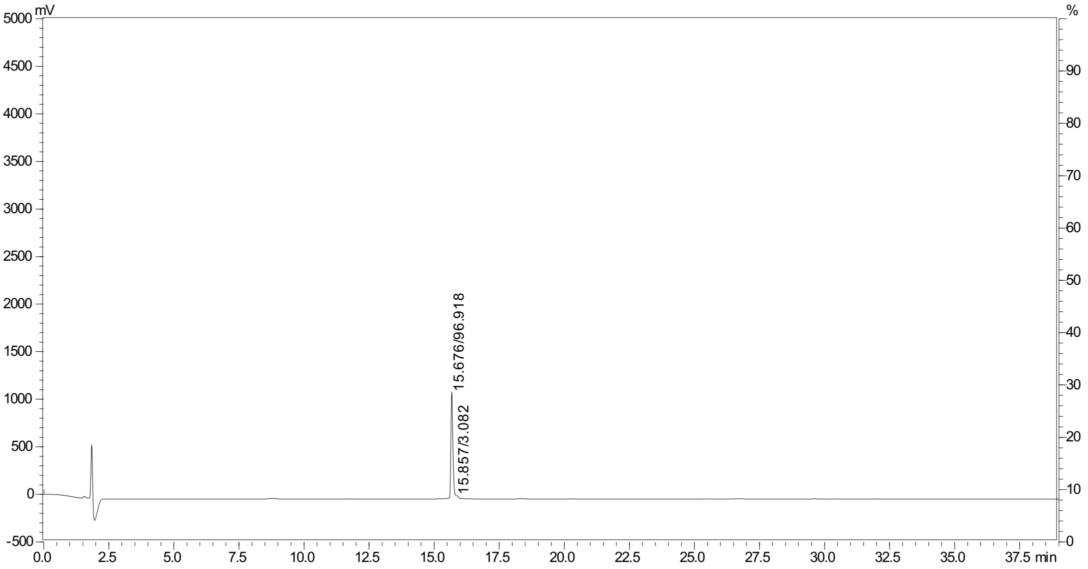

50 min HPLC

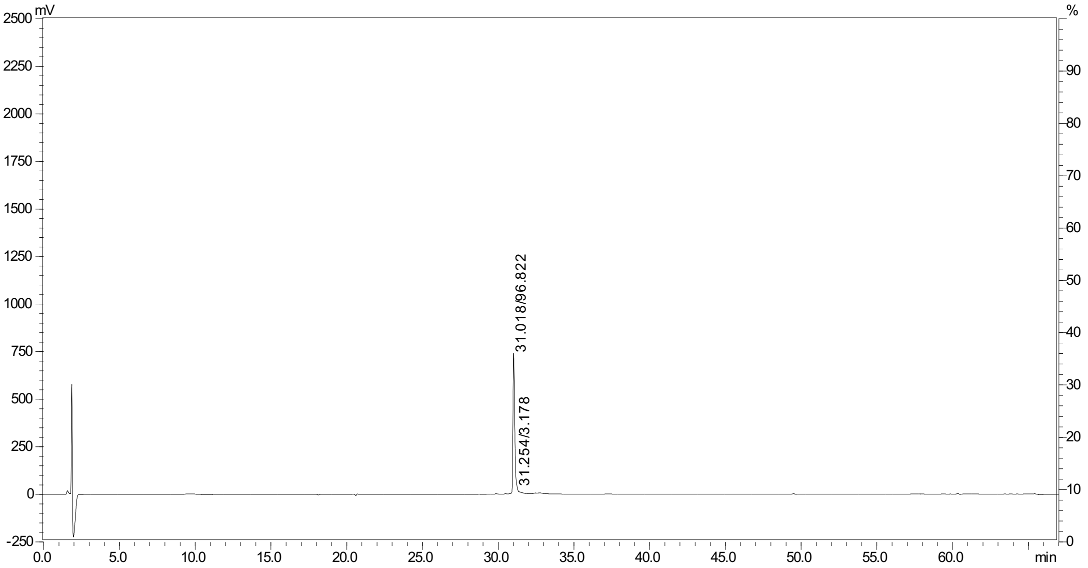

HR-MS

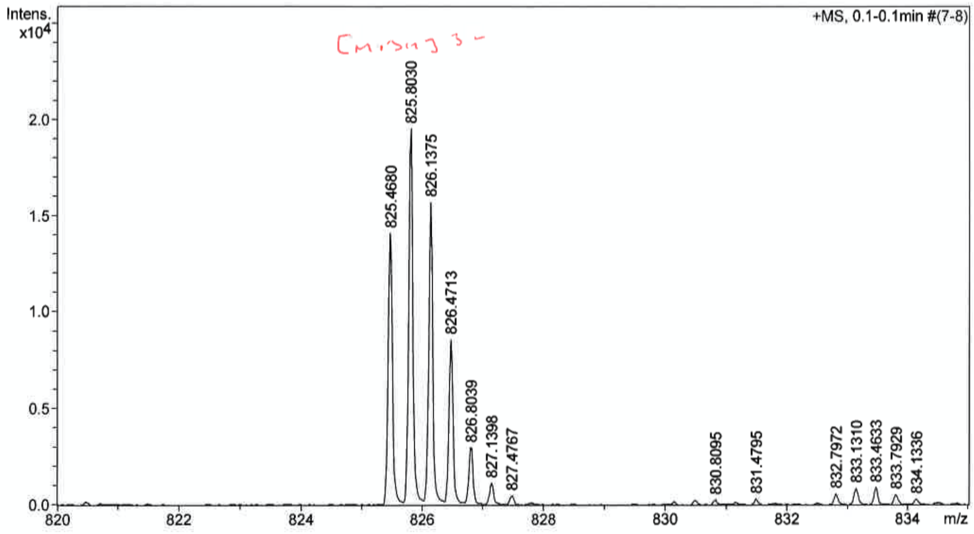

Formula	z	m/z	Meas. m/z	err [ppm]	err [mDa]
	3+		825.4680		
C 122 H 187 N 28 O 25 S		825.4642		-4.6	-3.8

37 (T-STAR-HCS-2-*cis*)

FTU-Ahx-AKR_8YAR(Nle)GRS_5LKEIAK-NH_2

20 min HPLC

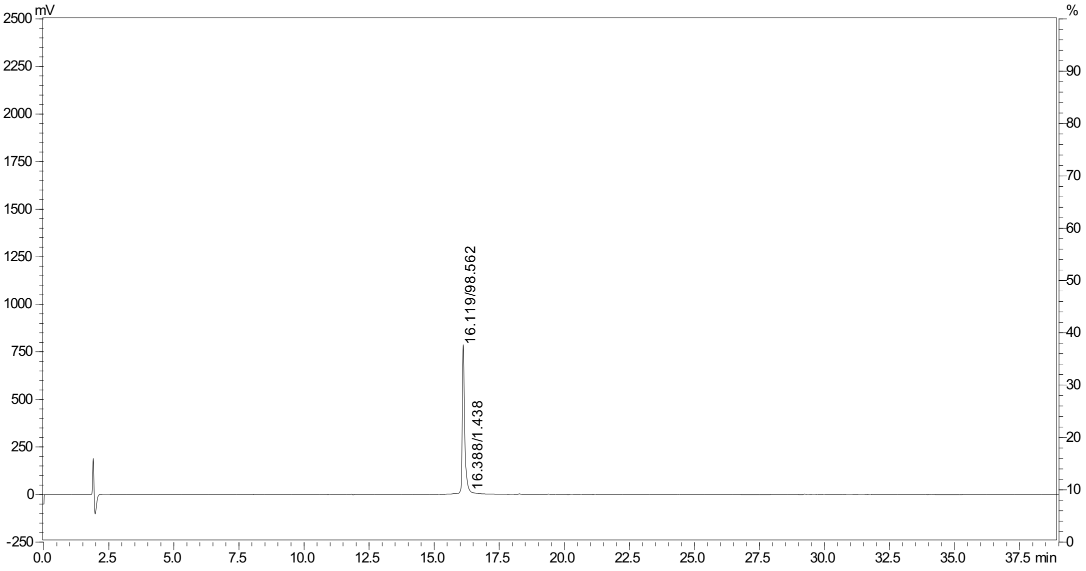

50 min HPLC

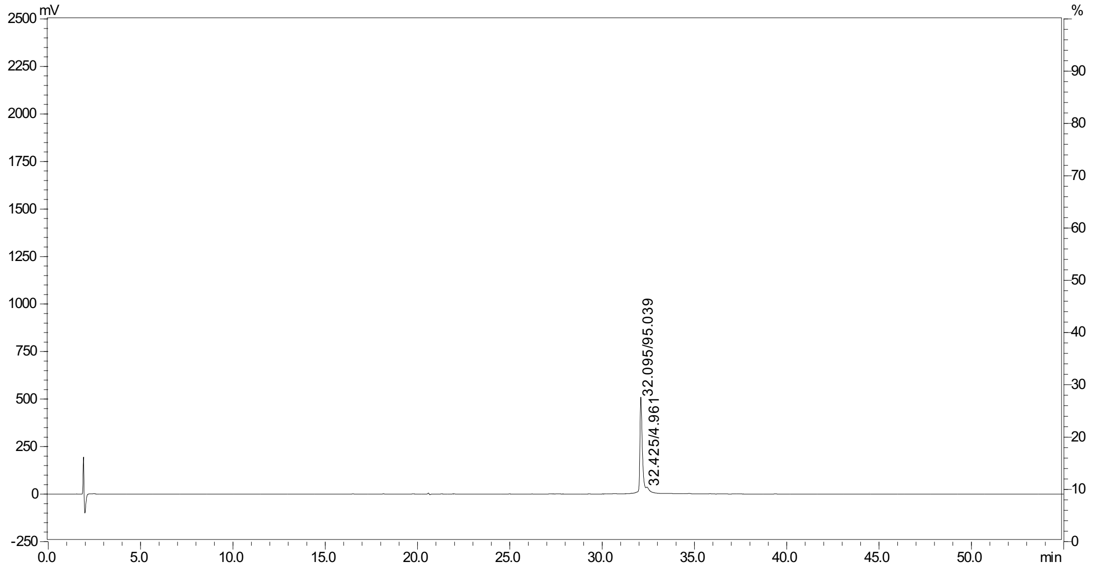

HR-MS

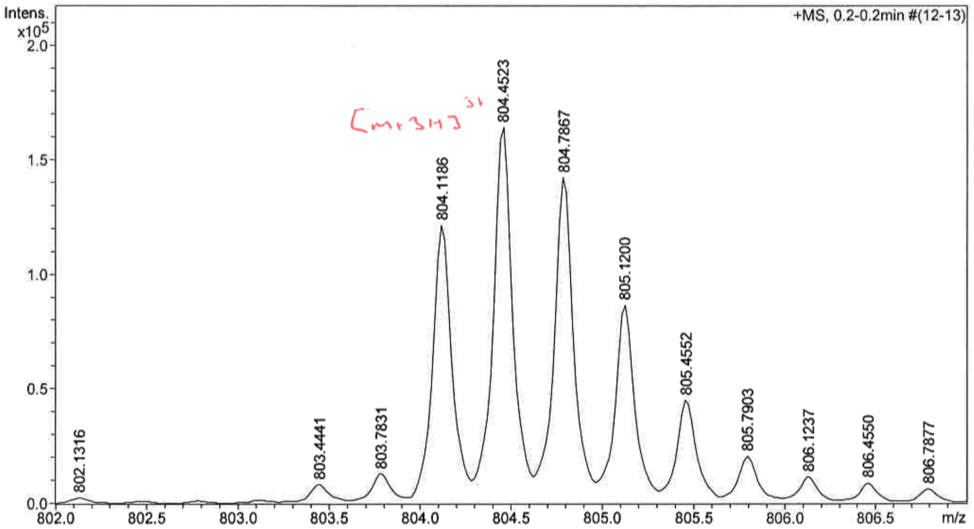

Formula	z	m/z	Meas. m/z	err [ppm]	err [mDa]
	3+		804.1186		
C 117 H 183 N 28 O 25 S		804.1204		2.3	1.8

38 (T-STAR-HCS-2-*trans*)

FTU-Ahx-AK*R*8YAR(Nle)GR*S*5LKEIAK-NH2

20 min HPLC

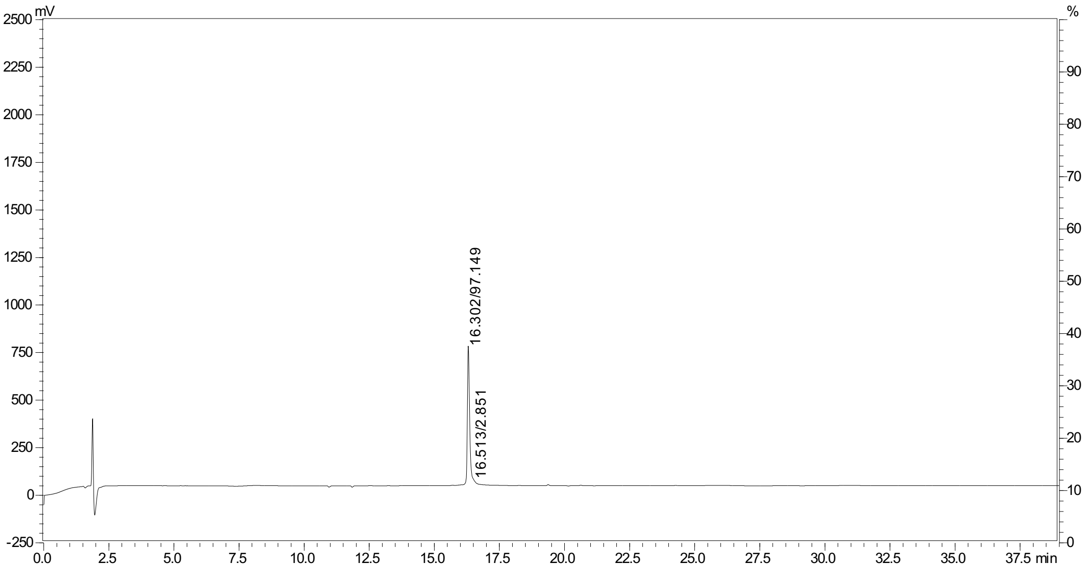

50 min HPLC

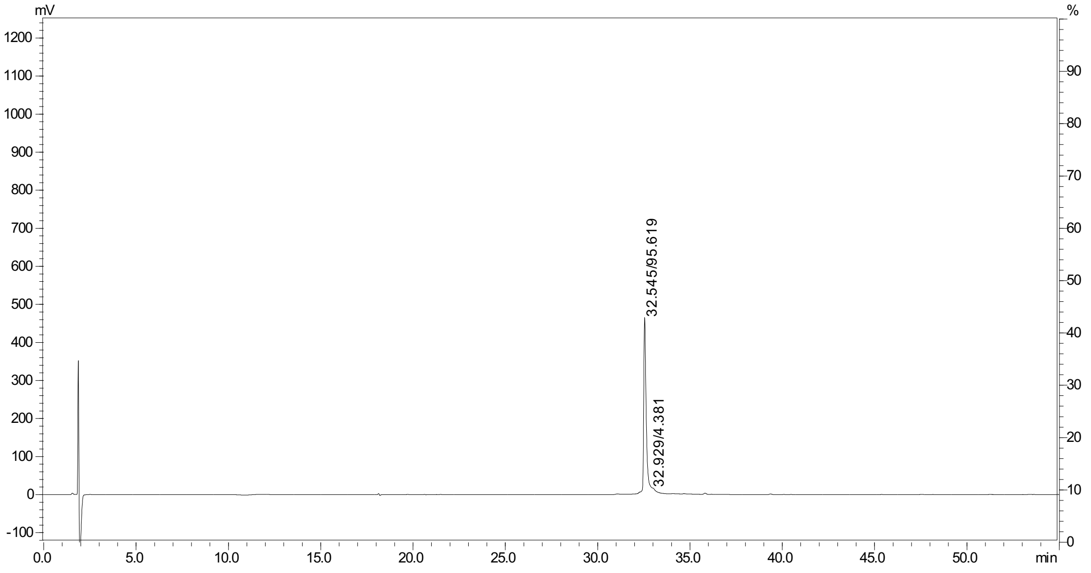

HR-MS

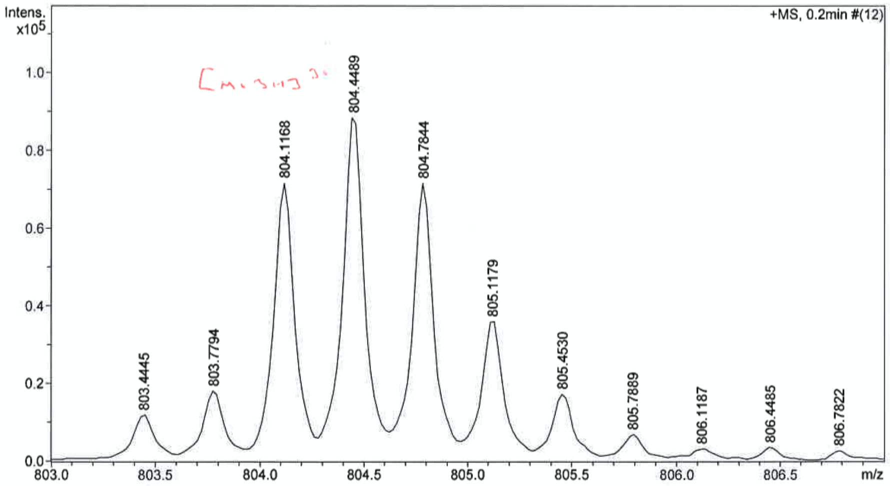

Formula	z	m/z	Meas. m/z	err [ppm]	err [mDa]
C 117 H 183 N 28 O 25 S	3+	804.1204	804.1168	4.5	3.6

39 (T-STAR-diyne-$S_{7H}S_{7H}$)

FTU-Ahx-AKS_{7H}YAR(Nle)GRS_{7H}LKEIAK-NH_2

20 min HPLC

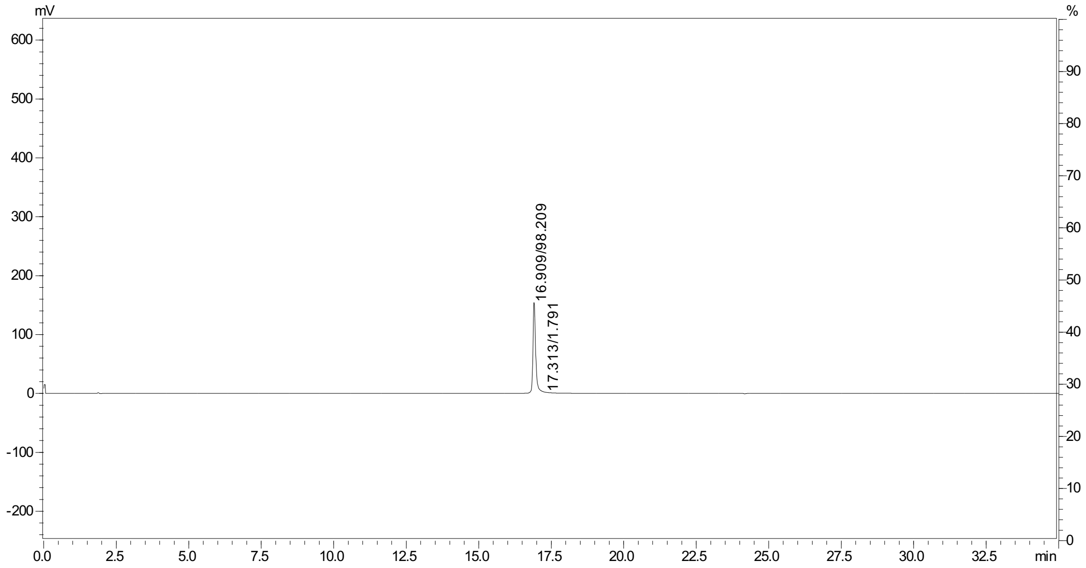

50 min HPLC

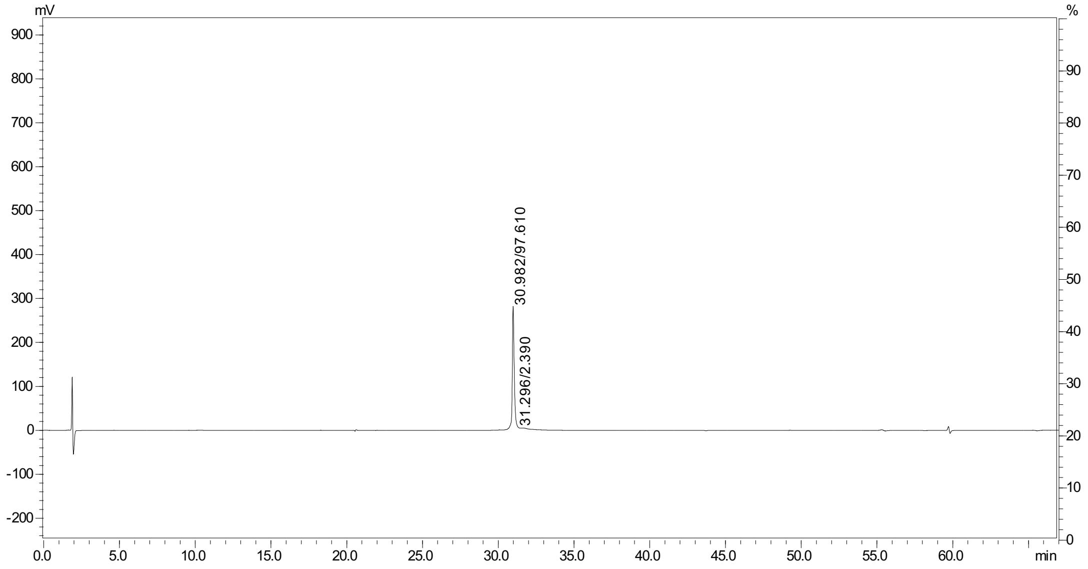

HR-MS

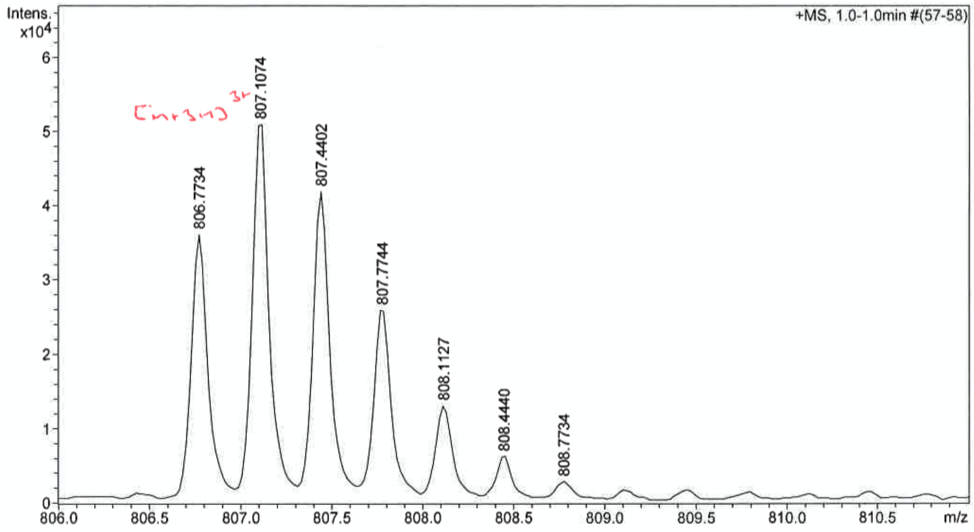

Formula	z	m/z	Meas. m/z	err [ppm]	err [mDa]
C 118 H 179 N 28 O 25 S	3+	806.7767	806.7734	4.1	3.3

40 (Ac-Native T-STAR)

Ac-AKAYAR(Nle)GRALKEIAK-NH_2

20 min HPLC

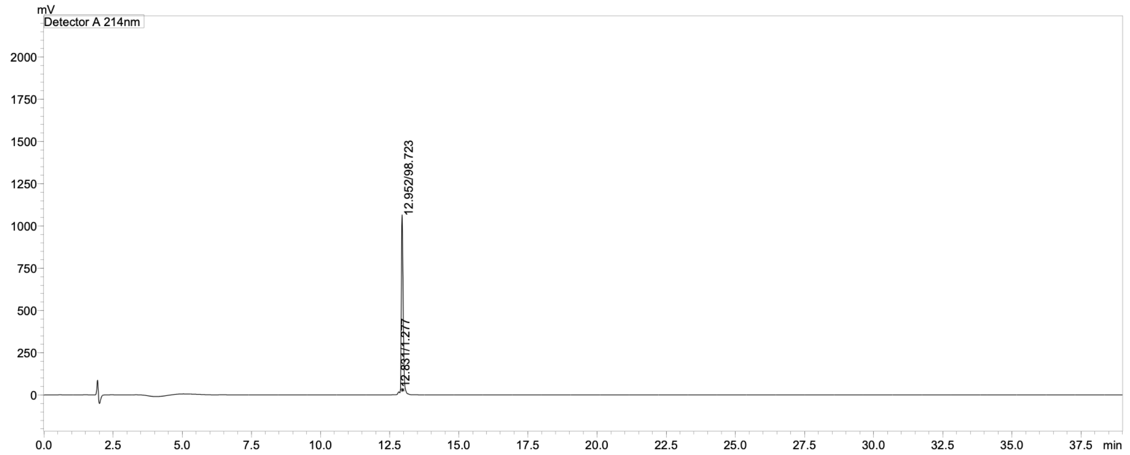

50 min HPLC

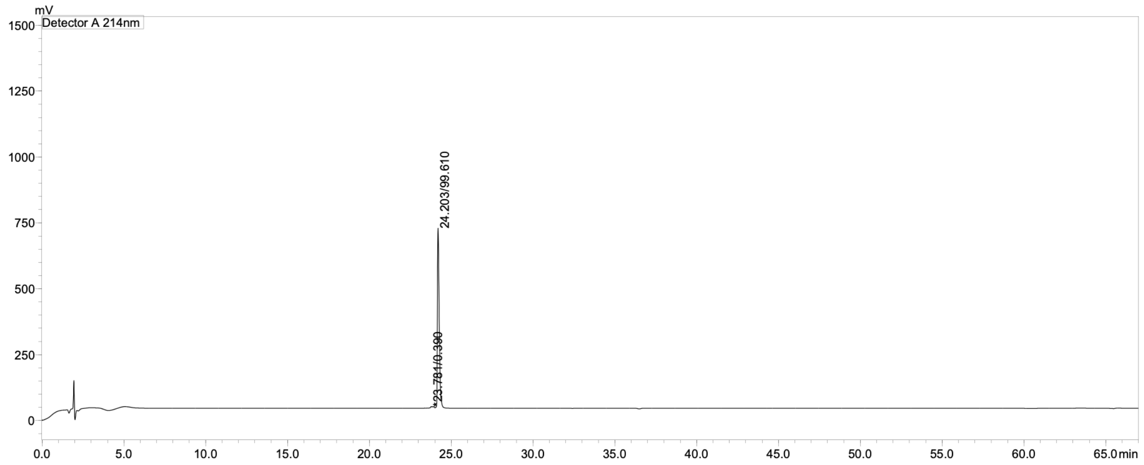

HR-MS

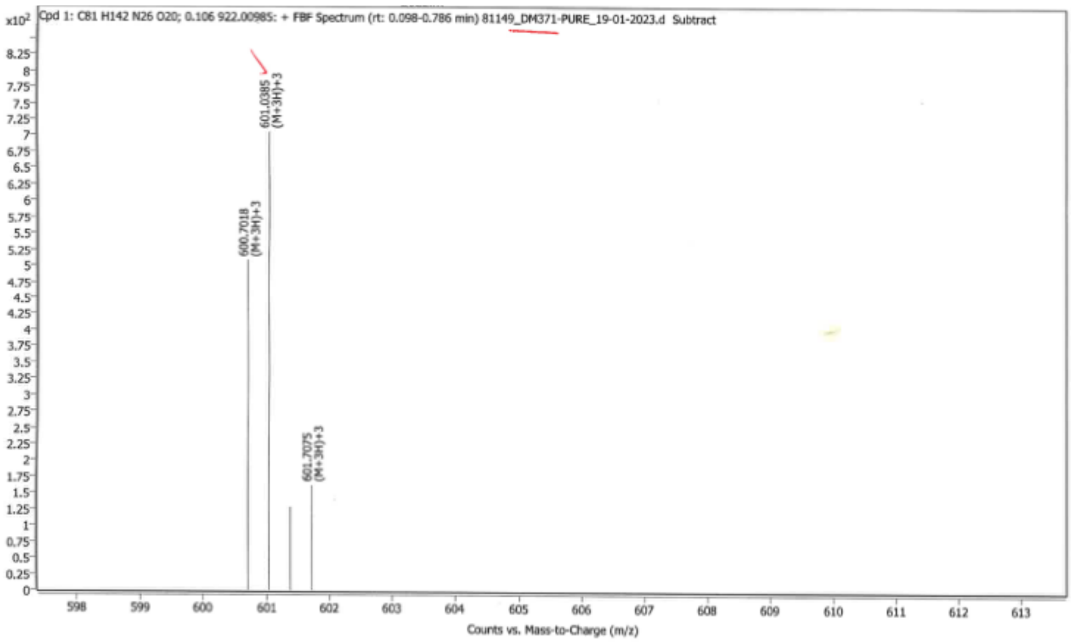

41 (Ac-T-STAR-diyne-S_7S_7)

Ac-AKS_7YAR(Nle)GRS_7LKEIAK-NH_2

20 min HPLC

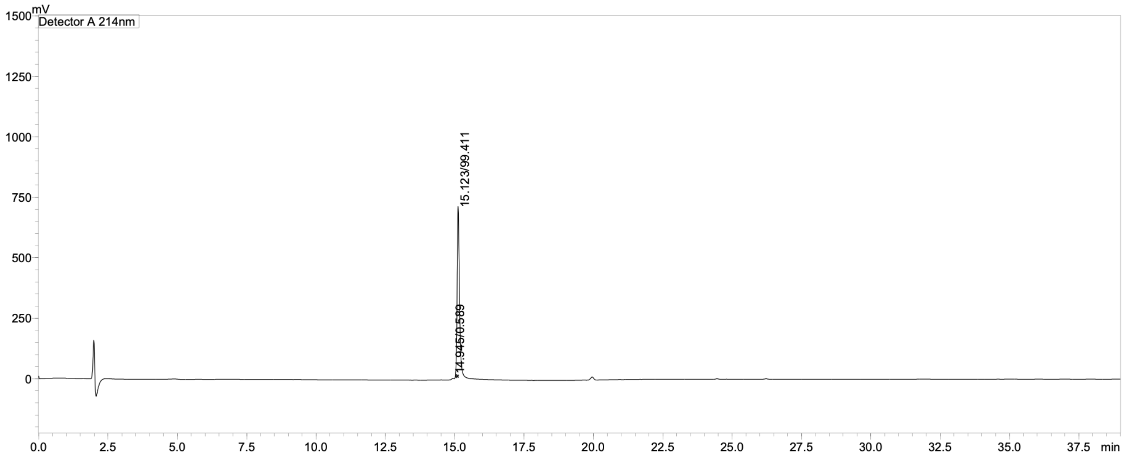

50 min HPLC

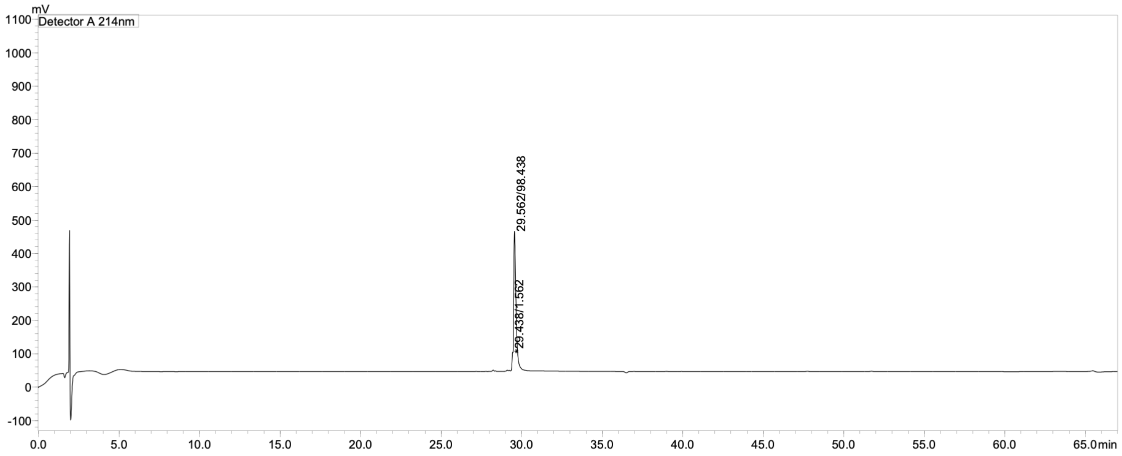

HR-MS

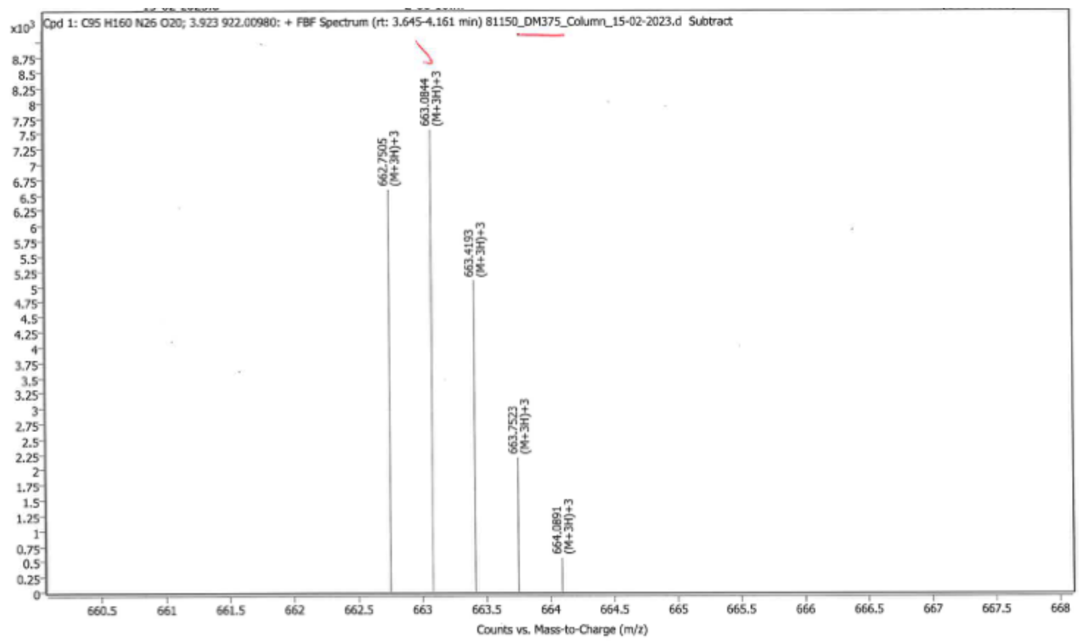

Supporting Information Tables and Figures

Table S2. Synthesis of the iodo electrophiles with varying carbon chain length.

Starting Material	Product	Yield (%)
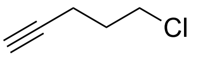	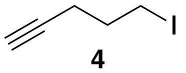 **4**	78
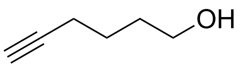	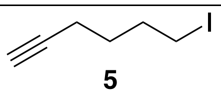 **5**	86
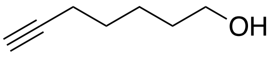	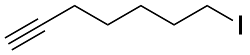 **6**	96
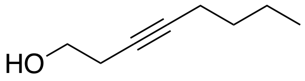	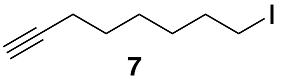 **7**	96

Circular Dichroism Data for all T-STAR Peptide Analogues

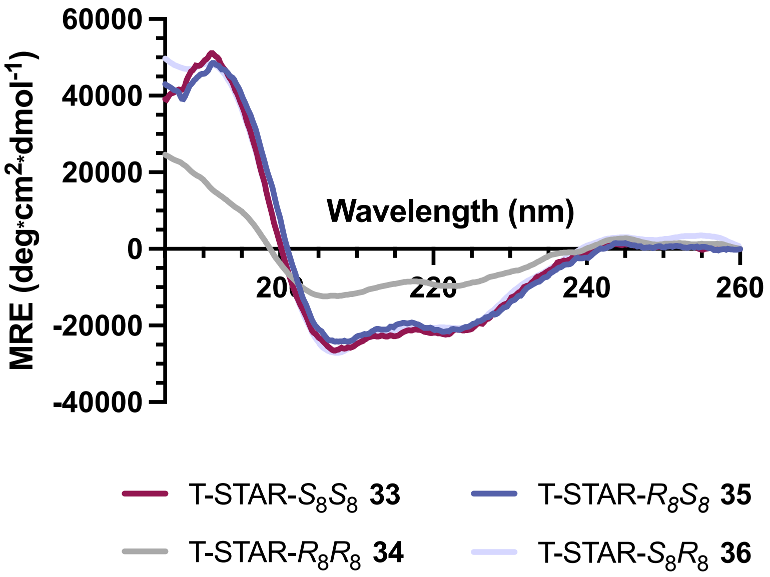

Figure S1. Circular dichroism spectra of stapled peptides **33**-**36**. Conditions: peptides 100 µM in PBS, pH 7.4. Spectra recorded between 190 and 260 nm.

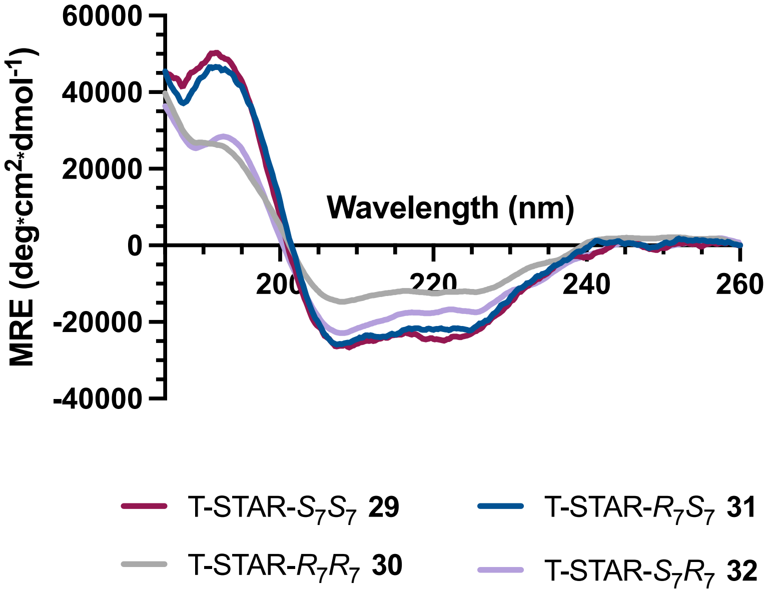

Figure S2. Circular dichroism spectra of stapled peptides **29**-**32**. Conditions: peptides 100 µM in PBS, pH 7.4. Spectra recorded between 190 and 260 nm.

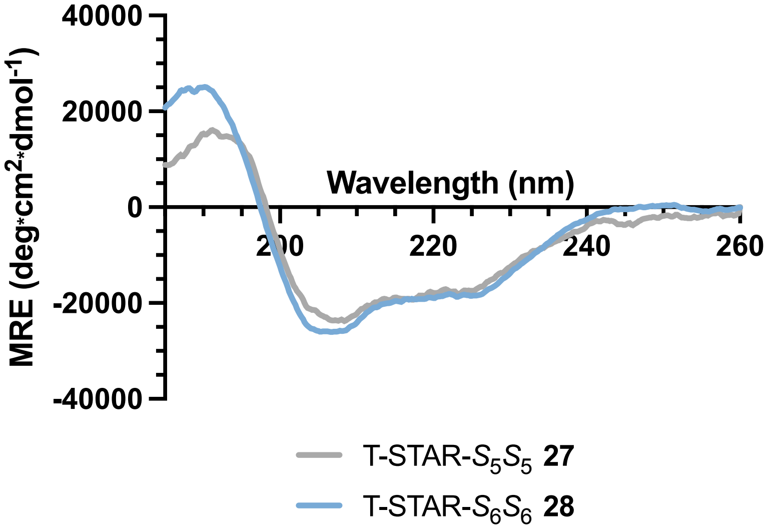

Figure S3. Circular dichroism spectra of stapled peptides **27** and **28**. Conditions: peptides 100 µM in PBS, pH 7.4. Spectra recorded between 190 and 260 nm.

Fluorescence Microscopy Images for all T-STAR Peptide Analogues

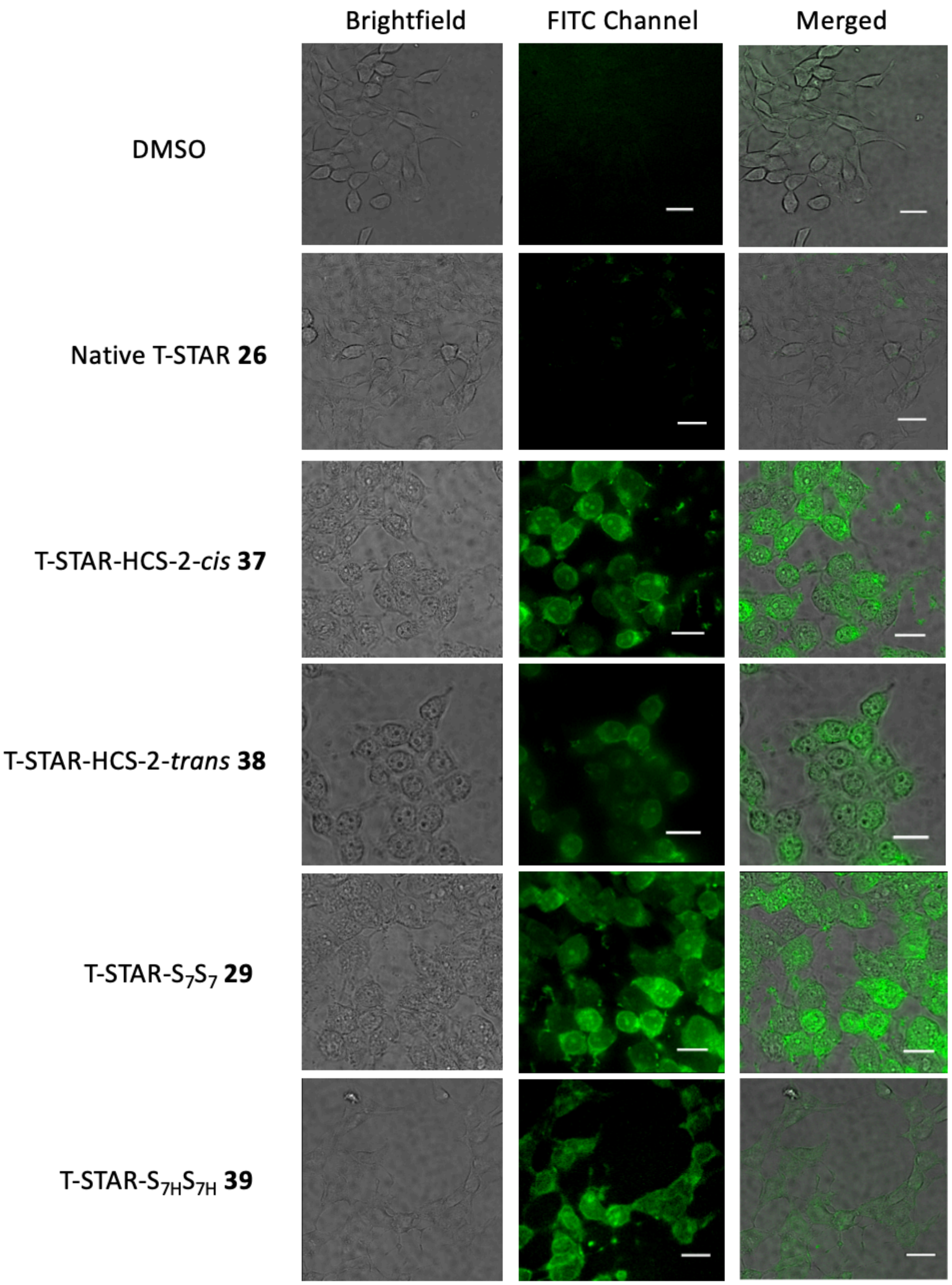

Figure S4. Fluorescence microscopy imaging showing the Brightfield channel (left), Fluorescein excitation channel at 495 nm (middle) and overlaid (right) images for the T-STAR peptide analogues incubated with HEK293 cells. Microscopy images were acquired with a custom-built multi-modal microscope setup. Images were processed using MetaMorph software. Scale bars = 40 µm, n = 2.
